# A systematic literature review of reviews on techniques for physical activity measurement in adults: a DEDIPAC study

**DOI:** 10.1186/s12966-017-0636-2

**Published:** 2018-02-08

**Authors:** Kieran P. Dowd, Robert Szeklicki, Marco Alessandro Minetto, Marie H. Murphy, Angela Polito, Ezio Ghigo, Hidde van der Ploeg, Ulf Ekelund, Janusz Maciaszek, Rafal Stemplewski, Maciej Tomczak, Alan E. Donnelly

**Affiliations:** 1 Department of Sport and Health Science, Athlone Institute of Technology, Athlone, Ireland; 2 University School of Physical Education in Poznan, Poznan, Poland; 3 Division of Endocrinology, Diabetology and Metabolism, Department of Internal Medicine, University of Turin, Corso Dogliotti 14, 10126 Torino, Italy; 4 School of Health Science, University of Ulster, Newtownabbey, UK; 5 National Institute for Food and Nutrition Research, Rome, Italy; 6 Department of Public and Occupational Health, VU University Medical Center, EMGO Institute for Health and Care Research, Amsterdam, The Netherlands; 7 Sydney School of Public Health, University of Sydney, Sydney, Australia; 8 Medical Research Council (MRC) Epidemiology Unit, University of Cambridge, Cambridge, UK; 9 The Department of Sport Medicine, Norwegian School of Sport Sciences, Oslo, Norway; 10 Department of Physical Education and Sport Sciences, Health Research Institute, University of Limerick, Limerick, Ireland

**Keywords:** Physical activity, Measurement, Assessment, Accelerometry, Questionnaires, Self-report, Motion sensors, Pedometers, Heart rate monitors, Adults

## Abstract

The links between increased participation in Physical Activity (PA) and improvements in health are well established. As this body of evidence has grown, so too has the search for measures of PA with high levels of methodological effectiveness (i.e. validity, reliability and responsiveness to change). The aim of this “review of reviews” was to provide a comprehensive overview of the methodological effectiveness of currently employed measures of PA, to aid researchers in their selection of an appropriate tool. A total of 63 review articles were included in this review, and the original articles cited by these reviews were included in order to extract detailed information on methodological effectiveness.

Self-report measures of PA have been most frequently examined for methodological effectiveness, with highly variable findings identified across a broad range of behaviours. The evidence-base for the methodological effectiveness of objective monitors, particularly accelerometers/activity monitors, is increasing, with lower levels of variability observed for validity and reliability when compared to subjective measures. Unfortunately, responsiveness to change across all measures and behaviours remains under-researched, with limited information available.

Other criteria beyond methodological effectiveness often influence tool selection, including cost and feasibility. However, researchers must be aware of the methodological effectiveness of any measure selected for use when examining PA. Although no “perfect” tool for the examination of PA in adults exists, it is suggested that researchers aim to incorporate appropriate objective measures, specific to the behaviours of interests, when examining PA in free-living environments.

**Electronic supplementary material:**

The online version of this article (10.1186/s12966-017-0636-2) contains supplementary material, which is available to authorized users.

## Background

Physical inactivity is the fourth leading cause of death worldwide [1]. Despite this, PA levels of adults across developed nations remain low and the promotion of regular participation in PA is a key public health priority [2]. Population level PA surveillance relies upon having tools to accurately measure activity across all population sub-groups. In addition to surveillance, it is essential that valid, reliable and sensitive measures of PA are available to practitioners, researchers and clinicians in order to examine the effectiveness of interventions and public health initiatives. The accurate measurement of PA in adults has relevance not only for refining our understanding of PA-related disorders [3], but also for defining the dose-response relationship between the volume, duration, intensity and pattern of PA and the associated health benefits.

A number of methods are available for the assessment of PA [4]. When selecting a measurement technique, researchers and practitioners need to consider not only feasibility and practicality of the measure, but also the methodological effectiveness, such as the validity, reliability and sensitivity. Validity refers to the degree to which a test measures what it is intended to measure, and is most often investigated by comparing the observed PA variables determined by the proposed measure with another comparable measure [5]. Criterion validity is when a measure is validated against the ‘gold standard’ measure. Good agreement between the proposed method and the gold standard provides some assurance that the results are an accurate reflection of PA behaviour. Other frequently examined forms of validity are concurrent validity (when two measures that give a result that is supposed to be equal are compared) and construct validity (when two measures that are in the same construct are compared). Reliability refers to the degree to which a test can produce consistent results on different occasions, when there is no evidence of change, while sensitivity is the ability of the test to detect changes over time [5].

In addition to methodological effectiveness, other factors need to be considered when selecting a method for assessing PA and interpreting the findings derived from these methods. Feasibility often drives the selection of the study measures. Some measures are more feasible than others depending on the setting, number of participants and cost. For example, the use of activity monitors to estimate PA may be less feasible in epidemiological studies where large numbers of individuals are being assessed. Reactivity may mean that the act of measuring PA may change a person’s behaviour: for example, being observed for direct observation [6] or wearing an activity monitor may cause the participant to alter their habitual PA behaviour [7]. When using self-report measures, social desirability may result in over-reporting of PA among participants keen to comply with the intervention aims [8]. These factors require careful consideration when selecting methods for assessing PA.

Although methods for the measurement of PA have been extensively examined, reviews to date have focused on specific categories of methods (i.e. self-report questionnaires [9–11], specific techniques i.e. Doubly Labelled Water (DLW) [12], smart phone technology [13], motion sensors and heart rate monitors (HRM) [14], pedometers [15] or a comparison of two or more methods [16]). Some reviews looked exclusively at specific PA behaviours (e.g. walking) [17] or focused solely on validity and/or reliability issues [18–20]. Other reviews have concentrated on methods for assessing PA in population subgroups (e.g. individuals with obesity [21] or older adults) [22–30]. Due to the level of variability in how information on measurement properties has been presented, and due to the wide range of different measures examined in existing reviews, it is extraordinarily difficult for researchers to compare and contrast measures of PA in adult populations.

The purpose of this article is to review existing reviews (a *review of reviews*) that have examined the methodological effectiveness of measures of PA. To aid in the comparison of measurement properties between different PA measures, original papers referred to within each review article were sourced, and additional analysis of these references was completed to enable better comparison and interpretation of findings. This review of reviews (as it will be referred to for the remainder of this article) is intended to provide a concise summary of PA measurement in adults. This work was completed as a component of the European DEDIPAC (DEterminants of DIet and Physical ACtivity) collaboration.

## Methods

### Literature search and search strategy

A systematic search of the electronic databases PubMed, ISI Web of Science, CINAHL, PsycINFO, SPORTDiscus and EMBASE took place in April 2014. The search strategy was developed by two of the authors from examining existing literature reviews, whereby common terminology utilised by published systematic reviews of specific methodologies or narrative reviews of all methodologies were included [4, 5, 31–35]. The developed search strategy was reviewed and agreed on by all members of the review team. The electronic databases were searched for the terms “Physical Activity” AND “Review OR Meta-Analysis” AND “Self-report” OR “Logs” OR “Diaries” OR “Questionnaire” OR “Recall” OR “Objective” OR “Acceleromet^*^” OR “Activity Monitor^*^” OR “Motion Sensor^*^” OR “Pedom^*^” OR “Heart Rate Monitor*” or “Direct Observation” AND “Valid^*^” OR “Reliab^*^” OR “Reproducib^*^” OR “Sensitiv^*^” OR “Responsiv^*^”. The search terms and criteria were tailored for each specific electronic database to ensure consistency of systematic searching. Only articles that were published in peer reviewed journals in the English language and were included in this review.

### Eligibility for inclusion

Although DLW is suggested as the gold standard measure of energy expenditure [36], it has not been included in the search strategy, as its feasibility for use in population surveillance research is limited due to its high cost and participant invasiveness [34]. Due to similar limitations, indirect calorimetry has also not been included in this search strategy. However, reviews that discuss studies which have examined the validity of PA measures against DLW and indirect calorimetry were included. The term Global Positioning System (GPS) was not included as it was felt that the limitations associated with GPS used alone [37] deemed it an inappropriate measure of PA for population surveillance in its current form.

Review articles that focused solely on the methodological effectiveness of measures of PA in clinical populations and in children/adolescents were not included in this review. Reviews identified in this study which described the methodological effectiveness of measures of PA in both adult and youths were included, but only the adult data extracted and included.

### Article selection

**Fig. 1:**
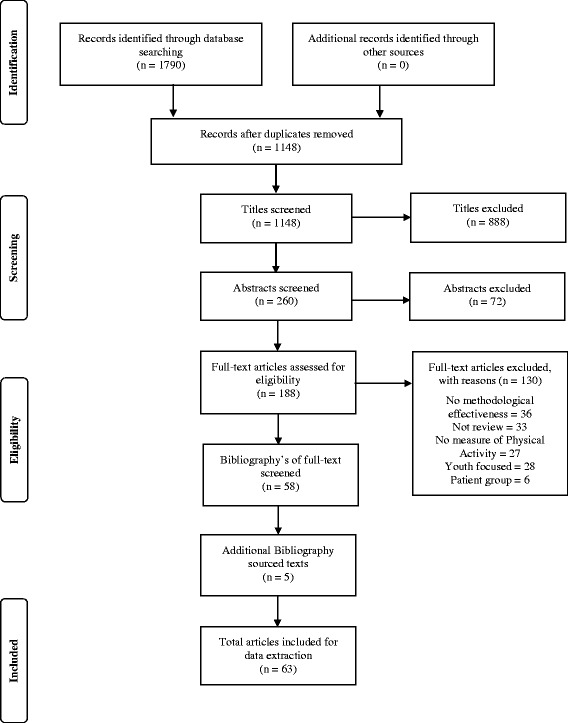
PRISMA flow diagram for search and inclusion process for identification of review articles

A single reviewer screened all article titles, with only articles that were clearly unrelated to the review of reviews removed at this level. Two independent reviewers examined the article abstracts. Results were collated and reported to a third reviewer, who made the final decision in the case of conflicting results. The full texts of included articles were reviewed by two reviewers using the same protocol for handling conflicting results. Reference lists of identified articles were reviewed to ensure that no relevant articles were overlooked. The collated list of accepted reviews was examined by three leading PA measurement experts, who identified key reviews they felt were not included. The full screening protocol was repeated for all supplementary articles identified (Fig. 1).

### Quality assessment

The methodological quality of the systematic reviews was evaluated using the Assessment of Multiple Systematic Reviews (AMSTAR) quality assessment tool [38]. No similar quality assessment tool exists for narrative reviews. The AMSTAR protocol was applied to each article by two researchers with any conflicting results resolved by a third reviewer.

### Data extraction

Initially, the full text and the reference list of each review article meeting the inclusion criteria was screened by a single reviewer for all references to methodological effectiveness, and each methods paper was sourced, screened and all relevant data extracted. The extracted data included general information about the article, the specific measure of PA examined and the demographic characteristics, including the sample population age, size and gender.

Finally, all relevant information relating to properties of methodological effectiveness (i.e. reliability, validity and sensitivity) was recorded. This included the key methodological details of the study and all relevant statistics used to examine measures of methodological effectiveness.

### Data synthesis

Data synthesis was conducted separately for each of the PA measurement methods, including general recommendations of the method and its effectiveness indicators. The results extracted from the methods papers were presented in the following order: Validity data is presented as mean percentage difference (MPD) in modified forest plots. Similar to Prince and colleagues (2008), where possible, the MPD was extracted or calculated from the original articles as (((Comparison Measure – Criterion Measure)/Criterion Measure)*100) [16]. Data points positioned around the 0 mark suggest high levels of validity compared to the reference measure. Data points positioned to the left of the 0 mark suggest an underestimation of the variable in comparison to the reference measure. Data points positioned to the right of the 0 mark suggest an overestimation of the variable in comparison to the reference measure. The further away from the 0 mark the point is positioned, the greater the under/overestimation. Data points 250% greater than or less than the reference measure were capped at 250%, and are marked with an asterisk. Due to the lack of reporting of variance results, and the use of differing and incompatible measurement units, confidence intervals are not reported.

## Results

### Study selection

The literature search produced 260 potentially relevant abstracts for screening, of which 58 were included in the review following abstract and full text review. After consultation from three international PA experts, and from bibliography review, a further 5 articles were identified for inclusion, providing a total of 63 articles for data extraction (Fig. 1) [4–7, 9–11, 13–19, 21–35, 39–72].

### Quality assessment

For this article, reviews were categorised as either “Narrative Reviews” or “Systematic Reviews”. A systematic review was defined as a review which described a search strategy for identification of relevant literature. Of the 63 articles, 41 were categorised as narrative reviews, while 22 were identified as systematic reviews. Findings of the AMSTAR quality assessment and review are described in Table 1. The mean AMSTAR score across the 22 articles was 5.4 (out of a possible score of 11), with three articles achieving a score of 3, four articles scoring 4, six articles scoring 5, four articles scoring 6, two articles scoring 7, two articles scoring 8 and one article achieving a score of 9 (Table 1). Based on AMSTAR categorisation, three reviews were considered low quality, 16 reviews were of medium quality and three reviews were considered high quality. The predominant measures examined/discussed in the identified review articles were activity monitors (n=44; 70%), self-report measures (n=28; 44%), pedometers (n=23; 37%) and HRM (n=18; 29%). Other measures included combined physiologic and motion sensors, multi-physiologic measures, multiphasic devices and foot pressure sensors. These measures were incorporated under the combined sensors heading.

**Table 1:** Details of the identified reviews, including AMSTAR quality assessment information

Author and Date	Physical Activity Measures of Interest that were Examined	Population Focus	Review Type	AMSTAR
Ainsle et al. (2003) [12]	1. Heart Rate Monitoring 2. Questionnaires and Activity Recalls 3. Pedometers 4. Uniaxial Accelerometers/Activity Monitors 5. Triaxial Accelerometers/Activity Monitors 6. Combined Heart Rate and Motion Sensors	Adult and Youth (age not specified)	Narrative Review	Not Appropriate
Andrew et al. (2010) [72]	1. Questionnaires and Activity Recalls	Adult (age not specified)	Non-Systematic Review	1. Y 2. CA 3. Y 4. N 5. N 6. N 7. N 8. N 9. N 10. N 11. Y Score = 3
Bassett (2000) [20]	1. Heart Rate Monitoring 2. Pedometers 3. Accelerometers/Activity Monitors	Adult and Youth (age not specified)	Narrative Review	Not Appropriate
Bassett et al. (2008) [17]	1. Pedometers 2. Accelerometers/Activity Monitors 3. Direct Observation 4. Questionnaires	Adults (age not specified)	Narrative Review	Not Appropriate
Berlin et al. (2006) [39]	1. Pedometers 2. Accelerometers/Activity Monitors	Adults and Youth (age not specified)	Narrative Review	Not Appropriate
Bonomi & Westerterp (2012) [21]	1. Pedometers 2. Accelerometers/Activity Monitors 3. Multi-site Activity Monitors	Adults (age not specified)	Narrative Review	Not Appropriate
Bort-Roig et al. (2014) [13]	1. Smartphone Technology	Adults (age not specified)	Systematic Review	1. Y 2. Y 3. Y 4. N 5. N 6. Y 7. N 8. N 9. N 10. N 11. Y Score = 5
Butte et al. (2012) [40]	1. Pedometers 2. Load Transducers 3. Accelerometers/Activity Monitors 4. Heart Rate Monitors 5. Combined Heart Rate and Motion Sensors	Adults and Youth (age not specified)	Narrative Review	Not Appropriate
Chen & Bassett (2005) [41]	1. Accelerometers/ Activity Monitors	Adults and Youth (age not specified)	Narrative Review	Not Appropriate
Cheung et al. (2011) [30]	1. Accelerometers/Activity Monitors	Adults/Older Adults (>17 years)	Systematic Review	1. Y 2. N 3. Y 4. N 5. N 6. Y 7. N 8. N 9. N 10. N 11. Y Score = 4
Corder et al. (2007) [42]	1. Accelerometers/Activity Monitors 2. Pedometers 3. Combined Heart Rate and Motion Sensors	Adults and Youth (age not specified)	Narrative Review	Not Appropriate
Davidson & deMorton (2007) [43]	1. Self-reported Human Activity Profile	Adults (age not specified)	Systematic Review	1. Y 2. Y 3. Y 4. Y 5. N 6. N 7. N 8. N 9. N 10. N 11. Y Score = 5
DeLany (2012) [44]	1. Accelerometers/Activity Monitors	Adults and Youth (age not specified)	Narrative Review	Not Appropriate
Dishman et al. (2001) [6]	1. Direct Observation 2. Questionnaires and Activity Recalls 3. Heart Rate Monitoring 4. Pedometers 5. Accelerometers/Activity Monitors	Adults (age not specified)	Narrative Review	Not Appropriate
Forsen et al. (2010) [29]	1. Self-Administered Physical Activity Questionnaires	Older Adults (mean age > 55 years.)	Systematic Review	1. Y 2. CA 3. Y 4. N 5. N 6. N 7. Y 8. Y 9. CA 10. N 11. Y Score = 5
Freedson & Miller (2000) [14]	1. Pedometers 2. Uniaxial Accelerometers/Activity Monitors 3. Triaxial Accelerometers/Activity Monitors 4. Heart Rate Monitors	Adults and Youth (age not specified)	Narrative Review	Not Appropriate
Garatachea et al. (2010) [27]	1. Accelerometers/Activity Monitors	Older Adults (age not specified)	Narrative Review	Not Appropriate
Gorman et al. (2014) [28]	1. Accelerometers/Activity Monitors	Older Adults (mean age ≥ 65 years or median age >60 years)	Systematic Review	1. Y 2. Y 3. Y 4. N 5. N 6. Y 7. N 8. N 9. Y 11. N 12. Y Score = 6
Haskell et al. (2000) [45]	1. Self-report surveys, Diaries, Physical Activity Logs, Recall Surveys, Retrospective Quantitative History, Global self-report 2. Heart Rate Monitors 3. Accelerometers/Activity Monitors	Adults and Youth (age not specified)	Narrative Review	Not Appropriate
Helmerhorst et al. (2012) [31]	1. Physical Activity Questionnaires	Adults and Youth (age not specified)	Systematic Review	1. Y 2. Y 3. Y 4. Y 5. N 6. Y 7. N 8. N 9. NA 10. N 11. Y Score = 6
Kim et al. (2013) [18]	1. International Physical Activity Questionnaire	Adults (15-69 years)	Systematic Review (Meta-analysis)	1. Y 2. Y 3. Y 4. N 5. N 6. Y 7. Y 8. Y 9. Y 10. N 11. Y Score = 8
Kowalski et al. (2012) [32]	1. Self-reported diaries, physical activity logs, physical activity questionnaires, physical activity surveys 2. Accelerometers/Activity Monitors 3. Pedometers 4. Heart Rate Monitoring 5. Direct Observation	Older Adults (mean age > 65 years)	Systematic Review	1. Y 2. Y 3. Y 4. Y 5. N 6. Y 7. Y 8. Y 9. NA 10. N 11. Y Score = 8
Kwak et al. (2011) [19]	1. Physical Activity Questionnaires (assess occupational physical activity)	Adults (age not specified)	Systematic Review	1. Y 2. Y 3. Y 4. N 5. N 6. Y 7. Y 8. Y 9. NA 10. N 11. Y Score = 7
Lamonte & Ainsworth (2001) [46]	1. Accelerometers/Activity Monitors 2. Pedometers 3. Physical Activity Questionnaires, Records, Logs and Recalls 4. Heart Rate Monitoring	Adults (age not specified)	Narrative Review	Not Appropriate
Lee et al. (2011) [47]	1. International Physical Activity Questionnaire (short form)	Adults and Youth (age not specified)	Systematic Review	1. Y 2. N 3. Y 4. N 5. N 6. Y 7. N 8. N 9. NA 10. N 11. Y Score = 4
Levine (2005) [48]	1. Heart Rate Monitoring 2. Physical Activity Recalls, Logs 3. Pedometers 4. Accelerometers/Activity Monitors	Adults and Youth (age not specified)	Narrative Review	Not Appropriate
Liu et al. (2012) [49]	1. Accelerometers/Activity Monitors 2. Foot Pressure Sensors 3. Heart Rate Monitoring 4. Armbands (Consisting of heat flux, galvanic skin response and skin temperature	Adults and Youth (age not specified)	Narrative Review	Not Appropriate
Lowe & O’Laighin (2014) [50]	1. Accelerometers/Activity Monitors	Adults and Youth (age not specified)	Narrative Review	Not Appropriate
Mathie et al (2004) [51]	1. Accelerometers/Activity Monitors	Adults and Youth (age not specified)	Narrative Review	Not Appropriate
Matthews (2005) [52]	1. Accelerometers/Activity Monitors	Adults (age not specified)	Narrative Review	Not Appropriate
Meyer et al. (2009) [24]	1. Physical Activity Recall Questionnaires, Surveys, Records, Diaries 2. Accelerometers/Activity Monitors 3. Pedometers	Older Adults (mean age >60 years)	Systematic Review	1. Y 2. CA 3. Y 4. N 5. N 6. Y 7. Y 8. Y 9. NA 10. N 11. N Score = 5
Murphy (2009) [25]	1. Accelerometers/Activity Monitors	Older Adults (age not specified)	Narrative Review	Not Appropriate
Neilson et al. (2008) [10]	1. Physical Activity Recall Questionnaires, Surveys, Records.	Adults (≥ 19 years)	Systematic Review	1. Y 2. CA 3. Y 4. N 5. N 6. Y 7. Y 8. Y 9. NA 10. N 11. N Score = 6
Pedišić et al. (2014) [53]	1. Accelerometers/Activity Monitors	Adults and Youth (age not specified)	Narrative Review	Not Appropriate
Pennathur et al. (2003) [23]	1. Physical Activity Diaries, Questionnaires 2. Accelerometers/Activity Monitors	Older Adults (age not specified)	Narrative Review	Not Appropriate
Pierannunzi et al. (2013) [54]	1. Behavioural Risk Factor Surveillance System	Adults (age not specified)	Systematic Review	1. Y 2. N 3. Y 4. N 5. N 6. N 7. Y 8. N 9. NA 10. N 11. Y Score = 4
Plasqui & Westerterp (2007) [33]	1. Accelerometers/Activity Monitors	Adults and Youth (age not specified)	Systematic Review	1. Y 2. CA 3. N 4. N 5. N 6. Y 7. N 8. N 9. NA 10. N 11. Y Score = 3
Plasqui et al. (2013) [55]	1. Accelerometers/Activity Monitors	Adults and Youth (age not specified)	Systematic Review	1. Y 2. N 3. N 4. N 5. N 6. Y 7. N 8. N 9. NA 10. N 11. Y Score = 3
Prince et al. (2008) [16]	1. Physical Activity Diaries, Logs, Questionnaires, Surveys and Recall interviews 2. Accelerometers/Activity Monitors 3. Pedometers 4. Heart Rate Monitoring 5. Direct Observation	Adults (mean age > 18 years)	Systematic Review	1. Y 2. Y 3. Y 4. Y 5. N 6. Y 7. Y 8. Y 9. Y 10. N 11. Y Score = 9
Reilly et al. (2008) [56]	1. Accelerometers/Activity Monitors	Adults and Youth (age not specified)	Narrative Review	Not Appropriate
Reiser & Schlenk (2009) [34]	1. Physical Activity Diaries, Logs, Questionnaires, Surveys and Recall interviews 2. Direct Observation 3. Pedometers 4. Accelerometers/Activity Monitors 5. Heart Rate Monitors	Adults and Youth (age not specified)	Narrative Review	Not Appropriate
Ridgers & Fairclough (2011) [57]	1. Accelerometers/Activity Monitors	Adults and Youth (age not specified)	Narrative Review	Not Appropriate
Sallis & Saelens (2000) [9]	1. Physical Activity Diaries, Logs, Questionnaires, Surveys and Recall interviews	Adults and Youth (age not specified)	Narrative Review	Not Appropriate
Schutz et al. (2001) [58]	1. Heart Rate Monitors 2. Accelerometers/Activity Monitors	Adults and Youth (age not specified)	Narrative Review	Not Appropriate
Shephard (2003) [59]	1. Physical Activity Diaries, Logs, Questionnaires, Surveys and Recall interviews	Adults and Youth (age not specified)	Narrative Review	Not Appropriate
Shephard and Aoyagi (2012) [4]	1. Direct Observation 2. Physical Activity Diaries, Logs, Questionnaires, Surveys and Recall interviews 3. Pedometers 4. Uniaxial Accelerometers/Activity Monitors 5. Triaxial Accelerometers/Activity Monitors 6. Mutiphasic Devices 7. Heart Rate Monitoring 8.Multi Physiologic Measures	Adults and Youth (age not specified)	Narrative Review	Not Appropriate
Strath et al. (2013) [60]	1. Accelerometers/Activity Monitors	Adults, Older Adults and Youth (age not specified)	Narrative Review	Not Appropriate
Trost et al. (2005) [7]	1. Accelerometers/Activity Monitors	Adults and Youth (age not specified)	Narrative Review	Not Appropriate
Tudor-Locke & Myers (2001) [15]	1. Pedometers	Adults and Youth (age not specified)	Narrative Review	Not Appropriate
Tudor-Locke & Rowe (2012) [61]	1. Pedometers	Adults (age not specified)	Systematic Review	1. Y 2. CA 3. Y 4. N 5. N 6. Y 7. N 8. N 9. NA 10. N 11. Y Score = 4
Tudor-Locke et al. (2002) [62]	1. Pedometers	Adults and Youth (age not specified)	Systematic Review	1. Y 2. CA 3. Y 4. Y 5. N 6. Y 7. N 8. N 9. NA 10. N 11. Y Score = 5
Tudor-Locke et al. (2004) [63]	1. Pedometers	Adults and Youth (age not specified)	Systematic Review	1. Y 2. CA 3. Y 4. Y 5. N 6. Y 7. N 8. N 9. NA 10. N 11. Y Score = 5
Valanou et al. (2006) [64]	1. Physical Activity Diaries, Logs, Recall Questionnaires, Quantitative History Questionnaires, Global self-report questionnaires 2. Direct Observation 3. Accelerometers/Activity Monitors 4. Pedometers 5. Heart Rate Monitoring	Adults and Youth (age not specified)	Narrative Review	Not Appropriate
van Poppel et al. (2010) [11]	1. Physical Activity Questionnaires	Adults (Mean age 18-55 years)	Systematic Review	1. Y 2. CA 3. Y 4. Y 5. N 6. Y 7. Y 8. Y 9. NA 10. N 11. Y Score = 7
Van Remoortel et al. (2012) [65]	1. Accelerometers/Activity Monitors	Adults (Mean age >18 years)	Systematic Review	1. Y 2. Y 3. Y 4. N 5. N 6. Y 7. N 8. N 9. Y 10. N 11. Y Score = 6
Vanhees et al. (2005) [66]	1. Accelerometers/Activity Monitors 2. Pedometers 3. Heart Rate Monitoring 4. Combined Heart Rate and Movement Sensors 5. Physical Activity Diaries, Logs, Questionnaires, Surveys and Recall interviews	Adults and Youth (age not specified)	Narrative Review	Not Appropriate
Warren et al. (2010) [5]	1. Physical Activity Diaries, Logs, Questionnaires, Surveys and Recall interviews 2. Accelerometers/Activity Monitors 3. Heart Rate Monitoring 4. Pedometers	Adults and Youth (age not specified)	Narrative Review	Not Appropriate
Washburn (2000) [22]	1. Selected self-reported measures of physical activity	Older Adults (age not specified)	Narrative Review	Not Appropriate
Washburn et al. (2000) [67]	1. Selected self-reported measures of physical activity	Adults and Youth (age not specified)	Narrative Review	Not Appropriate
Welk (2005) [68]	1. Accelerometers/Activity Monitors	Adults and Youth (age not specified)	Narrative Review	Not Appropriate
Westerterp (2009) [69]	1. Direct Observation 2. Physical Activity Diaries, Logs, Questionnaires, Surveys and Recall interviews 3. Heart Rate Monitoring 4. Accelerometers/Activity Monitors	Adults and Youth (age not specified)	Narrative Review	Not Appropriate
Westerterp & Plasqui (2004) [70]	1. Accelerometers/Activity Monitors	Adults and Youth (age not specified)	Narrative Review	Not Appropriate
Yang & Hsu (2010) [71]	1. Accelerometers/Activity Monitors	Adults and Youth (age not specified)	Narrative Review	Not Appropriate

The AMSTAR [38] tool was used to score the reviews as follows: 1. Was an ‘a priori’ design provided? 2. Was there duplicate study selection and data extraction? 3. Was a comprehensive literature search performed? 4. Was the status of publication (i.e. grey literature) used as an inclusion criterion? 5. Was a list of studies (included and excluded) provided? 6. Were the characteristics of the included studies provided? 7. Was the scientific quality of the included studies assessed and documented? 8. Was the scientific quality of the included studies used appropriately in formulating conclusions? 9. Were the methods used to combine the findings of studies appropriate? 10. Was the likelihood of publication bias assessed? 11. Was conflict of interest reported? Answers: Y=Yes; N=No; CA=Can’t answer; NA=Not applicable

### Data extraction

#### Self-report measures

##### Validity

*Criterion validity*: A total of 35 articles examined the criterion validity of self-reported measures by comparison to DLW determined energy expenditure [73–107]. Self-reported measures of PA included 7 day recall questionnaires, past year recall questionnaires, typical week questionnaires and PA logs/diaries were validated against 8-15 days of DLW measurement (Additional file 1: Table S1).

**Fig. 2:**
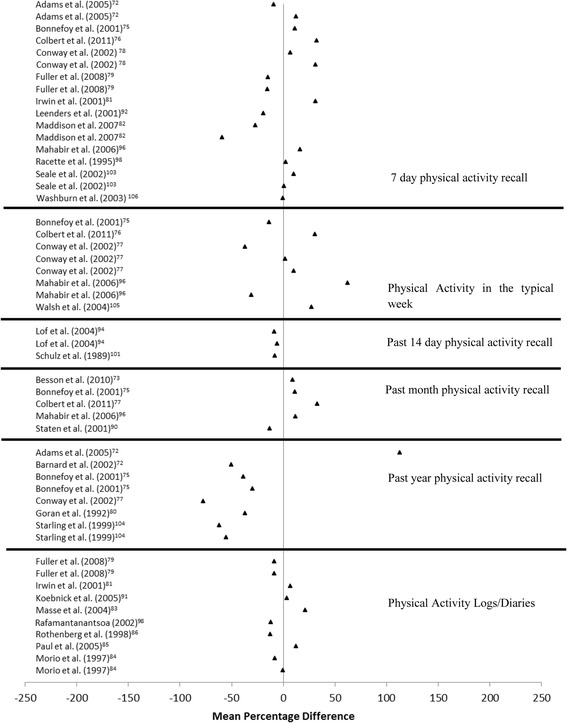
Forest plot of percentage mean difference between self-reported energy expenditure (TEE, PAEE, PAL) compared to criterion measure of energy expenditure (doubly labelled water)

The mean values for self-reported and criterion determined PA energy expenditure were available for the calculation of MPD in 27 articles [73–87, 91–93, 95, 97, 99, 100, 102, 104–107]. Energy expenditure was calculated from a range of behaviours, including leisure time PA, work based PA and PA frequency. The MPD between self-reported PA energy expenditure (time spent in PA normally converted to energy expenditure using a compendium of PA) is presented in Fig. 2. The MPDs observed in studies that examined the validity of PA diaries ranged from -12.9% to 20.8%. MPD for self-reported PA energy expenditure recalled from the previous 7 days (or typical week) were larger, ranging from -59.5% to 62.1%. MPDs from self-reported PA energy expenditure for the previous month compared to DLW determined energy expenditure ranged from -13.3% to 11.4%, while the difference between self-reported PA from the previous twelve months and DLW determined energy expenditure ranged from -77.6% to 112.5%.

*Concurrent validity:* A total of 89 articles reported on concurrent validity of self-reported measures [75, 80, 83, 84, 97, 102, 108–190]. Articles were collated based on the types of referent measures used (Additional file 1: Table S2). The MPD between self-reported energy expenditure and energy expenditure from PA log/diaries for 12 studies ranged from -67.6% to 23.8% (Additional file 1: figure S2a) [80, 108, 110, 111, 128, 145, 152, 157, 159, 160, 169, 175]. These findings suggest that self-report underestimates energy expenditure compared to activity logs/diaries. Seven studies compared self-reported time spent in specific activity intensities with PA intensities from logs/diaries (Additional file 1: figure S2a) [109, 120, 121, 146, 152, 182, 187]. A wider MPD range (-69.0% to 438.5%) was evident, with the greatest differences occurring for moderate intensity physical activity (MPA) and vigorous intensity physical activity (VPA) [109, 120, 121].

Eight studies compared two different self-reported measures of PA energy expenditure [80, 83, 97, 152, 158, 162, 175, 190], and and 6 studies compared two different self-reported measures of time spent in PA [112, 135, 136, 146, 152, 153, 158] (Additional file 1: figure S2b). Additional file 1: figure S2c presents 15 studies that compared self-reported PA energy expenditure with PA energy expenditure from activity monitors [80, 132, 142–144, 150, 159, 168, 170, 172, 174, 178, 183, 185, 191]. The MPD ranged from -74.7% to 82.8%, with self-reported measures tending to overestimate energy expenditure.

Self-reported time spent in light intensity physical activity (LIPA) (n=6) [75, 119, 131, 146, 179, 189], MPA (n=17) [75, 115, 119, 130, 131, 133, 134, 139–141, 146, 147, 161, 163, 176, 177, 187, 189] and moderate-to-vigorous intensity physical activity (MVPA) (n=7) [115, 116, 127, 145, 149, 153, 179, 192] was validated against activity monitors that mainly employed count-to-activity thresholds to determine PA intensity (Additional file 1: figure S2d), with the MPD for LIPA ranging from -70.1% to 129.2%, MPA ranging from -78.9% to 1007.6% and MVPA ranging from -34.9% to 217.1%. The MPD for VPA was also validated against activity monitors (Additional file 1: figure S2e) [75, 115, 119, 130, 131, 133, 134, 140, 141, 146, 147, 161, 163, 177, 187, 189], with all studies identifying an overestimation of self-reported VPA (Additional file 1: figure S2e).

The concurrent validity of additional self-reported variables, including total PA [163, 181, 184, 193], frequency of MVPA [149], active time [151, 161], time standing [192] and time stepping [192] were also compared to activity monitor determined variables (Additional file 1: figure S2e).

The MPD between self reported energy expenditure and both pedometer and HRM determined energy expenditure [80, 102, 123, 142, 194]; and self-reported time spent in PA intensities and HRM determined time spent in PA intensities [118, 129, 146, 154, 174, 195] are presented in Additional file 1: figure S2f. Self-reported energy expenditure overestimated pedometer determined energy expenditure (range=17.1% to 86.5%). Self-reported measures notably overestimated time spent in PA intensities when compared to HRM. Although self-reported energy expenditure underestimated HRM determined energy expenditure, this underestimation was small compared to other measures (-17.7% to -1.3%).

##### Reliability

*Intra-instrument reliability:* One article examined the intra-instrument reliability of a self-reported measure of PA [196]. A self-reported instrument examining the previous 14 days of PA was administered [196]. After 3 days, the instrument examined the PA of the same 14 day period. The findings identified high levels of intra-instrument reliability for total activity (ICC=0.90; 95% CI=0.86-0.93), MPA (ICC=0.77; 95% CI=0.69-0.84), VPA (ICC=0.90; 95% CI=0.86-0.93), walking (ICC=0.89; 95% CI=0.85-0.93) and energy expenditure (ICC=0.86; 95% CI=0.80-0.90) (Additional file 1: Table S3).

*Test-retest reliability:* The test-retest reliability of self-reported measures was examined in 116 studies [75, 77, 83, 110, 116, 117, 122, 125–127, 129, 131, 132, 135, 137, 140, 144, 145, 147, 149, 152, 153, 155, 157, 159, 161, 162, 167–169, 172, 175, 176, 178–181, 184, 187, 188, 190, 191, 196–269]. Due to the wide test-retest periods, articles were allocated to one of 5 periods, ≤1 week (Additional file 1: Table S4a), >1 - <4 weeks (Additional file 1: Table S4b), >4 - <8 weeks (Additional file 1: Table S4c), >8 weeks - <1 year (Additional file 1: Table S4d) and >1 year (Additional file 1: Table S4e). Test-retest statistics employed were extracted and are presented in Table 2. An overview of all identified studies examining the test-retest reliability of PA/energy expenditure measured by self-report, along with all test-retest statistics is provided in Additional file 1: Table S4a-e.

**Table 2:** Descriptive statistics for reliability of self-reported measures of physical activity across specified time periods

Duration of recall	Correlation Coefficient	Kappa	ICC
≤ 1 week	0.25 – 0.99	0.32 – 0.87	0.30 – 0.99
1 – 4 weeks	0.13 – 0.96	0.40 – 1.00	0.27 – 0.99
5 – 8 weeks	0.41 – 0.99	NA – 0.69	NA
≤ 1 year	0.25 – 0.95	0.54 – 0.82	0.62 – 0.92
> 1 year	0.17 – 0.41	0.20 – 0.85	0.14 – 0.93

*ICC* Intraclass Correlation Coefficient, *NA* No data Available

*Sensitivity*: Two studies examined the sensitivity of self-reported measures to detect change in PA behaviours over time [256, 270]. Both studies identified small to moderate effect sizes for specific PA behaviours over a six month period in older adults (Additional file 1: Table S5).

#### Activity monitors

##### Validity

*Criterion validity*: Fifty-eight articles examined the criterion validity of activity monitor determined PA variables [73, 77, 80, 96, 105, 119, 271–323]. The majority of articles compared activity monitor determined energy expenditure with DLW [73, 77, 80, 96, 105, 274, 275, 277, 278, 280, 281, 285, 292, 293, 295, 296, 300, 303–305, 311, 313, 317, 323], while activity monitor determined steps [119, 271, 283, 287, 289, 298, 299, 306, 307, 314, 315, 318], distance travelled [282] and activity type [272, 273, 276, 279, 284, 286, 288, 290, 291, 294, 297, 301, 302, 308–310, 312, 316, 319–322] were also compared to direct observation (Additional file 1: Table S6).

**Fig. 3:**
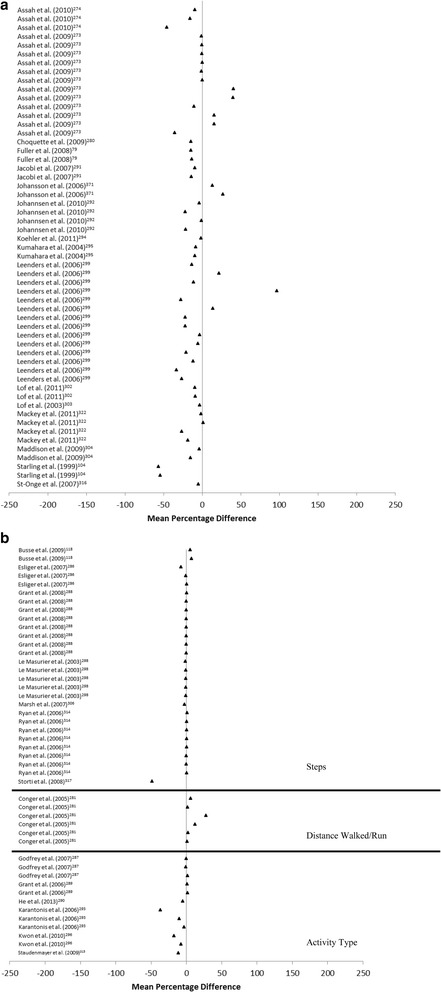
**a** Forest plot of percentage mean difference between accelerometer determined energy expenditure (TEE, PAEE, PAL) compared to criterion measure of energy expenditure (doubly labelled water). **b** Forest plot of percentage mean difference between accelerometer determined steps, distance walked and activity type compared to criterion measure of direct observation

The range of MPD observed in studies that examined the criterion validity of activity monitor determined energy expenditre ranged from -56.59% to 96.84% (Fig. 3a). However, a trend was apparent for activity monitor determined energy expenditure to underestimate the criterion measure. The range of MPD between activity monitor and direct observation determined steps was smaller, with values ranging from -48.52% to 7.47%, with 96% of studies having a MPD between -10% to 10% (Fig. 3b). Activity monitors overestimated distance walked/run (0.88% to 27.5%). Activity monitors also tended to underestimate activity classification, with MPD varying between -36.67% to 2.00%.

*Concurrent validity:* A total of 103 articles examined the concurrent validity of activity monitor measures of PA [73, 77, 80, 119, 146, 151, 174, 192, 194, 195, 262, 271, 282, 295, 305, 316, 324–409]. Data extractions were grouped by the types of measures used (Additional file 1: Table S7).

The MPD of activity counts from two different activity monitors ranged from -40.6% to 13.2% [262, 327, 351, 389, 392, 405]. The MPD for a wide range of activity behaviours from two different activity monitors were examined; LIPA (-12.5% - 13.7%) [146, 340, 392, 405], MPA (-10.9% - 3.1%) [146, 340], VPA (-9.7% - 20.3%) [146, 352], MVPA (-57.5% - 3.3%) [344, 392, 405], total PA (1.1%) [146]. Stepping [151, 192] and step counts [77, 119, 340, 405] were compared between 2 activity monitor devices (MPD ranged from -21.7% - 0% for step counts and -57.1% - 56% for stepping). Energy expenditure estimated by two activity monitors were compared [372, 404, 408], with MPD ranging from -21.1% - 61% (Additional file 1: figure S3c).

Energy expenditure at different PA intensities from activity monitors were compared against estimates from indirect calorimetry and whole room calorimetry. For LIPA, the MPD ranged from -79.8% - 429.1% [349, 394]. For MPA, MPD ranged from -50.4% - 454.1% [349, 395], while estimates for VPA ranged from -100% - 163.6%. Energy expenditure estimates from activity monitoring devices for total PA were compared against indirect calorimetry estimates [368, 394, 396, 398, 404], where MPD ranged from -41.4% to 115.7%. The MPD range for activity monitor determined total energy expenditure compared with whole-room calorimetry were narrower (-16.7% to -15.7%) [343, 364] (Additional file 1: figure S3d).

Activity monitor estimates of energy expendture were compared to HRM estimates of energy expenditure for total PA (-10.4% - 22.2%) [80, 402], for LIPA (-75.4% - 72.8%) [146], for MPA (49.2% - 677.7%), VPA (-46.2% - 46.2%) [146, 361] and for total time spent in PA (-16.1% - 34.9%) [146, 174]. Self-reported measures were used to examine the concurrent validity of activity monitors for energy expenditure [80] and total time spent in PA [174], with MPD ranging from -6.0% - 32.1% (Additional file 1: figure S3e).

Estimated energy expenditure was compared between activity monitors and indirect calorimetry (kcal over specified durations; Additional file 1: figure S3f (-68.5% - 81.1%)) [282, 328, 341, 358, 367, 369, 370, 375, 376, 380, 382, 383, 385, 387]; (METs over specified durations; Additional file 1: figure S3g (-67.3% -- 48.4%)) [195, 325, 345–347, 349, 350, 353, 357, 362, 384, 397, 400, 407, 409]. A single study compared the estimated energy expenditure from 5 different activity monitors and indirect calorimetry at incremental speeds (54, 80, 107, 134, 161, 188 and 214 m.min^-1^) in both men and women (MPD ranged from -60.4% - 90.8%) (Additional file 1: figure S3h) [374].

##### Reliability

*Inter-instrument reliability*: The inter-instrument reliability of activity monitoring devices (e.g. the reliability of the same device worn by the same participant over the same time period) was examined in 18 studies [301, 315, 337, 344, 370, 385, 387, 406, 409–418]. Study methodologies included the wearing of devices over the left and right hip [337, 370, 385, 387, 406, 413, 415, 417], over the hip and lower back [409], the wearing of devices side by side at the same location on the hip [301, 344, 411, 414, 416–418], devices worn at 3^rd^ intercostal space and just below the apex of the sternum [410], device worn on both wrists [412], worn on both legs [315] and worn side by side on the same leg [315]. Coefficients of variations ranged from 3% to 10.5% for the ActiGraph device [418] and from <6% to 35% for the RT3 accelerometer [387, 416]. All reported correlation coefficients were significant and greater than 0.56 [370, 385, 387, 406, 409, 412, 415, 417]. ICC values for the majority of devices were >0.90 [301, 315, 337, 344, 411, 413], excluding those observed for the RT3 accelerometer (0.72-0.95) [417], Actitrac (0.40 -0.87) and Biotrainer devices (0.60–0.71) [406] (Additional file 1: Table S8).

*Test-retest reliability:* Test-retest reliability of activity monitoring devices was examined in 26 studies [153, 155, 228, 234, 262, 282, 297, 314, 358, 385, 407, 411, 414, 416, 419–430]. For the laboratory-based studies, variables examined included distance walked [282], steps at different speeds [314, 420], resting periods [358], accelerometer counts [385, 407, 411, 414, 416, 425, 429, 430], energy expenditure [426] and postural position [297, 429]. For the free-living analyses, behaviours examined included activity behaviours [155, 419], accelerometer counts [262, 421, 422], step count [422], energy expenditure [228, 234] and the number of people achieving the recommended amount of PA [153] (Additional file 1: Table S9).

As the examination of PA over a number of days can be considered a measure of test-retest reliability, researchers have used statistical processes (i.e. generalizability theory or the Spearman Brown Prophecy formula) to determine the minimum number of days required to provide a reliability estimate of PA behaviours [431]. Studies reported that a minimum of three days of ActiGraph data are required to provide a reliable estimate of total PA [423] and time spent in MVPA [424], while a minimum of 2 days is required to provide a reliable estimate of ActiGraph determined steps per day, accelerometer counts per day and intermittent MVPA per day [427]. However, for the examination of continuous 10 minute bouts of MVPA (as suggested in the majority of international PA recommendations), a minimum of 6 days of measurement is required [427].

*Sensitivity*: The only study of responsiveness to change in activity monitors, using the ActiWatch, identified that this device was able to detect significant differences in activity counts accumulated between young adults and sedentary older adults and between active older adults and sedentary older adults [421]. However, no differences could be detected between the young adults and active older adults (Additional file 1: Table S10).

#### Pedometers

##### Validity

**Fig. 4:**
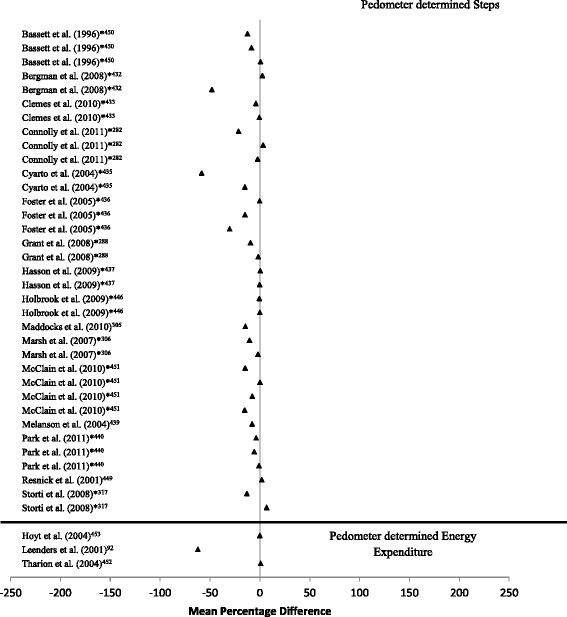
Forest plot of mean percentage difference between pedometer determined step count/energy expenditure compared to criterion measure (direct observation/doubly labelled water respectively). * denotes multiple devices compared in the same study

*Criterion validity:* A total of 30 studies were sourced that examined the criterion validity of step count in pedometer devices [283, 289, 298, 306, 307, 314, 318, 365, 391, 432–452], while 3 studies examined the criterion validity of pedometer determined energy expenditure compared to DLW [93, 453, 454]. Of the laboratory based studies assessing criterion validity, 30% used over ground walking protocols [307, 318, 365, 391, 442, 445–447, 450, 451] and the remaining treadmill-based protocols [283, 289, 298, 306, 314, 432–441, 443, 444, 448, 449] or a combination of the two [452]. In free-living studies which examined the criterion validity of pedometer determined energy expenditure, pedometers were worn for 2 [454], 7 [93] and 8 days [453] (Fig. 4; (-62.3% - 0.8%)). Pedometer determined step count was generally lower when compared to direct observation (-58.4% - 6.9%). Some studies also examined the effect of speed on pedometer output. Pedometers had relatively high levels of accuracy across all speeds, but appear to be more accurate at determining step-count at higher walking speeds compared to lower walking speeds (Additional file 1: Table S11) [306, 436, 438, 439].

*Concurrent validity:* The concurrent validity of pedometers was examined in a total of 22 articles [77, 194, 298, 376, 391, 399, 404, 422, 432–434, 441, 444, 448, 449, 451, 452, 455–459]. Various approaches were used to examine the concurrent validity of pedometers, with 14 studies comparing pedometer step count with steps determined from other pedometers [432, 451, 458] and activity monitors [77, 298, 391, 422, 433, 434, 444, 455–457, 459] and 4 studies comparing pedometer determined energy expenditure with energy expenditure determined from indirect calorimetry [376, 399, 404, 441, 448, 451] and/or energy expenditure determined from other activity monitors [451]. One study compared pedometer determined distance travelled with treadmill determined distance travelled [449], while one study compared pedometer determined MVPA with activity monitors determined MVPA [452] (Additional file 1: figure S4a). Pedometers appear to underestimate time spent in MVPA and estimated energy expenditure when compared to other measures. The findings are less clear for step count determined from pedometers when compared to other pedometers or activity monitors, with devices appearing to both over and underestimate step count (Additional file 1: Table S12).

##### Reliability

*Inter-instrument reliability:* A total of 6 articles examined the inter-instrument reliability of pedometer output obtained from 18 different devices [314, 315, 447, 449, 451, 457]. Many included articles examined the inter-instrument reliability of multiple devices in the same study (e.g. 2 pedometers [315], 5 pedometers [451], 10 pedometers [446, 449]). Inter-instrument reliability was examined by comparing pedometer outputs from two of the same model devices worn on the left and right hip [315, 449, 451, 457], on the left hip, right hip and middle back [447] and on the left and right hip and repeated with two further devices of the same model [446].

Three studies (1 examining the inter-instrument reliability of a single pedometer and 2 examining the inter-instrument reliability of multiple pedometers), identified that the majority of devices had acceptable levels of inter-instrument reliability (ICC ≥ 080) [446, 449, 457]. In the studies which examined the inter-instrument reliability of multiple devices, 8/10 pedometers [449] and 9/10 pedometers [446] achieved ICC ≥ 0.80. Using planned contrasts, Bassett and colleagues highlight that no significant differences were observed between devices worn on the left and right hip [451]. Two studies investigated the effect of walking speed on inter-instrument reliability, highlighting that ICC values increased as speed increased [315, 447] (Additional file 1: Table S13).

*Test-retest reliability:* A single laboratory-based test-retest reliability study in a laboratory-based treadmill protocol identified that the Yamax Digiwalker SW-200 (Tokyo, Japan) had appropriate test-retest reliability (ICC > 0.80 and significant) at 7 out of 11 treadmill speeds (non-significant speeds = 4, 20, 22 and 26 km.h^-1^) [314].

A total of 6 articles examined the reliability of pedometer steps obtained over a specified measurement period [423, 427, 460–463], presenting the minimum number of days of pedometer measurement to reliably estimate PA behaviours. The minimum number of days of measurement required for a reliable estimate (i.e. ICC >0.8) of pedometer steps was 2-4 days (Additional file 1: Table S14) [423, 427, 460–463].

*Sensitivity*: In the only study of pedometer responsiveness to change, effect size was used to examine the meaningfulness of difference between means [464]. A large effect size (>0.8) was observed, suggesting that pedometers, in this study, were sensitive to change (Additional file 1: Table S15).

#### Heart rate monitors

##### Validity

**Fig. 5:**
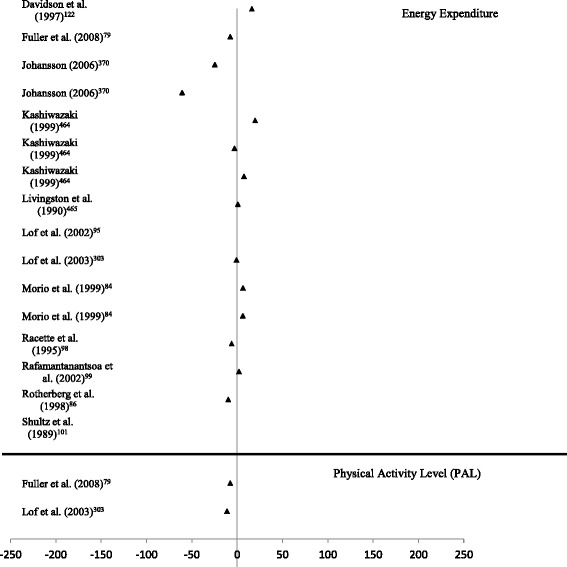
Forest plot of percentage mean difference between heart rate monitor determined energy expenditure/physical activity level compared to criterion measure (doubly labelled water)

*Criterion validity:* All 12 studies that examined the criterion validity of HRMs were unstructured, free-living protocols [80, 85, 87, 96, 99, 100, 102, 123, 304, 371, 465, 466]. The duration of monitoring for HRM ranged from 24 hours [102, 465] to 14 days [96, 371]. Two studies examined the validity of HRM determined physical activity levels (PAL) compared to DLW determined PAL. All remaining articles compared estimates of energy expenditure determined by HRM techniques with DLW determined energy expenditure. The flex heart rate methodology, which distinguishes between activity intensities based on heart rate versus VO_2_ calibration curves, were utilised in all studies using individual calibration curves. MPDs between HRM determined energy expenditure and DLW determined energy expenditure ranged from -60.8% - 19.7% across identified studies (Fig. 5). No clear trend for over/under estimation was apparent (MPDs for energy expenditure ranging between -60.8% - 19.7%). For PAL, a slight trend in underestimation was apparent (-11.1 to -7.6) (Additional file 1: Table S16).

*Concurrent validity:* The concurrent validity of HRM determined energy expenditure [80, 467–470], PAL [80] and PA intensity [146, 174] was examined using a range of measures, including direct/indirect calorimetry [467, 469, 470], activity monitoring [80, 146, 174, 401] and measures of self-reported PA [80, 174, 468] (Additional file 1: Table S17). A slight trend in overestimation of energy expenditure and PAL was observed (Additional file 1: figure S5a). For PA intensities, MPDs were larger and more variable, with MPA underestimated and VPA overestimated. The MPD between HRM determined LIPA and LIPA determined by the Tritrac and MTI activity monitors fell outside the range for the presented forest plot, with values of +306.4% and +367.2%, respectively [146] (Additional file 1: figure S5a). No articles sourced through the data extraction reported on the reliability or responsiveness to change of HRM.

#### Combined sensors

##### Validity

**Fig. 6:**
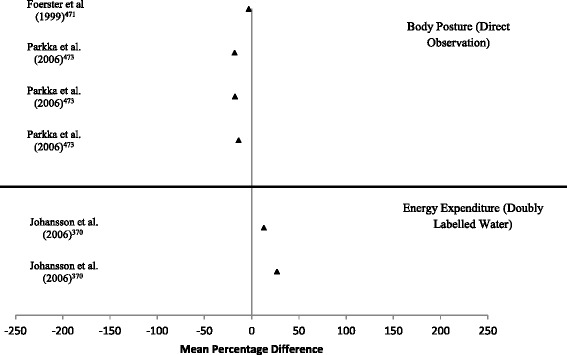
Forest plot of percentage mean difference between energy expenditure/body posture determined by combined sensors compared to criterion measure (doubly labelled water/direct observation)

*Criterion validity:* A total of 8 articles were identified that examined the criterion validity of multiple accelerometers [471–474] or accelerometers combined with gyroscopes [475] or HRMs [371, 476, 477]. The included studies had relatively small sample sizes, ranging from 3-31 participants. Studies primarily examined the effectiveness of data synthesis methodologies (i.e. Decision Tree Classification, Artificial Neural Networks, Support Vector Machine learning etc.) to identify specific postures/activities [471–477] or energy expenditure [371, 477]. Time spent in specific body postures/activity types tended to be underestimated from combined sensors when compared to direct observation (-33.3% to -3.2%; Fig. 6). In contrast, energy expenditure was overestimated by combined sensors when compared to DLW in free-living settings (13.0% to 26.8%) (Additional file 1: Table S18) [371].

*Concurrent validity:* Eleven studies examined the validity of combined accelerometry and HRM determined energy expenditure compared to whole room calorimetry [478–480] or indirect calorimetry [400, 477, 481–486] determined energy expenditure. No clear trend for under/overestimation was apparent, with combined sensors appearing to be relatively accurate in estimating energy expenditure when compared to indirect calorimetry in both a structured (-13.8% - 31.1%) and unstructured (0.13%) [485] settings (Additional file 1: Table S19). No articles sourced through the data extraction reported on the reliability or responsiveness to change of combined sensors.

## Discussion

To the authors’ knowledge, this is the first systematic literature review of reviews to simultaneously examine the methodological effectiveness of the majority of PA measures. The greatest quantity of information was available for self-reported measures of PA (198 data extraction points), followed by activity monitors (179 data extraction points), pedometers (52 data extraction points), HRMs (19 data extraction points) and combined sensors (18 data extraction points).

The criterion validity of measures was determined through the examination of energy expenditure via DLW and by direct observation of steps and PA behaviours. For accelerometry, although variability was lower, a substantial proportion of studies (44/54) underestimated energy expenditure compared to DLW when proprietary algorithms or count-to-activity thresholds were employed. Based on the amended forest plots for the criterion validity of measures of PA, a greater level of variability was apparent for self-reported measures compared to objective measures (Figs. 2–6). Limited data on the criterion validity of HRM and combined sensors determined energy expenditure was available. HRMs tended to underestimate DLW determined energy expenditure, while combined sensors often overestimated energy expenditure. Unfortunately, due to the lack of measures of variability, resulting in the absence of meta-analysis, it was not possible to describe the extent of differences between measures statistically. For step counts, both activity monitors and pedometers achieved high levels of criterion validity. When comparing the two, pedometers appeared to be less accurate than activity monitors at estimating step count, tending to underestimate steps when compared to direct observation. Activity monitors tended to slightly overestimate distance travelled, while time spent in each activity type (or posture) determined by both activity monitors and combined sensors was slightly underestimated when compared to direct observation (Fig. 3a and Fig. 6). For concurrent validity of all measure of PA, high levels of variability were observed across a wide range of activity behaviours. In particular, high levels of variability were apparent in the estimation of PA intensities, with VPA substantially overestimated in the majority of concurrent validations across all measures. In summary, objective measures are less variable than recall based measures across all behaviours, but high levels of variability across behaviours are still apparent.

For activity monitors and pedometers, acceptable inter-instrument reliability was observed in the majority of studies. Variability for inter-instrument reliability across different activity monitors and pedometers was apparent, with some instruments demonstrating better reliability compared to others. However, a detailed examination of study methodology, device wear locations and activities performed is necessary when interpreting the inter-instrument reliability of pedometers and activity monitors.

A wide range of values were reported for the test-retest reliability of self-reported measures, with apparent trends for reduced levels of test-retest reliability as the duration of recall increased. Researchers must be cognisant of potential differences in test-retest reliability due to duration between administrations and between PA behaviours assessed within each tool when selecting a self-reported measure of PA. Moderate to strong test-retest reliability was observed for activity monitors in free-living environments. However, the reliability of activity monitors attenuated as the duration between measurements increased. As expected, the test-retest reliability of different devices varied, while intensity of activity often had a significant effect. The test-retest reliability of pedometer determined steps in a laboratory setting was high across the majority of speeds, but the reliability appeared to weaken at higher speeds (e.g. 20, 22 and 26 km·h^-1^). Although moderate to strong test-retest reliability of both pedometers and activity monitors were apparent, researchers should be aware of differences between models and devices when selecting a measure for use. Furthermore consideration should be given to the duration between test and retest and the behaviour being assessed when considering test-retest reliability, as although a measure may be reliable for one output, it may not be reliable for all outcomes.

When examining PA in free-living environments, it is essential that sufficient data is gathered to ensure a reliable estimate is obtained [7, 431]. By determining the inter- and intra-individual variability across days of measurement, researchers can define the number of days of monitoring required to reliably estimate such behaviours. For activity monitors and pedometers, analysis has been conducted to estimate the minimum number of days of measurement required to provide a reliable estimate of PA behaviors. For activity monitors, two days of measurement are recommended for a reliable estimate of steps per day, accelerometer counts per day and intermittent MVPA per day measured, 3 days for a reliable estimate of total PA and time spent in MVPA and 6 days are required for a reliable estimate of continuous 10 minute bouts of MVPA. For pedometers, a minimum of 2-4 days of measurement was required to provide a reliable estimate of steps in older adults, while 2-5 days of measurement was required in adults. These findings highlight the importance of knowing what behaviours are to be examined prior to collecting objective data from free-living environments, to ensure that sufficient information is recorded to provide reliable estimates of the behaviours of interest.

The responsiveness of measures to detect change over time was the least reported property of measures of PA. When evaluating interventions, or indeed evaluating changes in PA behaviours in longitudinal research, it is critical to utilise measures that can detect such changes. Although validity and reliability are requirements for sensitivity/responsiveness to change [5], this does not imply that a measure is responsive to change simply because it is valid and reliable. Responsiveness to change must be evaluated, and not assumed. Currently, the research on the responsiveness to change for all types of PA measurement is at best limited. Substantial investigation into the responsiveness of PA measures to detect change is required to ensure that measures employed in future intervention and longitudinal research can detect meaningful change.

Although the validity, reliability and responsiveness to change are key when selecting a measure of PA and energy expenditure, other factors including feasibility and cost should be considered. For example, wearing several sensors around the body for a short period in a laboratory setting is often quite feasible, but prolonging the wear period for several days may be uncomfortable for participants, while reattachment of sensors may require specific and detailed training. The appropriateness of the measure for use in specific populations is critical. Activity monitors or HRMs may need to be attached to body locations that are visible and may be considered “embarrassing” for certain populations in free-living environments, likely resulting in lower compliance to wear protocols. Finally, while the cost of objective measures have reduced significantly and are now feasible for inclusion in large scale data collections (i.e. UK Biobank study, HELENA study), worn devices can be expensive to use in large populations, especially if recording needs to be concurrent, requiring 100’s or 1000’s of devices. Although these issues are often the dominant determinant for researchers when selecting a measure of PA, it is critical that researchers consider selecting the measure with the best validity, reliability and responsiveness to change available to them; a larger dataset with less valid measures may not always be superior to a smaller dataset.

The findings of this review have highlighted the substantial quantity of research which has focused on the validity, reliability and responsiveness to change of measures of PA. A substantial number of review articles have been conducted on the measurement of PA in adult populations. The majority of such reviews were not systematic in nature. Of the systematic reviews articles identified, the methodological quality (as assessed by the AMSTAR quality assessment tool) was relatively poor, with 3 reviews considered low quality, 16 articles considered medium quality and 3 articles considered high quality. An obvious increase in the quantity of research using objective measures of PA over the past number of decades is apparent. Unfortunately, with the enormous quantity of research on the methodological effectiveness of PA measures comes extreme variability in study design, data processing and statistical analysis conducted. Such variability makes comparison between measurement type and specific measurement devices/tools extremely difficult. The sometimes questionable study designs and research questions in some of the existing published literature is a reanalysis of “suitable” data, rather than from a study designed to collect data to answer a specific research questions. The authors propose that to aid researchers in making informed decisions on the best available measure of PA, the development of “best practise” protocols for study design and data collection, analysis and synthesis are required, which can be employed across all measures, providing comparable information that is easy for researchers from outside of the field to digest. The authors also propose that any future undertaking of reviews on the measurement of PA follow best practise, and ensure that the reviews conducted are of the highest possible quality. Such improvements will provide researchers with the best available evidence for making a decision on which measure of PA to employ.

## Strengths and limitations

This review of reviews had limitations that should be taken into account when considering the findings presented here. As this article reviewed existing literature reviews, and due to potential methodological errors within these reviews, it is likely that some relevant literature on the methodological effectiveness for measures of PA has been overlooked. Additionally, articles that have been published since the publication of each review will also have been overlooked. Due to the quantity of identified articles, and difficulties in contacting primary authors regarding articles published over the last 60 years, the primary data from these articles was not sourced. Although prior research has systematically reviewed the literature for accuracy of measures of PA, and some narrative reviews have compared the methodological effectiveness of different measures of PA, this is the first study to comprehensively examine and collate details on the validity, reliability and responsiveness to change of a range of measures of PA in adult populations. For researchers that are selecting a measure of PA, this will enable the comparison between different measures of PA within one article, rather than having to refer to a wide range of available literature that examines each single measure. Additionally, rather than focusing solely on information presented within each existing review of the literature, the original articles referred to within each review were sought and data was extracted independently.

## Conclusion

In general, objective measures of PA demonstrate less variability in properties of methodological effectiveness than self-report measures. Although no “perfect” tool for the examination of PA exists, it is suggested that researchers aim to incorporate appropriate objective measures, specific to the behaviours of interests, when examining PA in adults in free-living environments. Other criteria beyond methodological effectiveness often influence tool selection, including cost and feasibility. However, researchers must be cognisant of the value of increased methodological effectiveness of any measurement method for PA in adults. Additionally, although a wealth of research exists in relation to the methodological effectiveness of PA measures, it is clear that the development of an appropriate and consistent approach to conducting research and reporting findings in this domain is necessary to enable researchers to easily compare findings across instruments.

## Additional file

Additional file 1: Table S1. Criterion validity of self-reported measures of physical activity/energy expenditure. **Table S2**. Concurrent validity of self-reported measures of physical activity/energy expenditure. **Table S3**. Intra-instrument Reliability of self-reported measures of physical activity. **Table S4a**. Test-retest reliability of self-reported measures of physical activity/energy expenditure within a duration of less than or equal to one week. **Table S4b**. Test-retest reliability of self-reported measures of physical activity/energy expenditure within a duration of between 1 week and 4 weeks. **Table S4c**. Test-retest reliability of self-reported measures of physical activity/energy expenditure within a duration of between 4 weeks and 8 weeks. **Table S4d**. Test-retest reliability of self-reported measures of physical activity/energy expenditure within a duration of between 8 weeks and 1 year. **Table S4e**. Test-retest reliability of self-reported measures of physical activity/energy expenditure within a duration of greater than 1 year. **Table S5**. Sensitivity to change over time of self-reported measures of physical activity/energy expenditure. **Table S6**. Criterion validity of accelerometer activity monitor determined physical activity/energy expenditure. **Table S7**. Concurrent validity of accelerometer/activity monitor determined physical activity/energy expenditure. **Table S8**. Inter-instrument reliability of accelerometer/activity monitor determined physical activity/energy expenditure. **Table S9**. Test-retest reliability of accelerometer/activity monitor determined physical activity/energy expenditure. **Table S10**. Sensitivity to change over time of accelerometer devices. **Table S11**. Details of studies that examined the Criterion Validity of Pedometers. **Table S12**. Details of studies examining the concurrent validity of pedometers. **Table S13**. Details of studies examining inter-instrument reliability in pedometer devices. **Table S14**. Details of studies examining the test-retest reliability of pedometers. **Table S15**. Details of studies examining the sensitivity to change of pedometers. **Table S16**. Details of studies examining the criterion validity of heart rate monitoring devices. **Table S17**. Details of studies examining the concurrent validity of heart rate monitoring devices. **Table S18**. Details of studies examining the criterion validity of combined sensors. **Table S19**. Details of studies examining the concurrent validity of combined sensors. **Figure S1**. PRISMA Checklist. **Figure S2a**. Forest plot of percentage mean difference between self-report measures of energy expenditure compared to energy expenditure from activity logs/diaries. **Figure S2b**. Forest plot of percentage mean difference between self-report measures of energy expenditure and time spent in physical activity compared to other self-report measures of energy expenditure or time spent in physical activity. **Figure S2c**. Forest plot of percentage mean difference between self-report measures of energy expenditure compared to energy expenditure determined from activity monitors. **Figure S2d**. Forest plot of percentage mean difference between self-report measures of time spent in physical activity intensities (Light, Moderate and Moderate-to-Vigorous intensity physical activity) compared to time spent in physical activity intensities determined from activity monitors. **Figure S2e**. Forest plot of percentage mean difference between self-report measures of time spent in physical activity intensities (Vigorous physical activity, Total physical activity, times active, time standing, time stepping) compared to time spent in physical activity intensities determined from activity monitors. **Figure S2f**. Forest plot of percentage mean difference between self-report measures of energy expenditure and time spent in physical activity intensities (Vigorous physical activity, Total physical activity, times active, time standing, time stepping) compared to energy expenditure time spent in physical activity intensities determined from pedometers and heart rate monitors. **Figure S3c**. Forest plot of percentage mean difference between accelerometer/activity monitor determined variables (activity counts, time spent in light intensity physical activity, time spent in moderate intensity physical activity, time spent in moderate-to-vigorous intensity physical activity, time spent in vigorous intensity physical activity, total physical activity, stepping and energy expenditure) compared to an alternative accelerometer/activity monitor. **Figure S3d**. Forest plot of percentage mean difference between accelerometer/activity monitor determined energy expenditure (METs) in light intensity physical activity, moderate intensity physical activity, vigorous intensity physical activity and total physical activity (METs, MJ.d, KJ.h, KJ.kg.min^-1^) compared to estimates from indirect (IC) and whole room calorimetry (WRC). **Figure S3e**. Forest plot of percentage mean difference between accelerometer/activity monitor determined energy expenditure, energy expenditure from light intensity physical activity, moderate intensity physical activity, vigorous intensity physical activity, total physical activity compared to estimates from Heart Rate Monitoring (HRM) and Self-Report (SR) measures. **Figure S3f**. Forest plot of percentage mean difference between accelerometer/activity monitor determined energy expenditure (kcal.min^-1^, kcal.kg.hr^-1^) compared to indirect calorimetry determined energy expenditure (kcal.min^-1^, kcal.kg.hr^-1^). **Figure S3g**. Forest plot of percentage mean difference between accelerometer/activity monitor determined energy expenditure (METs.min^-1^, METs.hr^-1^) compared to indirect calorimetry determined energy expenditure (METs.min^-1^, METs.hr^-1^). **Figure S3h**. Forest plot of percentage mean difference between accelerometer/activity monitor determined total energy expenditure compared to indirect calorimetry determined total energy expenditure. **Figure S3h** (cont). Forest plot of percentage mean difference between accelerometer/activity monitor determined energy expenditure (kcal.min^-1^, kcal.kg.hr^-1^) compared to indirect calorimetry determined energy expenditure (kcal.min^-1^, kcal.kg.hr^-1^). **Figure S4a**. Forest plot of percentage mean difference between pedometer determined step count/energy expenditure/MVPA compared to concurrent measures (i.e. accelerometry, indirect calorimetry, pedometers). **Figure S5a**. Forest plot of percentage mean difference between heart rate monitor determined energy expenditure/physical activity level/physical activity intensity compared to concurrent measures (accelerometers, self-report, indirect calorimetry) **Figure S6**. Forest plot of percentage mean difference between energy expenditure determined by combined sensors compared to concurrent measure (indirect calorimetry). (DOCX 1304 kb)
