# Supplementary material for: A systematic literature review of reviews on techniques for physical activity measurement in adults: a DEDIPAC study
Source: Int J Behav Nutr Phys Act. 2018 Feb 8;15:15. doi: 10.1186/s12966-017-0636-2 (PMC5806271; doi:10.1186/s12966-017-0636-2)
Supplement: Supplementary file 1 — Criterion validity of self-reported measures of physical activity/energy expenditure. Table S2. Concurrent validity of self-reported measures of physical activity/energy expenditure. Table S3. Intra-instrument Reliability of self-reported measures of physical activity. Table S4a. Test-retest reliability of self-reported measures of physical activity/energy expenditure within a duration of less than or equal to one week. Table S4b. Test-retest reliability of self-reported measures of physical activity/energy expenditure within a duration of between 1 week and 4 weeks. Table S4c. Test-retest reliability of self-reported measures of physical activity/energy expenditure within a duration of between 4 weeks and 8 weeks. Table S4d. Test-retest reliability of self-reported measures of physical activity/energy expenditure within a duration of between 8 weeks and 1 year. Table S4e. Test-retest reliability of self-reported measures of physical activity/energy expenditure within a duration of greater than 1 year. Table S5. Sensitivity to change over time of self-reported measures of physical activity/energy expenditure. Table S6. Criterion validity of accelerometer activity monitor determined physical activity/energy expenditure. Table S7. Concurrent validity of accelerometer/activity monitor determined physical activity/energy expenditure. Table S8. Inter-instrument reliability of accelerometer/activity monitor determined physical activity/energy expenditure. Table S9. Test-retest reliability of accelerometer/activity monitor determined physical activity/energy expenditure. Table S10. Sensitivity to change over time of accelerometer devices. Table S11. Details of studies that examined the Criterion Validity of Pedometers. Table S12. Details of studies examining the concurrent validity of pedometers. Table S13. Details of studies examining inter-instrument reliability in pedometer devices. Table S14. Details of studies examining the test-retest reliability of pedometers. Table [file 12966_2017_636_MOESM1_ESM.docx]

**Supplementary Table 1:** Criterion validity of self-reported measures of physical activity/energy expenditure.

| **Author** | **Measure** | **Age Range** | **Sample Size** | **Sex** | **Reference** | **Results** | **Primary Source** |
| --- | --- | --- | --- | --- | --- | --- | --- |
| Adams et al. (2005)^[72]^ | Stanford 5 City Project Physical Activity Recall (S5CPPAR) | 49.1 ± 6.8 yrs. | 81 | F = 81 | S5CPPAR determined kcal.kg^-1^.d^-1^ – Doubly labelled water determined energy expenditure (kcal.kg^-1^.d^-1^).  14 days of DLW measurement compared to 7 days of PA recall from S5CPPAR (Unstructured). | Mean_DIFF_=1.51 ± 8.76 kcal.kg^-1^.d^-1^; p=0.14;  r=0.19; NS | Nielson (2009) |
| Adams et al. (2005)^[72]^ | Minnesota Leisure Time Physical Activity Survey (MLTPAS) | 49.1 ± 6.8 yrs. | 81 | F = 81 | MLTPAS determined kcal.kg^-1^.d^-1^ – Doubly labelled water determined energy expenditure (kcal.kg^-1^.d^-1^).  14 days of DLW and ActiGraph measurement compared to 7 days of PA recall from S5CPPAR (Unstructured). | Mean_DIFF_=13.62 ± 14.95 kcal.kg^-1^.d^-1^; p<0.0001  r=0.05; NS | Nielson (2009) |
| Adams et al. (2005)^[72]^ | 24 hour physical activity recall questionnaire (24PARQ) | 49.1 ± 6.8 yrs. | 81 | F = 81 | 24PARQ determined kcal.kg^-1^.d^-1^ – Doubly labelled water determined energy expenditure (kcal.kg^-1^.d^-1^).  14 days of DLW and ActiGraph measurement compared to 1 day of PA recall from 24PARQ (Unstructured). | Mean_DIFF_= -1.11 ± 5.40 kcal.kg^-1^.d^-1^; p=0.07;  r=0.24; p<0.05 | Nielson (2009) |
| Barnard et al. (2002)^[73]^ | Modifiable Activity Questionnaire (MAQ) | 22-59 yrs. | 15 | M = 7  F = 8 | MAQ determined energy expenditure – Doubly Labelled Water determined energy expenditure  Previous years physical activity behaviours monitored using the MAQ and compared to DLW assessed for 14 days (Unstructured). | No correlation between the two measures. Significantly different results identified (p=0.001) | Nielson (2009) |
| Besson et al. (2010)^[74]^ | Recent Physical Activity Questionnaire (RPAQ) | M = 34.3 ± 8.8 yrs.  F = 35.2 ± 9.9 yrs. | 50 | M = 25  F = 25 | RPAQ determined TEE – Doubly Labelled Water determined TEE.  (kJ.d)  RPAQ determined PAEE – Doubly Labelled Water determined PAEE.  RPAQ activities in last month with 14 days of DLW monitoring (Unstructured). | r_s_=0.67; p<0.01  Mean_DIFF_=-3451.9 ±2025.1 kJ/day  r_s_=0.39; p<0.01  Mean_DIFF_=-12.9 ± 23.9 kJ/day | Helmorhorst (2012) |
| Bonnefoy et al. (2001)^[75]^ | Minnesota Leisure Time Physical Activity Questionnaire | 66-82 yrs. | 19 | M = 19 | MLTPAQ determined PA levels compared with doubly labelled water total energy expenditure. (Kcal/day)  MLTPAQ determined PA levels compared with doubly labelled water total energy expenditure/RMR ratio.  Past 12 months activity behaviours (Unstructured) compared to DLW in older men. | r_p_=0.23  r_s_=0.17  r_p_=0.40  r_s_=0.44 | Westerterp (2009)  Ainslie (2003) |
| Bonnefoy et al. (2001)^[75]^ | Yale Physical Activity Survey (YPAS) | 66-82 yrs. | 19 | M = 19 | YPAS determined energy expenditure (typical week past month) compared with doubly labelled water total energy expenditure.  YPAS determined energy expenditure (typical week past month) compared with doubly labelled water total energy expenditure/RMR ratio.  Typical week past month energy expenditure (unstructured) compared to DLW in older men  YPAS determined PA (past month) compared with doubly labelled water total energy expenditure.  YPAS determined PA (past month) compared with doubly labelled water total energy expenditure/RMR ratio.  Past month summary index of PA behaviours (Unstructured) compared to DLW in older men. | r_p_=0.18  r_s_=0.10  r_p_=0.16  r_s_=0.01  r_p_=0.11  r_s_=0.10  r_p_=0.05  r_s_=0.03 | Westerterp (2009)  Ainslie (2003) |
| Bonnefoy et al. (2001)^[75]^ | Modified Baecke Questionnaire | 66-82 yrs. | 19 | M = 19 | Questionnaire determined Score compared with doubly labelled water total energy expenditure. (points)  Questionnaire determined Score compared with doubly labelled water total energy expenditure/RMR ratio.  Habitual activity behaviours of last year (Unstructured) compared to DLW in older men. | r_p_=0.21  r_s_=0.28  r_p_=0.19  r_s_=0.14 | Westerterp (2009)  Ainslie (2003) |
| Bonnefoy et al. (2001)^[75]^ | College Alumni Questionnaire | 66-82 yrs. | 19 | M = 19 | Questionnaire determined physical activity compared with doubly labelled water total energy expenditure. (kcal/week)  Questionnaire determined physical activity compared with doubly labelled water total energy expenditure/RMR ratio.  Current and past year activity behaviours (unstructured) compared to DLW in older men. | r_p_=0.39  r_s_=0.37  r_p_=0.46; p<0.05  r_s_=0.42 | Westerterp (2009)  Ainslie (2003) |
| Bonnefoy et al. (2001)^[75]^ | Modified Dallosso Questionnaire | 66-82 yrs. | 19 | M = 19 | Questionnaire determined physical activity score compared with doubly labelled water total energy expenditure.  Questionnaire determined physical activity score compared with doubly labelled water total energy expenditure/RMR ratio.  Typical day last week and typical week activity behaviours (Unstructured) compared to DLW in older men. | r_p_=0.21  r_s_=0.34  r_p_=0.26  r_s_=0.32 | Westerterp (2009)  Ainslie (2003) |
| Bonnefoy et al. (2001)^[75]^ | Lipid Research Clinics Questionnaire | 66-82 yrs. | 19 | M = 19 | Questionnaire determined current physical activities compared with doubly labelled water total energy expenditure.  Questionnaire determined current physical activities compared with doubly labelled water total energy expenditure/RMR ratio.  Current physical activity score (Unstructured) compared to DLW in older men. | r_p_=0.33  r_s_=0.29  r_p_=0.28  r_s_=0.37 | Westerterp (2009)  Ainslie (2003) |
| Bonnefoy et al. (2001)^[75]^ | Stanford Usual Activity Questionnaire | 66-82 yrs. | 19 | M = 19 | Questionnaire determined MPA compared with doubly labelled water total energy expenditure.  Questionnaire determined MPA compared with doubly labelled water total energy expenditure/RMR ratio.  Usual MPA (unstructured) compared to DLW in older men.  Questionnaire determined VPA compared with doubly labelled water total energy expenditure.  Questionnaire determined VPA compared with doubly labelled water total energy expenditure/RMR ratio.  Past 3 months VPA (unstructured) compared to DLW in older men. | r_p_=0.65; p<0.05  r_s_=0.46  r_p_=0.52; p<0.05  r_s_=0.38  r_p_=0.63; p<0.05  r_s_=0.64; p<0.05  r_p_=0.75; p<0.05  r_s_=0.66; p<0.05 | Westerterp (2009)  Ainslie (2003) |
| Bonnefoy et al. (2001)^[75]^ | Physical Activity Scale for the Elderly | 66-82 yrs. | 19 | M = 19 | Questionnaire determined physical activity score compared with doubly labelled water total energy expenditure.  Questionnaire determined physical activity score compared with doubly labelled water total energy expenditure/RMR ratio.  Past week activity behaviours (unstructured) compared to DLW in older men. | r_p_=0.28  r_s_=0.23  r_p_=0.36  r_s_=0.24 | Westerterp (2009)  Ainslie (2003) |
| Bonnefoy et al. (2001)^[75]^ | Seven Day recall Questionnaire | 66-82 yrs. | 19 | M = 19 | Questionnaire determined total energy expenditure compared with doubly labelled water total energy expenditure.  Questionnaire determined total energy expenditure compared with doubly labelled water total energy expenditure/RMR ratio.  Past week activity behaviours (unstructured) compared to DLW in older men. | r_p_=0.37; p<0.05  r_s_=0.51; p<0.05  r_p_=0.27  r_s_=0.37 | Westerterp (2009)  Ainslie (2003) |
| Bonnefoy et al. (2001)^[75]^ | Questionnaire d’Activité Physique  Saint-Etienne (QAPSE) | 66-82 yrs. | 19 | M = 19 | Questionnaire determined daily energy expenditure compared with doubly labelled water total energy expenditure.  Questionnaire determined daily energy expenditure compared with doubly labelled water total energy expenditure/RMR ratio.  Typical week past year activity behaviours (unstructured) compared to DLW in older men. | r_p_=0.32  r_s_=0.25  r_p_=0.30  r_s_=0.16 | Westerterp (2009)  Ainslie (2003) |
| Colbert et al. (2011)^[76]^ | The Community Healthy Activities Model Program for Seniors (CHAMPS) questionnaire | 74.7 ± 6.5 yrs. | 56 | M = 12  F = 44 | CHAMPS determined PAEE (kcal.d^-1^) - Doubly labelled water determined PAEE (kcal.d^-1^).  CHAMPS determined PA Index (kcal.kg.^-1^d^-1^) - Doubly labelled water determined PAEE (Adjusted kcal.kg^-1^.d^-1^).  CHAMPS determined PA Index (kcal.kg.^-1^d^-1^) - Doubly labelled water determined PA Index. | RMSE = 242; MAPE = 30.4; r_s_ = 0.28; p<0.05  RMSE = 226; MAPE = 3.1; r_s_ = 0.23; NS  RMSE = 3.42; MAPE = 29.0; r_s_ = 0.23; NS | Plasqui (2013) |
| Colbert et al. (2011)^[76]^ | Yale Physical Activity Survey (YPAS) | 74.7 ± 6.5 yrs. | 56 | M = 12  F = 44 | YPAS determined PAEE (kcal.d^-1^) - Doubly labelled water determined PAEE (kcal.d^-1^).  YPAS determined PA Index (kcal.kg.^-1^d^-1^) - Doubly labelled water determined PAEE (Adjusted kcal.kg^-1^.d^-1^).  YPAS determined PA Index (kcal.kg.^-1^d^-1^) - Doubly labelled water determined PA Index.  Participants wore the devices during the 15 days of DLW measurement and completed the questionnaire on day 8 and day 15 of the study (Unstructured) | RMSE = 252; MAPE = 32.8; r_s_ = 0.07; NS  RMSE = 231; MAPE = -15.6; r_s_ = 0.09; NS  RMSE = 3.45; MAPE = 29.4; r_s_ = 0.18; NS | Plasqui (2013) |
| Colbert et al. (2011)^[76]^ | Physical Activity Scale for the Elderly (PASE) | 74.7 ± 6.5 yrs. | 56 | M = 12  F = 44 | PASE determined PAEE (kcal.d^-1^) - Doubly labelled water determined PAEE (kcal.d^-1^).  PASE determined PA Index (kcal.kg.^-1^d^-1^) - Doubly labelled water determined PAEE (Adjusted kcal.kg^-1^.d^-1^).  PASE determined PA Index (kcal.kg.^-1^d^-1^) - Doubly labelled water determined PA Index.  Participants wore the devices during the 15 days of DLW measurement and completed the questionnaire on day 8 and day 15 of the study (Unstructured) | RMSE = 256; MAPE = 32.4; r_s_ = 0.20; NS  RMSE = 234; MAPE = -20.6; r_s_ = 0.11; NS  RMSE = 3.51; MAPE = 29.3; r_s_ = 0.18; NS | Plasqui (2013) |
| Conway et al. (2002)^[77]^ | Tecumseh Community Health Study Questionnaire | 27-65 yrs. | 24 | M = 24 | TCHSQ determined energy expenditure – Doubly labelled water energy expenditure (MJ/d). | Mean _DIFF_ = 1%-9% | Ainslie (2003) |
| Conway et al. (2002)^[77]^ | Minnesota Leisure time questionnaire | 27-65 yrs. | 24 | M = 24 | MLTPAS determined energy expenditure – Doubly labelled water energy expenditure (MJ/d). | Mean _DIFF_ = 1%-9% | Ainslie (2003) |
| Conway et al. (2002)^[77]^ | Combined Minnesota Leisure Time Survey and Tecumseh Community Health Study Questionnaire (MJ.d-1) | 27-65 yrs. | 24 | M = 24 | Combined MLTPAS and TCHSQ determined energy expenditure (MJ.d^-1^) – Doubly labelled water determined energy expenditure (MJ/d) | Mean_%DIFF_= 1.7% | Ainslie (2003) |
| Conway et al. (2002)^[77]^ | Combined Minnesota Leisure Time Survey and Tecumseh Community Health Study Questionnaire (+ parenting questionnaire) (MJ.d-1) | 27-65 yrs. | 24 | M = 24 | Combined MLTPAS and TCHSQ determined energy expenditure (with parental questionnaire) (MJ.d^-1^) – Doubly labelled water determined energy expenditure (MJ/d) | Mean_%DIFF_= 9.7% | Ainslie (2003) |
| Conway et al. (2002)^[78]^ | Stanford 7 day physical activity recall | 27-65 yrs. | 27 | M = 27 | 7 days physical activity determined energy expenditure – Doubly labelled water Energy Expenditure (MJ/d^-1^).  Past 7 days physical activities compared to 14 days DLW measurement. | Mean_%DIFF_ = 7.9% | Ainslie (2003) |
| Conway et al. (2002)^[78]^ | Stanford 7 day physical activity recall | 27-65 yrs. | 27 | M = 27 | 7 days physical activity determined energy expenditure – Doubly labelled water Energy Expenditure (MJ/d^-1^).  7 24 hour physical activities compared to 14 days DLW measurement. | Mean _DIFF_ = 30.6% | Ainslie (2003) |
| Fuller et al. (2008)^[79]^ | Physical Activity Diary (PAD) | 20-66 yrs. | 60 | M = 30  F = 30 | PAD determined energy expenditure (MJ.d^-1^) – Doubly Labelled Water determined energy expenditure (MJ.d^-1^)  15 days of DLW measurement, HRM, Physical Activity Diary, Caltrac device and 2 versions of the 7DPARQ (Day 8 and day 15 (Unstructured). | r=0.58 | Plasqui (2013) |
| Fuller et al. (2008)^[79]^ | Physical Activity Diary (PAD) | 20-66 yrs. | 60 | M = 30  F = 30 | PAD determined energy expenditure (PAL) – Doubly Labelled Water determined energy expenditure (PAL)  15 days of DLW measurement, HRM, Physical Activity Diary, Caltrac device and 2 versions of the 7DPARQ (Day 8 and day 15 (Unstructured). | r=0.09 | Plasqui (2013) |
| Fuller et al. (2008)^[79]^ | 7 day physical activity recall questionnaire (7DPAR) | 20-66 yrs. | 60 | M = 30  F = 30 | 7DPAR determined energy expenditure (MJ.d^-1^) – Doubly Labelled Water determined energy expenditure (MJ.d^-1^)  15 days of DLW measurement, HRM, Physical Activity Diary, Caltrac device and 2 versions of the 7DPARQ (Day 8 and day 15 (Unstructured). | r=0.61 | Plasqui (2013) |
| Fuller et al. (2008)^[79]^ | 7 day physical activity recall questionnaire | 20-66 yrs. | 60 | M = 30  F = 30 | 7DPAR determined energy expenditure (Physical Activity Level (PAL)) – Doubly Labelled Water determined energy expenditure (PAL)  15 days of DLW measurement, HRM, Physical Activity Diary, Caltrac device and 2 versions of the 7DPARQ (Day 8 and day 15 (Unstructured). | r=0.28 | Plasqui (2013) |
| Goran et al. (1992)^[80]^ | Minnesota Leisure Time Physical Activity Survey (MLTPAS) | 26-78 yrs. | 13 | M = 7  F = 6 | MLTPAS Past 12 months (kcal/d) – Doubly Labelled Water energy expenditure (kcal/d).  MLTPAS kcal/d compared to Doubly Labelled Water over a 10 day period (Unstructured). | r=0.74; p<0.05 | Ainslie (2003) |
| Irwin et al. (2001)^[81]^ | Stanford 7 day physical activity recall (S7DPAR) | 41.2 ± 9.6 yrs. | 24 | M = 24 | S7DPAR determined energy expenditure (kcal.d^-1^) – Doubly labelled water determined energy expenditure (kcal.d^-1^)  S7DPAR recorded during the DLW measurement protocol (Unstructured). | Mean_DIFF_=987 ± 324 kcal.d^-1^; p<0.05 | Prince (2008) |
| Irwin et al. (2001)^[81]^ | 7 day Physical Activity Record | 41.2 ± 9.6 yrs. | 24 | M = 24 | 7 day Physical Activity Record determined energy expenditure (kcal.d^-1^) – Doubly labelled water determined energy expenditure (kcal.d^-1^)  7 days of physical activity records recorded during the DLW measurement protocol (Unstructured). | Mean_DIFF_=224 ± 100 kcal.d^-1^; NS | Prince (2008) |
| Koebnick et al. (2005)^[91]^ | Physical Activity record ([www.dife.de](http://www.dife.de)) | 36.8 ± 11.4 yrs. | 31 | M = 15  F = 16 | 7 day Physical Activity record determined energy expenditure- Doubly Labelled Water determined energy expenditure (MJ/d)  7 days recorded physical activity (converted to EE) (Unstructured) compared to TEE DLW determined from 13 days analysis. | r=0.88; p<0.001 | Westerterp (2009) |
| Leenders et al. (2001)^[92]^ | 7-d Physical Activity Recall questionnaire | 25.8 ± 1.6 yrs. | 13 | F = 13 | 7DPARQ determined kcal/d - Doubly Labelled Water energy expenditure (kcal/d).  7 days of PAR measurement compared to 7 days DLW measurement (Unstructured). | Mean _DIFF_=5% | Ainslie (2003) |
| Livingston et al. (1991)^[93]^ | Occupational and Discretional Physical Activity Questionnaire | 17-54 yrs. | 32 | M = 16  F = 16 | Occupational and Discretionary PAQ determined activity score (hrs.wk^-1^ spent in MVPA) – Doubly labelled water determined physical activity energy expenditure (MJ.d^-1^).  Usual physical activity patterns assessed using interview administered questionnaire compared to 15 days DLW measurement (Unstructured). | r=0.79; p<0.01 | Nielson (2009) |
| Lof et al. (2002)^[95]^ | Physical Activity Questionnaire | 30 ± 4 yrs. | 24 | F = 24 | PAQ determined energy expenditure (kcal.d^-1^) - Doubly labelled water determined energy expenditure (kcal.d^-1^).  PAQ administered after the 14 days of DLW assessment (Unstructured). | Mean_DIFF_=204 kcal; 2 SDs = 1015 kcal.24hrs^-1^. R=0.56; p<0.05 | Plasqui (2007) |
| Lof et al. (2004)^[94]^ | Physical activity during work and leisure (Black) | 29 ± 4 yrs. | 37 | F = 37 | Total energy expenditure as determined by Questionnaire – Doubly labelled water determined total energy expenditure (KJ.d^-1^).  Usual habitual physical activity during work and leisure compared to 14 days of DLW measurement (Unstructured). | Significant underestimation; p<0.05 | Nielson (2009) |
| Lof et al. (2004)^[94]^ | Physical activity during work and leisure (Lof 2003) | 29 ± 4 yrs. | 37 | F = 37 | Total energy expenditure as determined by Questionnaire – Doubly labelled water determined total energy expenditure (KJ.d^-1^).  Total energy expenditure as determined by Questionnaire (KJ.d^-1^) – Doubly labelled water determined total energy expenditure.  Usual habitual physical activity during work and leisure compared to 14 days of DLW measurement (Unstructured). | Significant underestimation; p<0.05  Mean_DIFF_=544; NS; LoA = 3862 KJ.d^-1^. | Nielson (2009) |
| Maddison et al. (2007)^[82]^ | International Physical Activity Questionnaire (IPAQ) | 18-64 yrs. | 36 | M = 16  F = 20 | IPAQ energy expenditure – Doubly Labelled Water Energy Expenditure  Free living physical activity for 14 days (Unstructured). | r_s_ =0.31; p=0.06 | Westerterp (2009) |
| Maddison et al. (2007)^[82]^ | New Zealand Physical Activity Questionnaire (NZPAQ) | 18-64 yrs. | 36 | M = 16  F = 20 | NZPAQ energy expenditure – Doubly Labelled Water Energy Expenditure  Free living physical activity for 14 days (Unstructured). | r_s_=0.38; p<0.05 | Westerterp (2009) |
| Mahabir et al. (2006)^[96]^ | Harvard Alumni Questionnaire | 49–79 yrs. | 65 | F = 65 | Harvard Alumni Questionnaire Energy Expenditure – Doubly Labelled Water energy expenditure  Weighted METs/h computed an converted to kcal/d for questionnaire and compared against DLW kcal/d (Unstructured). | r_s_=0.36; p<0.05 | Westerterp (2009) |
| Mahabir et al. (2006)^[96]^ | Five City Project Questionnaire | 49–79 yrs. | 65 | F = 65 | Five City Project Questionnaire Energy Expenditure – Doubly Labelled Water energy expenditure  Weighted METs/h computed an converted to kcal/d for questionnaire and compared against DLW kcal/d (Unstructured). | r_s_=0.47; p<0.05 | Westerterp (2009) |
| Mahabir et al. (2006)^[96]^ | Cross-Cultural Activity Participation Study Four Week  Activity Recall | 49–79 yrs. | 65 | F = 65 | CCAPS 4 week activity recall Energy Expenditure – Doubly Labelled Water energy expenditure  Weighted METs/h computed an converted to kcal/d for questionnaire and compared against DLW kcal/d (Unstructured). | r_s_=0.16 | Westerterp (2009) |
| Mahabir et al. (2006)^[96]^ | Typical Week Activity Recall  questionnaires | 49–79 yrs. | 65 | F = 65 | Typical week activity recall questionnaire Energy Expenditure – Doubly Labelled Water energy expenditure  Weighted METs/h computed and converted to kcal/d for questionnaire and compared against DLW kcal/d (Unstructured). | r_s_=0.15 | Westerterp (2009) |
| Masse et al. (2004)^[83]^ | Physical Activity Diary | 40-70 yrs. | 136 | F = 136 | Physical activity diary determined kcal/d – Doubly Labelled water determined PAEE kcal/d.  Physical activity diary determined kcal/d – Doubly Labelled water determined TEE kcal/d.  PAD collected on second week of DLW measurement during free-living activity behaviours (Unstructured). | Mean_DIFF_=150 kcal/d.  r_p_=0.09-0.30  r_p_=0.04-0.50 | Corder (2007) |
| Morio et al (1997)^[84]^ | Self-reported physical activity | 70.1 ± 2.7 yrs. | 12 | M = 6  F = 6 | Self-reported physical activity determined energy expenditure (calculated using factorial method based on prolonged stay in Calorimetry chamber) (MJ.d^-1^) – Doubly Labelled Water determined energy expenditure (MJ.d^-1^).  Self-reported PA energy expenditure calculated using factorial method for 14 days based on prolonged stay in Calorimetry chamber compared to DLW measured for 14 days (Unstructured). | Mean_DIFF_ Men = -0.6 (SD = 4.8)%; (-5.8 – 7.4%)  Mean_DIFF_ Women = 0.5 (SD = 2.8)%; (-3.3 – 4.5%) | Kowalski (2012) |
| Paul et al. (2005)^[85]^ | Modified Bouchard physical activity log | 39 ± 9 yrs. | 12 | M = 12 | Physical activity log determined (MJ.d^-1^) – Doubly labelled water determined energy expenditure (MJ.d^-1^)  7 consecutive days of activity behaviours recorded using PAL compared with 7 days DLW measurement (Unstructured). | r=0.37; NS | Prince (2008) |
| Philippaerts et al. (1999)^[97]^ | Baecke Physical activity questionnaire | NA | 19 | M = 19 | Current and past 12 months physical activity (total activity index) – Doubly Labelled Water determined PAL. | r=0.69; p<0.001 | Ainslie (2003) |
| Philippaerts et al. (1999)^[97]^ | Tecumseh Community Health Study Questionnaire | NA | 19 | M = 19 | TCHSQ determined TDEE - Doubly Labelled Water determined PAL | r=0.64; p<0.05 | Ainslie (2003) |
| Philippaerts et al. (1999)^[97]^ | Five City Project Questionnaire | NA | 19 | M = 19 | FCPQ determined sweat index - Doubly Labelled Water determined PAL | r=0.57; p<0.001 | Ainslie (2003) |
| Racette et al. (1995)^[98]^ | 7 day Physical Activity Recall Questionnaire | 21-47 yrs. (obese) | 14 | F = 14 | 7DPARQ determined TDEE – Doubly labelled water determined TDEE. (MJ.d)  7 days free living physical activity behaviours through self-report compared to 14 days DLW TDEE (Unstructured). | Mean_DIFF_=+0.9% | Ainslie (2003) |
| Rafamantanantsoa et al. (2002)^[99]^ | Physical Activity Records | 48 ± 10 yrs. | 24 | M = 24 | Physical Activity determined energy expenditure (measured for 3 days) – Doubly labelled water determined energy expenditure (measured for 14 days).  Physical Activity Record recorded for 3 day period within the 14 day examination while DLW was measure over the same period (Unstructured). | r_p_=0.76; p<0.0001; Intra-individual variation = 7±4% | Plasqui (2007) |
| Reilly et al. (1993)^[100]^ | Physical activity questionnaire (modified from Dallosso et al. (1988)) | 73 ± 3 yrs. | 10 | F = 10 | Physical activity questionnaire determined score – Doubly labelled water determined physical activity level.  Physical activity for a typical day during DLW measurement period via self-report compared to DLW measured for 14-18 days (Unstructured). | r=0.77; p<0.01 | Neilson (2009) |
| Rothenberg et al. (1998)^[86]^ | Activity Diary | 73 yrs. | 20 | M = 8  F = 12 | Activity diary determined energy expenditure (MJ.d^-1^) – Doubly labelled water determined energy expenditure (MJ.d^-1^)  DLW measured for 20 days compared with 4 days of activity diary records (Unstructured). | Mean_DIFF_=0.65 ± 1.90 MJ.d^-1^; NS; Mean%_DIFF_ =6% ± 19%; r_s_=0.49; NS | Prince (2008) |
| Rush et al. (2008)^[87]^ | 7 day Physical Activity Diary | 18-27 yrs. | 29 | M = 29 | 7 day Physical Activity Diary Energy expenditure- Doubly Labelled Water Energy Expenditure. No units.  7 days of free-living activity behaviours (Unstructured). | r_p_=0.61; p<0.001 | Westerterp (2009) |
| Schuit et al. (1997)^[102]^ | Physical activity scale for the elderly | 60-80 yrs. | 21 | M = 10  F = 11 | Usual physical activity determined score - Doubly labelled water determined PAL and TEE.  Usual physical activities compared to 14 days DLW measurement. | r_s PAR_=0.68  r_s TEE_=0.58 | Ainslie (2003) |
| Schulz et al. (1989)^[101]^ | 2 week activity diary | NA | 6 | M = 4  F = 2 | 2 week activity diary determined energy expenditure – Doubly labelled water energy expenditure (MJ/d)  2 weeks of free-living physical activity behaviours using (Unstructured). | r=0.57-0.72; p<0.05 | Ainslie (2003) |
| Scultz et al. (1994)^[88]^ | Physical Activity Questionnaire | M: 35.4 ± 13.8 yrs.  F: 31.3 ± 13.0 yrs. | 21 | M = 12  F =9 | PAQ determined leisure and occupational physical activity - Doubly labelled water determined total energy expenditure (MET-hrs.wk^-1^).  PAQ determined leisure and occupational physical activity - Doubly labelled water determined total energy expenditure (MET-hrs.wk^-1^).  PAQ determined hours.wk^-1^ physical activity - Doubly labelled water determined total energy expenditure (MET-hrs.wk^-1^).  PAQ examined average physical activity behaviours over the last year compared to 11 days of DLW measurement (Unstructured). | r=0.74; p<0.05  r=0.56; p<0.05  r=0.69; p<0.05 | Prince (2008) |
| Seale et al. (2002)^[103]^ | 7-d Physical Activity Recall questionnaire | 74.1 ± 4.1 yrs. | 27 | M = 14  F = 13 | 7DPARQ determined MJ/d - Doubly Labelled Water energy expenditure (MJ/d).  7 days of PAR measurement compared to 14 days DLW measurement (Unstructured). | Mean _DIFF_=9.8-37.4% | Ainslie (2003) |
| Starling et al. (1998)^[89]^ | Minnesota Leisure Time Questionnaire | 52-79 yrs. | 65 | M = 28  F = 37 | MLTPAS determined leisure time PA energy expenditure (kcal.d^-1^) – Doubly labelled water determined PA energy expenditure (kcal.d^-1^) (MEN ONLY)  MLTPAS determined leisure time PA energy expenditure (kcal.d^-1^) – Doubly labelled water determined PA energy expenditure (kcal.d^-1^) (Adjusted) (MEN ONLY)  MLTPAS determined leisure time PA energy expenditure (kcal.d^-1^) – Doubly labelled water determined PA energy expenditure (kcal.d^-1^) (WOMEN ONLY)  MLTPAS determined leisure time PA energy expenditure (kcal.d^-1^) – Doubly labelled water determined PA energy expenditure (kcal.d^-1^) (Adjusted) (WOMEN ONLY)  MLTPAS examined previous 12 months PA behaviour with DLW examined for 10 days (Unstructured). | r_p_=-0.17  r_p_=-0.25  r_p_=-0.01  r_p_=-0.05 | Prince (2008) |
| Starling et al. (1999)^[104]^ | Minnesota Leisure Time Physical Activity Questionnaire | 45-84 yrs. | 77 | M = 32  F = 35 | MLTPAS Past 12 months (kcal/d) – Doubly Labelled Water energy expenditure (kcal/d).  MLTPAS kcal/d compared to Doubly Labelled Water over a 10 day period (Unstructured). | Mean _DIFF_ = 56%-62% | Ainslie (2003) |
| Staten et al. (2001)^[90]^ | Arizona Activity Frequency Questionnaire | 40–82 yrs. | 35 | F = 35 | Arizona Activity Frequency Questionnaire Energy expenditure – Doubly Labelled Water  28 days of free-living activity behaviours measurement compared to 9 days DLW measurement (Unstructured). | r^2^=0.45; p<0.001 (Mifflin equation).  r^2^=0.58; p<0.001 (MET conversion technique). | Westerterp (2009) |
| Walsh et al. (2004)^[105]^ | Tecumseh Occupational Activity Questionnaire | 20-46 yrs. | 75 | F = 75 | TOAQ determined energy expenditure (kcal.d^-1^) – Doubly Labelled water determined energy expenditure (kcal.d^-1^).  TOAQ determined work history, transportation to work, PA at work etc. compared to 14 days DLW measurement (Unstructured). | Significant overestimation; p<0.001 | Neilson (2009) |
| Walsh et al. (2004)^[105]^ | Baecke Activity Questionnaire | 20-46 yrs. | 75 | F = 75 | BPAQ determined energy expenditure (kcal.d^-1^) – Doubly Labelled water determined energy expenditure (kcal.d^-1^).  BPAQ determined physical activity index (previous year) compared to 14 days DLW measurement (Unstructured). | Significant overestimation; p<0.001 | Neilson (2009) |
| Walsh et al. (2004)^[105]^ | Minnesota Leisure Time PA Questionnaire | 20-46 yrs. | 75 | F = 75 | MLTPAQ determined energy expenditure (kcal.d^-1^) – Doubly Labelled water determined energy expenditure (kcal.d^-1^).  Previous 12 months usual physical activity compared to 14 days DLW measurement (Unstructured). | Significant overestimation; p<0.001 | Neilson (2009) |
| Washburn et al. (2003)^[106]^ | Standford 7 day physical activity recall questionnaire | 17-35 yrs. | 46 | M = 17  F= 29 | Questionnaire determined total daily energy expenditure compared with doubly labelled water total energy expenditure. (kJ.d-1)  Last week past year activity behaviours compared to DLW in overweight men and women (Unstructured). | r=0.58; p<0.01 | Westerterp (2009) |

PA = Physical Activity; MPA = Moderate Physical Activity; VPA = Vigorous Physical Activity; MVPA = Moderate-to-Vigorous Physical Activity; EE = Energy Expenditure; PAEE = Physical Activity Energy Expenditure; TEE = Total Energy Expenditure; RMR = Resting Metabolic Rate; MJ = Megajoules; PAL = Physical Activity Level; MET = Metabolic Equivalent; DLW = Doubly Labelled Water; HRM = Heart Rate Monitor; NS = Non-significant; RMSE = Root mean squared error; MAPE = Mean absolute percentage difference; M = Male; F = Female; S5CPPAR = Stanford 5 City Project Physical Activity Recall; S7DPAR = Stanford 7 day physical activity recall; MLTPAS = Minnesota Leisure Time Physical Activity Survey; 24PARQ = 24 hour physical activity recall questionnaire; MAQ = Modifiable Activity Questionnaire; RPAQ = Recent Physical Activity Questionnaire; YPAS = Yale Physical Activity Survey; TCHSQ = Tecumseh Community Health Study Questionnaire; PAD = Physical Activity Diary; CHAMPS = Community Healthy Activities Model Program for Seniors; PASE = Physical Activity Scale for the Elderly; 7DPAR = 7 day physical activity recall questionnaire; PAQ = Physical Activity Questionnaire; IPAQ = International Physical Activity Questionnaire; NZPAQ = New Zealand Physical Activity Questionnaire; CCAPS = Cross-Cultural Activity Participation Study; TOAQ = Tecumseh Occupational Activity Questionnaire; BPAQ = Baecke Physical Activity Questionnaire;

**Supplementary Table 2.** Concurrent validity of self-reported measures of physical activity/energy expenditure.

| **Author** | **Measure** | **Age Range** | **Sample Size** | **Sex** | **Reference** | **Results** | **Primary Source** |
| --- | --- | --- | --- | --- | --- | --- | --- |
| Aadahl et al. (2003)^[107]^ | 24 hour recall self-administered activity Scale | 20-60 yrs. | 39 | M = 19  F = 20 | 24PAQ determined energy expenditure (MET-time) – CSA determined energy expenditure (MET-time)  Participants wore the CSA for 4 days, while 4 days of physical activity diary information and 1 day of the completed physical activity scale (Unstructured). | r=0.74; p<0.001  Mean_DIFF_=26% | Prince (2008) |
| Ainsworth et al. (2000)^[108]^ | Physical Activity Questionnaire | M = 47.2 ± 15.3 yrs.  F = 45.4 ± 15 yrs. | 83 | M = 38  F = 45 | Minutes in MPA – ActiGraph determined minutes in MPA (Freedson)  Minutes in VPA – ActiGraph determined minutes in MPA (Freedson)  21 days free living physical activity behaviours. | r=0.26; p<0.01  r=0.32; p<0.01 | Berlin (2006) |
| Ainsworth et al. (2000)^[108]^ | Physical Activity Questionnaire | M = 47.2 ± 15.3 yrs.  F = 45.4 ± 15 yrs. | 83 | M = 38  F = 45 | Minutes in MPA – ActiGraph determined minutes in MPA (Hendelman)  Minutes in VPA – ActiGraph determined minutes in MPA (Hendelman)  21 days free living physical activity behaviours. | r=0.11  r=0.31; p<0.01 | Berlin (2006) |
| Ainsworth et al. (2000)^[108]^ | Physical Activity Questionnaire | M = 47.2 ± 15.3 yrs.  F = 45.4 ± 15 yrs. | 83 | M = 38  F = 45 | Minutes in MPA – ActiGraph determined minutes in MPA (Swartz)  Minutes in VPA – ActiGraph determined minutes in MPA (Swartz)  21 days free living physical activity behaviours. | r=0.19  r=0.33; p<0.01 | Berlin (2006) |
| Ainsworth et al. (2000)^[108]^ | Physical Activity Questionnaire | M = 47.2 ± 15.3 yrs.  F = 45.4 ± 15 yrs. | 83 | M = 38  F = 45 | Daily Minutes in MVPA – Daily PA Log (3x 1 week logs)  21 days free living physical activity behaviours. | r=0.40-0.54; p<0.01 | Berlin (2006) |
| Ainsworth et al. (2000)^[108]^ | Physical Activity Log | M = 47.2 ± 15.3 yrs.  F = 45.4 ± 15 yrs. | 83 | M = 38  F = 45 | Minutes in MPA – ActiGraph determined minutes in MPA (Freedson)  Minutes in VPA – ActiGraph determined minutes in MPA (Freedson)  21 days free living physical activity behaviours. | r=0.24; p<0.05  r=0.36; p<0.01 | Berlin (2006) |
| Ainsworth et al. (2000)^[108]^ | Physical Activity Log | M = 47.2 ± 15.3 yrs.  F = 45.4 ± 15 yrs. | 83 | M = 38  F = 45 | Minutes in MPA – ActiGraph determined minutes in MPA (Hendelman)  Minutes in VPA – ActiGraph determined minutes in MPA (Hendelman)  21 days free living physical activity behaviours. | r=0.35; p<0.01  r=0.36; p<0.01 | Berlin (2006) |
| Ainsworth et al. (2000)^[108]^ | Physical Activity Log | M = 47.2 ± 15.3 yrs.  F = 45.4 ± 15 yrs. | 83 | M = 38  F = 45 | Minutes in MPA – ActiGraph determined minutes in MPA (Swartz)  Minutes in VPA – ActiGraph determined minutes in MPA (Swartz)  21 days free living physical activity behaviours. | r=0.32; p<0.01  r=0.31; p<0.01 | Berlin (2006) |
| Ainsworth et al. (1993)^[109]^ | College Alumnus Questionnaire | 38±9 yrs. | 78 | M = 28  W = 50 | CAQ determined MET/min/wk – Caltrac determined kcals and METs  Self-Administered CAQ past 7 days activity compared to Caltrac measures for 2 days of monitoring. | r_kcal_=-0.00 – 0.17  r_METS_=0.07 – 0.29 | Andrews (2010) |
| Ainsworth et al. (1993)^[109]^ | College Alumnus Questionnaire | 38±9 yrs. | 78 | M = 28  W = 50 | CAQ determined MET/min/wk - Activity record diary determined METs/min  Self-Administered CAQ past 7 days activity. | r=-0.03 – 0.69 | Andrews (2010) |
| Ainsworth et al. (1999)^[110]^ | Tecumseh Occupational Activity Questionnaire (TOAQ) | 20-60 yrs. | 40 | F = 40 | TOAQ determined MET min/wk. – Physical activity record determined MET min/wk.  TOAQ determined MET min/wk. – Caltrac determined MET min/d.  Previous Year determined occupational physical activity compared to previous weeks Caltrac determined physical activity (Unstructured). | r=0.26-0.73; p<0.05  r=0.34 | Cheung (2011) |
| Ainsworth et al. (1999)^[110]^ | 7 day recall physical activity questionnaire (7DPARQ) | 20-60 yrs. | 40 | F = 40 | 7DRPAQ determined MET min/wk. – Physical activity record determined MET min/wk.  Past 7 days determined occupational physical activity compared to previous weeks Caltrac determined physical activity (Unstructured). | r=0.32-0.35; p<0.05  NR | Cheung (2011) |
| Arroll et al. (1991)^[111]^ | 3 Month physical activity questionnaire | M: 53.2 ± 9.6 yrs.  F: 55.7 ± 7.9 yrs. | 152 | M=77  F=75 | 3MPAQ determined moderate intensity physical activity – 7 day self-report physical activity diary determined MPA.  3MPAQ determined vigorous intensity physical activity – 7 day self-report physical activity diary determined VPA.  3MPAQ determined total physical activity – 7 day self-report physical activity diary determined total PA.  3MPAQ determined activity behaviours compared with 7 day diary (Unstructured). | r_s_=0.61; kappa= 0.36  r_s_=0.49; kappa= 0.23  r_s_=0.86; kappa= 0.62 | Van Poppel (2010) |
| Baranowski et al. (1984)^[112]^ | Weekly self-report of aerobic activity. | Adults = 23  Youth = 25 (data treated together) | 48 | NA | WSRAA determined minutes spent in aerobic activity – Daily self-monitoring form determined minutes spent in aerobic activity  Past 4 weeks recall of activity compared with daily self-monitoring technique (Unstructured). Insufficient information available to report on remaining measures (Study 2 here). | r_p_=0.00-0.17; NS | Kohl (2000) |
| Bassett et al. (2000)^[113]^ | College Alumnus Questionnaire | 25-70 yrs. | 96 | M = 48  F = 48 | College Alumnus Questionnaire (PAI-CAQ) determined daily walking distance – Yamax DW-500 Pedometer determined daily walking distance.  7 days of pedometer determined data against 7 days self-reported “walking, stair climbing and sports and recreation physical activity” (reported daily) (Unstructured). | r_men_=0.346; p<0.05  r_women_=0.481; p<0.01 | Bassett (2000) |
| Besson et al. (2010)^[74]^ | Recent Physical Activity Questionnaire | M = 34.3 ± 8.8 yrs.  F = 35.2 ± 9.9 yrs. | 50 | M = 25  F = 25 | RPAQ determined VPA (hrs) – ActiWatch determined VPA (hrs).  RPAQ determined MPA (hrs) – ActiWatch determined MPA (hrs).  RPAQ determined LIPA (hrs) – ActiWatch determined LIPA (hrs).  RPAQ activities in last month with 11 days of ActiHeart monitoring (Unstructured). | r_s_=0.70; p<0.01  Mean_DIFF_=0.2 ±0.4 hrs/day  Mean_DIFF_= -0.8 ±1.0 hrs/day  Mean_DIFF_= -0.1 ±2.4 hrs/day | Helmorhorst (2012) |
| Boon et al. (2010)^[114]^ | New Zealand Physical Activity Questionnaire | 18-65 yrs. | 64 | M = 28  F = 36 | NZPAQ determined MPA (mins) – ActiGraph determined MPA (Freedson equation).  NZPAQ determined VPA (mins) – ActiGraph determined VPA (Freedson equation).  NZPAQ determined MVPA (mins) – ActiGraph determined MVPA (Freedson equation).  Free-living activity behaviours examined using NZPAQ and validated with 7 days of ActiGraph measurement (Unstructured). | r_s_=0.30; 95% CI = -0.06-0.51  r_s_=0.37; 95% CI = 0.14-0.56  r_s_=0.32; 95% CI = -0.08-0.52 | Kim (2013) |
| Boon et al. (2010)^[114]^ | International Physical Activity Questionnaire | 18-65 yrs. | 64 | M = 28  F = 36 | IPAQ determined MPA (mins) – ActiGraph determined MPA (Freedson equation).  IPAQ determined VPA (mins) – ActiGraph determined VPA (Freedson equation).  IPAQ determined MVPA (mins) – ActiGraph determined MVPA (Freedson equation).  Free-living activity behaviours examined using IPAQ and validated with 7 days of ActiGraph measurement (Unstructured). | r_s_=0.19; 95% CI = -0.06-0.42  r_s_=0.42; 95% CI = 0.19-0.60  r_s_=0.30; 95% CI = -0.06-0.51 | Kim (2013) |
| Boon et al. (2010)^[114]^ | New Zealand Physical Activity Questionnaire | 18-65 yrs. | 64 | M = 28  F = 36 | NZPAQ determined MPA (mins) – IPAQ determined MPA (mins).  NZPAQ determined VPA (mins) – IPAQ determined VPA (mins).  NZPAQ determined MVPA (mins) – IPAQ determined MVPA (mins).  Free-living activity behaviours examined using NZPAQ and validated with 7 days of ActiGraph measurement (Unstructured). | r_s_=0.68  r_s_=0.67  r_s_=0.79 | Kim (2013) |
| Brown et al. (2008)^[115]^ | Active Australia Physical Activity Survey | 54-59 yrs. | 159 | F = 159 | AAPAS determined frequency of MVPA per week – ActiGraph determined MVPA frequency per week (Freedson equation).  AAPAS determined total mins MVPA per week – ActiGraph determined total mins MVPA per week (Freedson equation).  Free-living activity behaviours examined for 7 days using both AAPAS and ActiGraph (Unstructured). | r_s_=0.48; p<0.001  r_s_=0.52; p<0.001 | Helmorhorst (2012) |
| Bull et al. (2009)^[116]^ | Global Physical Activity Questionnaire | NA | 298 | NA | GPAQ determined total VPA minutes– ActiGraph MTI determined mean VPA counts/d  GPAQ determined total MPA minutes– ActiGraph MTI determined mean MPA counts/d.  7 days of free-living activity behaviours (Unstructured). | r_s_=0.23-0.26; p<0.05  r_s_=-0.03-0.23; | Helmorhorst (2012) |
| Bull et al. (2009)^[116]^ | Global Physical Activity Questionnaire | NA | 298 | NA | GPAQ determined total VPA– IPAQ determined total VPA  GPAQ determined total MPA– IPAQ determined total MPA  GPAQ determined total PA– IPAQ determined total PA  7 days of free-living activity behaviours (Unstructured). | r_s_=0.42-0.79; p<0.01  r_s_=0.29-0.75; p<0.01  r_s_=0.23-0.92; p<0.01 | Helmorhorst (2012) |
| Bulley et al. (2005)^[117]^ | Scottish Physical Activity Questionnaire | 18-25 yrs. | 25 | F = 25 | SPAQ determined minutes spent in MVPA – Heart rate monitor determined minutes spent in MVPA (from calibration study).  3 days of HRM and 7 days of physical activity recall (Unstructured). | Mean_DIFF_=10.1 mins; 95% LoA = 1.32 – 4.76; r= 0.59 | Prince (2008) |
| Busse et al. (2009)^[118]^ | Activity Diary | 19.9-27.2 yrs. | 22 | NA | Diary determined inactivity - SAM determined inactivity  Diary determined low activity - SAM determined low activity  Diary determined moderate activity - SAM determined moderate activity  Diary determined high activity - SAM determined high activity  4 consecutive days of SAM data collection and diary recorded activity levels (Unstructured). | r=0.47; p<0.05; Overestimation = 7.2 hrs.  r=0.42; p<0.05; Underestimation = 6.5 hrs.  r=0.48; p<0.05; No Difference  r=0.59; p<0.01; No Difference | Shephard (2012) |
| Carter-Nolan et al. (2006)^[119]^ | Black Women’s health study Self-administered physical activity questionnaire | 49.5 ± 10.7 yrs. | 101 | F = 101 | BWHSPAQ determined energy expenditure (MET-hours.wk^-1)^ – Activity diary determined energy expenditure (MET-hours.wk^-1)^  BWHSPAQ determined energy expenditure (MET-hours.wk^-1)^ from moderate activity– Activity diary determined energy expenditure (MET-hours.wk^-1)^ from moderate activity  BWHSPAQ determined energy expenditure (MET-hours.wk^-1)^ from vigorous activity– Activity diary determined energy expenditure (MET-hours.wk^-1)^ from vigorous activity  BWHSPAQ examined mean hours per week in specific activities over the past year compared with 7days of physical activity diaries and ActiGraph data (Unstructured). | r_s_= 0.32; p<0.01  r_s_= 0.26; p<0.05  r_s_= 0.41; p<0.01 | Prince (2008) |
| Cartmel et al. (1992)^[120]^ | Minnesota Leisure Time Physical Activity Survey | 66-80 yrs. | 24 | M = 9  F = 15 | MLTPAS determined minutes spent in light physical activity – Activity Diary determined number of minutes spent in light physical activity.  MLTPAS determined minutes spent in moderate/heavy physical activity – Activity Diary determined number of minutes spent in moderate/heavy physical activity.  MLTPAS and 4 days of activity diaries completed by all participants (Unstructured). | Mean_DIFF_=68; p<0.01  Mean_DIFF_=-5; NS | Pennathur (2003) |
| Cartmel et al. (1992)^[120]^ | Five City Projects Questionnaire | 66-80 yrs. | 24 | M = 9  F = 15 | FCPQ determined minutes spent in light physical activity – Activity Diary determined number of minutes spent in light physical activity.  FCPQ determined minutes spent in moderate/heavy physical activity – Activity Diary determined number of minutes spent in moderate/heavy physical activity.  FCPQ and 4 days of activity diaries completed by all participants (Unstructured). | Mean_DIFF_=-88; p<0.05  Mean_DIFF_=-170; p<0.05 | Pennathur (2003) |
| Chasan-Taber et al. (2002)^[187]^ | Self -Administered Physical Activity Questionnaire | 39-65 yrs. | 131 | F=131 | SAPAQ determined total activity (MET.hrs.wk^-1^) – Physical Activity log determined total physical activity (MET.hrs.wk^-1^)  SAPAQ determined moderate physical activity (MET.hrs.wk^-1^) – Physical Activity log determined moderate physical activity (MET.hrs.wk^-1^)  SAPAQ determined vigorous physical activity (MET.hrs.wk^-1^) – Physical Activity log determined vigorous physical activity (MET.hrs.wk^-1^)  SAPAQ determined total activity (MET.hrs.wk^-1^) – Physical Activity log determined physical activity levels (MET.hrs.wk^-1^)  SAPAQ determined moderate physical activity (MET.hrs.wk^-1^) – Physical Activity log determined physical activity levels (MET.hrs.wk^-1^)  SAPAQ determined vigorous physical activity (MET.hrs.wk^-1^) – Physical Activity log determined physical activity levels (MET.hrs.wk^-1^)  SAPAQ examined usual PA from the past year compared with 4 administrations of the physical activity log throughout the year (Unstructured). | r_p_=0.24; p<0.05  r_p_=0.18; p<0.05  r_p_=0.52; p<0.05  r_p_=0.26; p<0.05  r_p_=0.15; p<0.05  r_p_=0.52; p<0.05 | Van Poppel (2010) |
| Craig et al. (2003)^[121]^ | IPAQ -d | 18-65 yrs. | 2197 | NA | Pooled inter-instrument reliability across all measures (i.e. Long form last 7-d vs Short form last 7-d; Long form usual week vs Short form last usual week). | r_s_=0.67 (across countries) | Andrew (2010) |
| Davidson et al. (1997)^[122]^ | Polar Sports Tester 4000 | 25-54 yrs. | 9 | M = 9 | Activity Diary Energy Expenditure - Heart rate monitoring (FLEX HR) determined energy expenditure  9 days of free-living physical activity behaviours using FLEX HR calibration curves (Unstructured) | Mean _DIFF_=-12.1% | Ainslie (2003) |
| De Cocker et al. (2009)^[123]^ | International Physical Activity Questionnaire (long form) | 38.7±11.9 yrs. | 310 | M = 146  F = 164 | IPAQ-L determined total PA (mins) – IPAQ-S determined total PA (mins)  IPAQ-L determined walking (mins) – IPAQ-S determined walking (mins)  Free-living activity behaviours examined using IPAQ-L and validated with 7 days of Pedometer measurement (Unstructured). | r_s_=0.73; 95% CI = 0.67-0.78  r_s_=0.68; 95% CI = 0.62-0.74 | Kim (2013) |
| De Cocker et al. (2009)^[123]^ | International Physical Activity Questionnaire (long form) | 38.7±11.9 yrs. | 310 | M = 146  F = 164 | IPAQ-L determined total PA (mins) – MLTPAS determined total PA (mins)  IPAQ-L determined walking (mins) – MLTPAS determined walking (mins)  IPAQ-L determined MPA (mins) MLTPAS determined MPA (mins)  IPAQ-L determined VPA (mins) – MLTPAS determined VPA (mins)  Free-living activity behaviours examined using MLTPAS and validated with 7 days of Pedometer measurement (Unstructured). | r_s_=0.36; 95% CI = 0.26-0.45  r_s_=0.24; 95% CI = 0.13-0.34  r_s_=0.40; 95% CI = 0.30-0.49  r_s_=0.41; 95% CI = 0.31-0.50 | Kim (2013) |
| De Cocker et al. (2009)^[123]^ | International Physical Activity Questionnaire (long form) | 38.7±11.9 yrs. | 310 | M = 146  F = 164 | IPAQ-L determined total PA (mins) – Baecke PAQ determined total PA (index)  Free-living activity behaviours examined using IPAQ-L and validated with 7 days of Pedometer measurement (Unstructured). | r_s_=0.30; 95% CI = 0.20-0.40 | Kim (2013) |
| De Cocker et al. (2009)^[123]^ | International Physical Activity Questionnaire (short form) | 38.7±11.9 yrs. | 310 | M = 146  F = 164 | IPAQ-S determined total PA (mins) – MLTPAS determined total PA (mins)  IPAQ-S determined walking (mins) – MLTPAS determined walking (mins)  IPAQ-S determined MPA (mins) - MLTPAS determined MPA (mins)  IPAQ-S determined VPA (mins) – MLTPAS determined VPA (mins)  Free-living activity behaviours examined using IPAQ-S and validated with 7 days of Pedometer measurement (Unstructured). | r_s_=0.31; 95% CI = 0.21-0.41  r_s_=0.20; 95% CI = 0.10-0.31  r_s_=0.41; 95% CI = 0.31-0.50  r_s_=0.40; 95% CI = 0.30-0.50 | Kim (2013) |
| De Cocker et al. (2009)^[123]^ | International Physical Activity Questionnaire (short form) | 38.7±11.9 yrs. | 310 | M = 146  F = 164 | IPAQ-S determined total PA (mins) – Baecke PAQ determined total PA (index)  Free-living activity behaviours examined using IPAQ-S and validated with 7 days of Pedometer measurement (Unstructured). | r_s_=0.31; 95% CI = 0.21-0.41 | Kim (2013) |
| De Cocker et al. (2009)^[123]^ | Minnesota Leisure Time Physical Activity Questionnaire | 38.7±11.9 yrs. | 310 | M = 146  F = 164 | MLTPAS determined total PA (mins) – Baecke PAQ determined total PA (index)  Free-living activity behaviours examined using MLTPAS and validated with 7 days of Pedometer measurement (Unstructured). | r_s_=0.40; 95% CI = 0.30-0.49 | Kim (2013) |
| Deng et al. (2008)^[124]^ | IPAQ Chinese (Short form) | >50 yrs. | 224 | M = 76  W = 148 | Last 7 days short form IPAQ walking domain - 7 day pedometer step counts.  Range of self-reported physical activity variables compared to step counts (Unstructured). | r_s_=0.51; p<0.001 | Forsen (2010) |
| Dinger et al. (2006)^[126]^ | International Physical Activity Questionnaire | 20.8±1.5 yrs. | 123 | M = 32  F = 91 | IPAQ determined VPA (mins) – ActiGraph determined VPA (Freedson equation).  IPAQ determined MPA (mins) – ActiGraph determined MPA (Freedson equation).  Free-living activity behaviours examined using IPAQ and validated with 7 days of ActiGraph measurement (Unstructured). | r_s_=0.47; 95% CI = 0.32-0.62; p<0.01  r_s_=0.23; 95% CI = 0.05-0.41 | Kim (2013) |
| Dinger (2003)^[125]^ | National College Health Risk Behaviour Survey | 24.1 ± 3.5 yrs. | 20 | M = 11  F = 9 | NCHRBS determined Number of days achieving 20 mins of Vigorous Physical Activity –ActiGraph determined Number of days achieving 20 mins of Vigorous Physical Activity (Freedson cut-points).  NCHRBS determined Number of days achieving 30 mins of Moderate Physical Activity –ActiGraph determined Number of days achieving 30 mins of Moderate Physical Activity (Freedson cut-points).  NCHRBS determined Number of days achieving 20 mins of vigorous Physical Activity –Activity log determined numbers of days achieving 20 mins of vigorous Physical Activity.  NCHRBS determined Number of days achieving 30 mins of Moderate Physical Activity – Activity log determined numbers of days achieving 30 mins of moderate Physical Activity.  ActiGraph, pedometer and activity log data collected for 7 days, with previous 7 days of NCHRBS reported (Unstructured). | r_p_=0.60; p<0.01  r_p_=0.61; p<0.01  r_p_=0.82; p<0.01  r_p_=0.66; p<0.05 | Prince (2008) |
| Dishman et al. (1998)^[127]^ | 7 day recall diary (self-administered) | 21.9 ± 2.9 yrs. | 158 | M = 87  F = 71 | 7DRD determined kcal.kg^-1^.wk^-1^ – Activity diary determined kcal.kg^-1^.wk^-1^.  7DRD determined kcal.kg^-1^.wk^-1^ – Historical physical activity questionnaire determined kcal.kg^-1^.wk^-1^.  Participants completed the 7 day activity diary and then completed the recall questionnaire on completion of diary (Unstructured). | r=0.82; p<0.01  r=0.61; p<0.001 | Nielson (2008) |
| Dishman et al. (1998)^[127]^ | 7 day recall diary (interview-administered) | 21.9 ± 2.9 yrs. | 158 | M = 87  F = 71 | 7DRD determined kcal.kg^-1^.wk^-1^ – Activity diary determined kcal.kg^-1^.wk^-1^  Participants completed the 7 day activity diary and then completed the recall questionnaire on completion of diary (Unstructured). | r=0.81; p<0.01 | Nielson (2008) |
| Duncan et al. (2001)^[128]^ | 7 day Physical Activity Recall Questionnaire | 47.8±7.1 yrs. | 94 | M = 18  F = 76 | 7DPARQ determined very hard activity - Heart Rate Monitor determined very hard activity (60-84% HRR).  7DPARQ determined hard activity - Heart Rate Monitor determined hard activity (≥85% HRR).  7DPARQ determined hard activity - Heart Rate Monitor determined hard activity 45-59% HRR).  Free-living activity behaviours examined using 7DPARQ and validated with 1 weekday of HRM measurement (Unstructured). | Mean_DIFF_=0.00 hrs.  Mean_DIFF_=0.02 hrs.  Mean_DIFF_=0.21 hrs. | Helmorhorst (2012) |
| Ekelund et al. (2006)^[129]^ | IPAQ Short Form Last 7 Days | 20-69 yrs. | 185 | M = 87  F = 98 | Total Self-reported MVPA per day – ActiGraph MVPA (Ekelund cut-points). | r_p_= 0.17; p<0.05 | Andrew (2010) |
| Ekelund et al. (2006)^[129]^ | IPAQ Short Form Last 7 Days | 20-69 yrs. | 185 | M = 87  F = 98 | Total Self-reported activity mins per day – ActiGraph activity mins per day (>100 counts). | r_p_= 0.17; p<0.05 | Andrew (2010) |
| Fjeldsoe et al. (2009)^[130]^ | Australian Women’s Activity Survey | 33±5 yrs. | 75 | F = 75 | AWAS determined Health Enhancing Physical activity (mins/wk) –ActiGraph determined HEPA (Freedson cut points)  AWAS determined Health Enhancing Physical activity (mins/wk) –ActiGraph determined HEPA (Swartz cut points)  AWAS examined average activity of last week with ActiGraph device worn during 7 days of free-living activity behaviours (Unstructured). | r_s_=0.28; p<0.05  r_s_=0.06; NS | Helmorhorst (2012) |
| Friedenreich et al. (2006)^[131]^ | Past Year Total Physical Activity Questionnaire | 35-65 yrs. | 154 | M = 75  F = 79 | PYTPAQ determined total MET-hr/wk –ActiGraph determined total total MET-hr/wk (Swartz cut points)  PYTPAQ examined average activity of last year with ActiGraph device worn during 7 days of free-living activity behaviours (Unstructured). | r_s_=0.26; p <0.05  ICC=0.18; 95% CI = 0.03-0.32 | Helmorhorst (2012) |
| Fuller et al. (2008)^[79]^ | Physical Activity Diary | 20-66 yrs. | 60 | M = 30  F = 30 | PAD determined energy expenditure (MJ.d^-1^) – Heart rate monitor determined energy expenditure (MJ.d^-1^) (from calibrated FLEX HR)  PAD determined energy expenditure (MJ.d^-1^) – Caltrac determined energy expenditure (MJ.d^-1^)  PAD determined energy expenditure (MJ.d^-1^) –7DPAR determined energy expenditure (MJ.d^-1^)  15 days of DLW measurement, HRM, Physical Activity Diary, Caltrac device and 2 versions of the 7DPARQ (Day 8 and day 15 (Unstructured). | r=0.54  r=0.48  r=0.73 | Plasqui (2013) |
| Fuller et al. (2008)^[79]^ | Physical Activity Diary | 20-66 yrs. | 60 | M = 30  F = 30 | PAD determined energy expenditure (PAL) – Heart rate monitor determined energy expenditure (PAL) (from calibrated FLEX HR)  PAD determined energy expenditure (PAL) – Caltrac determined energy expenditure (PAL)  PAD determined energy expenditure (PAL) –7DPAR determined energy expenditure (PAL)  15 days of DLW measurement, HRM, Physical Activity Diary, Caltrac device and 2 versions of the 7DPARQ (Day 8 and day 15 (Unstructured). | r=0.00  r=0.23  r=0.11 | Plasqui (2013) |
| Fuller et al. (2008)^[79]^ | 7 day physical activity recall questionnaire | 20-66 yrs. | 60 | M = 30  F = 30 | 7DPARQ determined energy expenditure (MJ.d^-1^) – Heart rate monitor determined energy expenditure (MJ.d^-1^) (from calibrated FLEX HR)  7DPARQ determined energy expenditure (MJ.d^-1^) – Caltrac determined energy expenditure (MJ.d^-1^)  7DPARQ determined energy expenditure (MJ.d^-1^) – PAD determined energy expenditure (MJ.d^-1^)  15 days of DLW measurement, HRM, Physical Activity Diary, Caltrac device and 2 versions of the 7DPARQ (Day 8 and day 15 (Unstructured). | r=0.51  r=0.53  r=0.73 | Plasqui (2013) |
| Fuller et al. (2008)^[79]^ | 7 day physical activity recall questionnaire | 20-66 yrs. | 60 | M = 30  F = 30 | 7DPAR determined energy expenditure (PAL) – Heart rate monitor determined energy expenditure (PAL) (from calibrated FLEX HR)  7DPAR determined energy expenditure (PAL) – Caltrac determined energy expenditure (PAL)  7DPAR determined energy expenditure (PAL) – PAD determined energy expenditure (PAL)  15 days of DLW measurement, HRM, Physical Activity Diary, Caltrac device and 2 versions of the 7DPARQ (Day 8 and day 15 (Unstructured). | r=0.03  r=0.20  r=0.11 | Plasqui (2013) |
| Grimm et al. (2012)^[132]^ | IPAQ-Short form | 50-87 yrs. | 127 | M = 31  F = 96 | IPAQ determined moderate walking – ActiGraph 7164 determined moderate lifestyle activities  IPAQ determined vigorous – ActiGraph 7164 determined vigorous  7 days of free living activity behaviour from accelerometer with previous 7 days recall from IPAQ (Unstructured). | r_s_=0.164; NS  r_s_=0.176; NS | Gorman (2014) |
| Hagstromer et al. (2010)^[133]^ | International Physical Activity Questionnaire | 18-65 yrs. | 980 | M = 443  F = 537 | IPAQ determined total mins/day – ActiGraph determined total mins/day.  Free-living activity behaviours examined using IPAQ and validated with 7 days of ActiGraph measurement (Unstructured). | r_s_=0.28; p<0.01 | Helmorhorst (2012) |
| Hallal et al. (2010)^[134]^ | International Physical Activity Questionnaire | 40.3±15.1 yrs. | 156 | M = 75  F = 81 | IPAQ determined Total Score (minutes/week) – ActiGraph determined Total Score (MVPA minutes/week determined by Freedson equations).  Free-living activity behaviours examined using IPAQ (phone administered) and validated with 4 days of ActiGRaph measurement (Unstructured). | r_s_=0.22 | Helmorhorst (2012) |
| Hallal et al. (2004)^[135]^ | International Physical Activity Questionnaire (short form) | 40.0±17.1 yrs. | 186 | M = 67  F = 119 | IPAQ-s determined moderate to vigorous PA (mins/week) – IPAQ-l determined moderate to vigorous PA (mins/week).  IPAQ-s and IPAQ-l administered one after the other (order reversed for 50% of group)(Unstructured). | r_p_=0.61; p<0.001  %Agreement = 78.5%  Kappa=53.7% | Kim (2013) |
| Hart et al. (2011) | Bouchard Activity Record | 18-60 yrs. | 32 | M = 16  F = 16 | BAR determined minutes spent stepping – ActiGraph determined minutes spent stepping  BAR determined minutes spent MVPA – ActiGraph determined minutes spent MVPA (Freedson threshold)  Single day of physical activity monitoring (Unstructured). | Mean_DIFF_= -28.1 mins; p<0.001.  %Agr =58.9%  Mean_DIFF_= 96.0 mins; p<0.001  %Agr =86.7% | Gorman (2014) |
| Hart et al. (2011) | Bouchard Activity Record | 18-60 yrs. | 32 | M = 16  F = 16 | BAR determined minutes spent standing – activPAL determined minutes spent standing  BAR determined minutes spent stepping – activPAL determined minutes spent standing  Single day of physical activity monitoring (Unstructured). | Mean_DIFF_= -49.0 mins; p<0.01  %Agr =74.0%  Mean_DIFF_= 65.0 mins; NS  %Agr =79.1% | Gorman (2014) |
| Hekler et al. (2012)^[136]^ | CHAMPS | 66-80 yrs. | 870 | M = 377  F = 493 | CHAMPS determined mins/wk Low LIPA- Actigraph determined mins/wk Low LIPA (Copeland)  CHAMPS determined mins/wk High LIPA- Actigraph determined mins/wk High LIPA (Copeland)  CHAMPS determined mins/wk MVPA- Actigraph determined mins/wk MVPA (Copeland)  CHAMPS determined mins/wk Total PA- Actigraph determined mins/wk Total PA (Copeland)  CHAMPS determined caloric expenditure/wk MVPA - Actigraph determined mins/wk MVPA (Copeland)  CHAMPS determined caloric expenditure/wk Total PA - Actigraph determined mins/wk Total PA (Copeland)  7 days of free-living activity monitoring (Unstructured). | r_s_=0.06; NS  r_s_=0.27; p<0.001  r_s_=0.37; p<0.001  r_s_=0.38; p<0.001  r_s_=0.38; p<0.001  r_s_=0.39; p<0.001 | Gorman (2014) |
| Hopkins et al. (1991)^[137]^ | Life in New Zealand National Survey | 18-79 yrs. | 67 | NA | LNZNS determined High Intensity PA – Stanford 7 day recall questionnaire determined Hard physical activity  LNZNS determined Moderate Intensity PA – Stanford 7 day recall questionnaire determined moderate physical activity  LNZNS determined Low Intensity PA – Stanford 7 day recall questionnaire determined moderate physical activity  LNZNS examined average activity behaviour from previous 4 weeks compared with previous 7 days from Stanford PA questionnaire (Unstructured). | r=0.37; p<0.05  r=0.30; p<0.05  r=0.32; p<0.05 | Shephard (2003) |
| Hurtig-Wennlöf et al. (2010)^[138]^ | IPAQ (elderly) | 66-91 yrs. | 54 | M = 23  F = 31 | IPAQ determined MPA (mins/d) - Actigraph GT1M determined moderate (Matthews).  IPAQ determined VPA (mins/d) - Actigraph GT1M determined VPA (Matthews).  7 days of free-living activity monitoring (Unstructured). | r_s_=0.41; p<0.01  r_s_=0.40; p<0.01 | Gorman (2014) |
| Kurtze et al. (2008)^[139]^ | International Physical Activity Questionnaire | 20-39 yrs. | 108 | M = 108 | Self-administered IPAQ determined total active min/wk. –ActiReg determined total active min/wk.  IPAQ determined behaviours for last 7 days and ActiReg worn for 7 consecutive days (Unstructured). | Mean_DIFF_=-433 mins/wk. | Helmorhorst (2012) |
| Kwak et al. (2007)^[188]^ | SQUASH Physical Activity Questionnaire | 36.8 ± 7.3 yrs. | 103 | M=63  F=40 | SQUASH determined MVPA – MTI ActiGraph determined MVPA (Swartz cut points)  SQUASH determined physical activities in the usual week compared to 7 days of ActiGraph wear (Unstructured). | r=0.27; NS | Van Poppel (2010) |
| Kwak et al. (2007)^[188]^ | 24 hr recall Physical Activity Scale | 36.8 ± 7.3 yrs. | 103 | M=63  F=40 | PAS determined activity score (MET time) – MTI ActiGraph determined MVPA (Swartz cut points)  7 days of ActiGraph wear with PAS completed daily throughout the ActiGraph wear protocol (Unstructured). | r=0.66; 95%CI = 0.29-0.82; p<0.001 | Van Poppel (2010) |
| Lagerros et al. (2006)^[189]^ | Energy expenditure questionnaire | 20-59 yrs. | 293 | M=137  F=156 | EEQ determined daily energy expenditure (kcal.d^-1^) – 3 24 hr. interview administered recall questionnaire determined mean daily energy expenditure (kcal.d^-1^).  EEQ determined usual daily physical activity compared to mean of three 24 hour PA recall questionnaires completed 4 months after EEQ completed (Unstructured). | r_p_=0.73; CCC=0.51; Mean_DIFF_=680 kcal.d^-1^; Median_DIFF_=370 kcal.d^-1^. | Van Poppel (2010) |
| Lee et al. (2011)^[140]^ | International Physical Activity Questionnaire | 42.9±14.4 yrs. | 1270 | M = 585  F = 685 | IPAQ determined total MET-min/week –ActiGraph determined total MET-min/week (Freedson equation)  Free-living activity behaviours examined for 4 days using Tracmor and 7 days using IPAQ (Unstructured). | r_s_=0.11 (SE=0.03) p<0.001 | Helmorhorst (2012) |
| Leenders et al. (2000)^[141]^ | 7-day physical activity recall | 26±6 yrs. | 12 | F = 12 | Physical activity Energy expenditure from 7-d PAR – ActiGraph determined PAEE (Freedson)  7 day free living activity behaviours (Unstructured). | r=0.82; p<0.001 | Berlin (2006) |
| Leenders et al. (2000)^[141]^ | 7-day physical activity recall | 26±6 yrs. | 12 | F = 12 | Physical activity Energy expenditure from 7-d PAR – Tritrac determined PAEE  7 day free living activity behaviours (Unstructured). | r=0.89; p<0.001 | Berlin (2006) |
| Leenders et al. (2000)^[141]^ | 7-day physical activity recall | 26±6 yrs. | 12 | F = 12 | Physical activity Energy expenditure from 7-d PAR – Digi-Walker determined PAEE  7 day free living activity behaviours (Unstructured). | r=0.89; p<0.001 | Berlin (2006) |
| Lemmer et al. (2001)^[142]^ | Stanford 7 day physical activity recall questionnaire | 20—75 yrs. | 40 | M = 20  F = 20 | S7DPAR determined energy expenditure (kJ.d^-1^) – Tritrac determined physical activity energy expenditure (kJ.d^-1^)  4 days of physical activity assessed using Tritrac compared to 7 day recall from Tritrac RT3 (Unstructured). | r=0.71; p<0.001 | Prince (2008) |
| MacFarlane et al. (2011)^[143]^ | International Physical Activity Questionnaire | 26.2±9.9 yrs. | 83 | M = 47  F = 36 | IPAQ determined Total MET-mins/day – ActiTrainer determined total MET-mins/day.  Free-living activity behaviours examined using IPAQ and validated with 7 days of ActiTrainer measurement (Unstructured). | r_s_=0.35; p<0.01  Mean_DIFF_=-21.6± 575.5 MET-mins/day | Helmorhorst (2012) |
| MacFarlane et al. (2007)^[144]^ | International Physical Activity Questionnaire | 15-55 yrs. | 49 | M = 30  F = 19 | IPAQ determined total minutes/week – ActiGraph determined total MVPA mins/week (Freedson equations)  Free-living activity behaviours examined for 7 days using IPAQ and ActiGraph (Unstructured). | r_s_=0.09; NS | Helmorhorst (2012) |
| MacFarlane et al (2006)^[145]^ | Physical Activity log | 15-55 yrs. | 49 | M = 30  F = 19 | PA Log determined light intensity activity – IPAQ Last 7-d determined light intensity activity  PA Log determined light intensity activity – HR determined light intensity activity (Howley cut points)  PA Log determined light intensity activity – Tritrac determined light intensity activity (Nichols cut points)  PA Log determined light intensity activity – MTI determined light intensity activity (Freedson cut points)  Free-living physical activity behaviours over 7 days using each measure (Unstructured). | r_s Light_=0.25  r_s Light_ =0.06  r_s Light_ =0.22  r_s Light_ =0.21 | Andrew (2010) |
| MacFarlane et al (2006)^[145]^ | Physical Activity log | 15-55 yrs. | 49 | M = 30  F = 19 | PA Log determined moderate intensity activity – IPAQ Last 7-d determined moderate intensity activity  PA Log determined moderate intensity activity – HR determined moderate intensity activity (Howley cut points)  PA Log determined moderate intensity activity – Tritrac determined moderate intensity activity (Nichols cut points)  PA Log determined moderate intensity activity – MTI determined moderate intensity activity (Freedson cut points)  Free-living physical activity behaviours over 7 days using each measure (Unstructured). | r_s Moderate_=0.68; p<0.001  r_s Moderate_ = -0.26  r_s Moderate_ =0.32; p<0.05  r_s Moderate_ =0.10 | Andrew (2010) |
| MacFarlane et al (2006)^[145]^ | Physical Activity log | 15-55 yrs. | 49 | M = 30  F = 19 | PA Log determined vigorous intensity activity – IPAQ Last 7-d determined vigorous intensity activity  PA Log determined vigorous intensity activity – HR determined vigorous intensity activity (Howley cut points)  PA Log determined vigorous intensity activity – Tritrac determined vigorous intensity activity (Nichols cut points)  PA Log determined vigorous intensity activity – MTI determined vigorous intensity activity (Freedson cut points)  Free-living physical activity behaviours over 7 days using each measure (Unstructured). | r_s Vigorous_=0.72; p<0.001  r_s Vigorous_= 0.61; p<0.001  r_s Vigorous_=0.22  r_s Vigorous_=0.45; p<0.01 | Andrew (2010) |
| MacFarlane et al (2006)^[145]^ | IPAQ Short Form Last 7 days | 15-55 yrs. | 49 | M = 30  F = 19 | IPAQ Last 7-d determined light intensity activity - PA Log determined light intensity activity  IPAQ Last 7-d determined light intensity activity – HR determined light intensity activity (Howley cut points)  IPAQ Last 7-d determined light intensity activity – Tritrac determined light intensity activity (Nichols cut points)  IPAQ Last 7-d determined light intensity activity – MTI determined light intensity activity (Freedson cut points)  Free-living physical activity behaviours over 7 days using each measure (Unstructured). | r_s Light_ =0.25  r_s Light_ =0.24  r_s Light_ =0.35; p<0.05  r_s Light_ =0.37; p<0.01 | Andrew (2010) |
| MacFarlane et al (2006)^[145]^ | IPAQ Short Form Last 7 days | 15-55 yrs. | 49 | M = 30  F = 19 | IPAQ Last 7-d determined moderate intensity activity - PA Log determined moderate intensity activity  IPAQ Last 7-d determined moderate intensity activity – HR determined moderate intensity activity (Howley cut points)  IPAQ Last 7-d determined moderate intensity activity – Tritrac determined moderate intensity activity (Nichols cut points)  IPAQ Last 7-d determined moderate intensity activity – MTI determined moderate intensity activity (Freedson cut points)  Free-living physical activity behaviours over 7 days using each measure (Unstructured). | r_s Moderate_ =0.68; p<0.001  r_s Moderate_ = -0.11;  r_s Moderate_ =0.15  r_s Moderate_ = -0.06 | Andrew (2010) |
| MacFarlane et al (2006)^[145]^ | IPAQ Short Form Last 7 days | 15-55 yrs. | 49 | M = 30  F = 19 | IPAQ Last 7-d determined vigorous intensity activity - PA Log determined vigorous intensity activity  IPAQ Last 7-d determined vigorous intensity activity – HR determined vigorous intensity activity (Howley cut points)  IPAQ Last 7-d determined vigorous intensity activity – Tritrac determined vigorous intensity activity (Nichols cut points)  IPAQ Last 7-d determined vigorous intensity activity – MTI determined vigorous intensity activity (Freedson cut points)  Free-living physical activity behaviours over 7 days using each measure (Unstructured). | r_s Vigorous_=0.72; p<0.001  r_s Vigorous_= 0.56; p<0.001  r_s Vigorous_=0.18  r_s Vigorous_=0.44; p<0.01 | Andrew (2010) |
| Maddison et al. (2007)^[82]^ | New Zealand Physical Activity Questionnaire (NZPAQ) | 18-64 yrs. | 36 | M = 16  F = 20 | NZPAQ energy expenditure - IPAQ energy expenditure  Assessed at 3 time points over 15 days of free-living physical activity (Unstructured). | r_s_=0.74; p<0.01 | Westerterp (2009) |
| Mader et al. (2006)^[146]^ | IPAQ Short Form | 15-75 yrs. | 178 | M = 101  F = 77 | Self-reported IPAQ Moderate mins - ActiGraph determined Moderate mins  Self-reported IPAQ vigorous mins - ActiGraph determined vigorous mins (Swartz cut points). | r_s_=0.27  r_s_= -0.03 | Andrew (2010) |
| Mader et al. (2006)^[146]^ | Office In Motion Questionnaire (OIMQ) | 15-75 yrs. | 178 | M = 101  F = 77 | Self-reported OIMQ Moderate mins - ActiGraph determined Moderate mins  Self-reported OIMQ vigorous mins - ActiGraph determined vigorous mins (Swartz cut points). | r_s_=0.53; p<0.05  r_s_=0.08 | Andrew (2010) |
| Mahabir et al. (2006)^[96]^ | Harvard Alumni Questionnaire  (Self admin) | 49 – 79 yrs. | 65 | F = 65 | HAQ Energy Expenditure – Five City Project Questionnaire energy expenditure  HAQ Energy Expenditure - CCAPS 4 week activity recall Energy Expenditure  HAQ Energy Expenditure - Typical week activity recall questionnaire Energy Expenditure  Weighted METs/h computed an converted to kcal/d for questionnaire and compared against DLW kcal/d. (Unstructured). | r_s_=0.77; p<0.05  r_s_=0.32; p<0.05  r_s_=0.38; p<0.05 | Westerterp (2009) |
| Mahabir et al. (2006)^[96]^ | Five City Project Questionnaire  (Self admin) | 49 – 79 yrs. | 65 | F = 65 | Five City Project Questionnaire energy expenditure - HAQ energy expenditure  Five City Project Questionnaire energy expenditure - CCAPS 4 week activity recall Energy Expenditure  Five City Project Questionnaire energy expenditure - Typical week activity recall questionnaire Energy Expenditure  Weighted METs/h computed an converted to kcal/d for questionnaire and compared against DLW kcal/d. (Unstructured). | r_s_=0.77; p<0.05  r_s_=0.40; p<0.05  r_s_=0.33; p<0.05 | Westerterp (2009) |
| Mahabir et al. (2006)^[96]^ | Cross-Cultural Activity Participation Study Four Week  Activity Recall  (Self admin) | 49 – 79 yrs. | 65 | F = 65 | CCAPS 4 week activity recall Energy Expenditure - HAQ Energy Expenditure  CCAPS 4 week activity recall Energy Expenditure – Five City Project Questionnaire energy expenditure  CCAPS 4 week activity recall Energy Expenditure - Typical week activity recall questionnaire Energy Expenditure  Weighted METs/h computed an converted to kcal/d for questionnaire and compared against DLW kcal/d. (Unstructured). | r_s_=0.32; p<0.05  r_s_=0.40; p<0.05  r_s_=0.73; p<0.05 | Westerterp (2009) |
| Mahabir et al. (2006)^[96]^ | Typical Week Activity Recall  questionnaires | 49 – 79 yrs. | 65 | F = 65 | Typical week activity recall questionnaire Energy Expenditure - HAQ Energy Expenditure  Typical week activity recall questionnaire Energy Expenditure – Five City Project Questionnaire energy expenditure  Typical week activity recall questionnaire Energy Expenditure - CCAPS 4 week activity recall Energy Expenditure  Weighted METs/h computed an converted to kcal/d for questionnaire and compared against DLW kcal/d. (Unstructured). | r_s_=0.38; p<0.05  r_s_=0.33; p<0.05  r_s_=0.73; p<0.05 | Westerterp (2009) |
| Masse et al. (2004)^[83]^ | Physical Activity Diary | 40-70 yrs. | 136 | F = 136 | Physical activity diary determined kcal/d – ActiGraph determined counts/d  PAD collected on second week of DLW measurement during free-living activity behaviours (Unstructured). | r_p_=0.43 | Corder (2007) |
| Masse et al. (2005)^[147]^ | Women on the Move 7 day activity diary | 49.3 ± 7.1 yrs. | 224 | F = 224 | WOTM7DAD determined energy expenditure (using compendium coding) (MET.mins.d^-1^) – ActiGraph CSA determined energy expenditure (MET.mins.d^-1^)  WOTM7DAD determined energy expenditure (using participant rating) (MET.mins.d^-1^) – ActiGraph CSA determined energy expenditure (MET.mins.d^-1^)  WOTM7DAD determined energy expenditure (using revised participant rating) (MET.mins.d^-1^) – ActiGraph CSA determined energy expenditure (MET.mins.d^-1^)  7 days of monitoring using both the CSA and activity diary (Unstructured). | r_s_=0.305; p<0.05; % Within 500 MET-min=47.9  r_s_=0.273; p<0.05; % Within 500 MET-min=0.0  r_s_=0.264; p<0.05; % Within 500 MET-min=40.8 | Prince (2008) |
| Matthews et al. (1995)^[149]^ | 3 Day Physical Activity Log | 20-37 yrs. | 25 | M = 14  F = 11 | 3DPAL determined energy expenditure (kcal/d) – Tritrac determined energy expenditure (kcal/d).  7 days of monitoring of free-living activity behaviours (Unstructured). | Mean_DIFF_= 362.4 kcal/d  r=0.76-0.83; p<0.001 | Freedson (2000) |
| Matthews et al. (1995)^[149]^ | 7 Day Physical Activity Recall Questtionnaire | 20-37 yrs. | 25 | M = 14  F = 11 | 7DPAR determined energy expenditure (kcal/d) – Tritrac determined energy expenditure (kcal/d).  7 days of monitoring of free-living activity behaviours (Unstructured). | Mean_DIFF_= 310.3 kcal/d | Freedson (2000) |
| Matthews at al. (2003)^[151]^ | Shanghai Women’s Health Study Physical  Activity Questionnaire | 55.3 ± 8.9 yrs. | 191 | F = 191 | SWHSPAQ determined duration in PA per week – Physical activity log determined duration in PA per week.  SWHSPAQ determined duration in PA per week – Physical activity questionnaire determined duration in PA per week.  SWHSPAQ determined energy expenditure (MET hrs.wk^-1^) – Physical activity log determined energy expenditure (MET hrs.wk^-1^).  SWHSPAQ determined energy expenditure (MET hrs.wk^-1^) – Physical activity questionnaire determined energy expenditure (MET hrs.wk^-1^).  SWHSPAQ determined transportation (min.d^-1^) – Physical activity log determined transportation (min.d^-1^)  SWHSPAQ determined transportation (min.d^-1^) – Physical activity questionnaire determined transportation (min.d^-1^)  SWHSPAQ determined walking (mins.d^-1^) – Physical activity log determined walking (mins.d^-1^)  SWHSPAQ determined walking (mins.d^-1^) – Physical activity questionnaire determined walking (mins.d^-1^)  SWHSPAQ determined housework (hrs.d^-1^) – Physical activity log determined housework (hrs.d^-1^)  SWHSPAQ determined housework (hrs.d^-1^)– Physical activity questionnaire determined housework (hrs.d^-1^) | r_s_=0.49  r_s_=0.61  r_s_=0.50  r_s_=0.62  r_s_=-0.04  r_s_=0.67  r_s_=0.14  r_s_=0.33  r_s_=0.36  r_s_=0.46 | Nielson (2008) |
| Matthews et al. (2013)^[150]^ | Interview Administered Previous Day Recall Questionnaire | 12-17 yrs. | 91 | M = 43  F = 48 | PDRQ determined active time (hrsd^-1^) - activPAL determined active time (hrsd^-1^).  Participants completed 3 PDRQ over a 7 day period compared to7 days of ActiGraph measured free-living physical activity (Unstructured). | Mean_DIFF_=-0.24 - -0.30; CV_DIFF_% = 29%-32%; r_s_=0.15-0.25; NS | Pedisic (2014) |
| Matthews et al. (2013)^[150]^ | Interview Administered Previous Day Recall Questionnaire | 18-71 yrs. | 88 | M = 40  F = 48 | PDRQ determined active time (hrsd^-1^) - activPAL determined active time (hrsd^-1^).  Participants completed 3 PDRQ over a 7 day period compared to7 days of ActiGraph measured free-living physical activity (Unstructured). | Mean_DIFF_=-0.05 - -0.20; CV_DIFF_% = 21%-35%; r_s_=0.43-0.48; p<0.05 | Pedisic (2014) |
| Matthews et al. (2005)^[148]^ | Short Telephone Administers Recall Questionnaire | 46 yrs. | 88 | M = 40  F = 48 | STAR-Q (Closed Question) determined MVPA frequency in usual week (d^-1^) – ActiGraph determined 30 minutes of MVPA (Matthews equation).  STAR-Q (Closed Question) determined MVPA duration (mins.d^-1^) – ActiGraph determined mins.d^-1^ of MVPA (Matthews equation).  STAR-Q (Closed Question) determined MVPA duration (mins.wk^-1^) – ActiGraph determined mins.wk^-1^ of MVPA (Matthews equation).  Participants wore an ActiGraph accelerometer and completed the STAR-Q at the end of the measurement period (Unstructured). | p=0.00  p=0.22  p=0.01 | Matthews (2005) |
| Matthews et al. (2005)^[148]^ | Short Telephone Administers Recall Questionnaire | 46 yrs. | 88 | M = 40  F = 48 | STAR-Q (Open Question) determined MVPA frequency in usual week (d^-1^) – ActiGraph determined 30 minutes of MVPA (Matthews equation).  STAR-Q (Open Question) determined MVPA duration (mins.d^-1^) – ActiGraph determined mins.d^-1^ of MVPA (Matthews equation).  STAR-Q (Open Question) determined MVPA duration (mins.wk^-1^) – ActiGraph determined mins.wk^-1^ of MVPA (Matthews equation).  Participants completed the 24 hours PAR on XXX days and the STAR-Q (Unstructured). | p=0.00  p=0.03  p=0.00 | Matthews (2005) |
| Meriwether et al. (2006)^[152]^ | Physical Activity Assessment Tool | 18-64 yrs. | 68 | M = 10  F = 58 | PAAT determined VPA mins/week – ActiGraph determined VPA mins/week (Freedson equations)  PAAT determined MVPA mins/week – ActiGraph determined MVPA mins/week (Freedson equations)  PAAT determined MPA mins/week – ActiGraph determined MPA mins/week (Freedson equations)  14 days of free living physical activity examined using both PAAT and activity monitor (Unstructured). | r_s_=0.38; p<0.01  r_s_=0.392; p<0.01  r_s_=0.392; p<0.01 | Helmorhorst (2012) |
| Moy et al. (2008)^[153]^ | New Zealand Physical Activity Questionnaire | 48.6 ± 16.4 yrs. | 180 | M = 87  F = 93 | NZPAQ-Short determined minutes spent in brisk walking – Heart Rate monitor determined minutes spent in brisk walking.  NZPAQ-Short determined minutes spent in moderate PA – Heart Rate monitor determined minutes spent in moderate PA.  NZPAQ-Short determined minutes spent in moderate PA – Heart Rate monitor determined minutes spent in moderate PA.  NZPAQ-Long determined minutes spent in brisk walking – Heart Rate monitor determined minutes spent in brisk walking.  NZPAQ- Long determined minutes spent in moderate PA – Heart Rate monitor determined minutes spent in moderate PA.  NZPAQ- Long determined minutes spent in moderate PA – Heart Rate monitor determined minutes spent in moderate PA.  NZPAQ-Short determined meeting recommendations – Heart Rate monitor determined meeting recommendations.  NZPAQ-Long determined meeting recommendations – Heart Rate monitor determined meeting recommendations.  Physical activity log and simultaneous heart rate monitoring for 3 consecutive days (Unstructured). | r_s_=0.27; p<0.01; 95%CI = 0.12-0.42.  r_s_=0.07; NS; 95%CI = -0.08-0.22.  r_s_=0.27; p<0.01; 95%CI = 0.12-0.42.  r_s_=0.43; p<0.001; 95%CI = 0.28-0.58  r_s_=0.02; NS; 95%CI = -0.13-0.17.  r_s_=0.35; p<0.001; 95%CI = 0.20-0.50  Kappa=0.12  Kappa=0.12 | Warren (2010) |
| Nang et al. (2011)^[154]^ | International Physical Activity Questionnaire | 38.3±12.9 yrs. | 152 | M = 64  F = 88 | IPAQ determined kcal/d for VPA –Actical determined kcal/d for VPA.  IPAQ determined kcal/d for MPA –Actical determined kcal/d for MPA.  IPAQ determined behaviours for previous 7 days and Actical worn for 5 consecutive days (Unstructured). | r_s_=0.18; p<0.05  r_s_=0.13 | Helmorhorst (2012) |
| Nang et al. (2011)^[154]^ | Singapore Prospective Study Program Physical Activity Questionnaire | 38.3±12.9 yrs. | 152 | M = 64  F = 88 | SPSPPAQ determined kcal/d for VPA–Actical determined kcal/d for VPA.  SPSPPAQ determined kcal/d for MPA –Actical determined kcal/d for MPA.  SPSPPAQ determined behaviours for previous 3 months and Actical worn for 5 consecutive days (Unstructured). | r_s_=0.42; p<0.001  r_s_=0.24; p<0.001 | Helmorhorst (2012) |
| Nicaise et al. (2011)^[155]^ | International Physical Activity Questionnaire | 35.9±9.0 yrs. | 105 | F = 105 | IPAQ determined VPA – ActiGraph determined VPA (Freedson equations)  IPAQ determined MPA – ActiGraph determined MPA (Freedson equations)  Free-living activity behaviours examined for 7 days using both IPAQ and ActiGraph (Unstructured). | r_p_=-0.01  r_p_=0.08 | Helmorhorst (2012) |
| Norman et al. (2001)^[156]^ | Physical activity questionnaire (self-administered) | 44-78 yrs. | 111 | M = 111 | PAQ determined MET hours per day^-1^ (crude total activity) – Physical Activity records determined MET hours per day (crude total activity)  PAQ determined MET hours per day^-1^ (total activity score) – Physical Activity records determined MET hours per day (total activity score)  Usual physical activity in previous year from physical activity questionnaire compared with 7 day physical activity diary (Unstructured). | r_s_=0.22; p<0.05  r_s_=0.53; p<0.05 | Kwak (2011) |
| Resnick et al. (2008)^[157]^ | Yale Physical Activity Survey | CHAT: 60.6 ± 5.5 yrs.  HIP: 80.7 ± 6.8 yrs.  SENIOR: 76.2 ± 6.2 yrs. | 218  150  150 | M = 65  F = 163  M = NA  F = NA  M = 45  F = 105 | YPAS determined time in physical activity – CHAMPS determined time in physical activity.  YPAS determined time in MPA – CHAMPS determined time in MPA.  YPAS determined energy expended during PA – CHAMPS determined energy expended during PA.  YPAS determined energy expended during MPA – CHAMPS determined energy expended during MPA | r_p_=0.24-0.64; p≤0.05  r_p_=0.17-0.57; p≤0.05  r_p_=0.27-0.66; p≤0.05  r_p_=0.11-0.51; p≤0.05 | Kowalski (2012) |
| Roman Vinas et al. (2010)^[160]^ | International Physical Activity Questionnaire | 20-68 yrs. | 75 | M = 42  F = 33 | IPAQ determined VPA (mins) – ActiGraph MTI determined VPA (Freedson equation).  IPAQ determined MPA (mins) – ActiGraph MTI determined MPA (Freedson equation).  IPAQ determined MPA and walking (mins) – ActiGraph MTI determined MPA (Freedson equation).  Free-living activity behaviours examined using IPAQ and validated with a minimum of 7 days of MTI ActiGraph measurement (Unstructured). | r_s_=0.30; p<0.05  r_p_=0.15  r_p_=0.10 | Kim (2013) |
| Richardson et al. (2001)^[158]^ | Stanford 7 day Recall Questionnaire | 20-59 yrs. | 77 | M = 27  F = 50 | S7DRQ determined total MET-min/day – Caltrac determined total MET-min/day (Men).  S7DRQ determined total MET-min/day – Caltrac determined total MET-min/day (Women).  Free-living activity behaviours examined for 14 days (twice) using S7DRQ and Caltrac (Unstructured). | r_s_=0.45-0.54; p<0.05  r_s_=0.06-0.20; NS | Helmorhorst (2012) |
| Richardson et al. (1995)^[159]^ | Minnesota Leisure Time Physical Activity Survey | 20-59 yrs. | 78 | M = 28  W = 50 | MLTPAS determined mean METs/d – Caltrac Determined METs.  28 days of Caltrac readings over 1 year compared to 4 week version of the MLTQ obtained over 1 year period (Unstructured). | r_TOTAL_=0.30  r_MODERATE_=0.23  r_HEAVY_=0.36 | Berlin (2003) |
| Richardson et al. (1995)^[159]^ | Physical Activity Record | 20-59 yrs. | 78 | M = 28  W = 50 | PA record determined mean METs/d – Caltrac Determined METs.  48 hour physical activity record and 28 days of Caltrac readings every 26 days over 1 year compared (Unstructured). | r_TOTAL_=0.51  r_HEAVY_=0.34 | Berlin (2003) |
| Saglam et al. (2010)^[161]^ | International Physical Activity Questionnaire (long) | 18–32 yrs. | 80 | NA | IPAQ determined Total MET-mins/day – Caltrac determined total energy expenditure.  Free-living activity behaviours examined using IPAQ (long) and validated with 7 days of Caltrac measurement (Unstructured). | r_s_=0.29; 95% CI = 0.05-0.47; p<0.01 | Helmorhorst (2012) |
| Saglam et al. (2010)^[161]^ | International Physical Activity Questionnaire (short) | 18–32 yrs. | 80 | NA | IPAQ determined Total MET-mins/day – Caltrac determined total energy expenditure.  Free-living activity behaviours examined using IPAQ (long) and validated with 7 days of Caltrac measurement (Unstructured). | r_s_=0.30; 95% CI = 0.07-0.49; p<0.01 | Helmorhorst (2012) |
| Schulz et al. (1989)^[101]^ | 2 week activity diary | NA | 6 | M = 4  F = 2 | 2 week activity diary determined energy expenditure – Heart rate monitoring (FLEX HR) determined energy expenditure  2 weeks of free-living physical activity behaviours (self-reported) – compared to 1 day HRM (Unstructured). | r=0.23-0.84; p<0.05 | Ainslie (2003) |
| Schmidt et al. (2003)^[162]^ | Physical activity log | 39-65 yrs. | 59 | F = 59 | PA Log determined total activity (mins.d^-1^) – CSA determined total activity (mins.d^-1^) using Freedson equation  PA Log determined moderate activity (mins.d^-1^) – CSA determined moderate activity (mins.d^-1^) using Freedson equation  PA Log determined vigorous activity (mins.d^-1^) – CSA determined vigorous activity (mins.d^-1^) using Freedson equation  PA Log determined total activity (mins.d^-1^) – CSA determined total activity (mins.d^-1^) using Hendelman equation  PA Log determined moderate activity (mins.d^-1^) – CSA determined moderate activity (mins.d^-1^) using Hendelman equation  PA Log determined vigorous activity (mins.d^-1^) – CSA determined vigorous activity (mins.d^-1^) using Hendelman equation  PA Log determined total activity (mins.d^-1^) – CSA determined total activity (mins.d^-1^) using Swartz equation  PA Log determined moderate activity (mins.d^-1^) – CSA determined moderate activity (mins.d^-1^) using Swartz equation  PA Log determined vigorous activity (mins.d^-1^) – CSA determined vigorous activity (mins.d^-1^) using Swartz equation  PA Log determined total activity (mins.d^-1^) – CSA determined total activity (counts.d^-1^)  PA Log determined moderate activity (mins.d^-1^) – CSA determined moderate activity (counts.d^-1^) equation  PA Log determined vigorous activity (mins.d^-1^) – CSA determined vigorous activity (counts.d^-1^)  Physical activity behaviours recorded for 7 days using the PA Log and the CSA activity monitor (Unstructured). | r_s_=0.24; NS  r_s_=0.25; NS  r_s_=0.16; NS  r_s_=0.15; NS  r_s_=0.23; NS  r_s_=0.17; NS  r_s_=0.24; NS  r_s_=0.28; p<0.05  r_s_=0.08; NS  r_s_=0.23; NS  r_s_=0.28; p<0.05  r_s_=0.03; NS | Prince (2008) |
| Sidney et al. (1991)^[163]^ | Physical Activity History Questionnaire | 18-30 yrs. | 5115 | M =2353  F =2762 | PAHQ determined total PA exercise units – Physical activity recall questionnaire determined total PA kcal.kg^-1^.d^-1^.  PAHQ determined moderate PA exercise units – Physical activity recall questionnaire determined moderate PA kcal.kg^-1^.d^-1^.  Physical activity from previous year (as determined by PAHQ) compared with seven day recall physical activity questionnaire (Unstructured). | r=0.95  r=0.73 | Lamonte (2001) |
| Singh et al. (2001)^[164]^ | Physical activity questionnaire | F: 50 ± 15 yrs. | 94 | F = 94 | PAQ determined total energy expenditure (MET.min^-1^.wk^-1^) – Seven day activity recall determined total energy expenditure (MET.min^-1^.wk^-1^)  PAQ determined vigorous energy expenditure (MET.min^-1^.wk^-1^) – Seven day activity recall determined vigorous energy expenditure (MET.min^-1^.wk^-1^)  PAQ determined moderate energy expenditure (MET.min^-1^.wk^-1^) – Seven day activity recall determined moderate energy expenditure (MET.min^-1^.wk^-1^)  PAQ determined usual physical activity, 7 days of activity recalls and 10 days of pedometer wear (Unstructured). | r_p_=0.65; 95%CI = 0.32-0.93  r_p_=0.85; 95%CI = 0.15-1.00  r_p_=0.44; 95%CI = 0.16-0.67 | Prince (2008) |
| Singh et al. (2001)^[164]^ | Physical activity questionnaire | M: 48 ± 14 yrs. | 47 | M = 47 | PAQ determined total energy expenditure (MET.min^-1^.wk^-1^) – Seven day activity recall determined total energy expenditure (MET.min^-1^.wk^-1^)  PAQ determined vigorous energy expenditure (MET.min^-1^.wk^-1^) – Seven day activity recall determined vigorous energy expenditure (MET.min^-1^.wk^-1^)  PAQ determined moderate energy expenditure (MET.min^-1^.wk^-1^) – Seven day activity recall determined moderate energy expenditure (MET.min^-1^.wk^-1^)  PAQ determined usual physical activity, 7 days of activity recalls and 10 days of pedometer wear (Unstructured). | r_p_=0.51; 95%CI = 0.19-0.77  r_p_=0.13; 95%CI = -0.29-0.64  r_p_=0.53; 95%CI = 0.10-0.85 | Prince (2008) |
| Sirard et al. (2000)^[165]^ | Physical Activity Diary | 25.0 ± 3.6 yrs. | 19 | M = 9  F = 10 | PAD determined minutes spent in light physical activity – CSA determined light physical activity (Freedson cut-points)  PAD determined minutes spent in moderate physical activity – CSA determined moderate physical activity (Freedson cut-points)  PAD determined minutes spent in hard physical activity – CSA determined hard physical activity (Freedson cut-points)  PAD determined minutes spent in very hard physical activity – CSA determined very hard physical activity (Freedson cut-points)  PAD determined total activity minutes– CSA determined total activity minutes (Freedson cut-points)  7 days of physical activity recall and 7 days of CSA monitoring (Unstructured). | r_p_=0.69; p<0.05 Mean%_DIFF_=-5.9%; p<0.01  r_p_=0.11; NS  r_p_=-0.13; NS; Mean%_DIFF_=81%; p<0.01  r_p_=0.10; NS; Mean%_DIFF_=84%; p<0.01  r_p_=0.48; p<0.05; Mean%_DIFF_=50%; p<0.01 | Prince (2008) |
| Smith et al. (2005)^[166]^ | Physical Activity Questionnaire (2 equation) | 18-55 yrs. | 467 | M= 130  F= 337 | PAQ determined number of MPA bouts – AAQ determined frequency of MPA bouts.  PAQ determined number of VPA bouts – AAQ determined frequency of VPA bouts.  PAQ determined number of total PA bouts – AAQ determined frequency of total PA bouts.  PAQ determined number of MPA bouts – ActiGraph determined frequency of MPA bouts (Hendelman equation)  PAQ determined number of VPA bouts – ActiGraph determined frequency of VPA bouts (Hendelman equation)  PAQ determined number of total PA bouts – ActiGraph determined frequency of total PA bouts (Hendelman equation).  AAQ measured PA for previous week, to PAQ determined PA during the usual week and 7 days of ActiGraph measurement (Unstructured). | r_s_=0.45; 95%CI = 0.34-0.55; Kappa = 31.5; 95%CI = 18.7-44.3  r_s_=0.58; 95%CI = 0.49-0.66; Kappa = 52.6; 95%CI = 38.1-67.1  r_s_=0.54; 95%CI = 0.44-0.63; Kappa = 46.7; 95%CI = 35.6-57.9  r_s_=0.38; 95%CI = 0.26-0.49; Kappa = 18.7; 95%CI = 0.8-36.6  r_s_=0.36; 95%CI = 0.24-0.47; Kappa = 11.6; 95%CI = -10.4 – 33.7  r_s_=0.39; 95%CI = 28 – 49; Kappa = 18.2; 95%CI = 3.9-32.6 | Prince (2008) |
| Smith et al. (2005)^[166]^ | Physical Activity Questionnaire (3 equation) | 18-55 yrs. | 467 | M= 130  F= 337 | PAQ determined number of MPA bouts – AAQ determined frequency of MPA bouts.  PAQ determined number of VPA bouts – AAQ determined frequency of VPA bouts.  PAQ determined number of total PA bouts – AAQ determined frequency of total PA bouts.  PAQ determined number of MPA bouts ActiGraph determined frequency of MPA bouts (Hendelman equation)  PAQ determined number of VPA bouts – ActiGraph determined frequency of VPA bouts (Hendelman equation)  PAQ determined number of total PA bouts – ActiGraph determined frequency of total PA bouts (Hendelman equation)  AAQ measured PA for previous week, to PAQ determined PA during the usual week and 7 days of ActiGraph measurement (Unstructured). | r_s_=0.33; 95%CI = 0.21-0.44; Kappa = 31.2; 95%CI = 18.9-43.5  r_s_=0.56; 95%CI = 0.47-0.64; Kappa = 53.5; 95%CI = 38.0-69.0  r_s_=0.43; 95%CI = 0.32-0.53; Kappa = 38.7; 95%CI = 26.4-51.1  r_s_=0.20; 95%CI = 0.07-0.33; Kappa = 8.3; 95%CI = -5.2-21.9  r_s_=0.27; 95%CI = 0.14-0.39; Kappa = 14.8; 95%CI = -8.1-37.8  r_s_=0.31; 95%CI = 0.18-0.43; Kappa = 24.3; 95%CI = 11.6-36.9 | Prince (2008) |
| Sobngwi et al. (2001)^[167]^ | Sub-Saharan Africa Activity Questionnaire | 19-68 yrs. | 89 | M = 44  F = 45 | SSAAQ determined mean total METs/wk – Caltrac determined total METs/wk (Females Only).  SSAAQ determined mean total METs/wk – Caltrac determined total METs/wk (Males Only).  SSAAQ examined past 12 months activity levels with 24 hours activity monitoring (Unstructured). | r=0.74; p<0.01  r=0.60; p<0.01 | Helmorhorst (2012) |
| Stel et al. (2004)^[168]^ | LASA physical activity questionnaire (LAPAQ) (interview administered) | >65 yrs. | 419 | M = 191  F = 228 | LAPAQ determined total activity scores – 7 Day activity diary determined total activity scores.  LAPAQ determined walking – 7 Day activity diary determined walking.  LAPAQ determined Bicycling – 7 Day activity diary determined bicycling.  LAPAQ determined gardening – 7 Day activity diary determined gardening.  LAPAQ determined light household chores – 7 Day activity diary determined light household chores.  LAPAQ determined heavy household chores – 7 Day activity diary determined heavy household chores.  LAPAQ determined sport – 7 Day activity diary determined sport  LAPAQ determined frequency and duration of physical activity during previous 2 weeks compared to 7 day activity diary (Unstructured). | r_s_=0.68; p<0.001  r_s_=0.50; p<0.001  r_s_=0.84; p<0.001  r_s_=0.78; p<0.001  r_s_=0.66; p<0.001  r_s_=0.63; p<0.001  r_s_=0.71; p<0.001 | Kowalski (2012) |
| Strath et al. (2004)^[169]^ | College Alumnus Questionnaire | 30.0±10.5 yrs. | 25 | M = 12  F = 13 | CAQ physical activity index determined MET-min/week – ActiGraph + HRM determined MET-min/week  Free-living activity behaviours examined using CAQ physical activity index and validated with 7 days of ActiGraph and HRM measurement (Unstructured). | r_s_=0.35 | Helmorhorst (2012) |
| Strath et al. (2003)^[170]^ | Behavioral Risk Factor Surveillance System | 30.0 ± 10.5 | 25 | M = 12  F = 13 | BRFSS determined moderate PA (mins.d^-1^) – Heart rate monitor determined moderate intensity mins.d^-1^.  BRFSS determined vigorous PA (mins.d^-1^) – Heart rate monitor determined vigorous intensity mins.d^-1^.  BRFSS determined moderate and vigorous PA (mins.d^-1^) – Heart rate monitor determined moderate and vigorous intensity mins.d^-1^.  7 days of HRM with BRFSS assessment conducted once HRM was completed (Unstructured). | r_s_=-0.10; NS  r_s_=0.54; p<0.01  r_s_=0.10; NS | Prince (2008) |
| Suzuki et al. (1998)^[171]^ | Self-administered PAQ | 18-64 yrs. | 81 | M = 49  F = 32 | PAQ determined daily energy expenditure – Calorie Counter determined daily energy expenditure (Males only).  PAQ determined daily energy expenditure – Calorie Counter determined daily energy expenditure (Females only).  PAQ determined weekly physical activity – Calorie Counter determined weekly physical activity (Males only).  PAQ determined weekly physical activity – Calorie Counter determined weekly physical activity (Females only).  7 consecutive days of measurement of calorie counter with previous 7 days of PA from questionnaire (Unstructured). | Mean_DIFF_= -103 ± 246 kcal.d^-1^; %_DIFF_=-4.5%; p<0.01  Mean_DIFF_= -53 ± 188 kcal.d^-1^; %_DIFF_=-2.9%; NS  Mean_DIFF_= 261 ± 936 kcal.d^-1^; %_DIFF_=12.2%; NS  Mean_DIFF_= 421 ± 1368 kcal.d^-1^;  %_DIFF_=23.7%; NS | Shephard (2003) |
| Tehard et al. (2005)^[172]^ | Baecke Questionnaire | 20-50 yrs. | 757 (obese) | M = 191  F = 566 | Self-reported total activity from Baecke (PA score) – Self-reported total activity from 7-d short form IPAQ (MET mins/wk^-1^).  Self-reported walking from Baecke (walking score) – Self-reported total walking from 7-d short form IPAQ (mins/wk). | r_s_=0.51; p<0.001  r_s_=0.41; p<0.001 | Andrew (2010) |
| Theou et al. (2012)^[173]^ | Minesota Leisure Time Physical Activity Survey | 63-90 yrs. | 50 | F = 50 | MLTPAS total time spent across all activities – ActiTrainer determined time engaged in non-sedentary activities.  MLTPAS total time spent across all activities - HRM determined time engaged in non-sedentary activities.  10 hours of free-living activity behaviours (ActiTrainer and HRM) and 14 days of self-reported PA (MLTPAQ). | r_p_=0.53; p<0.01  r_p_=0.20 | Gorman (2014) |
| Thuy et al. (2010)^[174]^ | Global Physical Activity Questionnaire | 25-64 yrs. | 239 | M = 121  F = 118 | GPAQ determined total physical activity (MET-hrs/week) – IPAQ determined total physical activity (MET-hrs/week).  Free-living activity behaviours examined for 7 consecutive days (Unstructured). | r_s_= -0.01-0.41 | Kim (2013) |
| Thuy et al. (2010)^[174]^ | Global Physical Activity Questionnaire | 25-64 yrs. | 245 | M = 121  F = 124 | GPAQ determined total physical activity (MET-hrs/week) – PA log determined total physical activity (MET-hrs/week).  Free-living activity behaviours examined for 7 consecutive days (Unstructured). | r_s_=-0.03-0.51 | Kim (2013) |
| Thuy et al. (2010)^[174]^ | International Physical Activity Questionnaire | 25-64 yrs. | 242 | M = 121  F = 121 | IPAQ determined total physical activity (MET-hrs/week) – GPAQ determined total physical activity (MET-hrs/week).  Free-living activity behaviours examined for 7 consecutive days (Unstructured). | r_s_= 0.21-0.34 | Kim (2013) |
| Thuy et al. (2010)^[174]^ | International Physical Activity Questionnaire | 25-64 yrs. | 241 | M = 119  F = 122 | IPAQ determined total physical activity (MET-hrs/week) – PA log determined total physical activity (MET-hrs/week).  Free-living activity behaviours examined for 7 consecutive days (Unstructured). | r_s_=0.14-0.53 | Kim (2013) |
| Timperio et al. (2003)^[175]^ | 1 Week Physical Activity Recall Questionnaire | M =37.8 ±12.7 yrs.  F =39.6 ±17.0 yrs. | 118 | M = 56  F = 62 | 1WPARQ determined total mins/d – ActiGraph determined total mins/d (Salmon Thresholds) (Men Only).  1WPARQ determined total mins/d – ActiGraph determined total mins/d (Salmon Thresholds) (Women Only).  7 days of free living physical activity examined using both 1WPARQ and activity monitor (Unstructured). | r_s_=0.29; p<0.05  r_s_=0.25; p<0.05 | Helmorhorst (2012) |
| Timperio et al. (2004)^[176]^ | Active Australia Questionnaire | 18-75 yrs. | 551 | M = 241  F = 310 | AAQ determined moderate physical activity (mins/day) – Actigraph MTI determined moderate physical activity (mins/day) (Freedson equations) (with activity log also used)  AAQ determined moderate physical activity (mins/day) – Actigraph MTI determined moderate physical activity (mins/day) (Freedson equations) (without activity log used)  AAQ determined vigorous physical activity (mins/day) – Actigraph MTI determined vigorous physical activity (mins/day) (Freedson equations) (with activity log also used).  AAQ determined vigorous physical activity (mins/day) – Actigraph MTI determined vigorous physical activity (mins/day) (Freedson equations) (without activity log used)  Free-living activity behaviours examined using AAS (previous 7 days) and validated with a minimum of 7 days of ActiGraph (MTI) measurement (Unstructured). | r_s_=0.28; 95% CI = 0.014-0.41; p<0.001 Mean_DIFF_= 5.9 (43.9) mins/day.  r_s_=0.27; 95% CI = 0.03-0.45; p<0.001 Mean_DIFF_= -0.6 (57.5) mins/day.  r_s_=0.27; 95% CI = 0.13-0.40; p<0.001 Mean_DIFF_= 7.3 (27.6) mins/day.  r_s_=0.47; 95% CI = 0.30-0.61; p<0.001 Mean_DIFF_= 7.8 (18.3) mins/day. | Kim (2013) |
| Timperio et al. (2004)^[176]^ | International Physical Activity Questionnaire (short form) | 18-75 yrs. | 551 | M = 241  F = 310 | IPAQ-s determined moderate physical activity (mins/day) – Actigraph MTI determined moderate physical activity (mins/day) (Freedson equations) (with activity log also used)  IPAQ-s determined moderate physical activity (mins/day) – Actigraph MTI determined moderate physical activity (mins/day) (Freedson equations) (without activity log used)  IPAQ-s determined vigorous physical activity (mins/day) – Actigraph MTI determined vigorous physical activity (mins/day) (Freedson equations) (with activity log also used).  IPAQ-s determined vigorous physical activity (mins/day) – Actigraph MTI determined vigorous physical activity (mins/day) (Freedson equations) (without activity log used)  Free-living activity behaviours examined using IPAQ-s (previous 7 days) and validated with a minimum of 7 days of ActiGraph (MTI) measurement (Unstructured). | r_s_=0.13; 95% CI = -0.01-0.27; p<0.05 Mean_DIFF_= 25.6 (68.4) mins/day.  r_s_=0.27; 95% CI = 0.07-0.45; p<0.001 Mean_DIFF_= 27.0 (70.9) mins/day.  r_s_=0.15; 95% CI = -0.01-0.28; p<0.05 Mean_DIFF_= 17.3 (36.8) mins/day.  r_s_=0.28; 95% CI = 0.08-0.40; p<0.001 Mean_DIFF_= 18.1 (36.7) mins/day. | Kim (2013) |
| Timperio et al. (2004)^[176]^ | International Physical Activity Questionnaire (long form) | 18-75 yrs. | 551 | M = 241  F = 310 | IPAQ-L determined moderate physical activity (mins/day) – Actigraph MTI determined moderate physical activity (mins/day) (Freedson equations) (with activity log also used)  IPAQ-L determined moderate physical activity (mins/day) – Actigraph MTI determined moderate physical activity (mins/day) (Freedson equations) (without activity log used)  IPAQ-L determined vigorous physical activity (mins/day) – Actigraph MTI determined vigorous physical activity (mins/day) (Freedson equations) (with activity log also used).  IPAQ-L determined vigorous physical activity (mins/day) – Actigraph MTI determined vigorous physical activity (mins/day) (Freedson equations) (without activity log used)  Free-living activity behaviours examined using IPAQ-l (previous 7 days) and validated with a minimum of 7 days of ActiGraph (MTI) measurement (Unstructured). | r_s_=0.27; 95% CI = 0.12-0.41; p<0.001 Mean_DIFF_= 52.0 (98.6) mins/day.  r_s_=0.17; 95% CI = 0.03-0.35; Mean_DIFF_= 66.3 (101.2) mins/day.  r_s_=0.33; 95% CI = 0.18-0.46; p<0.001 Mean_DIFF_= 15.3 (35.3) mins/day.  r_s_=0.46; 95% CI = 0.29-0.60; p<0.001 Mean_DIFF_= 26.0 (59.0) mins/day. | Kim (2013) |
| Timperio et al. (2004)^[176]^ | Behavioral Risk Factor Surveillance System (BRFSS) | 18-75 yrs. | 551 | M = 241  F = 310 | BRFSS determined moderate physical activity (mins/day) – Actigraph MTI determined moderate physical activity (mins/day) (Freedson equations) (with activity log also used)  BRFSS determined moderate physical activity (mins/day) – Actigraph MTI determined moderate physical activity (mins/day) (Freedson equations) (without activity log used)  BRFSS determined vigorous physical activity (mins/day) – Actigraph MTI determined vigorous physical activity (mins/day) (Freedson equations) (with activity log also used).  BRFSS determined vigorous physical activity (mins/day) – Actigraph MTI determined vigorous physical activity (mins/day) (Freedson equations) (without activity log used)  Free-living activity behaviours examined using BRFSS (previous 7 days) and validated with a minimum of 7 days of ActiGraph (MTI) measurement (Unstructured). | r_s_=0.23; 95% CI = 0.08-0.37; p<0.01 Mean_DIFF_= 6.2 (71.7) mins/day.  r_s_=0.32; 95% CI = 0.12-0.49; p<0.001 Mean_DIFF_= 32.1 (339.1) mins/day.  r_s_=0.22; 95% CI = 0.01-0.40; p<0.01 Mean_DIFF_= 10.1 (53.6) mins/day.  r_s_=0.26; 95% CI = 0.06-0.44; p<0.01 Mean_DIFF_= 12.5 (31.4) mins/day. | Kim (2013) |
| Tomioka et al (2011)^[177]^ | International Physical Activity Questionnaire | 65-89 yrs. | 325 | M = 164  F = 161 | IPAQ determined MET-min/week – Lifecorder determined MET-min/week (Men)  IPAQ determined MET-min/week – Lifecorder determined MET-min/week (Women)  Free-living activity behaviours examined for 14 days using both IPAQ and Kenz Lifecorder (Unstructured). | r_s_=0.42-0.53; p<0.01  r_s_=0.49; p<0.01 | Helmorhorst (2012) |
| Vandelanotte et al. (2005)^[180]^ | International Physical Activity Questionnaire (computerized | 30.9±11.0 yrs. | 53 | M = 23  F = 30 | IPAQ determined Total PA (kcal) – Actigraph determined kcals (kcals/week; Freedson equations)  IPAQ determined MVPA (kcal) – Actigraph determined kcals (kcals/week; Freedson equations)  IPAQ determined VPA (kcal) – Actigraph determined kcals (kcals/week; Freedson equations)  Free-living activity behaviours examined using IPAQ (previous 7 days) and validated with a minimum of 7 days of ActiGraph (CSA) measurement (Unstructured). | r_s_=0.43; p<0.01  r_s_=0.42; p<0.01  r_s_=0.45; p<0.01 | Kim (2013) |
| Vandelanotte et al. (2005)^[180]^ | International Physical Activity Questionnaire (computerized | 30.9±11.0 yrs. | 53 | M = 23  F = 30 | IPAQ determined Total PA (mins) – Physical Activity Diary determined Total PA (mins/week)  IPAQ determined Total PA (kcal) – Physical Activity Diary determined kcals (kcals/week)  IPAQ determined MVPA (mins) – Physical Activity Diary determined Total PA (mins/week)  IPAQ determined MVPA (kcal) – Physical Activity Diary determined kcals (kcals/week)  IPAQ determined VPA (mins) – Physical Activity Diary determined Total PA (mins/week)  IPAQ determined VPA (kcal) – Physical Activity Diary determined kcals (kcals/week)  Free-living activity behaviours examined using IPAQ (previous 7 days) and validated with a 7 day activity diary (Unstructured). | r_s_=0.39; p<0.01  r_s_=0.46; p<0.01  r_s_=0.45; p<0.01  r_s_=0.46; p<0.01  r_s_=0.79; p<0.01  r_s_=0.79; p<0.01 | Kim (2013) |
| Van der Pleog et al. (2010)^[178]^ | Time Use Diary | 18-63 yrs. | 129 | NA | TUD determined min/day LIPA (non-occupational) – Accelerometer determined min/day LIPA (non-occupational) (Matthews cut point).  TUD determined min/day MVPA (non-occupational) – Accelerometer determined min/day MVPA (non-occupational) (Matthews cut point).  TUD determined min/day LIPA – Accelerometer determined min/day LIPA (Matthews cut point).  TUD determined min/day MVPA – Accelerometer determined min/day MVPA (Matthews cut point).  TUD determined min/day LIPA (Leisure Activity) – Accelerometer determined min/day LIPA (Leisure Activity) (Matthews cut point).  TUD determined min/day MVPA (Leisure Activity) – Accelerometer determined min/day MVPA (Leisure Activity) (Matthews cut point).  TUD determined min/day MVPA (Transportation) – Accelerometer determined min/day MVPA (Transportation) (Matthews cut point).  10 days of free-living activity behaviours, with 2 days of time use diary conducted twice (Day 1-2 and Day 9-10). | r_s_=0.27-0.39; p<0.05  r_s_=0.45-0.69; p<0.05  r_s_=0.17-0.24  r_s_=0.20-0.34; p<0.05  r_s_=-0.02-0.09  r_s_=0.61-0.70; p<0.05  r_s_=0.63-0.72; p<0.05 | Foley (2012) |
| Van der Ploeg^[179]^ | International Physical Activity Questionnaire | 18-65 yrs. | 728 | M = 341  F = 387 | IPAQ determined walking (mins) – ActiGraph determined MVPA mins/week  IPAQ determined walking and moderate physical activity (mins) – ActiGraph determined MVPA mins/week.  Free-living activity behaviours examined using IPAQ and validated with a minimum of 5 days of ActiGraph (CSA) measurement (Unstructured). | r_s_=0.18-0.39  r_s_=0.04-0.24 | Kim (2013) |
| Washburn et al. (1990)^[181]^ | Behavioural Risk Factor Surveillance Survey | >65 yrs. | 103 | M = 52  F = 51 | BRFSS determined total activity minutes per day – 3 Day physical activity diary determined total minutes per day.  BRFSS determined activity behaviours over the last month compared to 3DPAD over the previous 3 days (Unstructured). | Mean_Error_=-72.5 (201.7) minutes  Absolute_Error_= 168.6 (131.4) minutes | Kowalski (2011) |
| Welk et al. (2001)^[182]^ | 7 day physical activity recall | 38-57 yrs. | 33 | M = 20  F = 13 | 7DPAR determined energy expenditure (PAR 1) (kcal.d^-1^) – Tritrac determined energy expenditure (Using BMR) (kcal.d^-1^)  7DPAR determined energy expenditure (PAR 1) (kcal.d^-1^) – Tritrac determined energy expenditure (Using MET based approach) (kcal.d^-1^)  7DPAR determined energy expenditure (PAR 1) (kcal.d^-1^) – Tritrac determined energy expenditure (Using Nichol equation) (kcal.d^-1^)  7DPAR determined energy expenditure (PAR 2) (kcal.d^-1^) – Tritrac determined energy expenditure (Using BMR) (kcal.d^-1^)  7DPAR determined energy expenditure (PAR 2) (kcal.d^-1^) – Tritrac determined energy expenditure (Using MET based approach) (kcal.d^-1^)  7DPAR determined energy expenditure (PAR 2) (kcal.d^-1^) – Tritrac determined energy expenditure (Using Nichol equation) (kcal.d^-1^)  7 day physical activity recall and 8 days of Tritrac measurement (Unstructured). | r=0.72; p<0.01;  r=0.84; p<0.01  r=0.84; p<0.01  r=0.72; p<0.01  r=0.93; p<0.01  r=0.95; p<0.01 | Prince (2008) |
| Wendel-Vos et al. (2003)^[183]^ | Short Questionnaire to Assess Health enhancing  physical activity | 27 – 58 yrs. | 50 | M = 36  F = 14 | SQUASH determined physical activity levels – CSA determined physical activity (Freedson cut points)  7 days off free-living physical activity determined from CSA compared to SQUASH determined physical activity in the average week over the past month (Unstructured). | r_s_=0.45; p< 0.01; 95%CI = 0.17-0.66 | Prince (2008) |
| Wickel et al. (2006)^[184]^ | Bouchard Activity Diary | 18-23 yrs. | 70 | M = 13  F = 57 | BAD determined resting energy expenditure (kcal.kg^-1^.d^-1^) – Tritrac determined resting energy expenditure (kcal.kg^-1^.d^-1^)  BAD determined activity energy expenditure (kcal.kg^-1^.d^-1^) – Tritrac determined activity energy expenditure (kcal.kg^-1^.d^-1^)  BAD determined total energy expenditure (kcal.kg^-1^.d^-1^) – Tritrac determined total energy expenditure (kcal.kg^-1^.d^-1^)  Bouchard activity Diary and Tritrac-R3D recordings completed for 24 hours during weekdays (Unstructured). | r=0.92; p<0.01  Mean_DIFF_= 69 kcal.d^-1^.  r=0.72; p<0.01; Mean_DIFF_= 576 kcal.d^-1^.  r=0.86; p<0.01; Mean_DIFF_= 645 ± 494 kcal.d^-1^; p<0.05 | McClain (2009) |
| Wilbur et al. (2001)^[185]^ | Physical Activity Exercise log | 45-65 yrs. | 156 | F = 156 | PAEL determined walking periods – Heart rate monitor determined walking periods.  PAEL determined walking duration (mins.walk^-1^) – Heart rate monitor determined walking duration (mins.walk^-1^).  24 weeks of monitoring walking periods as part of an exercise intervention, whereby both HRM and Exercise log information was recorded during each walking period (Unstructured). | r=0.962; Mean_DIFF_=4.33 ± 7.09 walks  r=0.536; Mean_DIFF_=5.0 ± 8.08 mins | Prince (2008) |
| Yore et al. (2007)^[186]^ | Behavioral Risk Factor Surveillance System | 44.5±15.7 yrs. | 60 | M = 30  F = 30 | BRFSS determined VPA mins/wk – ActiGraph determined VPA mins/wk.  BRFSS determined MPA mins/wk – ActiGraph determined MPA mins/wk.  7 days of free living physical activity examined using both BRFSS and activity monitor (Unstructured). | r=0.52-0.63  r=0.16-0.27 | Helmorhorst (2012) |
| Yore et al. (2007)^[186]^ | Behavioral Risk Factor Surveillance System | 44.5±15.7 yrs. | 60 | M = 30  F = 30 | BRFSS determined VPA mins/wk – Physical Activity log determined VPA mins/wk.  BRFSS determined MPA mins/wk – Physical Activity log determined MPA mins/wk.  7 days of free living physical activity examined using both BRFSS and activity monitor (Unstructured). | r=0.60-0.66  r=0.04-0.21 | Helmorhorst (2012) |
| Yore et al. (2007)^[186]^ | Behavioral Risk Factor Surveillance System | 44.5±15.7 yrs. | 60 | M = 30  F = 30 | BRFSS determined MPA mins/wk – ActiGraph determined MPA mins/wk (Troiano equations).  7 days of free living physical activity examined using both BRFSS and activity monitor (Unstructured). | r=0.03-0.28 | Helmorhorst (2012) |

**PA = Physical Activity; MPA = Moderate Physical Activity; VPA = Vigorous Physical Activity; LIPA = Light Intensity Physical Activity; MVPA = Moderate-to-Vigorous Physical Activity; PAEE = Physical Activity Energy Expenditure; MJ = Megajoules; MET = Metabolic Equivalent; PAL = Physical Activity Level; HR = Heart Rate; HRM = Heart Rate Monitor; DLW = Doubly Labelled Water; NS = Non-significant; MeanDIFF = Mean Difference; rs= Spearman Correlation; rp = Pearson Correlation; r = Correlation (not specified); %Agr = Percentage Agreement; SE = Standard Error; SD = Standard Deviation; M = Male; F = Female.**

**24PARQ = 24 hour physical activity recall questionnaire; CAQ = College Alumnus Questionnaire; TOAQ = Tecumseh Occupational Activity Questionnaire; 7DPARQ = 7 day physical activity recall questionnaire; 3MPARQ = 3 Month Physical Activity Recall Questionnaire; WSRAA = Weekly self-report of aerobic activity; RPAQ = Recent Physical Activity Questionnaire; NZPAQ = NZPAQ = New Zealand Physical Activity Questionnaire; IPAQ = International Physical Activity Questionnaire; IPAQ-L = International Physical Activity Questionnaire Long Form; IPAQ-S= International Physical Activity Questionnaire Short Form; AAPAS = Active Australia Physical Activity Survey; GPAS = Global Physical Activity Questionnaire; SPAQ = Scottish Physical Activity Questionnaire; BWHSPAQ = Black Women’s Health Study Self-Administered Physical Activity Questionnaire; FCPQ = Five City Projects Questionnaire; SAPAQ = Self -Administered Physical Activity Questionnaire; PAQ = Physical Activity Questionnaire; NCHRBS = National College Health Risk Behaviour Survey; AWAS = Australian Women’s Activity Survey; PYTPAQ = Past Year Total Physical Activity Questionnaire; PAD = Physical Activity Diary; CHAMPS = Community Healthy Activities Model Program for Seniors; LNZNS = Life in New Zealand National Survey; PAS = Physical Activity Scale;EEQ = Energy Expenditure Questionnaire; S7DPAR = Stanford 7 day physical activity recall; OIMQ = Office In Motion Questionnaire; HAQ = Harvard Alumni Questionnaire; WOTM7DAD = Women on the Move 7 day Activity Diary; 3DPAL = 3 Day Physical Activity Log; SWHSPAQ = Shanghai Women’s Health Study Physical; PDPAR = Previous Day Recall Questionnaire; STAR-Q = Short Telephone Administers Recall Questionnaire; PAAT = Physical Activity Assessment Tool; SPSPPAQ = Singapore Prospective Study Program Physical Activity Questionnaire; YPAS = Yale Physical Activity Survey; PAHQ = Physical Activity History Questionnaire; AAQ = Active Australia Questionnaire; SSAAQ = Sub-Saharan Africa Activity Questionnaire; LAPAQ = LASA physical activity questionnaire; BRFSS = Behavioral Risk Factor Surveillance System; 1WPARQ = 1 Week Physical Activity Recall Questionnaire; TUD = Time Use Diary; SQUASH = Short Questionnaire to Assess Health Enhancing Physical Activity; BAD = Bouchard Activity Diary; PAEL = Physical Activity Exercise Log; CSA = Computer Science Application accelerometer; SAM = Solar Activity Monitor;**

**Supplementary Table 3.** Intra-instrument Reliability of self-reported measures of physical activity.

| **Author** | **Measure** | **Age Range** | **Sample Size** | **Sex** | **Details** | **Results** | **Primary Source** |
| --- | --- | --- | --- | --- | --- | --- | --- |
| Booth et al. (1996)^[195]^ | Self-reported Physical Activity | 18-79 yrs. | 116 | M = 55  F = 61 | Self-report measures administered twice, 3 days apart to assess the same previous 2 week period.  Total Activity  Vigorous Activity  Moderate Activity  Walking  Energy Expenditure | ICC=0.90; 95%CI = 0.86-0.93; 95%LoA = 7.8  ICC=0.90; 95%CI = 0.86-0.93; 95%LoA = 4.7  ICC=0.77; 95%CI = 0.69-0.84; 95%LoA = 4.0  ICC=0.89; 95%CI = 0.85-0.93; 95%LoA = 6.0  ICC=0.86; 95%CI = 0.80-0.90; 95%LoA = 4.4 | Pennathur (2003) |

ICC = Intraclass Correlation Coefficient; CI = Confidence Intervals; LoA = Limits of Agreeement

**Supplementary Table 4a.** Test-retest reliability of self-reported measures of physical activity/energy expenditure within a duration of less than or equal to one week.

| **Author** | **Measure** | **Age Range** | **Sample Size** | **Sex** | **Details** | **Results** | **Primary Source** |
| --- | --- | --- | --- | --- | --- | --- | --- |
| Ainsworth et al. (1993)^[109]^ | College Alumnus Questionnaire | 38±9 yrs. | 78 | M = 28  W = 50 | CAQ PA Index test retest reliability month 1 to 8  CAQ PA Index test retest reliability month 1 to 9  CAQ PA Index test retest reliability month 8 to 9  CAQ Flights of Stairs test retest reliability month 1 to 8  CAQ Flights of Stairs test retest reliability month 1 to 9  CAQ Flights of Stairs test retest reliability month 8 to 9  CAQ City Blocks Walked test retest reliability month 1 to 8  CAQ City Blocks Walked test retest reliability month 1 to 9  CAQ City Blocks Walked test retest reliability month 8 to 9  CAQ Sport and Recreation test retest reliability month 1 to 8  CAQ Sport and Recreation test retest reliability month 1 to 9  CAQ Sport and Recreation test retest reliability month 8 to 9 | r=0.34; p<0.05  r=0.43  r=0.72; p<0.01  r=0.59; p<0.01  r=0.59; p<0.01  r=0.88; p<0.01  r=0.27; p<0.05  r=0.38; p<0.01  r=0.55; p<0.01  r=0.23  r=0.25  r=0.43; p<0.01 | Andrews (2010) |
| Brown et al. (2004)^[203]^ | Active Australia | 18-75 yrs. | 356 | M = 161  F = 195 | Walking Items  Moderate activities  Vigorous activities  Total activities  Questionnaires repeated 1 day after initial assessment (telephone interviews). | ICC=0.56  ICC=0.16  ICC=0.64  ICC=0.64 | Andrew (2010) |
| Brown et al. (2004)^[203]^ | IPAQ Short Form | 18-75 yrs. | 104 | M = 43  F = 61 | Walking Items  Moderate activities  Vigorous activities  Total activities (MET min wk^-1^)  Questionnaires repeated 1 day after initial assessment (telephone interviews). | ICC=0.53  ICC=0.41  ICC=0.52  ICC=0.68 | Andrew (2010) |
| Brown et al. (2004)^[203]^ | Behavioral Risk Factor  Surveillance System (BRFSS) | 18-75 yrs. | 127 | M = 62  F = 65 | Walking Items  Moderate activities  Vigorous activities  Total activities  Questionnaires repeated 1 day after initial assessment (telephone interviews). | ICC=0.45  ICC=0.44  ICC=0.39  ICC=0.59 | Andrew (2010) |
| Brown et al. (2004)^[203]^ | Australian National Health Survey | 18-75 yrs. | 122 | M = 54  F = 68 | Walking Items  Moderate activities  Vigorous activities  Total activities (MET min wk^-1^)  Questionnaires repeated 1 day after initial assessment (telephone interviews). | ICC=0.78  ICC=0.21  ICC=0.22  ICC=0.57 | Andrew (2010) |
| Bull et al. (2009)^[116]^ | Global Physical Activity Questionnaire | 18-75 yrs. | 2122 | M = 1301  F = 821 | Leisure total minutes PA examined 3-7 days after initial examination  Occupational total minutes PA examined 3-7 days after initial examination  Transportation total minutes PA examined 3-7 days after initial examination. | r_s_=0.78; p<0.01  r_s_=0.77; p<0.01  r_s_=0.81; p<0.01 | Helmorhorst (2012) |
| Chasan Taber et al. (2004)^[205]^ | Pregnancy Physical Activity Questionnaire | 16-40 yrs. | 235 | F = 235 | Test retest reliability (Total activity) examined after 7 days from initial administration. | ICC=0.78 ; p<0.001 | Helmorhorst (2012) |
| Colbert et al. (2011)^[76]^ | The Community Healthy Activities Model Program for Seniors Questionnaire (CHAMPS) | 74.7 ± 6.5 yrs. | 56 | M = 12  F = 44 | Questionnaire administered at baseline and 1 week after initial assessment.  Log-transformed CHAMPS determined kilocalories per day | ICC= 0.64  Mean_BIAS_= -11 ± 181 kcal.d^-1^. | Plasqui (2013) |
| Colbert et al. (2011)^[76]^ | Yale Physical Activity Survey | 74.7 ± 6.5 yrs. | 56 | M = 12  F = 44 | Questionnaire administered at baseline and 1 week after initial assessment.  YPAS determined kilocalories per day | ICC= 0.60  Mean_BIAS_= 76 ± 354 kcal.d^-1^. | Plasqui (2013) |
| Colbert et al. (2011)^[76]^ | Physical Activity Scale for the Elderly | 74.7 ± 6.5 yrs. | 56 | M = 12  F = 44 | Questionnaire administered at baseline and 1 week after initial assessment.  PASE determined kilocalories per day | ICC= 0.73  Mean_BIAS_= -78 ± 501 kcal.d^-1^. | Plasqui (2013) |
| Craig et al. (2003)^[121]^ | IPAQ Long Form Last 7 Days | 18-65 yrs. | 200 | NA | Total Physical activity (All).  Telephone Administered at day 0 and up to 1 week later. | r_s_=0.79 | Andrew (2010) |
| Craig et al. (2003)^[121]^ | IPAQ Long Form Last 7 Days | 18-65 yrs. | 294 | NA | Total Physical activity (All).  Self-Administered at day 0 and up to 1 week later. | r_s_=0.70-0.91 (across countries) | Andrew (2010) |
| Craig et al. (2003)^[121]^ | IPAQ Long Form Usual Week | 18-65 yrs. | 57 | NA | Total Physical activity (All).  Telephone Administered at day 0 and up to 1 week later. | r_s_=0.72-0.96 (across countries) | Andrew (2010) |
| Craig et al. (2003)^[121]^ | IPAQ Long Form Usual Week | 18-65 yrs. | 57 | NA | Total Physical activity (All).  Interview Administered at day 0 and up to 1 week later. | r_s_=0.46-0.86 (across countries) | Andrew (2010) |
| Craig et al. (2003)^[121]^ | IPAQ Long Form Usual Week | 18-65 yrs. | 57 | NA | Total Physical activity (All).  Self- Administered at day 0 and up to 1 week later. | r_s_=0.69-0.93 (across countries) | Andrew (2010) |
| Craig et al. (2003)^[121]^ | IPAQ Short Form Last 7 Days | 18-65 yrs. | 301 | NA | Total Physical activity (All).  Telephone Administered at day 0 and up to 1 week later. | r_s_=0.66 - 0.87 | Andrew (2010) |
| Craig et al. (2003)^[121]^ | IPAQ Short Form Last 7 Days | 18-65 yrs. | 292 | NA | Total Physical activity (All).  Self-Administered at day 0 and up to 1 week later. | r_s_=0.66-0.88 (across countries) | Andrew (2010) |
| Craig et al. (2003)^[121]^ | IPAQ Short Form Usual Week | 18-65 yrs. | 57 | NA | Total Physical activity (All).  Telephone Administered at day 0 and up to 1 week later. | r_s_=0.73-0.83 (across countries) | Andrew (2010) |
| Craig et al. (2003)^[121]^ | IPAQ Short Form Usual Week | 18-65 yrs. | 148 | NA | Total Physical activity (All).  Interview Administered at day 0 and up to 1 week later. | r_s_=0.25-0.88 (across countries) | Andrew (2010) |
| Craig et al. (2003)^[121]^ | IPAQ Short Form Usual Week | 18-65 yrs. | 596 | NA | Total Physical activity (All).  Self-Administered at day 0 and up to 1 week later. | r_s_=0.32-0.84 (across countries) | Andrew (2010) |
| Craig et al. (2003)^[121]^ | IPAQ Short Form Usual Week | 18-65 yrs. | 906 | NA | Total Physical activity (All).  Self- Administered at day 0 and up to 1 week later. | r_s_=0.65-0.84 (across countries) | Andrew (2010) |
| Cyarto et al. (2006)^[210]^ | CHAMPS physical activity questionnaire | >60 yrs. | 43 | NA | 1 week test-retest reliability of the CHAMPS questionnaire (elderly).  Total PA hours.wk  Total PA frequency.wk  Total EE MET.hrs.wk | ICC= 0.76  ICC= 0.79  ICC= 0.75 | Forsen et al. (2010) |
| Dinger (2003)^[125]^ | National College Health Risk Behaviour Survey | 24.1 ± 3.5 yrs. | 20 | M = 11  F = 9 | NCHRBS administered at baseline and 7 days after initial assessment  Number of days achieving 20 mins of Vigorous Physical Activity  Number of days achieving 30 mins of Moderate Physical Activity (2000 version)  Number of days achieving 30 mins of Moderate Physical Activity (1995 version) | ICC = 0.98; 95%CI = 0.95-0.99  ICC = 0.94; 95%CI = 0.86-0.98  ICC = 0.96; 95%CI = 0.89-0.98 | Prince (2008) |
| Dinger et al. (2006)^[126]^ | International Physical Activity Questionnaire | 20.8±1.5 yrs. | 123 | M = 32  F = 91 | IPAQ administered 4-6 days after initial administration  Vigorous Physical Activity (mins/week)  Moderate Physical Activity (mins/week)  Walking (mins/week)  Total Physical Activity (mins/week) | ICC=0.89; 95% CI =0.84-0.93  ICC=0.71; 95% CI =0.58-0.80  ICC=0.89; 95% CI =0.84-0.93  ICC=0.86; 95% CI =0.80-0.91 | Kim (2013) |
| Dinger et al. (2004)^[212]^ | Physical activity scale for the elderly (PASE) | 75.7±7.9 yrs. | 56 | M = 13  F = 43 | PASE administered after 7 days free living physical activity behaviours, and administered to examine the same 7 days three days later..  Leisure Score  Home Score  Occupation Score  Total Score | ICC=0.56  ICC=0.94  ICC=0.91  ICC=0.91 | Forsen (2010) |
| Fjeldsoe et al. (2009)^[130]^ | Australian Women’s Activity Survey | 35±5 yrs. | 40 | F = 40 | Test retest reliability examined after 7 days from initial administration.  Total Activity  Health Enhancing Physical activity | ICC=0.73; 95% CI = 0.51-0.86  ICC=0.80; 95% CI = 0.65-0.89 | Helmorhorst (2012) |
| Gauthier et al. (2009)^[220]^ | International Physical Activity Questionnaire | 20-63 yrs. | 34 | M = 14  F = 20 | IPAQ administered 1 day after initial administration  Total MET-mins/week | ICC=0.93; 95% CI =0.86-0.97; p<0.01 | Helmorhorst (2012) |
| Giles-Corti et al. (2006)^[221]^ | Neighbourhood PA Questionnaire | 20-71 yrs. | 82 | M = 27  F = 55 | NPAQ administered at baseline and 1 week after initial assessment.  Walking undertaken within the neighbourhood  Walking undertaken outside the neighbourhood  Walking for transport undertaken within the neighbourhood  Walking for transport undertaken outside the neighbourhood  Walking for transport undertaken within the neighbourhood (frequency)  Walking for transport undertaken outside the neighbourhood (frequency)  Walking for transport undertaken within the neighbourhood (duration)  Walking for transport undertaken outside the neighbourhood (duration)  Recreational walking undertaken within the neighbourhood (frequency)  Recreational walking undertaken outside the neighbourhood (frequency)  Recreational walking undertaken within the neighbourhood (duration)  Recreational walking undertaken outside the neighbourhood (duration)  Total Walking  Total MET min of Physical Activity | Kappa=0.84; %Agreement =94%  Kappa=0.73; %Agreement =87%  Kappa=0.85;  Kappa=0.66;  ICC =0.92 (95%CI= 0.66-0.90)  ICC =0.87 (95%CI= 0.70-0.95)  ICC =0.96 (95%CI= 0.70-0.90)  ICC =0.84 (95%CI= 0.68-0.92)  ICC =0.92 (95%CI= 0.85-0.95)  ICC =0.81 (95%CI= 0.68-0.92)  ICC =0.90 (95%CI= 0.80-0.94)  ICC =0.55 (95%CI= 0.00-0.82)  ICC=0.91; (95%CI= 0.84-0.94)  ICC=0.82; (95%CI= 0.73-0.89) | Van Poppel (2010) |
| Gill et al. (2008)^[222]^ | Interview administered Phone-FITT | 79.4 ± 2.9 years | 43 | M = 21  F = 22 | Frequency and Duration of Tasks  Frequency, Intensity and Duration of Tasks  Telephone Administered at day 0 and up to 1 week later. | ICC = 0.77; 95% CI = 0.63-0.87  ICC = 0.74; 95% CI = 0.58-0.85 | Kowalski (2012) |
| Hallal et al. (2010)^[134]^ | International Physical Activity Questionnaire | 40.3±15.1 yrs. | 156 | M = 75  F = 81 | IPAQ administered 5 day after initial administration  Total Score (minutes/week) | r_s_=0.90  Mean_DIFF_=3 mins; Kappa=0.78 | Helmorhorst (2012) |
| Hayden-Wade et al. (2003)^[268]^ | 7 Day Physical Activity Recall Questionnaire | 33.8 ± 11.8 yrs. | 74 | M = 27  F = 47 | 7DPARQ assessed over the telephone and 3 days later an in-person 7DPARQ administered.  Total amount of physical activity  Strength  Flexibility | ICC = 0.96  ICC = 0.97  ICC = 0.99 | Prince (2008) |
| Kolbe-Alexander et al. (2006)^[228]^ | Yale Physical Activity Survey | 62-70 yrs. | 122 | M = 52  F = 70 | Test-retest conducted a minimum of 3 days and a maximum of 5 days apart. | r_s_=0.44-0.99 | Gorman (2014) |
| Kolbe-Alexander et al. (2006)^[228]^ | IPAQ | 62-70 yrs. | 122 | M = 52  F = 70 | Test-retest conducted a minimum of 3 days and a maximum of 5 days apart. | r_s_=0.29-0.77 | Gorman (2014) |
| Kurtze et al. (2008)^[139]^ | International Physical Activity Questionnaire | 20-39 yrs. | 108 | M = 108 | IPAQ determined Vigorous Physical Activity examined 1 week apart.  IPAQ determined Moderate Physical Activity examined 1 week apart. | ICC=0.62; 95% CI = 0.47-0.73  ICC=0.30; 95% CI = 0.09-0.49 | Helmorhorst (2012) |
| Kurtze et al. (2007)^[229]^ | Nord-  Trøndelag Health Study Questionnaire 2 | 20-39 yrs. | 108 | M = 108 | Test retest reliability (HUNT 2) examined after 7 days from initial administration.  Hard Activity  Occupational Activity  Light Activity | r_s_=0.50; p<0.01  Kappa=0.41 (0.29-0.54)  r_s_=0.85; p<0.01  Kappa=0.80 (0.71-0.89)  r_s_=0.17; Kappa=0.20 (0.04-0.35) | Helmorhorst (2012) |
| Kurtze et al. (2008)^[230]^ | Nord-  Trøndelag Health Study Questionnaire 1 | 20-39 yrs. | 108 | M = 108 | Test retest reliability (HUNT 1) examined after 7 days from initial administration.  Frequency  Intensity  Duration | r_s_=0.87; p<0.01  Kappa=0.80  r_s_=0.87; p<0.01  Kappa=0.82  r_s_=0.76; p<0.01  Kappa=0.69 | Helmorhorst (2012) |
| Lowther et al. (1999)^[234]^ | Scottish Physical Activity Questionnaire | 33±12 yrs. | 34 | M = 9  F = 25 | Questionnaire administers 2 days after initial administration. | r_p_=0.998; p<0.01  Mean_DIFF_=3.09 ± 26.5 mins. | Helmorhorst (2012) |
| MacFarlane et al. (2011)^[143]^ | International Physical Activity Questionnaire | 26.2±9.9 yrs. | 83 | M = 47  F = 36 | IPAQ administered 3 days after initial administration  Total MET-mins/week | ICC=0.93; %CV= 22.8 | Helmorhorst (2012) |
| MacFarlane et al. (2007)^[144]^ | International Physical Activity Questionnaire | 15-55 yrs. | 49 | M = 30  F = 19 | IPAQ administered 3 days after initial administration  Total MET-mins/week | ICC=0.79; 95% CI= 0.66-0.88 | Helmorhorst (2012) |
| Maddison et al. (2007)^[82]^ | IPAQ | 18 – 64 yrs. | 36 | M = 16  F = 20 | Administered at Day 0, Day 8 and Day 15. | r_s1-8_=0.79; p<0.001  r_s8-15_=0.74; p<0.001 | Westerterp (2009) |
| Maddison et al. (2007)^[82]^ | New Zealand Physical Activity Questionnaire | 18 – 64 yrs. | 36 | M = 16  F = 20 | NZPAQ administered at Day 0, Day 8 and Day 15. | r_s1-8_=NR;  r_s8-15_=0.69; p<0.001 | Westerterp (2009) |
| Mader et al. (2006)^[146]^ | Swiss Health Survey | 15-75 yrs. | 178 | M = 101  F = 77 | Sweat episodes, leisure time (d/wk^-1^)  Questionnaires repeated within 14-21 days. | r_s_=0.63 | Andrew (2010) |
| Mader et al. (2006)^[146]^ | Health-enhancing  physical activity | 15-75 yrs. | 178 | M = 101  F = 77 | Dichotomous measure active/inactive  Questionnaires repeated within 14-21 days. | kappa=0.46 | Andrew (2010) |
| Mader et al. (2006)^[146]^ | IPAQ Short Form | 15-75 yrs. | 178 | M = 101  F = 77 | Moderate activities  Walking  Vigorous Activities  Total Activities  Questionnaires repeated within 14-21 days. | r_s_=0.50  r_s_=0.48  r_s_=0.43  r_s_=0.54 | Andrew (2010) |
| Mader et al. (2006)^[146]^ | Office In Motion Questionnaire | 15-75 yrs. | 178 | M = 101  F = 77 | Moderate activities  Vigorous activities  Total activities (Met min wk^-1^)  Questionnaires repeated within 14-21 days. | r_s_=0.70  r_s_=0.69  r_s_=0.68 | Andrew (2010) |
| Matthews et al. (2005)^[148]^ | Short Telephone Administered Recall Questionnaire | 46 yrs. | 104 | M = 45  F = 59 | Questionnaire administered at baseline and 3 days after initial administration.  MPA frequency from open questions  MPA recommendations from open questions  VPA frequency from open questions  VPA recommendations from open questions  MPA frequency from closed questions  MPA recommendations from closed questions  VPA frequency from closed questions  VPA recommendations closed open questions | Kappa=0.32; 95%CI = 0.06-0.59  Kappa=0.52; 95%CI = 0.27-0.77  Kappa=0.68; 95%CI = 0.51-0.86  Kappa=0.81; 95%CI = 0.63-0.99  Kappa=0.53; 95%CI = 0.33-0.74  Kappa=0.46; 95%CI = 0.19-0.73  Kappa=0.73; 95%CI = 0.58-0.89  Kappa=0.73; 95%CI = 0.54-0.92 | Matthews (2005) |
| Meriwether et al. (2006)^[152]^ | Physical Activity Assessment Tool | 18-64 yrs. | 68 | M = 10  F = 58 | Questionnaire administers 7 days after initial administration.  Total PA  MPA  VPA | r_s_=0.618; p<0.01  r_s_=0.49; p<0.01  r_s_=0.77; p<0.01 | Helmorhorst (2012) |
| Meriwether et al. (2006)^[152]^ | IPAQ Long Form | 18-64 yrs. | 68 | M = 10  F = 58 | Questionnaire administers 7 days after initial administration. | r_s_=0.627; p<0.01 | Helmorhorst (2012) |
| Roman Vinas et al. (2010)^[160]^ | International Physical Activity Questionnaire | 20-68 yrs. | 75 | M = 42  F = 33 | IPAQ administered 3-4 days after initial administration  Total Physical Activity (MET-mins-day)  Vigorous Physical Activity (mins/week)  Moderate Physical Activity (mins/week)  Walking (mins/week) | r_s_=0.82; p<0.01  r_s_=0.79; p<0.01  r_p_=0.83; p<0.01  r_p_=0.73; p<0.01 | Kim (2013) |
| Smith et al. (2005)^[166]^ | Physical Activity Questionnaire | 18-55 yrs. | 237 | M = 66  F = 171 | 2 equation Questionnaire administered at baseline and 3 days after initial assessment.  Moderate activity walking  Vigorous activity  Total activity | r_s_=0.42; 95%CI = 0.30-0.52; Kappa = 43.1; 95%CI = 28.7-57.5  r_s_=0.65; 95%CI = 0.57-0.72; Kappa = 58.9; 95%CI = 46.4-71.4  r_s_=0.61; 95%CI = 0.53-0.69; Kappa = 58.0; 95%CI = 47.2-68.8 | Prince (2008) |
| Smith et al. (2005)^[166]^ | Physical Activity Questionnaire | 18-55 yrs. | 230 | M = 64  F = 166 | 3 equations Questionnaire administered at baseline and 3 days after initial assessment.  Moderate activity walking  Vigorous activity  Total activity | r_s_=0.57; 95%CI = 0.47-0.66; Kappa = 47.2; 95%CI = 35.1-59.3  r_s_=0.68; 95%CI = 0.60-0.75; Kappa = 61.2; 95%CI = 47.2-75.1  r_s_=0.63; 95%CI = 0.53-0.70; Kappa = 55.6; 95%CI = 43.8-67.4 | Prince (2008) |
| Timperio et al. (2003)^[175]^ | 1 Week Physical Activity Recall Questionnaire | M =37.8 ±12.7 yrs.  F =39.6 ±17.0 yrs. | 118 | M = 56  F = 62 | Questionnaire administered 3 days after initial administration.  Men  Women | ICC=0.45; 95% CI =0.20-0.64  ICC=0.80; 95% CI =0.69-0.87 | Helmorhorst (2012) |
| Van der Ploeg et al. (2010)^[178]^ | Time Use Diary | 18-63 yrs. | 134 | M = 79  F = 55 | Time Use Diary (Day 2-3) compared to time use diary (Day 9-10). Results for all participants (not including similar activity and non-similar activity). | ICC_Light_=0.50 (95%CI=0.36-0.62)  ICC_MVPA_=0.53 (95%CI=0.39-0.64)  ICC_Others_=0.38-0.63 | Foley (2012) |
| Van der Ploeg et al. (2010)^[179]^ | International Physical Activity Questionnaire | 18-65 yrs.. | 1344 | M = 581  F = 763 | IPAQ administered 3 days after initial administration  Walking (mins/week)  IPAQ administered 7 days after initial administration  Walking (mins/week) | r_s_=0.69-0.91  r_s_=0.72 | Kim (2013) |
| Vandelanotte et al. (2005)^[180]^ | International Physical Activity Questionnaire (computerized) | 30.9±11.0 yrs. | 53 | M = 23  F = 30 | IPAQ administer 7 days (Time 2) and 11, 12, 13 or 14 (Time 3) days after initial administration.  IPAQ determined Total PA (mins)  IPAQ determined Total PA (kcal)  IPAQ determined MVPA (mins)  IPAQ determined MVPA (kcal)  IPAQ determined VPA (mins)  IPAQ determined VPA (kcal) | ICC=0.69  ICC=0.69  ICC=0.66  ICC=0.69  ICC=0.81  ICC=0.82 | Kim (2013) |
| Yore et al. (2007)^[186]^ | Behavioral Risk Factor Surveillance System | 44.5±15.7 yrs. | 60 | M = 30  F = 30 | Questionnaire administered 1-5 days after initial administration.  VPA  MPA  Recommended PA  Walking  Strengthening | Kappa = 0.86; 95%CI: 0.72-0.99  Kappa = 0.53; 95%CI: 0.31-0.75  Kappa = 0.84; 95%CI: 0.69-0.99  Kappa = 0.56; 95%CI: 0.34-0.77  Kappa = 0.92; 95%CI: 0.81-1.00 | Helmorhorst (2012) |

CAQ = College Alumnus Questionnaire; IPAQ = International Physical Activity Questionnaire; BRFSS = Behavioral Risk Factor Surveillance System; CHAMPS = Community Healthy Activities Model Program for Seniors; YPAS = Yale Physical Activity Survey; PASE = Physical Activity Scale for the Elderly; NPAQ = Neighbourhood PA Questionnaire; FITT = Frequency, Intensity, Type and Time; 7DPARQ = 7 Day Physical Activity Recall Questionnaire; HUNT1 = Nord- Trøndelag Health Study Questionnaire 1; HUNT2 = Nord-Trøndelag Health Study Questionnaire 2; NZPAQ = New Zealand Physical Activity Questionnaire; PA = Physical Activity; MPA = Moderate Physical Activity; VPA = Vigorous Physical Activity; MET = Metabolic Equivalent; EE = Energy Expenditure; PAEE = Physical Activity Energy Expenditure; ICC = Intraclass Correlation Coefficient; CI = Confidence Intervals; r = Correlation Coefficient; rs = Spearman Correlation Coefficient; MeanDIFF = Mean Difference; rp = Pearsons Correlation Coefficient

**Supplementary Table 4b.** Test-retest reliability of self-reported measures of physical activity/energy expenditure within a duration of between 1 week and 4 weeks.

| **Author** | **Measure** | **Age Range** | **Sample Size** | **Sex** | **Details** | **Results** | **Primary Source** |
| --- | --- | --- | --- | --- | --- | --- | --- |
| Ainsworth et al. (1993)^[196]^ | Lipid research Clinic Questionnaire | 39.8 ±9.1 yrs. | 78 | M = 28  F = 50 | LRCQ administered at baseline and after 26 days.  2 point score  4 point score | r_p_=0.85  r_p_=0.88 | LaMonte (2001) |
| Ainsworth et al. (2000)^[197]^ | Kaiser Physical Activity Survey | 20-60 yrs | 50 | F = 50 | Test retest completed within 1 month.  3 point summary index  4 point summary index | ICC=0.82; p<0.001  ICC=0.83; p<0.001 | Helmorhorst (2012) |
| Besson et al. (2010)^[74]^ | Recent Physical Activity Questionnaire | 31-57 yrs. | 131 | M = 60  F = 71 | RPAQ determined PAEE repeated approximately 2 weeks apart. | ICC=0.76; p<0.001 | Helmorhorst (2012) |
| Bharathi et al. (2000)^[200]^ | Physical Activity Questionnaire | 18-60 yrs. | 112 | M = 45  F = 67 | Questionnaire administered at baseline at 2-4 weeks after initial administration.  Total daily Energy Expenditure  Energy Expenditure of exercise  Energy Expenditure of hobbies  Energy Expenditure of household chores  Total Energy Expenditure of occupational activities  Residual Energy Expenditure  Physical activity levels | r=0.86; p<0.01  r=0.71; p<0.01  r=0.62; p<0.01  r=0.85; p<0.01  r=0.70; p<0.01  r=0.50; p<0.01  r=0.54; p<0.01 | Van Poppel (2010) |
| Booth et al. (1996)^[195]^ | Self-reported Physical Activity | 18-79 yrs. | 116 | M = 55  F = 61 | 2 measures of self-reported examined at baseline and 2 weeks after initial administration.  Total Activity  Vigorous Activity  Moderate Activity  Walking  Energy Expenditure | ICC=0.64; 95%CI = 0.52-0.73; 95%LoA = 14.9  ICC=0.67; 95%CI = 0.56-0.76; 95%LoA = 5.5  ICC=0.55; 95%CI = 0.42-0.67; 95%LoA = 8.8  ICC=0.74; 95%CI = 0.65-0.81; 95%LoA = 8.8  ICC=0.58; 95%CI = 0.45-0.69; 95%LoA = 8.7 | Pennathur (2003) |
| Bouchard et al. (1983)^[202]^ | Bouchard Activity Record | 10-50 yrs. | 300 | NA | BAR repeated 6-10 days after initial administration. | r=0.86-0.95 | Foley (2012) |
| Brown et al. (2008)^[115]^ | Active Australia Physical Activity Survey | 54-59 yrs. | 159 | F = 159 | AAPAS administered between 7-28 days after initial administration  PA frequency per week  Total minutes PA per week. | r_s_=0.58  r_s_=0.64 | Helmorhorst (2012) |
| Chinapaw et al. (2009)^[207]^ | Activity Questionnaire for Adults and Adolescents | 28.9±3.5 yrs. | 58 | M = 20  F = 28 | Questionnaire administered twice with an interval of 2 weeks. | ICC=-0.005-0.58 | Foley (2012) |
| Deng et al. (2008)^[124]^ | IPAQ Chinese (Short form) | >50 yrs. | 224 | M = 76  W = 148 | Test retest reliability after 9 days from initial assessment  Total PA  Walking  Moderate PA  Vigorous PA | ICC=0.84  ICC= 0.85  ICC=0.81  ICC=0.83 | Forsen (2010) |
| De Abajo et al. (2001)^[211]^ | Yale Physical Activity Survey | 61-80 yrs. | 108 | M = 38  F = 70 | YPAS administered 2 weeks after initial administration  Total Time spent in PA  Total Energy Expenditure  YPAS Summary Index | ICC=0.66; p<0.001  ICC=0.65; p<0.001  ICC=0.31; p<0.01 | Helmorhorst (2012) |
| Dipietro et al. (1993)^[213]^ | Yale Physical Activity Survey | 60-86 yrs. | 76 | M =20  F = 56 | YPAS administered at baseline and 14 days after initial administration.  Total Time (hrs.wk^-1^)  Energy Expenditure (kcal.wk^-1^)  Summary (total units)  Vigorous (units.month^-1^)  Leisurely walk (units.month^-1^)  Moving (hrs.d^-1^)  Standing (hrs.d^-1^) | Mean_DIFF_=3.68; r_p_=0.57; p<0.001  Mean_DIFF_=873.42; r_p_=0.58; p<0.001  Mean_DIFF_=1.31; r_p_=0.65; p<0.001  Mean_DIFF_=4.66; r_p_=0.61; p<0.001  Mean_DIFF_=-1.6; r_p_=0.48; p<0.001  Mean_DIFF_=-0.12; r_p_=0.49; p<0.001  Mean_DIFF_=0.51; r_p_=0.48; p<0.001 | LaMonte (2001) |
| Dolan et al. (2006)^[214]^ | Bone Loading History Questionnaire | 31 ± 7.7 yrs. | 80 | F = 80 | Questionnaire administered at baseline and 4-6 weeks after initial administration.  Total self-reported spine bone loading  Total self-reported spine bone loading | ICC=0.92; p<0.001  ICC=0.89; p<0.001 | Van Poppel (2010) |
| Dubbert et al. (2004)^[215]^ | 7 day Physical Activity Recall Questionnaire | 60-80 yrs. | 220 | M = 220 | 7DPAR administered 2-4 weeks after initial administration  Total Energy Expenditure | ICC=0.89; p<0.001 | Helmorhorst (2012) |
| Duncan et al. (2001)^[128]^ | 7 day Physical Activity Recall Questionnaire | 47.8±7.1 yrs. | 94 | M = 18  F = 76 | 7DPAR administered 7 days after initial administration  Total Energy Expenditure | ICC=0.44; 95% CI =0.26-0.59 | Helmorhorst (2012) |
| Folsom et al. (1986)^[218]^ | Minnesota Leisure Time Physical Activity Questionnaire | NA | 290 | NA | Questionnaire administered at baseline and 5 weeks after initial administration.  Total physical activity  Light, Moderate and Vigorous Intensity | r_s_=0.79-0.88  r_s_=0.69-0.86 | Shephard (2003) |
| Giles et al. (2009)^[267]^ | Community Healthy Activities Model Program for Seniors (CHAMPS) | 65-74 years | 73 | M = 28  F = 45 | CHAMPS administered at baseline and <2 weeks after initial assessment.  Walking Frequency  MPA Frequency  VPA Frequency  Total Activity Frequency  Walking Duration  MPA Duration  VPA Duration  Total PA Duration  Walking Volume (MET.min.wk)  MPA Volume (MET.min.wk)  VPA Volume (MET.min.wk)  Total Activity Volume (MET.min.wk) | ICC=0.93  ICC=0.83  ICC=0.86  ICC=0.89  ICC=0.83  ICC=0.79  ICC=0.79  ICC=0.81  ICC=0.85  ICC=0.80  ICC=0.78  ICC=0.84 |  |
| Hagiwara et al. (2008)^[223]^ | Physical activity scale for the elderly (PASE) | >65 yrs. | 325 | M =134  F = 191 | PASE repeated 3-4 weeks after initial assessment. | Weighted Kappa=  0.33-0.71 | Forsen (2010) |
| Harada et al. (2001)^[224]^ | CHAMPS physical activity questionnaire | >65 yrs. | 87 | M = 33  F = 54 | 2 week test retest reliability of CHAMPS determined kcal/wk | ICC=0.62-0.76  r_p_=0.62-0.76 | Forsen (2010) |
| Hopkins et al. (1991)^[225]^ | Life in New Zealand Survey | M = 44 ± 16 yrs.  F = 43 ± 16 yrs. | 259 | M = 132  F = 127 | Two week test-retest reliability of physical activity measures (recalled from last 4 weeks of behaviours.  High Intensity Physical Activities  Low Intensity Physical Activities | ICC = 0.7-0.88  ICC = 0.60–0.72 | Lamonte (2001) |
| Lagerros et al. (2006)^[189]^ | Energy expenditure questionnaire | 20-59 yrs. | 293 | M=137  F=156 | EEQ administered at baseline and 3 times over three weeks. | ICC=0.55; 95%CI = 0.47-0.64 | Van Poppel (2010) |
| Levin et al (1999)^[233]^ | 4 Week History Questionnaire | 37.4 ± 9.7 yrs. | 77 | M = 28  F = 49 | 4WHQ completed during 14 clinic visits 26 days apart (MET minutes of activity per day). | ICC = 0.59 | LaMonte (2001) |
| Levin et al (1999)^[233]^ | Physical Activity Records | 37.4 ± 9.7 yrs. | 77 | M = 28  F = 49 | PAR completed 48 hours prior to 14 clinic visits 26 days apart (MET minutes of activity per day). | ICC = 0.31 | LaMonte (2001) |
| Maddison et al. (2007)^[82]^ | IPAQ | 18 – 64 yrs. | 36 | M = 16  F = 20 | Administered at Day 0, Day 8 and Day 15. | r_s1-8_=0.79; p<0.001  r_s8-15_=0.74; p<0.001 | Westerterp (2009) |
| Maddison et al. (2007)^[82]^ | NZPAQ | 18 – 64 yrs. | 36 | M = 16  F = 20 | Administered at Day 0, Day 8 and Day 15. | r_s1-8_=NR;  r_s8-15_=0.69; p<0.001 | Westerterp (2009) |
| Matton et al. (2007)^[235]^ | Flemish Physical Activity Computerized Questionnaire | 48-78 yrs. | 49 | M =30  F = 19 | FPACQ test retest reliability with 2 week interval between administrations. | ICC=0.57-0.96 | Forsen (2010) |
| O’Brien-Cousins et al. (1996)^[237]^ | Older Adult Exercise Status Inventory (OAESI) | 58-80 yrs. | 17 | F = 17 | OAESI amount of exercise in the past week administered twice in a four week period. | r=0.11-0.76 | Forsen (2010) |
| Pettee et al. (2009)^[239]^ | Modifiable Activity Questionnaire | 52.6±5.4 yrs. | 66 | F = 66 | MAQ administered between 1 and 4 weeks after initial administration  MET-hr/week | ICC=0.64; 95% CI= 0.48 - 0.77 | Helmorhorst (2012) |
| Pettee et al. (2009)^[239]^ | Nurses health study physical activity questionnaire | 52.6±5.4 yrs. | 66 | F = 66 | NHSPAQ administered between 1 and 4 weeks after initial administration  MET-hr/week | ICC=0.48; 95% CI= 0.26-0.65 | Helmorhorst (2012) |
| Pettee et al. (2009)^[239]^ | Active Australia Physical Activity Survey | 52.6±5.4 yrs. | 66 | F = 66 | AAPAS administered between 1 and 4 weeks after initial administration  Mins/day | ICC=0.32; 95% CI= 0.09-0.52 | Helmorhorst (2012) |
| Pettee et al. (2009)^[239]^ | Womens health Initiative Physical Activity Questionnaire | 52.6±5.4 yrs. | 66 | F = 66 | WHIPAQ administered between 1 and 4 weeks after initial administration  MET-hr/week | ICC=0.91; 95% CI= 0.86-0.95 | Helmorhorst (2012) |
| Philippaerts et al. (1998)^[240]^ | Tecumseh Community Health Study Questionnaire | 30-40 yrs. | 90 | M = 90 | TCSHSQ administered at baseline and 1 month after initial administration.  Total Activity Index (MET)  Total daily energy expenditure (kcal/day) | ICC = 0.83  ICC = 0.89 | Kwak (2011) |
| Philippaerts et al. (1998)^[240]^ | Five City Projects Questionnaire | 30-40 yrs. | 90 | M = 90 | FCPQ administered at baseline and 1 month after initial administration.  7 day index (kcal/day)  Sweat index | ICC = 0.59  ICC = 0.60 | Kwak (2011) |
| Philippaerts et al. (1998)^[240]^ | Baecke Physical Activity Questionnaire | 30-40 yrs. | 90 | M = 90 | BPAQ administered at baseline and 1 month after initial administration.  Total Activity Index | ICC = 0.88 | Kwak (2011) |
| Pols et al. (1995)^[242]^ | Baecke Physical Activity Questionnaire | 20-70 yrs. | 126 | M = 64  F = 62 | Questionnaire administered 3 times over a 12 month period  Total Physical activity Men (Baseline – Month 5)  Total Physical activity Men (Baseline – Month 11)  Total Physical activity Women (Baseline – Month 5)  Total Physical activity Women (Baseline – Month 11) | r_p_=0.85; 95%CI = 0.76-0.91; Kappa=57.1%  r_p_=0.80; 95%CI = 0.69-0.88; Kappa=55.7%  r_p_=0.83; 95%CI = 0.72-0.90 Kappa=41.0%  r_p_=0.77; 95%CI = 0.63-0.86 Kappa=45.5% | Nielson (2009) |
| Rauh et al. (1992)^[244]^ | Physical Activity Recall | 33 ± 10.6 yrs. | 45 | M = 24  F = 21 | PAR administered at baseline and 2 weeks after initial administration.  Moderate activity  Hard activity  Very hard activity  Kcal.kg^-1^.wk^-1^. | r_p_=0.52; p<0.05  r_p_=0.33; p<0.05  r_p_=0.86; p<0.05  r_p_=0.69; p<0.05 | Prince (2008) |
| Rauh et al. (1992)^[244]^ | Harvard Alumni Survey | 33 ± 10.6 yrs. | 45 | M = 24  F = 21 | HAS administered at baseline and 2 weeks after initial administration.  Flights of Stairs walked  Blocks walked  Sports  Kcal.wk^-1^. | r_p_=0.68; p<0.05  r_p_=0.23; NS  r_p_=0.67; p<0.05  r_p_=0.34; p<0.05 | Prince (2008) |
| Rauh et al. (1992)^[244]^ | Vigorous Exercise Frequency | 33 ± 10.6 yrs. | 45 | M = 24  F = 21 | VEFQ administered at baseline and 2 weeks after initial administration. | r_p_=0.51; p<0.05 | Prince (2008) |
| Rauh et al. (1992)^[244]^ | National health Interview Survey | 33 ± 10.6 yrs. | 45 | M = 24  F = 21 | NHIS administered at baseline and 2 weeks after initial administration.  Walking | r_p_=0.31; p<0.05 | Prince (2008) |
| Rauh et al. (1992)^[244]^ | Godin Shephard Survey | 33 ± 10.6 yrs. | 45 | M = 24  F = 21 | GSS administered at baseline and 2 weeks after initial administration.  Strenuous activity  Moderate activity  Mild activity  Total (MET) | r_p_=0.84; p<0.05  r_p_=0.37; p<0.05  r_p_=0.47; p<0.05  r_p_=0.75; p<0.05 | Prince (2008) |
| Rauh et al. (1992)^[244]^ | Baecke Physical Activity Questionnaire | 33 ± 10.6 yrs. | 45 | M = 24  F = 21 | BPAQ administered at baseline and 2 weeks after initial administration.  Work  Sport  Leisure | r_p_=0.87; p<0.05  r_p_=0.79; p<0.05  r_p_=0.25; p<0.05 | Prince (2008) |
| Reed et al. (2005)^[245]^ | Godin Physical Activity Questionnaire | NA | 43 | NA | Usual week physical activity measured using the GPAQ tested at baseline and 1 week after. | r=0.82 | Andrews (2010) |
| Reed et al. (2005)^[245]^ | Home Environment Exercise Questionnaire | NA | 43 | NA | Usual week physical activity measured using the GPAQ tested at baseline and 1 week after. | r=0.85 | Andrews (2010) |
| Reis et al. (2005)^[246]^ | Occupational Physical Activity Questionnaire | 38.8±9.9 yrs. | 41 | M = 13  F = 28 | Questionnaire administered 2 weeks after initial administration. | ICC=0.76; 95% CI =0.59-0.86 | Helmorhorst (2012) |
| Richardson et al. (2001)^[158]^ | Stanford 7 day physical activity recall | 20-59 yrs. | 77 | M = 27  F = 50 | S7DPAR administered 1 month after initial administration  Total MET-mins/day (Men)  Total MET-mins/day (Women) | r_s_=0.60; p<0.01  r_s_=0.36; p<0.05 | Helmorhorst (2012) |
| Richardson et al. (1995)^[190]^ | The atherosclerosis risk in communities/ Baecke Questionnaire | 20-59 yrs. | 78 | M = 28  F = 50 | Questionnaire administered at baseline and 1 month after initial assessment  Total Leisure (Men)  Total Leisure (Women)  Sport and Leisure (Men)  Sport and Leisure (Women)  Non-Sport and Exercise Leisure (Men)  Non-Sport and Exercise Leisure (Women) | r_p_=0.92; p<0.01  r_p_=0.90; p<0.01  r_p_=0.92; p<0.01  r_p_=0.87; p<0.01  r_p_=0.88; p<0.01  r_p_=0.86; p<0.01 | Prince (2008) |
| Richardson et al. (1994)^[247]^ | Minnesota Leisure time questionnaire | 20-59 yrs. | 78 | NA | Test – retest correlation over a one month period | r=0.92 | Ainslie (2003) |
| Rutten et al. (2003)^[249]^ | IPAQ Short Form Last 7 days | > 18 yrs. | 951 | NA | IPAQ variables (all) across countries.  1-3 weeks after initial questionnaire responded. | ICC=0.3-0.62 | Andrew (2010) |
| Saglam et al. (2010)^[161]^ | International Physical Activity Questionnaire (long) | 18–32 yrs | 1097 | M = 376  F = 721 | IPAQ administered 3 to 7 days after initial administration  Total MET-mins/week | r_s_=0.64; 95% CI = 0.56-0.72; p<0.001 | Helmorhorst (2012) |
| Saglam et al. (2010)^[161]^ | International Physical Activity Questionnaire (short) | 18–32 yrs | 1097 | M = 376  F = 721 | IPAQ administered 3 to 7 days after initial administration  Total MET-mins/week | r_s_=0.69; 95% CI = 0.61-0.77; p<0.001 | Helmorhorst (2012) |
| Sallis et al. (1985)^[250]^ | Five City Project Questionnaire | 11-74 years | 64 | NA | Two week test-retest reliability of 7 day recall questionnaire.  Light Activity  Moderate Activity  Hard Activity  Very hard Activity  Kcal.d^-1^  kcal.kg.d^-1^ | r=0.65; p<0.001  r=0.08; p<0.001  r=0.31; p<0.001  r=0.61; p<0.001  r=0.67; p<0.001  r=0.34; p<0.001 | Dishman (2001) |
| Schuler et al. (2001)^[251]^ | Yale Physical Activity Survey | 56-86 yrs. | 56 | M = 25  F = 31 | Total Time  Total Energy Expenditure  Total Activity Summary Index  Vigorous  Walking  Moving  Standing  Data collected at baseline and 2 weeks after initial administration. | r_s_=0.74; p<0.05  r_s_=0.72; p<0.05  r_s_=0.55; p<0.05  r_s_=0.48; p<0.05  r_s_=0.41; p<0.05  r_s_=0.60; p<0.05  r_s_=0.22 | Pennathur (2003) |
| Smitherman et al. (2009)^[253]^ | Jackson Heart Study Physical Activity Survey | 54.4±15.7 yrs. | 40 | M = 20  F = 20 | JHSPAS administered 2 weeks after initial administration  Total score | ICC=0.99 | Helmorhorst (2012) |
| Sobngwi et al. (2001)^[167]^ | Sub-Saharan Africa Activity Questionnaire | 19-68 yrs. | 89 | M = 44  F = 45 | Questionnaire administered 10-15 days after initial administration. | r_s_=0.95; p<0.01 | Helmorhorst (2012) |
| Stein et al. (1993)^[254]^ | Behavioural Risk Factor Surveillance Survey | 40.7 ± 16.7 yrs. | 210 | M=87  F=123 | Participants completed the telephone interview administered questionnaire at baseline and a minimum of 21 days after initial administration.  Regular Aerobic Exercise | Kappa=0.45; p<0.001 | Washburn (2000) |
| Thuy et al. (2010)^[174]^ | Global Physical Activity Questionnaire | 25-64 yrs. | 238 | M = 120  F = 118 | GPAQ administer 3 weeks after initial administration.  GPAQ determined Total physical activity (Men)  GPAQ determined Total physical activity (Women) | r_s_=0.32  r_s_=0.13 | Kim (2013) |
| Thuy et al. (2010)^[174]^ | International Physical Activity Questionnaire | 25-64 yrs. | 229 | M = 115  F = 114 | IPAQ administer 3 weeks after initial administration.  IPAQ determined Total physical activity (Men)  IPAQ determined Total physical activity (Women) | r_s_=0.34  r_s_=0.20 | Kim (2013) |
| Tomioka et al (2011)^[177]^ | International Physical Activity Questionnaire | 65-89 yrs. | 325 | M = 164  F = 161 | IPAQ administered 2 weeks after initial administration  MET-min/week (Men)  MET-min/week (Women) | ICC=0.50-0.65  ICC=0.56-0.57 | Helmorhorst (2012) |
| Trinh et al. (2009)^[256]^ | Global Physical Activity Questionnaire | 25-64 yrs. | 169 | M = 82  F = 87 | GPAQ administered 2 weeks (dry season) after initial administration  GPAQ total score.  GPAQ administered 2 months (wet season) after initial administration  GPAQ total score. | r_s_=0.69; p<0.001  Mean_DIFF_=1.00; 95% CI =0.03-31.82  r_s_=0.55; p<0.001  Mean_DIFF_=1.12; 95% CI =0.02-71.09 | Helmorhorst (2012) |
| Voorrips et al. (1991)^[257]^ | Baecke physical activity questionnaire (Elderly) | 63-80 yrs. | 29 | NA | Questionnaire administered twice within a 20 day interval. | r_s_=0.89; Mean_DIFF_=-0.4 ±2.6 (Baecke Score) | Haskell et al (2000) |
| Vuillemin et al. (2000)^[258]^ | Modifiable Activity Questionnaire | 36-63 yrs. | 84 | M = 22  F = 62 | MAQ (self-administration) determined leisure physical activity (hrs-week) - MAQ (Interview-administered) determined leisure physical activity (hrs-week)  MAQ (self-administration) determined leisure physical activity (MET hrs-week) - MAQ (Interview-administered) determined leisure physical activity (MET hrs -week)  MAQ self-administered completed 7.9 (5.8) days after and MAQ interview-administered questionnaire (unstructured) (Past year activity examined). | ICC = 0.82-0.93  ICC = 0.82-0.93 | Kim (2013) |
| Weller et al. (1998)^[263]^ | Canada Fitness Survey Questionnaire | 49.2 ± 7.1 yrs. | 127 | M = 64  F = 63 | Physical activity behaviours were examined using CFSQ at baseline and 3-4 weeks after initial assessment.  Total leisure and non-leisure activities performed in last week.  Total leisure and non-leisure activities performed in last month.  Total leisure and non-leisure activities performed in last year.  Total leisure and non-leisure activities performed in last (EE.wk^1^)  Energy expended during strenuous activities (>6 METs).  Energy expended during moderate activities (4-6 METs).  Energy expended during light activities (<4 METs). | ICC=0.53  ICC=0.50  ICC=0.47  ICC=0.48  ICC=0.79  ICC=0.02  ICC=0.50 | Shephard (2012) |
| Yore et al. (2007)^[186]^ | Behavioral Risk Factor Surveillance System | 44.5±15.7 yrs. | 60 | M = 30  F = 30 | Questionnaire administered 10-19 days after initial administration.  VPA  MPA  Recommended PA  Walking  Strengthening | Kappa = 0.80; 95%CI: 0.65-0.95  Kappa = 0.35; 95%CI: 0.11-0.59  Kappa = 0.67; 95%CI: 0.46-0.88  Kappa = 0.34; 95%CI: 0.10-0.57  Kappa = 0.85; 95%CI: 0.71-0.99 | Helmorhorst (2012) |
| Yore et al. (2005)^[266]^ | Behavioral Risk Factor Surveillance System | 39.5± 13 yrs. | 45 | M = 25  F = 20 | Participants completed an interview administered questionnaire 3 times in the space of 3 weeks; twice during the first week and once during the third week.  Occupational Physical Activity Classification; Survey 1 – Survey 2  Occupational Physical Activity Classification; Survey 2 – Survey 3  Occupational Physical Activity Classification; Survey 1 – Survey 3 | Kappa = 1.0; %Agreement = 100%  Kappa = 0.45; %Agreement = 82%  Kappa = 0.40; %Agreement = 81% | Pierannunzi (2013) |

LRCQ = Lipid research Clinic Questionnaire; RPAQ = Recent Physical Activity Questionnaire; BAR = Bouchard Activity Record; AAPAS = Active Australia Physical Activity Survey; IPAQ = International Physical Activity Questionnaire; YPAS = Yale Physical Activity Survey; 7DPARQ = 7 day physical activity recall questionnaire; CHAMPS = Community Healthy Activities Model Program for Seniors; PASE = Physical Activity Scale for the Elderly; EEQ = Energy Expenditure Questionnaire; 4WHQ = 4 Week History Questionnaire; PAR = Physical Activity Record; NZPAQ = New Zealand Physical Activity Questionnaire; OAESI = Older Adult Exercise Status Inventory; MAQ = Modifiable Activity Questionnaire; NHSPAQ = Nurses’ Health Study Physical Activity Questionnaire; AAPAS = Active Australia Physical Activity Survey; WHIPAQ = Women’s health Initiative Physical Activity Questionnaire; TCHSQ = Tecumseh Community Health Study Questionnaire; FCPQ = Five City Projects Questionnaire; BPAQ = Baecke Physical Activity Questionnaire; HAS = Harvard Alumni Survey; VEFQ = Vigorous Exercise Frequency Questionnaire; NHIS = National Health Interview Survey; GSS = Godin Shephard Survey; S7DPAR = Stanford 7 day physical activity recall; JHSPAS = Jackson Heart Study Physical Activity Survey; GPAQ = Global Physical Activity Questionnaire; PA = Physical Activity; MPA = Moderate Physical Activity; VPA = Vigorous Physical Activity; PAEE = Physical Activity Energy Expenditure; ICC = Intraclass Correlation Coefficient; CI = Confidence Intervals; r = Correlation Coefficient; rs = Spearman Correlation Coefficient; MeanDIFF = Mean Difference; rp = Pearson’s Correlation Coefficient.

**Supplementary Table 4c.** Test-retest reliability of self-reported measures of physical activity/energy expenditure within a duration of between 4 weeks and 8 weeks.

| **Author** | **Measure** | **Age Range** | **Sample Size** | **Sex** | **Details** | **Results** | **Primary Source** |
| --- | --- | --- | --- | --- | --- | --- | --- |
| Batty (2000)^[198]^ | Physical Activity Questionnaire (Cadbury study) | 47.39 (SE = 0.93) yrs. | 54 | M = 54 | Questionnaire administered 4-6 weeks after initial administration  Work description  Overall work activity  Walking  Bike use  Active hobbies  Strenuous Activity  Overall Leisure activity | Kappa = 0.56; p<0.001  Kappa = 0.49; p<0.001  Kappa = 0.19; p<0.001  Kappa = 0.63; p<0.001  Kappa = 0.39; p<0.001  Kappa = 0.57; p<0.001  Kappa = 0.69; p<0.001 | Kwak (2011) |
| Berthouze et al. (1993)^[199]^ | Saint Etienne Physical Activity Questionnaire | 56-72 yrs. | 20 | M = 7  F = 13 | Mean Habitual Daily Energy Expenditure  Questionnaire completed at baseline and 6 weeks after initial assessment. | r=0.997; p<0.001 | Meyer (2009) |
| Friedenreich et al. (1998)^[219]^ | Past Year Total Physical Activity Questionnaire | 61.2 ± 6.4 yrs. | 113 | F = 113 | Questionnaire administered at baseline and 6-8 weeks after initial administration.  Total lifetime activity  Occupational activity  Household  Exercise/Sports | r_p_=0.74  r_p_=0.87  r_p_=0.77  r_p_=0.72 | Kwak (2011) |
| Singh et al. (1996)^[252]^ | Physical Activity Questionnaire |  | 112 | M=59  F=53 | Questionnaire administered at baseline and 6 weeks after initial administration.  Pace of Walking  Physical Activity index  Stairs (MET.min.wk^-1^)  Blocks (MET.min.wk^-1^)  Sports and Recreation (MET.min.wk^-1^)  Run-Walk-Jog Index (MET.min.wk^-1^)  Speed (mph)  Duration (mins.wk^-1^)  Sweat Frequency (workouts.wk^-1^)  Sweat Duration (mins.workout) | r=0.55-0.90; p<0.05  r=0.56-0.80; p<0.01  r=0.48-0.82; p<0.01  r=0.41-0.84; p<0.05  r=0.53-0.81; p<0.01  r=0.70-0.85; p<0.001  r=0.52-0.72; p<0.05  r=0.70-0.77; p<0.01  r=0.49-0.89; p<0.05  r=0.48-0.82; p<0.05 | Van Poppel (2010) |
| Trinh et al. (2009)^[256]^ | Global Physical Activity Questionnaire | 25-64 yrs. | 169 | M = 82  F = 87 | GPAQ administered 2 weeks (dry season) after initial administration  GPAQ total score.  GPAQ administered 2 months (wet season) after initial administration  GPAQ total score. | r_s_=0.69; p<0.001  Mean_DIFF_=1.00; 95% CI =0.03-31.82  r_s_=0.55; p<0.001  Mean_DIFF_=1.12; 95% CI =0.02-71.09 | Helmorhorst (2012) |
| Wendel-Vos et al. (2003)^[183]^ | Short Questionnaire to Assess Health enhancing  physical activity | 27 – 58 yrs. | 50 | M = 36  F = 14 | SQUASH administered at baseline and 5 weeks after initial administration. | r_s_=0.58; p< 0.001; 95%CI = 0.36-0.74 | Prince (2008) |
| Yasunaga et al. (2007)^[265]^ | Physical Activity Questionnaire for Elderly Japanese | >65 yrs. | 147 | M = 61  F = 86 | Test retest reliability 3-7 week’s post initial administration in the elderly. | r_p_=0.61-0.70 | Forsen (2010) |

GPAQ = Global Physical Activity Questionnaire; SQUASH = Short Questionnaire to Assess Health enhancing physical activity; IPAQ = International Physical Activity Questionnaire; MET = Metabolic Equivalent; r = Correlation Coefficient; rs = Spearman Correlation Coefficient; rp = Pearson’s Correlation Coefficient; CI = Confidence Intervals; MeanDIFF = Mean Difference.

**Supplementary Table 4d.** Test-retest reliability of self-reported measures of physical activity/energy expenditure within a duration of between 8 weeks and 1 year.

| **Author** | **Measure** | **Age Range** | **Sample Size** | **Sex** | **Details** | **Results** | **Primary Source** |
| --- | --- | --- | --- | --- | --- | --- | --- |
| Ainsworth et al. (1993)^[109]^ | College Alumnus Questionnaire | 38±9 yrs. | 78 | M = 28  W = 50 | CAQ PA Index test retest reliability month 1 to 8  CAQ PA Index test retest reliability month 1 to 9  CAQ PA Index test retest reliability month 8 to 9  CAQ Flights of Stairs test retest reliability month 1 to 8  CAQ Flights of Stairs test retest reliability month 1 to 9  CAQ Flights of Stairs test retest reliability month 8 to 9  CAQ City Blocks Walked test retest reliability month 1 to 8  CAQ City Blocks Walked test retest reliability month 1 to 9  CAQ City Blocks Walked test retest reliability month 8 to 9  CAQ Sport and Recreation test retest reliability month 1 to 8  CAQ Sport and Recreation test retest reliability month 1 to 9  CAQ Sport and Recreation test retest reliability month 8 to 9 | r=0.34; p<0.05  r=0.43  r=0.72; p<0.01  r=0.59; p<0.01  r=0.59; p<0.01  r=0.88; p<0.01  r=0.27; p<0.05  r=0.38; p<0.01  r=0.55; p<0.01  r=0.23  r=0.25  r=0.43; p<0.01 | Andrews (2010) |
| Brownson et al. (1999)^[204]^ | Behavioural Risk Factor Surveillance System | >40 yrs. | 199 | F=199 | Questionnaire administered at baseline and 12 months after initial administration.  Regular Physical Activity  Vigorous Physical Activity  Occupational Physical Activity  Housework Activity | Kappa= 0.26; %_DISCORDANCE_= 12.4; 95% CI = 7.6-17.2  Kappa= 0.30; %_DISCORDANCE_= 13.4; 95% CI = 8.4-18.4  Kappa= 0.51; %_DISCORDANCE_= 15.4; 95% CI = 10.2-20.6  Kappa= 0.45; %_DISCORDANCE_= 27.3; 95% CI = 20.9-33.7 | Washburn (2000) |
| Chasan-Taber et al. (2002)^[187]^ | Self -Administered Physical Activity Questionnaire | 39-65 yrs. | 131 | F=131 | Questionnaire administered at baseline and 1 year after initial administration.  Total Activity  Moderate Activity  Vigorous Activity  Recreational Activity  Household Activity | ICC=0.82  ICC=0.79  ICC=0.86  ICC=0.80  ICC=0.73 | Van Poppel (2010) |
| Cust et al. (2008)^[209]^ | European Prospective Investigation into Cancer and Nutrition Questionnaire | 50-65 yrs. | 182 | M = 100  F = 82 | EPIC administered at baseline and after 10 month period.  Reliability of continuous measures  Reliability of categorical measures | r_s_=0.63-0.73  ICC=0.62-0.66 | Forsen (2010) |
| Cust et al. (2009)^[208]^ | European Prospective Investigation into Cancer and Nutrition Questionnaire | 50-65 yrs. | 177 | M = 97  F = 80 | EPIC administered 10 months after initial administration  High Confidence Total PA Index  Low Confidence Total PA Index  High Confidence Cambridge PA Index  Low Confidence Cambridge PA Index | Kappa=0.65; 95% CI = 0.53-0.76;  Kappa=0.58; 95% CI = 0.45-0.71  Kappa=0.73; 95% CI = 0.61-0.84  Kappa=0.59; 95% CI = 0.47-0.71 | Helmorhorst (2012) |
| Cust et al. (2009)^[208]^ | International Physical Activity Questionnaire | 50-65 yrs. | 177 | M = 97  F = 80 | IPAQ administered 10 months after initial administration  High Confidence Total MET-hrs/week.  Low Confidence Total MET-hrs/week. | r_s_=0.53; 95% CI = 0.36-0.67  r_s_=0.33; 95% CI = 0.11-0.52 | Helmorhorst (2012) |
| Cust et al. (2008)^[209]^ | EPIC Physical activity questionnaire | 50-65 yrs. | 182 | M = 100  F = 82 | EPIC administered at baseline and after 10 month period.  Reliability of continuous measures  Reliability of categorical measures | r_s_=0.63-0.73  ICC=0.62-0.66 | Forsen (2010) |
| Eagan et al. (2005)^[216]^ | Historical Physical Activity Questionnaire | 20.6 ± 2.7 yrs. | 31 | F = 31 | Questionnaire administered at baseline and 6.5 months after initial administration.  Cumulative recall MET.hrs.d^-1^  Cumulative recall Hrs.d^-1^  Occupation PA MET.hrs.d^-1^  Occupation PA Hrs.d^-1^  Athletic PA MET.hrs.d^-1^  Athletic PA Hrs.d^-1^  Leisure PA MET.hrs.d^-1^  Leisure PA Hrs.d^-1^  Exercise PA MET.hrs.d^-1^  Exercise PA Hrs.d^-1^ | r_s_=0.76  r_s_=0.51  r_s_=0.48  r_s_=0.35  r_s_=0.82  r_s_=0.82  r_s_=0.70  r_s_=0.68  r_s_=0.55  r_s_=0.55 | Van Poppel (2010) |
| Friedenreich et al. (2006)^[131]^ | Past Year Total Physical Activity Questionnaire | 35-65 yrs. | 154 | M = 75  F = 79 | Test retest reliability examined an average of 9 weeks from initial administration. | ICC = 0.66; 95% CI = 0.56-0.74  r_s_=0.64; p <0.001 | Helmorhorst (2012) |
| Hekler et al. (2012)^[136]^ | CHAMPS | 66-80 yrs. | 870 | M = 377  F = 493 | CHAMPS determined mins/wk Low LIPA  CHAMPS determined mins/wk High LIPA  CHAMPS determined mins/wk MVPA  CHAMPS determined mins/wk Total PA  CHAMPS determined caloric expenditure/wk MVPA  CHAMPS determined caloric expenditure/wk Total  Questionnaire administered at baseline and after a 6 month period. | ICC=0.70  ICC=0.68  ICC=0.66  ICC=0.69  ICC=0.61  ICC=0.64 | Gorman (2014) |
| Jacobs et al. (1993)^[227]^ | Minnesota Leisure Time Questionnaire | 20-59 yrs. | 78 | M = 28  F = 50 | Past 12 months physical activity (SAFE Month 1-13 (12 months) and Month 13-14 (1 month))  Total PA (MET·mins·d^-1^)  Light PA (MET·mins·d^-1^)  Moderate PA (MET·mins·d^-1^)  Heavy PA (MET·mins·d^-1^)  Household Chores (MET·mins·d^-1^) | r=0.69-0.92  r=0.60-0.73  r=0.32-0.80  r=0.71-0.95  r=0.71-0.88 | Andrews (2010) |
| Jacobs et al. (1993)^[227]^ | Lipid Research Clinic | 20-59 yrs. | 78 | M = 28  F = 50 | Current physical activity 4 point score (SAFE Month 1-2 (1 month)) | r=0.93 | Andrews (2010) |
| Jacobs et al. (1993)^[227]^ | Godin Physical Activity Questionnaire | 20-59 yrs. | 78 | M = 28  F = 50 | Usual Week physical activity (SAFE Month 7-8 (1 month))  Leisure Index (Weighted times per week)  Light/Mild (Times per week)  Moderate (Times per week)  Strenuous (Times per week)  Usual Sweating (Times per week) | r=0.62  r=0.24  r=0.36  r=0.84  r=0.69 | Andrews (2010) |
| Jacobs et al. (1993)^[227]^ | Health Insurance Plan of NY Questionnaire | 20-59 yrs. | 66 | M = 23  F = 43 | Current physical activity 28 point score (SAFE Month 1-2 (1 month))  Work Index | r=0.86 | Andrews (2010) |
| Jacobs et al. (1993)^[227]^ | CARDIA Physical Activity History | 20-59 yrs. | 78 | M = 28  F = 50 | Past 12 months physical activity (SAFE Month 11-12 (1 month))  Total PA (Weighted Frequency)  Moderate PA (Weighted Frequency)  Heavy PA (Weighted Frequency) | r=0.88  r=0.66  r=0.91 | Andrews (2010) |
| Jacobs et al. (1993)^[227]^ | Baecke Physical Activity Questionnaire | 20-59 yrs. | 77 | M = 28  F = 49 | Current and past 12 months physical activity (SAFE Month 9-10 (1 month))  Total Index (15 point score)  Work Index (5 point score)  Sports Score (MET·mins·d^-1^)  Leisure Index (5 point score) | r=0.93  r=0.78  r=0.90  r=0.86 | Andrews (2010) |
| Jacobs et al. (1993)^[227]^ | 7 day Physical Activity Recall | 20-59 yrs. | 74 | M = 26  F = 49 | Past week physical activity (SAFE Month 11-12 (1 month))  Total (hrs·wk^-1^)  Light (hrs·wk^-1^)  Moderate (hrs·wk^-1^)  Hard (hrs·wk^-1^)  Very Hard (hrs·wk^-1^)  Heavy (hrs wk^-1^)  Occupational (MET·min·d^-1^)  Non-occupational (MET·min·d^-1^) | r=0.34  r=0.56  r=0.12  r=0.31  r=0.33  r=0.37  r=0.42  r=0.41 | Andrews (2010) |
| Jacobs et al. (1993)^[227]^ | College Alumnus (Paffenbarger) | 20-59 yrs. | 77 | M = 28  F = 49 | Current, past week or past 12 months physical activity (SAFE Month 1-9 (9 months) and Month 9-10 (1 month))  Total Index (MET·mins·d^-1^)  Stairs (MET·mins·d^-1^)  Walking (MET·mins·d^-1^)  Sports (MET·mins·d^-1^) | r=0.50-0.72  r=0.30-0.78  r=0.39-0.63  r=0.63-0.75 | Andrews (2010) |
| Jacobs et al. (1993)^[227]^ | Minnesota Heart Health Program | 20-59 yrs. | 77 | M = 28  F = 49 | Current physical activity (SAFE Month 3-4 (1 month))  Leisure Index (5 point score)  Work Index (MET·mins·d^-1^) | r=0.86  r=0.91 | Andrews (2010) |
| Jacobs et al. (1993)^[227]^ | Stanford Usual Activity | 20-59 yrs. | 78 | M = 28  F = 50 | Usual or past 3 months physical activity (SAFE Month 5-6 (1 month))  Moderate Index (6 point score)  Vigorous Index (5 point score) | r=0.77  r=0.67 | Andrews (2010) |
| Lee et al. (1992)^[232]^ | Past lifestyle habits questionnaire | 46.1 yrs. | 873 | M = 377  F = 496 | Reliability of physical activity energy expenditure (kcal.d^-1^) examined 11 years apart (1972 – 1983).  Recalled versus baseline  Recalled versus current  Current versus baseline | r_s_=0.38  r_s_=0.50  r_s_=0.35 | Shephard (2003) |
| Meyer et al. (2009)^[236]^ | Womens health Initiative physical activity questionnaire | 50-79 yrs. | 1092 | F = 1092 | Average test-retest of WHIPAQ was 3 months. | Weighted Kappa = 0.35-0.60  ICC=0.51-0.77 | Forsen (2010) |
| Nang et al. (2011)^[154]^ | International Physical Activity Questionnaire | NA | 43 | NA | IPAQ determined Vigorous Physical Activity examined a mean of 6 months apart.  IPAQ determined Moderate Physical Activity examined a mean of 6 months apart. | r_s_=0.38; p<0.05  r_s_=0.58; p<0.001 | Helmorhorst (2012) |
| Nang et al. (2011)^[154]^ | Singapore Prospective Study Program Physical Activity Questionnaire | NA | 43 | NA | SPSPPAQ determined Vigorous Physical Activity examined a mean of 6 months apart.  SPSPPAQ determined Moderate Physical Activity examined a mean of 6 months apart. | r_s_=0.75; p<0.001  r_s_=0.55; p<0.001 | Helmorhorst (2012) |
| Norman et al. (2001)^[156]^ | Physical activity questionnaire (self-administered) | 44-78 yrs. | 111 | M = 111 | PAQ administered at baseline and 6 months after initial administration.  PAQ determined MET hours per day^-1^ (crude total activity)  PAQ determined MET hours per day^-1^ (total activity score) | r_s_=0.65; p<0.05  r_s_=0.65; p<0.05 | Kwak (2011) |
| Orsini et al. (2007)^[238]^ | Swedish Mamography Cohort PA Questionnaire | 56-75 yrs. | 303 | F = 303 | 1 year test retest reliability  Current PA  PA at age 50  PA at age 30  PA at age 15 | ICC=0.64-0.74  ICC=0.66-0.79  ICC=0.71-0.85  ICC=0.76-0.81 | Forsen (2010) |
| Pols et al. (1996)^[241]^ | Modified Baecke questionnaire | 51-71 yrs. | 35 | F = 35 | Test retest of the questionnaire after 5 months  Test retest of the questionnaire after 11 months | r_p_=0.82; p<0.05  r_p_=0.73; p<0.05 | Forsen (2010) |
| Pols et al. (1996)^[241]^ | Pre-EPIC questionnaire | 51-71 yrs. | 35 | F = 35 | Test retest of the questionnaire after 5 months  Test retest of the questionnaire after 11 months | r_p_=0.42; p<0.05  r_p_=0.60; p<0.05 | Forsen (2010) |
| Pols et al. (1995)^[242]^ | Baecke Physical Activity Questionnaire | 20-70 yrs. | 126 | M = 64  F = 62 | Questionnaire administered 3 times over a 12 month period  Total Physical activity Men (Baseline – Month 5)  Total Physical activity Men (Baseline – Month 11)  Total Physical activity Women (Baseline – Month 5)  Total Physical activity Women (Baseline – Month 11) | r_p_=0.85; 95%CI = 0.76-0.91; Kappa=57.1%  r_p_=0.80; 95%CI = 0.69-0.88; Kappa=55.7%  r_p_=0.83; 95%CI = 0.72-0.90 Kappa=41.0%  r_p_=0.77; 95%CI = 0.63-0.86 Kappa=45.5% | Nielson (2009) |
| Pols et al. (1997)^[243]^ | Physical Activity Questionnaire (self-administered) | 50-70 yrs. | 126 | M = 64  F = 62 | Questionnaire administered at baseline and after 5 months  Occupational (Men)  Leisure Time (Men)  Total (Men)  Occupational (Women)  Leisure Time (Women)  Total (Women) | r_s_=0.90 (95% CI 0.84-0.94)  r_s_=0.85 (95% CI 0.76-0.91)  r_s_=0.76 (95% CI 0.63-0.85)  r_s_=0.79 (95% CI 0.65-0.87)  r_s_=0.68 (95% CI 0.50-0.81)  r_s_=0.58 (95% CI 0.36-0.74) | Kwak (2011) |
| Pols et al. (1997)^[243]^ | Physical Activity Questionnaire (self-administered) | 50-70 yrs. | 126 | M = 64  F = 62 | Questionnaire administered at baseline and after 11 months  Occupational (Men)  Leisure Time (Men)  Total (Men)  Occupational (Women)  Leisure Time (Women)  Total (Women) | r_s_=0.89 (95% CI 0.83-0.94)  r_s_=0.72 (95% CI 0.56-0.82)  r_s_=0.70 (95% CI 0.63-0.85)  r_s_=0.80 (95% CI 0.68-0.88)  r_s_=0.69 (95% CI 0.52-0.81)  r_s_=0.71 (95% CI 0.54-0.82) | Kwak (2011) |
| Roeykens et al. (1998)^[248]^ | MOSPA-Q | M = 35.9 yrs.  F = 35.7 yrs. | 167 | M = 108  F = 59 | Questionnaire administered at baseline and <3 months after initial administration.  Work (time scores mins/wk^-1^)  Transportation (time scores mins/wk^-1^)  Household chores (time scores mins/wk^-1^)  Leisure time physical activity (time scores mins/wk^-1^)  Work (energy expenditure kcal/wk^-1^)  Transportation (energy expenditure kcal/wk^-1^)  Household chores (energy expenditure kcal/wk^-1^)  Leisure time activity (energy expenditure kcal/wk^-1^)  Total (energy expenditure kcal/wk^-1^) | ICC = 0.68  ICC = 0.85  ICC = 0.62  ICC = 0.91  ICC = 0.67  ICC = 0.87  ICC = 0.57  ICC = 0.92  ICC = 0.87 | Kwak (2011) |
| Roeykens et al. (1998)^[248]^ | MOSPA-Q | M = 35.9 yrs.  F = 35.7 yrs. | 167 | M = 108  F = 59 | Questionnaire administered at baseline and >3 months after initial administration.  Work (time scores mins/wk^-1^)  Transportation (time scores mins/wk^-1^)  Household chores (time scores mins/wk^-1^)  Leisure time physical activity (time scores mins/wk^-1^)  Work (energy expenditure kcal/wk^-1^)  Transportation (energy expenditure kcal/wk^-1^)  Household chores (energy expenditure kcal/wk^-1^)  Leisure time activity (energy expenditure kcal/wk^-1^)  Total (energy expenditure kcal/wk^-1^) | ICC = 0.82  ICC = 055  ICC = 0.48  ICC = 0.75  ICC = 0.85  ICC = 0.53  ICC = 0.45  ICC = 0.70  ICC = 0.74 | Kwak (2011) |
| Stel et al. (2004)^[168]^ | LASA physical activity questionnaire (LAPAQ) (interview administered) | >65 yrs. | 348 | NA | Total number of activities performed.  Total number of activities performed (retested in same month).  Total number of activities performed (retested in same season).  Test-retest reliability of individual activity types  LAPAQ administered at baseline and after 12 months. | Weighted Kappa=  0.65; p<0.01  Weighted Kappa=  0.75; p<0.01  Weighted Kappa=  0.66; p<0.01  Weighted Kappa=  0.35-0.82 | Kowalski (2012) |
| Stewart et al. (2001)^[255]^ | CHAMPS physical activity questionnaire | 65-90 yrs. | 148 | M = 91  F = 57 | Test-retest reliability 6 months post initial administration in the elderly. | ICC=0.58-0.67 | Forsen (2010) |
| Susuki et al. (1998)^[171]^ | Self-administered Physical Activity Questionnaire | 35-73 yrs. | 81 | M = 95  F = 119 | Questionnaire administered at baseline and 1 year after initial administration.  Daily Energy Expenditure (Males only)  Daily Energy Expenditure (Females only)  Weekly Physical Activity (Males only)  Weekly Physical Activity (Females only) | r_p_=0.59; p<0.001  r_p_=0.62; p<0.001  r_p_=0.37; p<0.001  r_p_=0.43; p<0.001 | Shephard (2003) |
| Wareham et al. (2002)^[259]^ | EPIC-Norfolk Physical Activity Questionnaire | M =58.8 ±7.9 yrs.  F =55.4 ±6.7 yrs. | 173 | M = 84  F = 89 | Questionnaire administered 3 months after initial administration. | r_p_=0.72-0.74; p<0.05 | Helmorhorst (2012) |
| Wareham et al. (2003)^[260]^ | EPIC Questionnaire | 40-65 yrs. | 2271 | M = NA  W = NA | Administered at baseline and 18-21 months follow up | Weighted κ = 0.60; p<0.001 | Westerterp (2009) |
| Washburn et al. (1991)^[261]^ | Harvard Alumni Activity Survey | 25-65 yrs. | 645 | M = 271  F = 374 | HAAS test retest reliability with 7-12 weeks between the two tests. | r=0.58 | Andrews (2010) |
| Washburn et al. (1993)^[262]^ | Physical activity scale for the elderly | Mean = 75 yrs. | 668 | M = 256  F = 412 | Test-retest reliability 6 months post initial administration in the elderly. | r_p_=0.68-0.84 | Forsen (2010) |

CAQ = College Alumnus Questionnaire; EPICPAQ = European Prospective Investigation into Cancer and Nutrition Questionnaire; IPAQ = International Physical Activity Questionnaire; CHAMPS = Community Healthy Activities Model Program for Seniors; WHIPAQ = Women’s health Initiative Physical Activity Questionnaire; SPSPPAQ = Singapore Prospective Study Program Physical Activity Questionnaire; PAQ = Physical Activity Questionnaire; LAPAQ = LASA Physical Activity Questionnaire; HAAS = Harvard Alumni Activity Survey; PA = Physical Activity; LIPA = Light Intensity Physical Activity; MVPA = Moderate to Vigorous Intensity Physical Activity; M = Male; F = Female; NA = Not Available; r = Correlation Coefficient; rs = Spearman Correlation Coefficient; rp = Pearson’s Correlation Coefficient; CI = Confidence Intervals; ICC = Intraclass Correlation Coefficient.

**Supplementary Table 4e.** Test-retest reliability of self-reported measures of physical activity/energy expenditure within a duration of greater than 1 year.

| **Author** | **Measure** | **Age Range** | **Sample Size** | **Sex** | **Details** | **Results** | **Primary Source** |
| --- | --- | --- | --- | --- | --- | --- | --- |
| Blair et al. (1991)^[201]^ | Health and Lifestyle Questionnaire | 41.3 ± 10.2 yrs. | 451 | M=129  F=322 | Long term recall assessed between 1-10 years after initial assessment  Total Leisure Time PA  Light activities  Moderate activities  Vigorous Activities | M: r=0.20; p<0.05  F: r=0.29; p<0.05  M: r=0.17; p<0.05  F: r=0.25; p<0.05  M: r=0.17; NS  F: r=0.26; p<0.05  M: r=0.47; p<0.05  F: r=0.41; p<0.05 | Haskell (2000) |
| Chasan-Taber et al. (1996)^[206]^ | Self-Administered Physical Activity Questionnaire | NA | 238 | M = NA  F = NA | Self-administered PAQ administered at baseline and 2 years after initial administration.  Inactivity  Non-vigorous activity  Vigorous Activity | ICC = 0.39  ICC = 0.42  ICC = 0.52 | LaMonte (2001) |
| Iwai et al (2001)^[226]^ | Single Item PA Questions | 22-79 yrs. | 1075 | 302  295  275  452  440  409 | Interview administered at baseline and between 11.5-12.4 months after initial assessment  Time spent on sport and exercise (Male)  Daily Walking Time (Male)  Frequency of sport and exercise (Male)  Time spent on sport and exercise (Female)  Daily Walking Time (Female)  Frequency of sport and exercise (Female) | Weighted kappa=0.44 (95%CI=0.34-0.54)  Weighted kappa=0.34 (95%CI=0.26-0.43)  Weighted kappa=0.48 (95%CI=0.39-0.58)  Weighted kappa=0.40 (95%CI=0.33-0.48)  Weighted kappa=0.30 (95%CI=0.22-0.37)  Weighted kappa=0.55 (95%CI=0.47-0.63) | Van Poppel (2010) |
| Lee et al. (1992)^[232]^ | Past lifestyle habits questionnaire | 46.1 yrs. | 873 | M = 377  F = 496 | Reliability of physical activity energy expenditure (kcal.d^-1^) examined 11 years apart (1972 – 1983).  Recalled versus baseline  Recalled versus current  Current versus baseline | r_s_=0.38  r_s_=0.50  r_s_=0.35 | Shephard (2003) |
| Matthews at al. (2003)^[151]^ | Shanghai Women’s Health Study Physical  Activity Questionnaire | 55.3 ± 8.9 yrs. | 191 | F = 191 | Questionnaire administered at baseline and 2 years after initial administration.  Adult exercise participation (past 5 years)  Duration (hours.wk^-1^)  MPA MET hours.wk/yr  VPA MET hours.wk/yr  Total MET hours.wk/yr  Lifestyle activities | κ = 0.64; ICC=0.67  ICC=0.66  ICC = 0.59  ICC = 0.93  ICC = 0.70  ICC=0.14-0.54 | Nielson (2008) |
| Matthews at al. (2003)^[151]^ | Shanghai Women’s Health Study Physical  Activity Questionnaire | 55.3 ± 8.9 yrs. | 191 | F = 191 | Questionnaire administered at baseline and 2 years after initial administration.  Exercise participation in adolescence  Duration (hours.wk^-1^)  Average hours.wk/yr | κ = 0.85; ICC=0.83  ICC=0.58  ICC = 0.40 | Nielson (2008) |

PAQ = Physical Activity Questionnaire; PA = Physical Activity; M = Male; F = Female; MPA = Moderate Physical Activity; VPA = Vigorous Physical Activity; r = Correlation Coefficient; NS = Non-significant; ICC = Intraclass Correlation Coefficient; CI = Confidence Intervals.

**Supplementary Table 5.** Sensitivity/responsiveness of self-reported measures of physical activity/energy expenditure.

| **Author** | **Measure** | **Age Range** | **Sample Size** | **Sex** | **Details** | **Results** | **Primary Source** |
| --- | --- | --- | --- | --- | --- | --- | --- |
| Hamdorf (1992)^[269]^ | Human Activity Profile Questionnaire | 60-70 yrs. | 40 | F = 40 | Sensitivity to detect change after 26 weeks of intervention in elderly women (differences observed between intervention group and control group).  MAS = Maximum Activity Score  AAS = Adjusted Activity Score | ES_MAS_=0.46  ES_AAS_=0.49 | Davidson and de Morton (2007) |
| Stewart et al. (2001)^[255]^ | CHAMPS physical activity questionnaire | 65-90 yrs. | 148 | M = 91  F = 57 | Sensitivity to detect change in 6 month intervention in the elderly (differences observed between intervention group and control group). | ES _kcal Exp_= 0.38-0.42  ES _FREQ Meas_= 0.54-0.64 | Forsen (2010) |

CHAMPS = Community Healthy Activities Model Program for Seniors; ES = Effect Size; M= Male; F = Female.

**Supplementary Table 6:** Criterion validity of accelerometer activity monitor determined physical activity/energy expenditure.

| **Author** | **Measure** | **Age Range** | **Sample Size** | **Sex** | **Reference** | **Results** | **Primary Source** |
| --- | --- | --- | --- | --- | --- | --- | --- |
| Abel et al. (2008)^[270]^ | ActiGraph GT1M | 29.4 ± 7.1 yrs. | 20 | M = 10  F = 10 | AG determined steps – Direct Observation determined steps (@ 54 m.min^-1^).  AG determined steps – Direct Observation determined steps (@ 80 m.min^-1^).  AG determined steps – Direct Observation determined steps (@ 107 m.min^-1^).  AG determined steps – Direct Observation determined steps (@ 134 m.min^-1^).  AG determined steps – Direct Observation determined steps (@ 161 m.min^-1^).  AG determined steps – Direct Observation determined steps (@ 188 m.min^-1^).  Participants completed 6 treadmill trials at 54, 80, 107, 134, 161 and 188 m.min^-1^ while wearing the devices (Structured). | Underestimated; p<0.01; r=-0.37; NS  NS diff; r=-0.58; p<0.05  Underestimated; p<0.01; r=-0.69; p<0.05  NS diff; r=-0.64; p<0.05  Underestimated; p<0.01; r=-0.58; p<0.05  NS diff; r=-0.54; p<0.05 | Shephard (2012) |
| Adams et al. (2005)^[72]^ | ActiGraph 7164 | 49.1 ± 6.8 yrs. | 81 | F = 81 | ActiGraph determined counts.min^-1^.d^-1^ - Doubly labelled water determined energy expenditure (kcal.kg^-1^.d^-1^)  14 days of DLW and ActiGraph measurement compared to 1 day of PA recall from 24PARQ (Unstructured). | r=0.30; p<0.05 | Nielson (2009) |
| Allen et al. (2006)^[271]^ | Waist Mounted Triaxial Accelerometer | 80-86 yrs. | 6 | M = 2  F = 4 | Accelerometer determined activity classification (using Gaussian mixture models)– Direct Observation  Accelerometer determined activity classification (using Heuristic System)– Direct Observation  A range of free-living activities including sit-to-stand transitions and ambulatory activities (Structured (Elderly)). | Mean accuracy = 91.3%  Mean accuracy = 71.1% | Cheung (2011) |
| Aminian et al. (1999)^[272]^ | Physilog | NA | 5 | M = 4  F = 1 | Phsyilog determined activity classification – Video Observation  1 hour in a studio like room completing specific tasks (sitting/standing and dynamic activities (Structured). | Accuracy = 89.3% | Cheung (2011) |
| Aminian et al. (1999)^[272]^ | Physilog | NA | 5 | M = 4  F = 1 | Physilog determined sitting, lying, standing and locomotion – Direct Observation  1 hour of monitoring of normal daily activities in laboratory room (Structured). | Mean_ERROR_=10.7% | Lowe (2014) |
| Assah et al. (2010)^[274]^ | ActiHeart | 34.2±7.3 yrs. | 35 | M = 16  F = 1 | ActiHeart determined PAEE – Doubly Labelled Water determined TEE, PAL and PAEE  7 continuous days of monitoring from both DLW and ActiHeart (Unstructured). | r=0.32-0.54; p<0.05  Bias = -32.5 -5.0 | Bonomi (2012) |
| Assah et al. (2009)^[273]^ | ActiGraph GT1M | 34.2 ± 7.3 yrs. | 33 | M = 16  F = 17 | ActiGraph determined energy expenditure (KJ.kg^-1^.d^-1^) (from developed regression equation 1) – Doubly Labelled Water determined energy expenditure (KJ.kg^-1^.d^-1^).  ActiGraph determined energy expenditure (KJ.kg^-1^.d^-1^) (from developed regression equation 2) – Doubly Labelled Water determined energy expenditure (KJ.kg^-1^.d^-1^).  ActiGraph determined energy expenditure (KJ.kg^-1^.d^-1^) (from developed regression equation 3) – Doubly Labelled Water determined energy expenditure (KJ.kg^-1^.d^-1^).  ActiGraph determined energy expenditure (KJ.kg^-1^.d^-1^) (from developed regression equation 4) – Doubly Labelled Water determined energy expenditure (KJ.kg^-1^.d^-1^).  ActiGraph determined energy expenditure (KJ.kg^-1^.d^-1^) (from developed regression equation 5) – Doubly Labelled Water determined energy expenditure (KJ.kg^-1^.d^-1^).  ActiGraph determined energy expenditure (KJ.kg^-1^.d^-1^) (from developed regression equation 6) – Doubly Labelled Water determined energy expenditure (KJ.kg^-1^.d^-1^).  ActiGraph determined energy expenditure (KJ.kg^-1^.d^-1^) (from developed Hendelman equation) – Doubly Labelled Water determined energy expenditure (KJ.kg^-1^.d^-1^).  ActiGraph determined energy expenditure (KJ.kg^-1^.d^-1^) (from developed Swartz equation) – Doubly Labelled Water determined energy expenditure (KJ.kg^-1^.d^-1^).  ActiGraph determined energy expenditure (KJ.kg^-1^.d^-1^) (from developed Freedson equation) – Doubly Labelled Water determined energy expenditure (KJ.kg^-1^.d^-1^).  ActiGraph determined energy expenditure (KJ.kg^-1^.d^-1^) (from developed Hendelman equation) – Doubly Labelled Water determined energy expenditure (KJ.kg^-1^.d^-1^).  ActiGraph determined energy expenditure (KJ.kg^-1^.d^-1^) (from developed Swartz equation) – Doubly Labelled Water determined energy expenditure (KJ.kg^-1^.d^-1^).  ActiGraph determined energy expenditure (KJ.kg^-1^.d^-1^) (from developed Freedson equation) – Doubly Labelled Water determined energy expenditure (KJ.kg^-1^.d^-1^)  ActiGraph worn for 7 days during the 7 days of DLW measurement (Unstructured). | Bias = -0.67 (5.34); RMSE = 30.22; 95% LoA = -60.8 – 59.47.  Bias = -0.15 (5.41); RMSE = 30.61; 95% LoA = -61.1 – 60.8.  Bias = -0.33 (5.24); RMSE = 29.64; 95% LoA = -59.3 – 58.7.  Bias = 0.23 (5.39); RMSE = 29.66; 95% LoA = -58.8 – 59.26.  Bias = -0.65 (5.09); RMSE = 28.8; 95% LoA = -57.9 – 56.7.  Bias = -0.05 (5.08); RMSE = 28.76; 95% LoA = -57.3 – 57.2.  Bias = 23.45 (6.34); RMSE = 42.86; 95% LoA = -47.95 – 94.86.  Bias = 23.28 (6.08); RMSE = 41.52; 95% LoA = -45.15 – 91.71.  Bias = -6.29 (5.56); RMSE = 32.06; 95% LoA = -68.85 – 56.27.  Bias = -9.09 (5.52); RMSE = 32.54; 95% LoA = -53.11 – 71.28.  Bias = -8.91 (5.35); RMSE = 31.55; 95% LoA = -51.33 – 69.15.  Bias = -20.66 (4.98); RMSE = 34.92; 95% LoA = -76.7 – 35.38. | Plasqui (2013) |
| Bonomi et al. (2009)^[275]^ | Tracmor | 29±6 yrs. | 20 | M = 13  F = 7 | Tracmor determined activity classification (decision trees) – Direct observation of physical activities.  Series of structured free-living activities (Structured). | Accuracy = 59-100% (Standing lowest level of accuracy) | Bonomi (2012) |
| Bonomi et al. (2009)^[276]^ | Tracmor | 26-59 yrs. | 15 | M = 9  F = 6 | Accelerometer determined activity counts (PAL regression Model) – Doubly Labelled Water determined PAL.  Accelerometer determined METs/d (PAL regression Model) – Doubly Labelled Water determined PAL.  Accelerometer determined activity counts (TEE regression Model) – Doubly Labelled Water determined TEE.  Accelerometer determined METs/d (TEE regression Model) – Doubly Labelled Water determined TEE.  Accelerometer determined activity counts (AEE regression Model) – Doubly Labelled Water determined AEE.  Accelerometer determined METs/d (AEE regression Model) – Doubly Labelled Water determined AEE.  14 days of doubly labelled water monitoring compared with 5 days of activity monitoring (unstructured). METs determined by classification of activity from decision tree, and applying a mean MET value (compendium) to this activity classification (Unstructured). | R^2^=0.46 (r=0.68; p<0.05)  R^2^=0.51  (r=0.71; p<0.05)  R^2^=0.85 (r=0.92)  R^2^=0.87  (r=0.93)  R^2^=0.47 (r=0.68)  R^2^=0.60  (r=0.77) | Butte (2012) |
| Bonomi et al. (2010)^[277]^ | DirectLife triaxial accelerometer (New Tracmor) | 41±11 yrs. | 30 | M = 18  F = 12 | DirectLife determined activity counts per day – Doubly labelled water determined TEE and AEE.  15 consecutive days monitoring of free-living activity behaviours (Unstructured). | R^2^=0.76 (based on sleeping metabolic rate and counts/day)  r_AEE_=0.54; p<0.01  r_TEE_=0.68; p<0.01 | Bonomi (2012) |
| Busse et al. (2009)^[118]^ | Step Activity Monitor | 22-39 yrs. | 18 | NA | SAM determined steps – Direct Observation determined steps (Indoor).  SAM determined steps – Direct Observation determined steps (Indoor).  Participants wore the SAM and had steps directly observed at both an indoor and outdoor circuit (Structured). | %_ACCURACY_=96.1%; Mean_DIFF_=5.76; LoA = -4.6 - 16.2; %_DIFF_=5.18%  %_ACCURACY_=99.6%; Mean_DIFF_=2.82; LoA = -12.2 – 17.8; %_DIFF_=7.47% | Shephard (2012) |
| Bussman et al. (1998)^[278]^ | 4 x IC3031 uniaxial accelerometers | 19-24 yrs. | 3 | M = 3 | Accelerometer determined activity classification – video recorded direct observation.  4 hours of video recording in a living room space (Classification of Lying Back, Side and Prone, Standing, Sitting and Dynamic (Unstructured). | % agreement =88%  (Range = 58 – 100%) | Bonomi (2012) |
| Carter et al. (2008)^[279]^ | 3dNX^TM^ accelerometer | 19 ± 3 yrs. | 14 | M = 14 | 3dNX^TM^  determined total energy expenditure (regression model including only activity counts per day) – Doubly Labelled Water determined total energy expenditure  3dNX^TM^  determined total energy expenditure (regression model including activity counts per day and height) – Doubly Labelled Water determined total energy expenditure  3dNX^TM^  determined total energy expenditure (regression model including activity counts per day and fat free mass) – Doubly Labelled Water determined total energy expenditure  3dNX^TM^  determined activity energy expenditure (regression model including activity counts per day and fat free mass) – Doubly Labelled Water determined activity energy expenditure  3dNX^TM^  worn for 10 days compared with DLW examined for 14 days (Unstructured). | R^2^=0.35; p=0.05; SEE = 1519 kJ.day^-1^.  R^2^=0.73; p<0.05; SEE = 1027 kJ.day^-1^.  R^2^=0.69; p<0.05; SEE = 1092 kJ.day^-1^.  R^2^=0.33; p<0.05; SEE = 950 kJ.day^-1^. | Liu (2012) |
| Choquette et al (2009)^[280]^ | Caltrac | 60-78 yrs. | 17 | M=10  F=7 | Caltrac determined total energy expenditure (kcal/d^-1^) – Doubly Labelled Water determined energy expenditure (kcal/d^-1^).  Participants wore the Caltrac for 7 days and had DLW examined for the same period (Unstructured). | r_s_=0.37; NS | Van Remoortel (2012) |
| Colbert et al. (2011)^[76]^ | ActiGraph GT1M | 74.7 ± 6.5 yrs. | 56 | M = 12  F = 44 | ActiGraph GT1M determined PAEE (Crouter equation) - Doubly labelled water determined PAEE (kcal.d^-1^).  ActiGraph GT1M determined PAEE (Freedson equation) - Doubly labelled water determined PAEE (kcal.d^-1^).  Participants wore the devices during the 15 days of DLW measurement and completed the questionnaire on day 8 and day 15 of the study (Unstructured). | RMSE = 185; MAPE = 22.5; r_s_ = 0.60; p<0.01  RMSE = 202; MAPE = 24.4; r_s_ = 0.49; p<0.01 | Plasqui (2013) |
| Colbert et al. (2011)^[76]^ | Sensewear Pro Armband | 74.7 ± 6.5 yrs. | 56 | M = 12  F = 44 | SWPA determined PAEE (kcal.d^-1^) – Doubly labelled water determined PAEE (kcal.d^-1^).  Participants wore the devices during the 15 days of DLW measurement and completed the questionnaire on day 8 and day 15 of the study (Unstructured) | RMSE = 210; MAPE = 26.8; r_s_ = 0.48; p<0.01 | Plasqui (2013) |
| Conger et al. (2005)^[281]^ | Fitsense FS1 (Speedometer) | 27.2±5.7 yrs. | 24 | M = 15  F = 9 | Fitsense determined distance walked /run – Direct Observation  Walking 1600 metres on a track (Structured). | No difference with measured walking (p>0.05)  Underestimated running (p<0.05) | Chen (2005) |
| Connolly et al. (2011)^[282]^ | ActiGraph GT3X | 30.6 ± 5.6 yrs. | 30 | F=30 | AG determined steps – Direct Observation determined steps.  Participants walked on a treadmill for 8-13 minutes wearing all devices and steps were recorded using direct observation (Structured). | %Accuracy=86.9 ± 16.2% | Van Remoortel (2012) |
| Dijkstra et al. (2010)^[283]^ | Dynaport Minimod | 68.5±7.4 yrs. | 20 | NA | Minimod determined physical activity classification – Direct observation  Laboratory based sitting, standing, lying, walking and 3 minutes of free-living ambulation (structured).  30 minutes of home based free-living activities (while also completing similar activities as completed in the lab) (Structured). | Sensitivity = 49.4 – 99.8%  Specificity = 71.8 – 91.2%  Predictive Value = 49.7 - 97.6 | Cheung (2011) |
| Dugas et al. (2009)^[284]^ | Actical | M: 26.5 ± 3.1 yrs.  F: 23.5 ± 3.3 yrs. | 28 | M = 8  F = 20 | Actical determined total energy expenditure (MJ.d^-1^) – Doubly Labelled Water determined total energy expenditure (MJ.d^-1^).  Actical and DLW measurements completed for a two week period (Unstructured). | r=0.57 | Van Remoortel (2012) |
| Ermes et al. (2008)^[285]^ | Multiple body worn Sensors (Hip, Wrist worn accelerometers and GPS device. | 27.1±9.2 yrs. | 12 | M = 10  F = 2 | Combined sensor determined activity type – Direct Observation  6 hours of “training activities” for model development, including both structured and unstructured activities. Models developed using only structured activities and both structured and unstructured activities. | Accuracy (using both supervised and unsupervised activities) = 89%  Accuracy (using only supervised activities) = 72% | Cheung (2011) |
| Esliger et al. (2007)^[286]^ | Actical | 9-59 yrs. | 38 | M = 16  F = 22 | Actical determined steps (at 50 m.min^-1^) – Direct observation determined steps (at 50 m.min^-1^).  Actical determined steps (at 83 m.min^-1^) – Direct observation determined steps (at 83 m.min^-1^).  Actical determined steps (at 133 m.min^-1^) – Direct observation determined steps (at 133 m.min^-1^).  Participants wore Actical and ActiGraph while walking on a treadmill at 3 different speeds while being video recorded (Structured). | Mean_DIFF_=-7 ± 7.4 steps; p<0.001; r=0.73  Mean_DIFF_=-1 ± 0.008 steps; r=0.99  Mean_DIFF_= 1 ± 0.006 steps; r=0.99 | Murphy (2009) |
| Esliger et al. (2007)^[286]^ | ActiGraph | 9-59 yrs. | 38 | M = 16  F = 22 | ActiGraph determined steps (at 50 m.min^-1^) – Direct observation determined steps (at 50 m.min^-1^).  ActiGraph determined steps (at 83 m.min^-1^) – Direct observation determined steps (at 83 m.min^-1^).  ActiGraph determined steps (at 133 m.min^-1^) – Direct observation determined steps (at 133 m.min^-1^).  Participants wore Actical and ActiGraph while walking on a treadmill at 3 different speeds while being video recorded (Structured). | Mean_DIFF_=-5 ± 5.3 steps; p<0.01; r=0.52  Mean_DIFF_=-1 ± 0.008 steps; r=0.99  Mean_DIFF_= 1 ± 0.006 steps; r=0.99 | Murphy (2009) |
| Fuller et al. (2008)^[79]^ | Caltrac | 20-66 yrs. | 60 | M = 30  F = 30 | Caltrac determined energy expenditure (MJ.d^-1^) – Doubly Labelled Water determined energy expenditure (MJ.d^-1^)  15 days of DLW measurement, HRM, Physical Activity Diary, Caltrac device and 2 versions of the 7DPARQ (Day 8 and day 15 (Unstructured). | r=0.59 | Plasqui (2013) |
| Fuller et al. (2008)^[79]^ | Caltrac | 20-66 yrs. | 60 | M = 30  F = 30 | Caltrac determined energy expenditure (PAL) – Doubly Labelled Water determined energy expenditure (PAL)  15 days of DLW measurement, HRM, Physical Activity Diary, Caltrac device and 2 versions of the 7DPARQ (Day 8 and day 15 (Unstructured). | r=0.43 | Plasqui (2013) |
| Godfrey et al. (2007)^[287]^ | activPAL | 24.9 ± 1.7 yrs. | 10 | M = 7  F = 3 | activPAL determined sit/lie – Direct Observation.  activPAL determined stand – Direct Observation.  activPAL determined stepping – Direct Observation.  Both devices worn during 6 hours of free-living physical activity (Unstructured). | %Accuracy_SIT_ = 98%  %Accuracy_SIT_ = 98%  %Accuracy_SIT_ = 98% | Lowe (2014) |
| Grant et al. (2006)^[289]^ | activPAL | 43±10.6 yrs. | 10 | M = 4  F = 6 | activPAL determined activity classification – Video recorded direct observation.  Common activities of daily living and controlled activities (e.g. sitting, standing and treadmill walking) in a controlled laboratory (structured). | Mean%Diff_Sitting_=0.19%  Mean%Diff_Upright_= -0.27%  Mean%Diff_Standing_=1.4%  Mean%Diff_Stepping_= -2.0% | Cheung (2011) |
| Grant et al. (2008) ^[288]^ | activPAL | 65-87 yrs. | 21 | M = 10  F = 11 | activPAL determined step count – Direct Observation determined step count  Walking on a treadmill at 5 different speeds and over ground walking on an outdoor track (Structured). | Mean_DIFF_=2.6 (LOA = -3.1 – 8.2).  Absolute % Error <1% | Kowlaski (2012) |
| He et al. (2013)^[290]^ | Smartphone application | 25±5 yrs. | 10 | M = 6  F = 4 | Chest worn smartphone utilizing triaxial accelerometer, gyroscope and orientation sensor – Direct Observation.  Wide range of laboratory based structured activities (Structured). | Accuracy of recognition = 95.03% | Bort Ruig (2014) |
| Jacobi et al. (2007)^[291]^ | RT3 accelerometer | 38.3±10.5 yrs. | 13 | F = 13 | RT3 accelerometer determined energy expenditure (kcal/d) – Doubly Labelled Water determined energy expenditure (kcal/d)  14 days of free-living activity behaviours in overweight/obese individuals (Unstructured). | r=0.55; p<0.05  Relative_DIFF_=-17.1 ± 16.7% | Corder (2007) |
| Jacobi et al. (2007)^[291]^ | Tritrac R3D accelerometer | 38.3±10.5 yrs. | 13 | F = 13 | Tritrac R3D determined energy expenditure (kcal/d) – Doubly Labelled Water determined energy expenditure (kcal/d)  14 days of free-living activity behaviours in overweight/obese individuals (Unstructured). | Relative_DIFF_ =-20.0 ± 44.6% | Corder (2007) |
| Johannsen et al. (2010)^[292]^ | Sensewear Pro Armband | 24-60 yrs. | 30 | M = 15  F = 15 | Sensewear Pro Armband determined total energy expenditure and physical activity energy expenditure – Doubly Labelled water determined total energy expenditure and physical activity energy expenditure  14 days of consecutively measured free-living physical activity (Unstructured). | 8.1% - 26% | Bonomi (2012) |
| Johannsen et al. (2010)^[292]^ | Sensewear Mini Armband | 24-60 yrs. | 30 | M = 15  F = 15 | Sensewear Mini Armband determined total energy expenditure and physical activity energy expenditure – Doubly Labelled water determined total energy expenditure and physical activity energy expenditure  14 days of consecutively measured free-living physical activity (Unstructured). | 8.3% - 28% | Bonomi (2012) |
| Karantonis et al. (2006)^[293]^ | Triaxial Accelerometer (make and model NA) | 22-60 yrs. | 6 | NA | Accelerometer determined activity classification – Direct Observation.  A range of free-living activities including sit-to-stand transitions and ambulatory activities (Structured). | Accuracy = 90.8% | Cheung (2011) |
| Koehler et al. (2011)^[294]^ | Sensewear Pro Armband | 30.4 ± 6.2 yrs. | 14 | M = 14 | SPA determined total energy expenditure - Doubly Labelled Water determined total energy expenditure.  Participants wore the SPA for 7 days while DLW was measured for 7 days (Unstructured). | r=0.73; p<0.01; Mean_DIFF_=65 ± 665 kcal.d^-1^; 95% LoA=-1368 – 1238 kcal.d^-1^ | Shephard (2012) |
| Kumahara et al. (2004)^[295]^ | LifeCorder | 40 ± 12 yrs. | 79 | M = 28  F = 51 | LifeCorder determined TEE (Kj/day) – Doubly Labelled water determined TEE (Kj/day).  LifeCorder determined PAEE (Kj/day) – Doubly Labelled water determined PAEE (Kj/day).  Accelerometer data collected on second week of DLW measurement during free-living activity behaviours (Unstructured). | Mean_DIFF_=-702 Kj/d (-8.1%)  r=0.93; p<0.001  Mean_DIFF_=-238 Kj/d (-7.3%)  r=0.56; p<0.001 | Corder (2007) |
| Kwon et al. (2010)^[296]^ | IDEEA activity monitor | 42.5 ± 13.0 yrs. | 12 | F=12 | IDEEA determined physical activity type –Direct Observation determined physical activity type (normal weight).  IDEEA determined physical activity type –Direct Observation determined physical activity type (obese weight).  Participants performed a range of activities while wearing the IDEEA and being observed by an investigator (Structured). | %Accuracy = 92.7%  %Accuracy = 82.3% | Van Remoortel (2012) |
| Lee et al. (2011)^[321]^ | I-Pod | M; 27.4 ± 3.5 yrs.  F; 34 yrs. | 6 | M = 5  F = 1 | I-pod touch worn in the trouser pocket and on the waist with classification of activities using triaxial accelerometer (using Fuzzy cluster algorithm) – Direct observation.  Range of free-living activities on outdoor track (Structured). | Accuracy =98%  Accuracy_Waist_=99.6%  Accuracy_Pocket_=99.1% | Bort Ruig (2014) |
| Leenders et al. (2006)^[299]^ | TriTrac R3D | 25.8 ± 1.6 yrs. | 13 | F=13 | TriTrac R3D determined total daily energy expenditure (Chen regression equation 1) – Doubly Labelled Water determined total daily energy expenditure.  TriTrac R3D determined total daily energy expenditure (Chen regression equation 2) – Doubly Labelled Water determined total daily energy expenditure.  TriTrac R3D determined total daily energy expenditure (Hendelman outdoors) – Doubly Labelled Water determined total daily energy expenditure.  TriTrac R3D determined total daily energy expenditure (Hendelman lifestyle activities) – Doubly Labelled Water determined total daily energy expenditure.  TriTrac R3D determined total daily energy expenditure (Nichols treadmill walking) – Doubly Labelled Water determined total daily energy expenditure.  TriTrac R3D determined total daily energy expenditure (Leenders treadmill walking) – Doubly Labelled Water determined total daily energy expenditure.  Participants wore the TriTrac for 7 days during a 7 day protocol for DLW (Unstructured). | %_DIFF_=-23.5 ± 5%; r_p_=0.652; CCC=0.342.  %_DIFF_=12.0 ± 3%; r_p_=0.668; CCC=0.456.  %_DIFF_=-10.0 ± 4%; r_p_=0.615; CCC=0.418.  %_DIFF_=101 ± 9%; r_p_=0.508; CCC=0.035.  %_DIFF_=-8.0 ± 4%; r_p_=0.610; CCC=0.445.  %_DIFF_=16 ± 5%; r_p_=0.579; CCC=0.383. | Van Remoortel (2012) |
| Leenders et al. (2006)^[299]^ | ActiGraph | 25.8 ± 1.6 yrs. | 13 | F=13 | ActiGraph determined total daily energy expenditure (Freedson treadmill walking) – Doubly Labelled Water determined total daily energy expenditure.  ActiGraph determined total daily energy expenditure (Hendelman outdoors) – Doubly Labelled Water determined total daily energy expenditure.  ActiGraph determined total daily energy expenditure (Hendelman lifestyle activities) – Doubly Labelled Water determined total daily energy expenditure.  ActiGraph determined total daily energy expenditure (Swaartz lifestyle activities) – Doubly Labelled Water determined total daily energy expenditure.  ActiGraph determined total daily energy expenditure (Nichols treadmill walking) – Doubly Labelled Water determined total daily energy expenditure.  ActiGraph determined total daily energy expenditure (Leenders treadmill walking) – Doubly Labelled Water determined total daily energy expenditure.  ActiGraph determined total daily energy expenditure (Yngve walk/run outdoors) – Doubly Labelled Water determined total daily energy expenditure.  ActiGraph determined total daily energy expenditure (Yngve walk/run treadmill) – Doubly Labelled Water determined total daily energy expenditure.  Participants wore the ActiGraph for 7 days during a 7 day protocol for DLW (Unstructured). | %_DIFF_=-21.0 ± 3%; r_p_=0.672; CCC=0.220.  %_DIFF_=-21.0 ± 3%; r_p_=0.667; CCC=0.211.  %_DIFF_=-2.0 ± 5%; r_p_=0.556; CCC=0.495.  %_DIFF_= -4 ± 5%; r_p_=0.407; CCC=0.350.  %_DIFF_=-20.0 ± 4%; r_p_=0.650; CCC=0.291.  %_DIFF_= -10 ± 5%; r_p_=0.616; CCC=0.384.  %_DIFF_=-32.0 ± 3%; r_p_=0.631; CCC=0.106.  %_DIFF_= -25 ± 3%; r_p_=0.654; CCC=0.162. | Van Remoortel (2012) |
| Le Masurier et al. (2004)^[297]^ | ActiGraph (CSA) | 20-55 yrs. | 12 | M = 6  F = 6 | ActiGraph steps – Direct Observation  Treadmill walking at 5 different speeds (Structured). | Diff_Lab_>0.05 | Berlin (2006) |
| Le Masurier et al. (2003)^[298]^ | ActiGraph (CSA) | 20-55 yrs. | 20 | M = 13  F = 7 | ActiGraph steps – Direct Observation  Treadmill walking at 5 different speeds (Structured). | Diff_Lab_>0.05 all activities | Berlin (2006) |
| Levine et al. (2008)^[300]^ | Physical Activity Monitor (PAMS) | NA | NA | NA | Posture determined from the PAMS – Direct observation  Structured laboratory based activities (Structured). | Accuracy = 100% | Bonomi (2012) |
| Liu et al. (2009)^[301]^ | 3 triaxial accelerometers (chest, waist and thigh) | 21.3±1.1 yrs. | 10 | M = 5  F = 5 | Triaxial accelerometer determined activity classification (SONFIN models) – Direct observation.  Completed walking, sitting down and falling activities (Structured). | Total classification accuracy (cross-validation) = 88.7% | Cheung (2011) |
| Liu et al. (2009)^[301]^ | 3 triaxial accelerometers (chest, waist and thigh) | 68-86 yrs. | 7 | M = 5  F = 2 | Triaxial accelerometer determined activity classification (SONFIN models) – Direct observation.  Completed walking, sitting down and falling activities (Structured). | Total classification accuracy (cross-validation) = 80.4% | Cheung (2011) |
| Lof et al. (2011)^[302]^ | IDEEA | Pregnant: 32 ± 4 yrs.  Non-Pregnant: 36 ± 8 yrs. | 18  21 | F=18  F=21 | IDEEA determined total energy expenditure (MJ.d^-1^) – Doubly Labelled Water determined total energy expenditure (MJ.d^-1^) (Pregnant only).  IDEEA determined total energy expenditure (MJ.d^-1^) – Doubly Labelled Water determined total energy expenditure (MJ.d^-1^) (Non-pregnant only).  Participants wore the IDEEA for 5 days and had DLW measurement completed for 14 days (Unstructured). | Mean_DIFF_=-1.0 ±.1 MJ.d^-1^; r=0.66; p<0.001  Mean_DIFF_=-1.0 ±.1 MJ.d^-1^; r=0.81; p<0.001 | Van Remoortel (2012) |
| Lof et al. (2003)^[303]^ | ActiGraph CSA | 29 ± 4 yrs. | 37 | F = 37 | Physical activity level as determined by CSA – Doubly labelled water determined physical activity level.  Total energy expenditure as determined by CSA (KJ.d^-1^) – Doubly labelled water determined total energy expenditure.  4 days of physical activity monitoring from CSA compared to 14 days of DLW measurement (Unstructured). | Mean_DIFF_=-0.27; p<0.05  Mean_DIFF_=-370; NS; LoA = 2930 KJ.d^-1^. | Nielson (2009) |
| Lof et al. (2002)^[95]^ | ActiGraph CSA | 30 ± 4 yrs. | 24 | F = 24 | CSA determined energy expenditure (kcal.d^-1^) – Doubly labelled water determined energy expenditure (kcal.d^-1^).  CSA device worn during the 14 days of DLW assessment (Unstructured). | Mean_DIFF_= 6 kcal; 2SDs = 650kcal.24hrs^-1^. | Plasqui (2007) |
| Mackey et al. (2011)^[322]^ | Sensewear Pro Armband | 82.0 ± 3.3 yrs. | 19 | M=11  F=8 | SPA determined total energy expenditure (kcal.d^-1^) (Version 6.1) – Doubly Labelled Water determined TEE (kcal.d^-1^).  SPA determined total energy expenditure (kcal.d^-1^) (Version 5.1) – Doubly Labelled Water determined TEE (kcal.d^-1^).  SPA determined activity energy expenditure (kcal.d^-1^) (Version 6.1) – Doubly Labelled Water determined AEE (kcal.d^-1^).  SPA determined activity energy expenditure (kcal.d^-1^) (Version 5.1) – Doubly Labelled Water determined AEE (kcal.d^-1^).  Participants wore SPA and participated in DLW measurement for a two week period (Unstructured). | r_p_=0.893  r_p_=0.901  r_p_=0.760; Mean_DIFF_= -108 kcal.d^-1^; p<0.01  r_p_=0.786; Mean_DIFF_= -156 kcal.d^-1^; p<0.05 | Van Remoortel (2012) |
| Maddison et al. (2009)^[304]^ | RT3 accelerometer | 39 ± 10 yrs. | 36 | M = 16  F = 20 | RT3 accelerometer determined total energy expenditure (kJ) – Doubly labelled water determined total energy expenditure (kJ)  RT3 accelerometer determined activity energy expenditure (kJ) - Doubly labelled water determined activity energy expenditure (kJ)  14 days of RT3 data compared with 15 days of DLW measurement (Unstructured). | Mean_DIFF_=539 kJ %_DIFF_=4%  Mean_DIFF_=485 kJ  %_DIFF_=14% | Plasqui (2013) |
| Maddocks et al. (2010)^[305]^ | activPAL | 28 ± 8 yrs. | 40 | M = 15  F = 25 | activPAL determined steps – Direct Observation determined steps.  Treadmill Speed 0.6 m.s^-1^  Treadmill Speed 0.8 m.s^-1^  Treadmill Speed 1.0 m.s^-1^  Treadmill Speed 1.2 m.s^-1^  Treadmill Speed 1.4 m.s^-1^  Motor vehicle transport  Participants walked on a treadmill at 5 different speeds and on a 500m outdoor course (at 3 different speeds) while wearing the devices and being video observed (Structured). | Mean%_Error_=1.7; 95% CI = 0.5-2.9.  Mean%_Error_=0.7; 95% CI = 0.3-1.2.  Mean%_Error_=1.4; 95% CI = 0.9-1.9.  Mean%_Error_=1.8; 95% CI = 0.9-2.7.  Mean%_Error_=0.7; 95% CI = 0.3-1.1.  Mean%_Error_=0 | Tudor-Locke (2012) |
| Maddocks et al. (2010)^[305]^ | PALlite | 28 ± 8 yrs. | 40 | M = 15  F = 25 | PALlite determined steps – Direct Observation determined steps.  Treadmill Speed 0.6 m.s^-1^  Treadmill Speed 0.8 m.s^-1^  Treadmill Speed 1.0 m.s^-1^  Treadmill Speed 1.2 m.s^-1^  Treadmill Speed 1.4 m.s^-1^  Motor vehicle transport  Participants walked on a treadmill at 5 different speeds and on a 500m outdoor course (at 3 different speeds) while wearing the devices and being video observed (Structured). | Mean%_Error_=3.4; 95% CI = 1.8-4.9.  Mean%_Error_=2.9; 95% CI = 0.3-5.5.  Mean%_Error_=1.6; 95% CI = 0.9-2.2.  Mean%_Error_=3.9; 95% CI = 1.5-9.2.  Mean%_Error_=1.1; 95% CI = 0.3-1.8.  Mean_Error_=254 steps | Tudor-Locke (2012) |
| Marsh et al. (2007)^[306]^ | IDEEA | 70-85 yrs. | 29 | M = 9  F = 29 | IDEEA determined step count – Direct Observation determined step count.  Assessment completed during 131 metres of walking around an indoor track (Structured). | %Error = 2.5 ± 3.7  r=0.965; p<0.001 | Kowalski (2012) |
| Mathie et al. (2004)^[307]^ | Waist Mounted Triaxial Accelerometer | 30.5±6.3 yrs. | 26 | M = 7  F = 19 | Accelerometer determined activity classification – Direct Observation.  Range of free-living postural changes and ambulatory activities (Structured). | Sensitivity = 97.7%  Specificity = 98.7% | Cheung (2011) |
| Mathie et al. (2003)^[308]^ | Triaxial Accelerometer | 30.5 ± 6.3 yrs. | 26 | M = 19  F = 7 | Accelerometer determined rest and activity – Direct Observation  13 subjects carried out 11 activities (lasting a total of 8 minutes) while wearing the accelerometer and being observed to develop the algorithm, which was validated on the remaining 13 subjects (Structured). | Sens = 98%  Spec = 88% - 94%. | Lowe (2014) |
| Najafi et al. (2003)^[309]^ | Chest worn kinematic sensor | 66 ± 14 yrs. | 9 | NA | Kinematic sensor determined Standing and Walking (mins.) – Direct Observation.  Older adults worn the device while being observed for 45-60 minutes (Unstructured). | Sens = 92.2%  Spec = 92.1%  Detection Error as percent of range = 3.9% | Lowe (2014) |
| Najafi et al. (2003)^[309]^ | Physilog | 79±6 yrs. | 11 | M = 5  F = 6 | Physilog determined activity classification – Video observation  Performed 6 different activities including sit to stand transitions and walking (Structured (elderly)). | Sensitivity = 63%-100%  Specificity = 63% - 100% | Cheung (2011) |
| Najafi et al. (2003)^[309]^ | Physilog | 66±14 yrs. | 9 | NA | Physilog determined activity classification and posture – Video observation,  Performed free-living activities without external supervision (Unstructured (elderly)). | Sensitivity = 90.2%-98.4%  Specificity = 92.1% - 99.7% | Cheung (2011) |
| Plasqui et al. (2005)^[310]^ | Tracmor | 18-40 yrs. | 29 | M = 10  F = 19 | Tracmor determined activity counts per day (regression analysis examined contribution to model) – Doubly labelled water determined TEE. MJ/d  Tracmor determined activity counts per day (regression analysis examined contribution to model) – Doubly labelled water determined AEE. MJ/d  15 consecutive days monitoring of free-living activity behaviours (Unstructured). | R^2^=0.77  R^2^=0.83  (based on age, body mass, height and activity counts/day). | Bonomi (2012) |
| Preece et al. (2008)^[311]^ | Pegasus Activity Monitors | 31±7 yrs. | 20 | M = 10  F = 10 | Pegasus determined time and frequency of activity classification (waist, thigh and ankle) – Video observation.  8 different activities including walking, jogging, running, stair ascent/descent, hopping and jumping (Structured). | Sensitivity = 64-99%  Specificity = 98-99% | Cheung (2011) |
| Rafamantanantsoa et al. (2003)^[312]^ | Kenz Lifecorder | 50 ± 12 yrs. | 40 | M = 40 | KL determined energy expenditure from high exercise physical activity (mins) – Doubly labelled water determined total energy expenditure (kcal.d^-1^).  Accelerometer worn for 14 days while DLW was measure over the same period (Unstructured). | r=0.28; NS; R^2^=0.51; SEE=263 kcal.d (51% of variability in TEE explained by FFM and HEPA); p<0.001 | Plasqui (2007) |
| Rowlands et al. (2007)^[313]^ | Actigraph | 23.1 ± 3.4 yrs. | 10 | M = 10 | ActiGraph determined steps – Direct Observation  Walking and jogging at 11 speeds on a treadmill. Speeds ranged from 4-26 kmph (Structured). | r=0.88 | Corder (2007) |
| Ryan et al. (2006)^[314]^ | activPAL | 34.5 ± 6.9 yrs. | 20 | M = 8  F = 12 | activPAL determined steps – Direct Observation determined steps.  Treadmill Speed 0.9 m.s^-1^  Treadmill Speed 1.12 m.s^-1^  Treadmill Speed 1.33 m.s^-1^  Treadmill Speed 1.56 m.s^-1^  Treadmill Speed 1.78 m.s^-1^  Outdoor Speed Slow  Outdoor Speed Normal  Outdoor Speed Fast  Participants walked on a treadmill at 5 different speeds and on a 500m outdoor course (at 3 different speeds) while wearing the devices and being video observed (Structured). | Mean_DIFF_=0.94 (95%LoA=0.01-1.87); APE= 0.94 ± 0.47  Mean_DIFF_=0.59 (95%LoA=0.06-1.13) ; APE= 0.59 ± 0.27  Mean_DIFF_=0.50 (95%LoA=-0.18-1.18) ; APE= 0.53 ± 0.28  Mean_DIFF_=0.44 (95%LoA=-0.20-1.08) ; APE= 0.47 ± 0.27  Mean_DIFF_=0.63 (95%LoA=-0.63-1.89) ; APE= 0.66 ± 0.60  Mean_DIFF_=0.06 (95%LoA=-1.05-1.17) ; APE= 0.42 ± 0.37  Mean_DIFF_=0.63 (95%LoA=-0.63-1.89) ; APE= 0.37 ± 0.47  Mean_DIFF_=0.18 (95%LoA=-1.03-1.39) ; APE= 0.47 ± 0.43 | Tudor-Locke (2012) |
| Starling et al. (1999)^[104]^ | Caltrac | 45-84 yrs. | 77 | M = 32  F = 35 | Caltrac determined kcal/d - Doubly Labelled Water energy expenditure (kcal/d).  9 days Caltrac data compared Doubly Labelled Water over a 10 day period (Unstructured). | Mean _DIFF_ = 50-55% | Ainslie (2003) |
| Staudenmayer et al. (2009)^[315]^ | ActiGraph 7164 | 35.0 (21-69) yrs. | 48 | M = 24  F = 24 | ActiGraph determined Activity Type (using Artificial Neural Networks) – Direct Observation.  ActiGraph and Cosmed worn during the completion of activity routines in the laboratory (Structured). | Accuracy = 88.8% (95% CI = 86.4 – 91.2) | Liu (2012) |
| St-Onge et al. (2007)^[316]^ | HealthWear Bodymedia portable armband | 35.1 ± 14.0 yrs. | 45 | M = 13  F = 32 | Bodymedia portable armband determined daily energy expenditure (kcal.d^-1^) – Doubly Labelled Water determined daily energy expenditure (kcal.d^-1^).  DLW was determined for 10 days compared against data from the armband measured for 10 days (Unstructured). | Mean_DIFF_=-225 kcal.d^-1^ (SEE=±179); p<0.01; r^2^=0.74; p<0.01 | Van Remoortel (2012) |
| Storti et al. (2008)^[317]^ | ActiGraph accelerometer | 79.2 ±6.0 yrs. | 34 | M = 10  F = 24 | AG determined steps – Direct Observation determined steps.  Participants wore the devices while completing a 100 step test (Structured). | %_DIFF_=6.9%; Mean Ab_ERROR_= 8.7 ± 12.0 | Yang (2010) |
| Wu et al. (2012)^[318]^ | i-Pod touch | 19-60 yrs. | 16 | M = 6  F = 10 | Armband worn I-pod touch classification utilizing triaxial accelerometer and gyroscope – Direct observation.  13 activities to simulate free-living conditions (Structured). | Weighted average = 90.2 %  Range = 52.3% - 100% | Bort Ruig (2014) |
| Yang et al. (2008)^[319]^ | MMA7260Q triaxial accelerometer | 24.1±1.8 yrs. | 7 | M = 3  F = 4 | Triaxial accelerometer determined activity classification (using Proposed Neural Classifier and k-nearest neighbour method (models/algorithms) – Direct Observation.  Eight common domestic activities in a controlled laboratory (Structured). | Accuracy PNC = 95%  Accuracy KNN = 87% | Cheung (2011) |
| Zhang et al. (2003)^[320]^ | IDEEA | 36.3 ± 14.9 yrs. | 76 | M = 33  F = 43 | Activity classification from IDEEA – Direct Observation  Locomotive activities only included here, walking at different speeds, stair ascending and descending (Structured). | r=0.986; p<0.0001 | Westerterp (2009) |

AG = ActiGraph; DLW = Doubly Labelled Water; 24PARQ = 24 Hour Physical Activity Recall Questionnaire; SAM = Step Activity Monitor; HRM = Heart Rate Monitor; 7DPARQ = 7 Day Physical Activity Recall Questionnaire; SPA = Sensewear Pro Armband; PAMS = Physical Activity Monitor; CSA = Computer Science Applications accelerometer; PNC = Proposed Neural Classifier; KNN = k-nearest neighbour method; PA = Physical Activity; PAEE = Physical Activity Energy Expenditure; TEE = Total Energy Expenditure; AEE = Activity Energy Expenditure; PAL = Physical Activity Level; M= Male; F = Female; r = Correlation Coefficient; NS = Non-significant; RMSE = Root Mean Squared Error; LoA = Limits of Agreement; CI = Confidence Intervals; MeanDIFF = Mean Difference; %DIFF = Percentage Difference; SEE = Standard Error of the Estimate; MAPE = Mean Absolute Percentage Error; rp = Pearson’s Correlation.

**Supplementary Table 7.** Concurrent validity of accelerometer/activity monitor determined physical activity/energy expenditure.

| **Author** | **Measure** | **Age Range** | **Sample Size** | **Sex** | **Details** | **Results** | **Primary Source** |
| --- | --- | --- | --- | --- | --- | --- | --- |
| Abel et al. (2008)^[270]^ | ActiGraph GT1M | 29.4 ± 7.1 yrs. | 20 | M = 10  F = 10 | AG determined activity counts – Indirect Calorimetry determined activity energy expenditure (@ 54 m.min^-1^).  AG determined activity counts – Indirect Calorimetry determined activity energy expenditure (@ 80 m.min^-1^).  AG determined activity counts – Indirect Calorimetry determined activity energy expenditure (@ 107 m.min^-1^).  AG determined activity counts – Indirect Calorimetry determined activity energy expenditure (@ 134 m.min^-1^).  AG determined activity counts – Indirect Calorimetry determined activity energy expenditure (@ 161 m.min^-1^).  AG determined activity counts – Indirect Calorimetry determined activity energy expenditure (@ 188 m.min^-1^).  AG determined total energy expenditure (Freedson Equations) – Indirect Calorimetry determined activity energy expenditure (@ 54 m.min^-1^).  AG determined total energy expenditure (Freedson Equations) – Indirect Calorimetry determined activity energy expenditure (@ 80 m.min^-1^).  AG determined total energy expenditure (Freedson Equations) – Indirect Calorimetry determined activity energy expenditure (@ 107 m.min^-1^).  AG determined total energy expenditure (Freedson Equations) – Indirect Calorimetry determined activity energy expenditure (@ 134 m.min^-1^).  AG determined total energy expenditure (Freedson Equations) – Indirect Calorimetry determined energy expenditure (@ 161 m.min^-1^).  AG determined total energy expenditure (Freedson Equations) – Indirect Calorimetry determined energy expenditure (@ 188 m.min^-1^).  Participants completed 6 treadmill trials at 54, 80, 107, 134, 161 and 188 m.min^-1^ while wearing the devices (Structured). | Underestimated; p<0.001; r=-0.23; NS  Underestimated; p<0.001; r=0.12; NS  Underestimated; p<0.001; r=0.31; NS  Underestimated; p<0.001; r=0.36; NS  Underestimated; p<0.001; r=0.53; p<0.05  Underestimated; p<0.001; r=0.40; NS  NS diff; r=0.82; p<0.05  NS diff; r=0.91; p<0.05  NS diff; r=0.93; p<0.05  NS diff; r=0.84; p<0.05  Underestimated; p<0.001; r=0.90; p<0.05  Underestimated; p<0.001; r=0.84; p<0.05 | Shephard (2012) |
| Adams et al. (2005)^[72]^ | ActiGraph 7164 | 49.1 ± 6.8 yrs. | 81 | F = 81 | ActiGraph determined counts.min^-1^.d^-1^ - Doubly labelled water determined energy expenditure (kcal.kg^-1^.d^-1^)  14 days of DLW and ActiGraph measurement compared to 1 day of PA recall from 24PARQ (Unstructured). | r=0.30; p<0.05 | Nielson (2009) |
| Anderson et al. (2007)^[323]^ | Smartphone | 28-54 yrs. | 9 | M = 4  F = 5 | Smartphone determined activity classification (Artificial Neural Networks and Hidden Markov Models) – Self-reported diary determined activity classification.  In built accelerometer and gyroscope. | Accuracy_ANN_ =73% - 87%  Accuracy_HMM_ =74% - 92% | Bort Ruig (2014) |
| Atallah et al. (2011)^[324]^ | Miniaturised ear worn sensor | 29.96 ± 4.53 yrs. | 25 | M = 18  F = 7 | Ear worn sensor determined predicted METs – Indirect Calorimetry determined actual METs (lying down).  Ear worn sensor determined predicted METs – Indirect Calorimetry determined actual METs (standing).  Ear worn sensor determined predicted METs – Indirect Calorimetry determined actual METs (computer work).  Ear worn sensor determined predicted METs – Indirect Calorimetry determined actual METs (vacuuming).  Ear worn sensor determined predicted METs – Indirect Calorimetry determined actual METs (vacuuming).  Ear worn sensor determined predicted METs – Indirect Calorimetry determined actual METs (slow walk 5 km.h^-1^).  Ear worn sensor determined predicted METs – Indirect Calorimetry determined actual METs (brisk walk 6.2 km.h^-1^).  Ear worn sensor determined predicted METs – Indirect Calorimetry determined actual METs (brisk walk 9.5 km.h^-1^).  Ear worn sensor determined predicted METs – Indirect Calorimetry determined actual METs (brisk walk 12 km.h^-1^).  Ear worn sensor determined predicted METs – Indirect Calorimetry determined actual METs (cycle).  Ear worn sensor determined predicted METs – Indirect Calorimetry determined actual METs (rowing). | Mean_DIFF_=0.01; 95%CI=-0.10-0.11  Mean_DIFF_=-0.02; 95%CI=-0.10-0.06  Mean_DIFF_=-0.04; 95%CI=-0.12-0.04  Mean_DIFF_=-0.17; 95%CI=-0.37-0.03  Mean_DIFF_=-0.02; 95%CI=-0.56-0.52  Mean_DIFF_=0.01; 95%CI=-0.21-0.22  Mean_DIFF_=0.04; 95%CI=-0.18-0.27  Mean_DIFF_=0.14; 95%CI=-0.32-0.60  Mean_DIFF_=-0.35; 95%CI=-1.05-0.34  Mean_DIFF_=0.32; 95%CI=-0.15-0.79  Mean_DIFF_=0.10; 95%CI=-0.38-0.57 | Shephard (2012) |
| Ayen and Montoye (1988)^[325]^ | 3 Caltrac devices worn on the waist (to signify Triaxial device) | 22-34 yrs. | 30 | M = 15  F = 15 | Three Caltrac devices determined energy expenditure (METs) based on vector magnitude using linear regression analysis for walking and running only) – Indirect Calorimetry determined energy expenditure (METs)  Walking  Running  Bench Stepping  Squat Thrust  Half knee bends  Mopping  Treadmill and ADL completed while wearing the devices and indirect calorimetry collected in the laboratory (Structured). | r=0.70; SEE=0.13  r=0.90; SEE=0.23  r=0.75; SEE=0.26  r=0.86; SEE=0.28  r=0.65; SEE=0.39  r=0.77; SEE=0.12 | Mathie (2004) |
| Balogun et al. (1989)^[327]^ | Caltrac | 18-38 yrs. | 25 | M = 10  F = 15 | Caltrac estimated energy expenditure walking– Indirect Calorimetry  Treadmill walking at 4 different speeds (structured) | r=0.92 | Bassett et al. (2000) |
| Balogun et al. (1988)^[326]^ | Caltrac | NA | 20 | M = 15  F = 5 | Caltrac determined activity counts (waist) – Calcount determined activity counts (waist)  Treadmill walking at 2 different speeds (structured) | r_p_=-0.09 – 0.24 | Haskell (2000) |
| Balogun et al. (1988)^[326]^ | Caltrac | NA | 20 | M = 15  F = 5 | Caltrac determined activity counts (chest) – Calcount determined activity counts (chest)  Treadmill walking at 2 different speeds (structured) | r_p_=0.49; p<0.05 | Haskell (2000) |
| Balogun et al. (1989)^[327]^ | Caltrac | 18-38 yrs. | 25 | M = 10  F = 15 | Caltrac estimated VO2 mL/kg/min walking– Indirect Calorimetry determined VO2 mL/kg/min  Treadmill walking at 4 different speeds (structured) | r=0.76 | Bassett et al. (2000) |
| Bao et al. (2004)^[328]^ | Multiple ADXL210E accelerometers (Analog devices) | 17-48 yrs. | 20 | M = 13  F = 7 | Accelerometer determined activities (using different algorithms) – Self reported physical activity types.  Devices worn for a mean of 54-131 minutes during completion of structured activities (Structured). | Accuracy = 80% | Bonomi (2012) |
| Bassett et al. (2000)^[193]^ | ActiGraph (CSA) | 19-74 yrs. | 81 | M =38  F = 43 | ActiGraph EE (determined using counts: Manufacturer) – Indirect Calorimetry  ActiGraph EE (determined using counts: Freedson) – Indirect Calorimetry  ActiGraph EE (determined using counts: Hendelman) – Indirect Calorimetry)  Completion of selected free living tasks (structured) | r_p_=0.62; p<0.01  r_p_=0.32; p<0.01  r_p_=0.62; p<0.01 | Bassett et al. (2000) |
| Bassett et al. (2000)^[193]^ | Caltrac | 19-74 yrs. | 81 | M =38  F = 43 | Caltrac determined EE – Indirect Calorimetry  Completion of selected free living tasks (structured) | r_p_=0.58; p<0.01 | Bassett et al. (2000) |
| Bassett et al. (2000)^[193]^ | Kenz Select 2 | 19-74 yrs. | 81 | M =38  F = 43 | Kenz Select 2 determined EE – Indirect Calorimetry  Completion of selected free living tasks (structured) | r_p_=0.55; p<0.01 | Bassett et al. (2000) |
| Bassett et al. (2000)^[193]^ | ActiGraph (CSA) | 19-74 yrs. | 81 | M =38  F = 43 | ActiGraph EE (determined using counts: Manufacturer) – ActiGraph EE (determined using counts: Freedson)  Completion of selected free living tasks (structured) | r_p_=0.62; p<0.01 | Bassett et al. (2000) |
| Bassett et al. (2000)^[193]^ | ActiGraph (CSA) | 19-74 yrs. | 81 | M =38  F = 43 | ActiGraph EE (determined using counts: Manufacturer) – Caltrac determined EE  Completion of selected free living tasks (structured) | r_p_=0.82; p<0.01 | Bassett et al. (2000) |
| Bassett et al. (2000)^[193]^ | ActiGraph (CSA) | 19-74 yrs. | 81 | M =38  F = 43 | ActiGraph EE (determined using counts: Manufacturer) – Kenz determined EE  Completion of selected free living tasks (structured) | r_p_=0.78; p<0.01 | Bassett et al. (2000) |
| Bassett et al. (2000)^[193]^ | ActiGraph (CSA) | 19-74 yrs. | 81 | M =38  F = 43 | ActiGraph EE (determined using counts: Freedson) – ActiGraph EE (determined using counts: Hendelman)  Completion of selected free living tasks (structured) | r_p_=0.62; p<0.01 | Bassett et al. (2000) |
| Bassett et al. (2000)^[193]^ | ActiGraph (CSA) | 19-74 yrs. | 81 | M =38  F = 43 | ActiGraph EE (determined using counts: Freedson) – Caltrac determined EE  Completion of selected free living tasks (structured) | r_p_=0.47 p<0.01 | Bassett et al. (2000) |
| Bassett et al. (2000)^[193]^ | ActiGraph (CSA) | 19-74 yrs. | 81 | M =38  F = 43 | ActiGraph EE (determined using counts: Freedson) – Kenz determined EE  Completion of selected free living tasks (structured) | r_p_=0.41; p<0.01 | Bassett et al. (2000) |
| Bassett et al. (2000)^[193]^ | Caltrac | 19-74 yrs. | 81 | M =38  F = 43 | Caltrac determined EE – ActiGraph CSA (determined using counts: Manufacturer)  Completion of selected free living tasks (structured) | r_p_=0.82; p<0.01 | Bassett et al. (2000) |
| Bassett et al. (2000)^[193]^ | Caltrac | 19-74 yrs. | 81 | M =38  F = 43 | Caltrac determined EE – ActiGraph CSA (determined using counts: Freedson)  Completion of selected free living tasks (structured) | r_p_=0.47; p<0.01 | Bassett et al. (2000) |
| Bassett et al. (2000)^[193]^ | Caltrac | 19-74 yrs. | 81 | M =38  F = 43 | Caltrac determined EE – ActiGraph CSA (determined using counts: Hendelman)  Completion of selected free living tasks (structured) | r_p_=0.83; p<0.01 | Bassett et al. (2000) |
| Bassett et al. (2000)^[193]^ | Caltrac | 19-74 yrs. | 81 | M =38  F = 43 | Caltrac determined EE – Kenz  Completion of selected free living tasks (structured) | r_p_=0.88; p<0.01 | Bassett et al. (2000) |
| Bassett et al. (2000)^[193]^ | Kenz Select 2 | 19-74 yrs. | 81 | M =38  F = 43 | Kenz determined EE – ActiGraph CSA (determined using counts: Manufacturer)  Completion of selected free living tasks (structured) | r_p_=0.78; p<0.01 | Bassett et al. (2000) |
| Bassett et al. (2000)^[193]^ | Kenz Select 2 | 19-74 yrs. | 81 | M =38  F = 43 | Kenz determined EE – ActiGraph CSA (determined using counts: Freedson)  Completion of selected free living tasks (structured) | r_p_=0.41; p<0.01 | Bassett et al. (2000) |
| Bassett et al. (2000)^[193]^ | Kenz Select 2 | 19-74 yrs. | 81 | M =38  F = 43 | Kenz determined EE – ActiGraph CSA (determined using counts: Hendelman)  Completion of selected free living tasks (structured) | r_p_=0.78; p<0.01 | Bassett et al. (2000) |
| Bassett et al. (2000)^[193]^ | Kenz Select 2 | 19-74 yrs. | 81 | M =38  F = 43 | Kenz determined EE – Caltrac  Completion of selected free living tasks (structured) | r_p_=0.88; p<0.01 | Bassett et al. (2000) |
| Barreira et al. (2013)^[329]^ | ActiGraph GT3X+ (Default Filter) | 61-82 yrs. | 15 | M = 7  F = 8 | AG determined steps.day^-1^ – NL-1000 pedometer determined steps.day^-1^.  Participants wore thee ActiGraph and pedometer for 7 days in free-living situations (Unstructured). | r=0.80;  Mean_DIFF_=-769 steps/d^-1^.  %Diff = -7.4%  Absolute Diff= 16.0% | Lowe (2014) |
| Barreira et al. (2013)^[329]^ | ActiGraph GT3X+ (Low Frequency Extension) | 61-82 yrs. | 15 | M = 7  F = 8 | AG determined steps.day^-1^ – New Lifestyles-1000 pedometer determined steps.day^-1^.  Participants wore thee ActiGraph and pedometer for 7 days in free-living situations (Unstructured). | r=0.90;  Mean_DIFF_= 8140 steps/d^-1^.  %Diff = 121.9%  Absolute Diff= 121.9% | Lowe (2014) |
| Bernsten et al. (2010)^[331]^ | Sensewear Pro Armband | 24-43 yrs. | 29 | F=29 | SPA determined METs –Indirect Calorimeter determined METs  Participants (pregnant women) wore the SPA while performing 90 minutes of daily activities and wearing the indirect calorimeter (Structured). | Mean_DIFF_= -136 ± 343 KJ; %_DIFF_= 9% | Van Remoortel (2012) |
| Berntsen et al. (2010)^[330]^ | Sensewear Pro_2_ Armband | M: 31 ± 9.6 yrs.  F: 39 ± 7.6 yrs. | 20 | M = 14  F = 6 | SPA determined time spent in MVPA – Indirect Calorimetry determined time spent in MVPA.  SPA determined energy expenditure in moderate intensity activity – Indirect Calorimetry determined energy expenditure.  SPA determined energy expenditure in very vigorous intensity activity – Indirect Calorimetry determined energy expenditure.  SPA determined total energy expenditure – Indirect Calorimetry determined total energy expenditure.  Participants wore the devices and the indirect calorimeter while completing activities such as conditioning and strength exercises, ball games, home repair, occupational and home activities (Structured). | Mean_DIFF_=1.1; LoA = 49.9 mins; Overestimated 2.9%;  Agreement: ICC=0.54; 95%CI=0.13-0.79; p<0.01  Overestimated 57%; p<0.05;  Underestimated 92%; p<0.001  Mean_DIFF_=-43.4; LoA = 261.0 kcal; Underestimated 9%;  Agreement: ICC=0.73; 95%CI=0.41-0.87; p<0.001 | Shephard (2012) |
| Berntsen et al. (2010)^[330]^ | ActiGraph 7164 | M: 31 ± 9.6 yrs.  F: 39 ± 7.6 yrs. | 20 | M = 14  F = 6 | AG determined time spent in MVPA – Indirect Calorimetry determined time spent in MVPA.  AG determined energy expenditure in moderate intensity activity (Freedson equation) – Indirect Calorimetry determined energy expenditure.  AG determined energy expenditure in vigorous intensity activity (Freedson equation) – Indirect Calorimetry determined energy expenditure.  AG determined energy expenditure in very vigorous intensity activity (Freedson equation) – Indirect Calorimetry determined energy expenditure.  AG determined energy expenditure in MVPA (Freedson equation) – Indirect Calorimetry determined energy expenditure.  AG determined total energy expenditure – Indirect Calorimetry determined total energy expenditure.  Participants wore the devices and the indirect calorimeter while completing activities such as conditioning and strength exercises, ball games, home repair, occupational and home activities (Structured). | Mean_DIFF_=2.5; LoA = 83.5 mins; Overestimated 2.5%;  Agreement: ICC=0.54; 95%CI=0.15-0.79; p<0.01  Underestimated 67%; p<0.001;  Underestimated 80%; p<0.001  Underestimated 90%; p<0.001  Underestimated 73%; p<0.01  Mean_DIFF_=-50.0; LoA = 396.7 kcal; Underestimated 15%;  Agreement: ICC=0.55; 95%CI=0.16-0.79; p<0.01 | Shephard (2012) |
| Berntsen et al. (2010)^[330]^ | ikcal | M: 31 ± 9.6 yrs.  F: 39 ± 7.6 yrs. | 20 | M = 14  F = 6 | ikcal determined time spent in MVPA – Indirect Calorimetry determined time spent in MVPA.  ikal determined energy expenditure in moderate intensity activity– Indirect Calorimetry determined energy expenditure.  ikal determined energy expenditure in vigorous intensity activity – Indirect Calorimetry determined energy expenditure.  ikal determined energy expenditure in very vigorous intensity activity – Indirect Calorimetry determined energy expenditure.  ilcal determined total energy expenditure – Indirect Calorimetry determined total energy expenditure.  Participants wore the devices and the indirect calorimeter while completing activities such as conditioning and strength exercises, ball games, home repair, occupational and home activities (Structured). | Mean_DIFF_=-4.9; LoA = 44.6 mins; Underestimated 11.6%  Agreement: NS  Overestimated 57%; p<0.05;  Underestimated 56%; p<0.05  Underestimated 87%; p<0.001  Mean_DIFF_=-39.9; LoA = 265.2 kcal; Underestimated 5%  Agreement: ICC=0.71; 95%CI=0.41-0.87; p<0.001 | Shephard (2012) |
| Berntsen et al. (2010)^[330]^ | ActiReg | M: 31 ± 9.6 yrs.  F: 39 ± 7.6 yrs. | 20 | M = 14  F = 6 | ActiReg determined time spent in MVPA – Indirect Calorimetry determined time spent in MVPA.  ActiReg determined energy expenditure in vigorous intensity activity – Indirect Calorimetry determined energy expenditure.  ActiReg determined energy expenditure in very vigorous intensity activity – Indirect Calorimetry determined energy expenditure.  ActiReg determined energy expenditure in MVPA– Indirect Calorimetry determined energy expenditure.  ActiReg determined total energy expenditure – Indirect Calorimetry determined total energy expenditure.  Participants wore the devices and the indirect calorimeter while completing activities such as conditioning and strength exercises, ball games, home repair, occupational and home activities (Structured). | Mean_DIFF_=-34.2; LoA = 52.9 mins; Underestimated 98.7%  Agreement: NS  Underestimated 68%; p<0.001  Underestimated 91%; p<0.001  Underestimated 74%; p<0.01  Mean_DIFF_=-111.1; LoA = 298.2 kcal; Underestimated 21%  Agreement: ICC=0.47; 95%CI=0.02-0.75; p<0.01 | Shephard (2012) |
| Bouten et al. (1994)^[334]^ | Tracmor | 23.5 ± 1.8 yrs. | 11 | M = 11 | Tracmor determined m/s^2^ (Regression analysis) – Indirect Calorimetry determined activity energy expenditure  Sitting, standing and treadmill based activities (structured) | r=0.97; p<0.01  Underestimated 35-140% | Ainslie (2003) |
| Bouten et al. (1997)^[333]^ | Triaxial Accelerometer (Lower Back) | 23-24 yrs. | 2 | M = 2 | Triaxial accelerometer determined integral of the modulus of body acceleration (m.s^-1^) (Linear regression analysis) – Indirect Calorimetry determined energy expenditure (Watts).  Triaxial accelerometer determined integral of the modulus of body acceleration (Simulated based on lower back accelerations) (Linear regression analysis) – Indirect Calorimetry determined energy expenditure (Watts).  Participants walked on a treadmill for 3 minutes at 5 different speeds while measurements were obtained (Structured). | r = 0.92-0.97  r = 0.87 - 0.92 | Mathie (2004) |
| Bouten et al. (1997)^[332]^ | Traixial Accelerometer | 27±4 yrs. | 13 | M = 13 | Triaxial accelerometer determined counts/min (with regression equation to estimate EE_ACT_) – Whole room calorimeter determined watts corrected for body mass (W.kg)  36 hours of monitoring in WRC completing everyday tasks, including household activities, walking etc. (Structured). | r_p_=0.89  Overestimation of 7.5% for estimated EE_ACT_ compared to measured EE_ACT_ | Cheung (2011) |
| Brage et al. (2003)^[336]^ | ActiGraph (CSA) | 22.7-30.0 yrs. | 12 | M = 12 | ActiGraph determined counts/min (regression analysis converting to VO_2_·kg^-1^) – Indirect Calorimetry determined VO_2_·kg^-1^.  3 walking/running trials on treadmill, with a further over ground walking/running trial (structured). | R^2^=0.92;p<0.001  Estimation errors (prediction equations) = 11-48%. | Chen (2005) |
| Brage et al. (2005)^[335]^ | ActiHeart | 26-50 yrs. | 20 | M = 11  F = 9 | ActiHeart determined Physical Activity intensity (Prediction Models) – Indirect Calorimetry  Treadmill protocol involving rest, walking and running ono a treadmill at 6 different speeds (structured). | R^2^>0.84; p<0.001 | DeLany (2012) |
| Brooks et al. (2005)^[337]^ | CSA accelerometer | 35-45 yrs. | 72 | M=36  F=36 | CSA determined counts.mins^-1^ – Douglas Bag determined energy expenditure METs  Participants wore CSA and had Douglas bag measurements while completing 15 minutes of structured walking (Structured). | r=0.41; | Van Remoortel (2012) |
| Brooks et al. (2005)^[337]^ | CSA accelerometer | 35-45 yrs. | 72 | M=36  F=36 | CSA determined energy expenditure METs (Regression1 from counts.mins^-1^) – Douglas Bag determined energy expenditure METs  CSA determined energy expenditure METs (Regression2 from counts.mins^-1^) – Douglas Bag determined energy expenditure METs  CSA determined energy expenditure kcal.min^-1^ (Regression 3 from counts.mins^-1^) – Douglas Bag determined energy expenditure METs  CSA determined energy expenditure kcal.min^-1^ (Regression 4 from counts.mins^-1^) – Douglas Bag determined energy expenditure METs  CSA determined energy expenditure METs (Leenders equation) – Douglas Bag determined energy expenditure METs  CSA determined energy expenditure METs (Hendelman equation) – Douglas Bag determined energy expenditure METs  CSA determined energy expenditure METs (Nichols equation) – Douglas Bag determined energy expenditure METs  CSA determined energy expenditure METs (Freedson equation) – Douglas Bag determined energy expenditure METs  CSA determined energy expenditure METs (Yngve equation) – Douglas Bag determined energy expenditure METs  CSA determined energy expenditure kcal.min^-1^ (Ekelund equation) – Douglas Bag determined energy expenditure METs  CSA determined energy expenditure kcal.min^-1^ (Freedson equation) – Douglas Bag determined energy expenditure METs  Participants wore CSA and had Douglas bag measurements while completing 15 minutes of structured walking (Structured). | r^2^=0.51; SEE=0.44  r^2^=0.60; SEE=0.40  r^2^=0.17; SEE=0.95  r^2^=0.77; SEE=0.50  r^2^=0.51; SEE=0.50; Mean_ERROR_=0.8  r^2^=0.51; SEE=0.52; Mean_ERROR_=0.3  r^2^=0.51; SEE=0.59; Mean_ERROR_=0.8  r^2^=0.51; SEE=0.64; Mean_ERROR_=0.8  r^2^=0.51; SEE=0.66; Mean_ERROR_=0.2  r^2^=0.66; SEE=0.62; Mean_ERROR_=1.2  r^2^=0.76; SEE=1.47; Mean_ERROR_=1.7 | Van Remoortel (2012) |
| Brugniaux et al. (2010)^[338]^ | Polar Activity Watch 200 | 35-55 yrs. | 31 | M=15  F=16 | Activity Watch determined energy expenditure – Indirect Calorimetry determined energy expenditure.  After 30 minutes  After 60 minutes  After 90 minutes  After 120 minutes  Activity monitors worn during 9.7 km hike while also wearing indirect calorimeter (Structured). | r=0.987; p<0.001  Mean_DIFF_=-18.7 kJ; 95%CI = -38.1-0.6  Mean_DIFF_=-94.4 kJ; 95%CI = -130.2 - -58.6  Mean_DIFF_=-64.6 kJ; 95%CI = -200.3 – 128.9  Mean_DIFF_=-300.0 kJ; 95%CI = -351.4 – -248.6 | Van Remoortel (2012) |
| Busse et al. (2009)^[118]^ | Step Activity Monitor | 17-38 yrs. | 20 | NA | SAM determined steps – activPAL determined steps.  Participants wore devices while completing an outdoor track/circuit (Structured). | r=0.96; p<0.001; Underestimation = 0.03 steps.m^-1^. | Shephard (2012) |
| Cain et al. (2013)^[339]^ | ActiGraph GT3X+ | 32.8 ± 11.3 yrs. | 25 | M = 12  F = 13 | ActiGraph GT3X+ (normal filtering) determined steps - ActiGraph 7164 determined steps  ActiGraph GT3X+ (normal filtering) determined Sed mins - ActiGraph 7164 determined Sed mins  ActiGraph GT3X+ (normal filtering) determined LIPA mins - ActiGraph 7164 determined LIPA mins  ActiGraph GT3X+ (normal filtering) determined MPA mins - ActiGraph 7164 determined MPA mins  Two devices worn on same belt for 3 days of free-living activity behaviours (Unstructured). | Mean_DIFF_ = -2041 steps/day; p<0.001.  Mean_DIFF_ = 25.6 mins/day; p<0.001.  Mean_DIFF_ = -31.2 mins/day; p<0.001.  Mean_DIFF_ = -2.9 mins/day; p<0.001. | Gorman (2014) |
| Cain et al. (2013)^[339]^ | ActiGraph GT3X+ | 32.8 ± 11.3 yrs. | 25 | M = 12  F = 13 | ActiGraph GT3X+ (low frequency extension) determined steps - ActiGraph 7164 determined steps  ActiGraph GT3X+ (low frequency extension) determined Sed mins - ActiGraph 7164 determined Sed mins  ActiGraph GT3X+ (low frequency extension) determined LIPA mins - ActiGraph 7164 determined LIPA mins  ActiGraph GT3X+ (low frequency extension) determined MPA mins - ActiGraph 7164 determined MPA mins  Two devices worn on same belt for 3 days of free-living activity behaviours (Unstructured). | Mean_DIFF_ = -3597 steps/day; p<0.001.  Mean_DIFF_ = 7.8 mins/day; NS.  Mean_DIFF_ = -4.3 mins/day; NS.  Mean_DIFF_ = -0.3 mins/day; p<0.001. | Gorman (2014) |
| Campbell et al. (2002)^[340]^ | Tritrac R3D | 20-29 yrs. | 20 | F = 20 | Tritrac determined energy expenditure kcal/min (vector magnitude) – Indirect Calorimetry determined energy expenditure kcal/min.  5 minute bouts of a range of activities wear Cosmed and Tritrac r3d (structured). | Mean _DIFF_=-31%-65%  (Mean diff across range of activities) | Ainslie (2003) |
| Chen et al. (1997)^[342]^ | Tritrac | M 35.7± 10 yrs  F 37.1±9.8 yrs | 125 | M = 53  F = 72 | Tritrac determined activity energy expenditure –Whole room Calorimetry determined energy expenditure (MJ/24 hrs).  24 hour measurement of activity behaviours in whole room calorimeter. Estimated values from Tritrac determined from linear and non-linear models. | r=0.66 – 0.94 | Ainslie (2003) |
| Chen et al. (2003)^[341]^ | Tritrac R3D and Actiwatch | 35.4 ± 9.0 yrs. | 60 | F = 60 | Combined hip worn Tritrac and wrist worn Actiwatch determined AEE (prediction model) – Whole room Calorimetry determined AEE  Hip worn Tritrac determined AEE (prediction model) – Whole room Calorimetry determined AEE  Actiwatch determined AEE (prediction model) – Whole room Calorimetry determined AEE  Range of activity intensities completed within the whole room calorimeter (structured). | Accuracy = 97.7 ±3.2%  Accuracy = 90.0 ±4.6%  Accuracy = 86.0 ±4.7% | Chen (2005) |
| Colbert et al. (2011)^[76]^ | ActiGraph GT1M | 74.7 ± 6.5 yrs. | 56 | M = 12  F = 44 | ActiGraph GT1M determined step count.d^-1^ - NL-2000 determined step count.d^-1^  ActiGraph GT1M determined step count.d^-1^ – SPA determined step count.d^-1^.  Participants wore the devices during the 15 days of DLW measurement and completed the questionnaire on day 8 and day 15 of the study (Unstructured) | r=0.88; Mean_DIFF_ =-1116 ± 179; p<0.001  r=0.89; Mean_DIFF_ =-1104 ± 130; p<0.001 | Plasqui (2013) |
| Colbert et al. (2011)^[76]^ | Sensewear Pro Armband | 74.7 ± 6.5 yrs. | 56 | M = 12  F = 44 | SPA determined step count.d^-1^ – ActiGraph GT1M determined step count.d^-1^  SPA determined step count.d^-1^ – NL-2000 determined step count.d^-1^  Participants wore the devices during the 15 days of DLW measurement and completed the questionnaire on day 8 and day 15 of the study (Unstructured) | r=0.89; Mean_DIFF_ = 1104 ± 130; p<0.001  r=0.87; Mean_DIFF_ = -12 ± 184; NS | Plasqui (2013) |
| Conger et al. (2005)^[281]^ | Fitsense FS1 (Speedometer) | 28.4±5.6 yrs. | 12 | M = 7  F = 5 | Fitsense determined energy expenditure during walking /running – Indirect Calorimetry  Treadmill walking/running at different speeds (structured) | Underestimation with measured walking (p<0.001)  No differences between measured and predicted EE during running (p>0.05) | Chen (2005) |
| Copeland and Esliger (2009)^[343]^ | ActiGraph 7164 | 64-77 yrs. | 38 | M = 18  F = 20 | ActiGraph determined counts/min (regression, but not specified) – Indirect Calorimetry determined energy expenditure (ml·kg^-1^_·min_^-1^)  Participants walked on a treadmill at three walking speeds (Structured) | r=0.60; SEE = 2.48 ml·kg^-1^_·min_^-1^) | Gorman (2014) |
| Copeland and Esliger (2009)^[343]^ | ActiGraph 7164 | 64-77 yrs. | 33 | M = 15  F = 18 | ActiGraph determined MVPA (Copeland) – ActiGraph determined MVPA (Freedson)  7 days free-living activity behaviours (unstructured). | MVPA_Copeland_=68.2 mins/d  MVPA_Freedson_=29.0 mins/d  P<0.001 | Gorman (2014) |
| Crouter et al. (2006)^[347]^ | ActiGraph | 35±11.4 yrs. | 48 | M = 24  F = 24 | Two regression analysis to predict METs using ActiGraph determined counts – Indirect Calorimetry in Cross-Validation group.  Completed 1, 2 or 3 routines of free living activity behaviours (structured). | r=0.96; p<0.001 | Bassett (2008) |
| Crouter et al. (2006)^[346]^ | ActiGraph (7164) | 35 ± 11.4 yrs. | 48 | M = 24  F = 24 | ActiGraph determined count/min (Swart cut point) – Indirect Calorimetry determined Energy Expenditure  ActiGraph determined count/min (Hendelman (lifestyle) cut point) – Indirect Calorimetry determined Energy Expenditure  ActiGraph determined count/min (Leenders cut point) – Indirect Calorimetry determined Energy Expenditure  ActiGraph determined count/min (Nichols cut point) – Indirect Calorimetry determined Energy Expenditure  ActiGraph determined count/min (Freedson (MET) cut point) – Indirect Calorimetry determined Energy Expenditure  ActiGraph determined count/min (Yngve (Treadmill) cut point) – Indirect Calorimetry determined Energy Expenditure  ActiGraph determined count/min (Freedson (kcal) cut point) – Indirect Calorimetry determined Energy Expenditure  ActiGraph determined count/min (Hendelman (walk) cut point) – Indirect Calorimetry determined Energy Expenditure  ActiGraph determined count/min (Yngve (Overground) cut point) – Indirect Calorimetry determined Energy Expenditure  ActiGraph determined count/min (Heil cut point) – Indirect Calorimetry determined Energy Expenditure  ActiGraph determined count/min (Brooks overground kcal cut point) – Indirect Calorimetry determined Energy Expenditure  ActiGraph determined count/min (Brooks overground MET cut point) – Indirect Calorimetry determined Energy Expenditure  ActiGraph determined count/min (Brooks overground kcal cut point) – Indirect Calorimetry determined Energy Expenditure  ActiGraph determined count/min (Brooks overground MET cut point) – Indirect Calorimetry determined Energy Expenditure  3 routines of structured activities (structured). | Kappa = 0.498; p<0.001.  Kappa = 0.447; p<0.001.  Kappa = 0.386; p<0.001.  Kappa = 0.365; p<0.001.  Kappa = 0.362; p<0.001.  Kappa = 0.34; p<0.001.  Kappa = 0.330; p<0.001.  Kappa = 0.328; p<0.001.  Kappa = 0.308; p<0.001.  Kappa = 0.297; p<0.001.  Kappa = 0.271; p<0.001.  Kappa = 0.261; p<0.001.  Kappa = 0.216; p<0.001.  Kappa = 0.194; p<0.001. | Bonomi (2012) |
| Crouter et al. (2006)^[346]^ | Actical | 35 ± 11.4 yrs. | 48 | M = 24  F = 24 | Actical determined count/min (single equation) – Indirect Calorimetry determined Energy Expenditure  Actical determined count/min (2 regression equation) – Indirect Calorimetry determined Energy Expenditure  3 routines of structured activities (structured). | Kappa = 0.413; p<0.001.  Kappa = 0.387; p<0.001. | Bonomi (2012) |
| Crouter et al. (2006)^[346]^ | AMP-331 | 35 ± 11.4 yrs. | 48 | M = 24  F = 24 | AMP-331 determined count/min (Manufacturer equation) – Indirect Calorimetry determined Energy Expenditure  3 routines of structured activities (structured). | Kappa = 0.233; p<0.001. | Bonomi (2012) |
| Crouter et al. (2008)^[344]^ | Actical | 35±11.4 yrs. | 48 | M = 24  F = 24 | Two regression analysis to predict METs using Actical determined counts – Indirect Calorimetry in Cross-Validation group.  Linear regression (Development Group)  Exponential regression (Development Group)  Cubic regression (Development Group)  Completed 1, 2 or 3 routines of free living activity behaviours (structured), and cross validated. | r=0.89; p<0.05  Mean_DIFF_for all activities ≤0.56 METs  r^2^=0.895; SEE=1.051  r^2^=0.912; SEE=0.149  r^2^=0.884; SEE=0.804 | Murphy (2009) |
| Crouter et al. (2008)^[345]^ | ActiHeart | 35±11 yrs. | 48 | M = 24  F = 24 | ActiHeart determined AEE prediction equation using counts – PAEE determined from indirect Calorimetry.  ActiHeart determined PAEE prediction equation using Heart Rate – PAEE determined from indirect Calorimetry.  ActiHeart determined PAEE prediction equation using combined counts and heart rate – PAEE determined from indirect Calorimetry.  Treadmill walking and running (structured) | RMSE = 0.00 – 0.36 Kj/Kg/min  RMSE = 0.03 – 0.18 Kj/Kg/min  RMSE = 0.00 – 0.18 Kj/Kg/min | Corder (2007) |
| Crouter et al. (2011)^[349]^ | Actical | 35 ± 11.4 yrs. | 29 | M=12  F=17 | Actical determined energy expenditure (METs) (2008 2 regression model) during light physical activity – Indirect Calorimeter determined METs during light physical activity.  Actical determined energy expenditure (METs) (2008 2 regression model) during moderate to vigorous physical activity – Indirect Calorimeter determined METs during moderate to vigorous physical activity.  Actical determined energy expenditure (METs) (refined 2008 2 regression model) during light physical activity – Indirect Calorimeter determined METs during light physical activity.  Actical determined energy expenditure (METs) (refined 2008 2 regression model) during moderate to vigorous physical activity – Indirect Calorimeter determined METs during moderate to vigorous physical activity.  Actical determined energy expenditure (METs) (Klippel and Heil regression 1) during light physical activity – Indirect Calorimeter determined METs during light physical activity.  Actical determined energy expenditure (METs) (Klippel and Heil regression 1) during moderate to vigorous physical activity – Indirect Calorimeter determined METs during moderate to vigorous physical activity.  Actical determined energy expenditure (METs) (Klippel and Heil regression 2) during light physical activity – Indirect Calorimeter determined METs during light physical activity.  Actical determined energy expenditure (METs) (Klippel and Heil regression 2) during moderate to vigorous physical activity – Indirect Calorimeter determined METs during moderate to vigorous physical activity.  Participants wore the Actical and the indirect calorimeter from 6 hours during free-living activities (Unstructured). | Mean_DIFF_=34.8: 95% CI = 144.4; R^2^=0.77  Mean_DIFF_=-70.8: 95% CI = 87.9; R^2^=-0.02  Mean_DIFF_=-0.8: 95% CI = 89.1; R^2^=0.25  Mean_DIFF_=8.6: 95% CI = 77.0; R^2^=0.20  Mean_DIFF_=34.1: 95% CI = 97.7; R^2^=0.58  Mean_DIFF_=-29.5: 95% CI = 72.6; R^2^=-0.01  Mean_DIFF_=33.2; 95% CI = 97.5; R^2^=0.57  Mean_DIFF_=-28.6: 95% CI = 72.1; R^2^=-0.01 | Van Remoortel (2012) |
| Crouter et al. (2013)^[348]^ | ActiGraph GT1M | 38 ± 11.7 yrs. | 29 | M = 12  F = 17 | ActiGraph GT1M determined LIPA (mins from Crouter 2006 algorithm) – Indirect Calorimetry LIPA (mins).  ActiGraph GT1M determined MPA (mins from Crouter 2006 algorithm) – Indirect Calorimetry MPA (mins).  ActiGraph GT1M determined MPA (mins from NHANES cut-points) – Indirect Calorimetry MPA (mins).  ActiGraph GT1M determined MPA (mins from Matthews cut-points) – Indirect Calorimetry MPA (mins).  ActiGraph GT1M determined LIPA (mins from Matthews cut-points) – Indirect Calorimetry LIPA (mins).  Both the ActiGraph and indirect calorimeter were worn for 6 hours while completing free-living activities (Unstructured). | Mean_DIFF_= -40.3 mins (34.4%); p<0.05  Mean_DIFF_= 36.1 mins (76.5%); p<0.05  Mean_DIFF_= -23.8 mins (50.4%); p<0.05  Mean_DIFF_= 33.4%; p<0.05  Mean_DIFF_= -30.2 mins (25.7%); p<0.05 | Pedisic (2014) |
| Crouter et al. (2013)^[348]^ | ActiGraph GT1M | 38 ± 11.7 yrs. | 29 | M = 12  F = 17 | ActiGraph GT1M determined energy expenditure (METs from Crouter 2010 algorithm) – Indirect Calorimetry determined energy expenditure (METs).  ActiGraph GT1M determined energy expenditure (METs from Crouter 2006 algorithm) – Indirect Calorimetry determined energy expenditure (METs).  Both the ActiGraph and indirect calorimeter were worn for 6 hours while completing free-living activities (Unstructured). | Mean_DIFF_=0.18; NS  Mean_DIFF_=0.42; p<0.05 | Pedisic (2014) |
| DeVoe et al. (2003)^[350]^ | RT3 research tracker | M: 22.7 ± 2.3 yrs.  F: 24.2 ± 1.5 yrs. | 17 | M=12  F=5 | RT3 determined vector magnitude activity counts – R3D determined vector magnitude activity counts  Participants wore the devices while completing a maximal treadmill test, treadmill walking and outdoor walking (Structured). | r=0.96; p<0.001; Mean_DIFF_= 327 ± 464 VM counts; LoA= -582 – 1236 VM counts | Van Remoortel (2012) |
| DeVoe et al. (2003)^[350]^ | Tritrac R3D | M: 22.7 ± 2.3 yrs.  F: 24.2 ± 1.5 yrs. | 17 | M=12  F=5 | R3D determined vector magnitude activity counts – RT3 determined vector magnitude activity counts  Participants wore the devices while completing a maximal treadmill test, treadmill walking and outdoor walking (Structured). | r=0.96; p<0.001; Mean_DIFF_= -327 ± 464 VM counts; LoA= -582 – 1236 VM counts | Van Remoortel (2012) |
| Donaire-Gonzalez et al. (2012)^[351]^ | Smartphone based CalFit software | 31±8 yrs. | 36 | M =13  F = 23 | Belt worn CalFit determined METs – ActiGraph determined METs (Freedson cut points).  5 days of free-living behaviours (unstructured). | r_s_=0.932  Mean_DIFF_=0.07 METs. | Bort Ruig (2014) |
| Drenowatz et al. (2011)^[352]^ | Sensewear Pro Armband | 24.3 ± 2.8 yrs. | 20 | M = 10  F = 10 | SPA determined METs –Indirect Calorimeter determined METs  Participants wore the SPA while performing 3 treadmill runs (10 minutes) and 1 outdoor run (30 minutes) and wearing the indirect calorimeter (Structured). | r=0.66 ± 0.25; Sig Diff between SPA and IC determined METs; p<0.05 | Van Remoortel (2012) |
| Duncan et al. (2011)^[353]^ | Actical | 39.2 ± 13.5 yrs. | 57 | M = 15  F = 32 | Actical determined activity – Direct Observation determined activity (Laboratory).  Actical determined energy expenditure (kcal) – Indirect Calorimetry determined energy expenditure (kcal) (Laboratory).  Actical determined activity – Direct Observation determined activity (Field).  Actical determined energy expenditure (kcal) – Indirect Calorimetry determined energy expenditure (kcal) (Field).  Participants wore Actical and indirect calorimeter during treadmill walking and running and during field-based activities (Structured). | %_ACCURACY_=60.6 ± 10.5 %; Absolute Accuracy = 60.6 ± 10.5%.  %_ACCURACY_=-44.5 ± 16.1 %; p<0.001  %_ACCURACY_=65.5 ± 9.0 %; Absolute Accuracy = 65.5 ± 9.0%.  %_ACCURACY_=-18.4 ± 6.9%; p<0.001 | Shephard (2012) |
| Duncan et al. (2011)^[353]^ | Multi-sensor board | 39.2 ± 13.5 yrs. | 57 | M = 15  F = 32 | MSB determined activity – Direct Observation determined activity (Laboratory).  MSB determined energy expenditure (kcal) – Indirect Calorimetry determined energy expenditure (kcal) (Laboratory).  MSB determined activity – Direct Observation determined activity (Field).  MSB determined energy expenditure (kcal) – Indirect Calorimetry determined energy expenditure (kcal) (Field).  Participants wore MSB and indirect calorimeter during treadmill walking and running and during field-based activities (Structured). | %_ACCURACY_=92.1 ± 9.7 %; Absolute Accuracy = 89.4 ± 6.6%.  %_ACCURACY_=-10.0 ± 12.4 %;  %_ACCURACY_=76.0 ± 10.1 %; Absolute Accuracy = 76.0 ± 10.1%.  %_ACCURACY_=-12.8 ± 6.5%; p<0.001 | Shephard (2012) |
| Esliger et al. (2011)^[354]^ | GENEActiv | 49.4 ± 6.5 yrs. | 60 | M = 23  F = 37 | GENEA (left wrist) determined sum of the vector magnitude –Indirect Calorimetry determined energy expenditure (METs)  GENEA (right wrist) determined sum of the vector magnitude –Indirect Calorimetry determined energy expenditure (METs)  GENEA (waist) determined sum of the vector magnitude –Indirect Calorimetry determined energy expenditure (METs)  GENEA (waist) determined sum of the vector magnitude – Actigraph determined counts.m^-1^  GENEA (waist) determined sum of the vector magnitude – RT3 determined sum of the vector magnitude  Participants wore devices while completing 10-12 activities in a laboratory setting (Structured). | r=0.86; Accuracy = 0.93  r=0.83; Accuracy = 0.90  r=0.87; Accuracy = 0.95  r=0.86  r=0.88 | Plassqui (2013) |
| Esliger et al. (2011)^[354]^ | ActiGraph GT1M | 49.4 ± 6.5 yrs. | 60 | M = 23  F = 37 | Actigraph determined counts.m^-1^ –Indirect Calorimetry determined energy expenditure (METs)  Participants wore devices while completing 10-12 activities in a laboratory setting (Structured). | r=0.86; Accuracy = 0.94 | Plassqui (2013) |
| Esliger et al. (2011)^[354]^ | RT3 accelerometer | 49.4 ± 6.5 yrs. | 60 | M = 23  F = 37 | RT3 determined sum of the vector magnitude –Indirect Calorimetry determined energy expenditure (METs)  Participants wore devices while completing 10-12 activities in a laboratory setting (Structured). | r=0.88; Accuracy = 0.95 | Plassqui (2013) |
| Fehling et al. (1999)^[355]^ | Caltrac | 70.6±3.7 yrs. | 86 | M = 44  F = 42 | Caltrac determined kcal/min – Indirect Calorimetry determined kcal/min.  Caltrac determined kcal/min – Indirect Calorimetry determined kcal/min (During Stepping Activities  3 submaximal progressive levels of treadmill walking and running (5 minutes in duration) (Structured). | %_DIFF_=10-52%  %_DIFF_=-19 - -28% | Chen (2005) |
| Fehling et al. (1999)^[355]^ | Tritrac | 70.6±3.7 yrs. | 86 | M = 44  F = 42 | Tritrac determined kcal/min – Indirect Calorimetry determined kcal/min.  Tritrac determined kcal/min – Indirect Calorimetry determined kcal/min (During Stepping Activities  3 submaximal progressive levels of treadmill walking and running (5 minutes in duration) (Structured). | %_DIFF_=-37 - -12%  %_DIFF_=-58 - -60% | Chen (2005) |
| Freedson et al (1998)^[356]^ | ActiGraph (CSA) | 24.8 ± 4.2 yrs. | 50 | M = 25  F = 25 | ActiGraph determined counts (regression) – Indirect Calorimetry  Treadmill-based structured activities | r=0.88 | Reilly (2008)  Bassett (2000) |
| Fruin et al. (2004)^[357]^ | Sensewear Pro Armband | 19-22 yrs. | 13 | M = 13 | SWA determined PAEE (kcal/min) – Indirect Calorimetry determined PAEE (kcal/min)  Structured rest, cycle ergometer and treadmill walking/running. | r_REST_=0.73; p<0.01  r_ERGOMETER_= 0.03-0.12  r_Treadmill_= 0.47-0.69  Mean_error_= -22-38% | Corder (2008) |
| Fudge et al. (2007)^[358]^ | ActiGraph (CSA) | 19-31 yrs. | 16 | M = 16 | ActiGraph (MTI) determined counts/min – Indirect Calorimeter determined (mL.Kg.min).  Incremental walking and running (3-22 kmph) on a treadmill (Trained Athletes) (structured) | r_Walk_=0.70; p<0.001; R^2^=0.48; SEE=3.86 | Corder (2007) |
| Fudge et al. (2007)^[358]^ | ActiGraph GT1M | 19-31 yrs. | 16 | M = 16 | ActiGraph GT1M determined counts/min – Indirect Calorimeter determined (mL.Kg.min).  Incremental walking and running (3-22 kmph) on a treadmill (Trained Athletes) (structured) | r_Walk_ =0.91; p<0.001; R^2^=0.81; SEE=2.17 | Corder (2007) |
| Fudge et al. (2007)^[358]^ | ActiHeart | 19-31 yrs. | 16 | M = 16 | ActiHeart determined counts/min – Indirect Calorimeter determined (mL.Kg.min).  Incremental walking and running (3-22 kmph) on a treadmill (Trained Athletes) (structured) | r_Walk_ =0.90; p<0.001; R^2^=0.81; SEE=2.17 | Corder (2007) |
| Fudge et al. (2007)^[358]^ | BioTel 3dNX | 19-31 yrs. | 16 | M = 16 | 3dNX determined counts/min – Indirect Calorimeter determined (mL.Kg.min).  Incremental walking and running (3-22 kmph) on a treadmill (Trained Athletes) (structured) | r_Walk_ =0.91; p<0.001; R^2^=0.76; SEE=4.88  r_Run_=0.87; p<0.001 | Corder (2007) |
| Fuller et al. (2008)^[79]^ | Caltrac | 20-66 yrs. | 60 | M = 30  F = 30 | Caltrac determined energy expenditure (MJ.d^-1^) – Heart rate monitor determined energy expenditure (MJ.d^-1^) (from calibrated FLEX HR)  Caltrac determined energy expenditure (MJ.d^-1^) – PAD determined energy expenditure (MJ.d^-1^)  Caltrac determined energy expenditure (MJ.d^-1^) – 7DPAR determined energy expenditure (MJ.d^-1^)  15 days of DLW measurement, HRM, Physical Activity Diary, Caltrac device and 2 versions of the 7DPARQ (Day 8 and day 15 (Unstructured). | r=0.68  r=0.48  r=0.53 | Plasqui (2013) |
| Fuller et al. (2008)^[79]^ | Caltrac | 20-66 yrs. | 60 | M = 30  F = 30 | Caltrac determined energy expenditure (PAL) – Heart rate monitor determined energy expenditure (PAL) (from calibrated FLEX HR)  Caltrac determined energy expenditure (PAL) – PAD determined energy expenditure (PAL)  Caltrac determined energy expenditure (PAL) – 7DPAR determined energy expenditure (PAL)  15 days of DLW measurement, HRM, Physical Activity Diary, Caltrac device and 2 versions of the 7DPARQ (Day 8 and day 15 (Unstructured). | r=0.45  r=0.23  r=0.20 | Plasqui (2013) |
| Haymes and Barnes (1993)^[362]^ | Caltrac | NA | 20 | M = 10  F = 10 | Caltrac estimated energy expenditure walking– Indirect Calorimetry  Caltrac estimated energy expenditure running– Indirect Calorimetry  Treadmill walking and running at a range of speeds (structured) | r=0.91  r=0.71 | Bassett et al. (2000) |
| Harrington et al. (2011)^[361]^ | activPAL | 15-25 yrs. | 62 | F = 62 | activPAL determined MET estimates – Indirect Calorimetry determined MET estimates.  activPAL determined steps – Indirect Calorimetry determined MET estimates.  activPAL determined counts.15 seconds^-1^ – Indirect Calorimetry determined MET estimates.  activPAL determined steps – Direct Observation  activPAL, indirect Calorimetry and direct observation of treadmill walking at 5 different speeds (Structured). | Mean_DIFF_=-0.4 – 2.0 METs; p<0.001; ICC=0.57;  r=0.59; p<0.001  r=0.76; p<0.001.  95%LoA = -3.3 – 3.9 steps; Mean bias = 0.3 steps. | Lowe (2014) |
| Hageman et al. (2004)^[359]^ | Caltrac | 52 ± 7.7 yrs. | 43 | M=28  F=15 | Caltrac determined energy expenditure (kcals) – Indirect Calorimetry determined energy expenditure (kcals).  Participants had indirect Calorimetry and activity monitors measurement obtained during the 1 mile walking test (Structured). | r_p_=0.67; p<0.001  Mean_DIFF_=-31.9 ± 19.9 kcals; 95%CI=-37.8-26.0 | Van Remoortel (2012) |
| Hageman et al. (2004)^[359]^ | Biotrainer | 52 ± 7.7 yrs. | 43 | M=28  F=15 | Biotrainer determined energy expenditure (kcals) – Indirect Calorimetry determined energy expenditure (kcals).  Participants had indirect Calorimetry and activity monitors measurement obtained during the 1 mile walking test (Structured). | r_p_=0.43; p<0.01  Mean_DIFF_=-35.2 ± 26.1 kcals; 95%CI=-43.0-27.4 | Van Remoortel (2012) |
| Ham et al. (2007)^[360]^ | ActiGraph 7164 | 22-56 yrs. | 12 | M = 7  F = 5 | ActiGraph determined MVPA bouts > 10 minutes (Freedson Equation) – MVPA bouts > 10 minutes using 45%HRR  7 consecutive days of free-living activity behaviours (unstructured) | Accuracy = 21.7% | Corder (2007) |
| Ham et al. (2007)^[360]^ | ActiGraph 7164 | 22-56 yrs. | 12 | M = 7  F = 5 | ActiGraph determined MVPA bouts > 10 minutes (Hendelman Equation) – MVPA bouts > 10 minutes using 45%HRR  7 consecutive days of free-living activity behaviours (unstructured) | Accuracy = 12.0% | Corder (2007) |
| Ham et al. (2007)^[360]^ | ActiGraph 7164 | 22-56 yrs. | 12 | M = 7  F = 5 | ActiGraph determined MVPA bouts > 10 minutes (Swartz Equation) – MVPA bouts > 10 minutes using 45%HRR  7 consecutive days of free-living activity behaviours (unstructured) | Accuracy = 5.3% | Corder (2007) |
| Hart et al. (2011)^[191]^ | ActiGraph GT1M | 18-60 yrs. | 32 | M = 16  F = 16 | ActiGraph determined minutes spent stepping – activPAL determined minutes spent stepping  Single day of physical activity monitoring (unstructured) | Mean_DIFF_= 93.2 mins; p<0.001  %Agr =54.0% | Gorman (2014) |
| Haymes and Barnes (1993)^[362]^ | Caltrac | NA | 20 | M = 10  F = 10 | Caltrac determined energy expenditure during walking (kcal.min) – Indirect Calorimetry determined energy expenditure (kcal.min)  Caltrac determined energy expenditure during running (kcal.min) – Indirect Calorimetry determined energy expenditure (kcal.min)  Treadmill walking and running at a range of speeds (structured) | r=0.87; p<0.05  r=0.29; NS | Bassett et al. (2000) |
| Hendelman et al. (2000)^[364]^ | Tritrac | 30-50 yrs. | 25 | M = 10  F = 15 | Tritrac determined counts – ActiGraph determined counts  Over ground self-paced walking (5minutes in duration), played 2 holes of golf and performed household tasks (structured). | r_walk_=0.87  r_all_=0.93 | Westerterp (2009) |
| Hendelman et al. (2000)^[364]^ | ActiGraph (CSA) | 30-50 yrs. | 25 | M = 10  F = 15 | ActiGraph determined counts (regression analysis) – Indirect Calorimetry  Over ground self-paced walking (5minutes in duration), played 2 holes of golf and performed household tasks (structured). | r_walk_=0.77; R^2^=0.589, SEE=0.868  r_all_=0.59 R^2^=0.352, SEE=0.957 | Westerterp (2009) |
| Hendelman et al. (2000)^[364]^ | Tritrac | 30-50 yrs. | 25 | M = 10  F = 15 | Tritrac determined counts (regression analysis) – Indirect Calorimetry  Over ground self-paced walking (5minutes in duration), played 2 holes of golf and performed household tasks (structured). | r_walk_=0.89; R^2^=0.784, SEE=0.624  r_all_=0.62; R^2^=0.386, SEE=0.931 | Westerterp (2009) |
| Herman Hansen et al. (2013)^[365]^ | ActiGraph GT1M | 28.2 ± 3.3 yrs. | 20 | NA | ActiGraph GT1M determined activity counts.min^-1^ (Level walking) – Indirect Calorimetry determined energy expenditure (VO_2_ ml.kg^-1^.min^-1^).  ActiGraph GT1M determined activity counts.min^-1^ (Graded Walking at 5%) – Indirect Calorimetry determined energy expenditure (VO_2_ ml.kg^-1^.min^-1^).  ActiGraph GT1M determined activity counts.min^-1^ (Graded Walking at 8%) – Indirect Calorimetry determined energy expenditure (VO_2_ ml.kg^-1^.min^-1^).  ActiGraph GT1M determined activity counts.min^-1^ (Cycling) – Indirect Calorimetry determined energy expenditure (VO_2_ ml.kg^-1^.min^-1^).  Participants wore the ActiGraph GT1M and had indirect Calorimetry measured while completing treadmill walking and cycling (Structured). | R^2^=0.82  R^2^=0.82(EE underestimated with increased gradient)  R^2^=0.67; p<0.001 (EE underestimated with increased gradient)  R^2^=0.000-0.002; | Pedisic (2014) |
| Howe et al. (2009)^[366]^ | RT3 accelerometer | 20-60 yrs. | 212 | M=91  F=121 | RT3 determined activity energy expenditure – Indirect Calorimetry determined activity energy expenditure  Participants wore an indirect calorimeter and RT3 accelerometer while completing treadmill activities and activities of daily living (Structured). | Mean_DIFF_=-0.47 ± 0.06 kcal.min^-1^; p<0.0001; %_DIFF_=8.4% | Van Remoortel (2012) |
| Hustvedt et al. (2004)^[367]^ | ActiReg | 23.3 ± 2.1 yrs. | 10 | M = 6  F = 4 | ActiReg determined energy expenditure (kJ/h) – Indirect Calorimetry determined energy expenditure (kJ/h)  Participants completed a range of activities while wearing the ActiReg (with HRM) in a whole room calorimeter (Structured). | Mean_DIFF_=-8 kJ/h; LoA = -168 – 152 kJ/h | Plasqui (2007) |
| Hustvedt et al. (2004)^[367]^ | ActiReg | 23.7 ± 2.5 yrs. | 18 | F = 18 | ActiReg (with Heart Rate from calibrated Flux HR point) determined energy expenditure (kJ/h) – Indirect Calorimetry determined energy expenditure (kJ/h)  ActiReg (Only) determined energy expenditure (kJ/h) – Indirect Calorimetry determined energy expenditure (kJ/h)  Participants completed a range of activities while wearing the ActiReg (with HRM) during 10 days of DLW measurement (Unstructured). | Mean_DIFF_=0.41 kJ/h; LoA = -2.3 – 3.1 kJ/h  Mean_DIFF_=0.78 kJ/h; LoA = -1.66 – 3.2 kJ/h | Plasqui (2007) |
| Jakicic et al. (1999)^[369]^ | Tritrac R3D | 21.5±3.4 yrs. | 20 | M = 6  F = 14 | Tritrac determined kcal/min – indirect Calorimetry determined kcal/min.  5 different exercises for 10 minutes in duration (structured) | r=0.17-86 (Across a wide range of activities) | Ainslie (2003) |
| Jakicic et al. (2004)^[368]^ | SenseWear Pro Armband | 23.2 ± 3.8 yrs. | 40 | M =  F = | Sensewear determined energy expenditure (kcal.min^-1^ (General Equations)) – Indirect Calorimetry determined energy expenditure (kcal.min^-1^).   - Walking - Cycle Ergometry - Step - Arm Ergometry   Sensewear determined energy expenditure (kcal.min^-1^ (Exercise Specific Equations)) – Indirect Calorimetry determined energy expenditure (kcal.min^-1^).   - Walking - Cycle Ergometry - Step - Arm Ergometry   4 exercise protocols while wearing the equipment (Structured). | ICC=0.77; 95%CI = 0.57 – 0.88  ICC=0.28; 95%CI = -0.05 – 0.56  ICC=0.63; 95%CI = 0.39 – 0.79  ICC=0.74; 95%CI = 0.55 – 0.86  ICC=0.87; 95%CI = 0.75 – 0.93  ICC=0.89; 95%CI = 0.74 – 0.95  ICC=0.82; 95%CI = 0.58 – 0.92  ICC=0.66; 95%CI = 0.28 – 0.86 | Matthews (2005) |
| John et al. (2010)^[371]^ | ActiGraph 7164 | 23.6 ± 2.7 yrs. | 10 | M = 10 | ActiGraph 7164 determined activity counts.min^-1^ – ActiGraph GT1M Version 1 determined activity counts.min^-1^.  ActiGraph 7164 determined activity counts.min^-1^ – ActiGraph GT1M Version 2 determined activity counts.min^-1^.  ActiGraph 7164 determined activity counts.min^-1^ – ActiGraph GT1M Version 3 determined activity counts.min^-1^.  Participants wore both devices while walking and running on a treadmill at 10 different speeds for 3 minutes each (Structured). | No significant differences observed.  No significant differences observed.  No significant differences observed. | Loprinzi (2012) |
| Kane et al. (2010)^[372]^ | Nike+ Device | 24.1 ± 4.0 yrs. | 20 | M=11  F=9 | Nike+ determined energy expenditure (kcal.min) – Indirect Calorimetry determined energy expenditure (kcal.min) (during walking activities only)  Nike+ determined energy expenditure (kcal.min) – Indirect Calorimetry determined energy expenditure (kcal.min) (during jogging activities only)  Participants wore the Nike+ device and had indirect Calorimetry measurement completed during 8 six minute stages on the treadmill (Structured). | %_DIFF_=18-37%  No sig differences, | Van Remoortel (2012) |
| King et al. (2004)^[373]^ | Sensewear Pro Armband | M=25.2 ± 4.5 yrs.  F=24.7 ± 5.4 yrs. | 21 | M = 10  F = 11 | SWA determined TEE –Indirect Calorimetry determined TEE  Treadmill walking and running at 7 different speeds (structured) | r=0.65-0.85 | Corder (2008) |
| King et al. (2004)^[373]^ | ActiGraph (CSA) | M=25.2 ± 4.5 yrs.  F=24.7 ± 5.4 yrs. | 21 | M = 10  F = 11 | ActiGraph determined TEE (Freedson Equations) –Indirect Calorimetry determined TEE  ActiGraph determined TEE (Manufacturer Equations) –Indirect Calorimetry determined TEE  Treadmill walking and running at 7 different speeds (structured) | r=0.49-0.73  r=0.14-0.56 | Corder (2008) |
| King et al. (2004)^[373]^ | Tritrac R3D | M=25.2 ± 4.5 yrs.  F=24.7 ± 5.4 yrs. | 21 | M = 10  F = 11 | Tritrac R3D determined TEE –Indirect Calorimetry determined TEE  Treadmill walking and running at 7 different speeds (structured) | r=0.48-0.84 | Corder (2008) |
| King et al. (2004)^[373]^ | RT3 | M=25.2 ± 4.5 yrs.  F=24.7 ± 5.4 yrs. | 21 | M = 10  F = 11 | RT3 determined TEE –Indirect Calorimetry determined TEE  Treadmill walking and running at 7 different speeds (structured) | r=0.39-0.76 | Corder (2008) |
| King et al. (2004)^[373]^ | Biotrainer Pro | M=25.2 ± 4.5 yrs.  F=24.7 ± 5.4 yrs. | 21 | M = 10  F = 11 | Biotrainer determined TEE –Indirect Calorimetry determined TEE  Treadmill walking and running at 7 different speeds (structured) | r=0.34-0.64 | Corder (2008) |
| Koehler et al. (2011)^[294]^ | Sensewear Pro Armband | 30.4 ± 6.2 yrs. | 14 | M = 14 | SPA determined total energy expenditure – Indirect Calorimetry determined total energy expenditure.  SPA determined total energy expenditure – Indirect Calorimetry determined total energy expenditure.  Participants wore the SPA during a range of activities while also wearing and indirect calorimeter device (Structured). | Significant underestimation at all activities (excl. 2.4 m.s^-1^; p<0.01; Mean_DIFF_=4.5 kcal.min^-1^; 95% LoA=-11.4 – 2.4 kcal.min^-1^  Mean_DIFF_=-6.6 kcal.min^-1^; 95% LoA=-14.8 – 1.6 kcal.min^-1^; p<0.001 | Shephard (2012) |
| Kuffel et al. (2011)^[374]^ | ActiGraph GT1M | 28 ± 7.7 yrs. | 30 | M = 14  F = 16 | ActiGraph determined energy expenditure kcal.kg.hr^-1^ (Crouter 2regression method (2006)) – Indirect Calorimetry determined energy expenditure kcal.kg.hr^-1^.  ActiGraph determined energy expenditure kcal.kg.hr^-1^ (Crouter 2regression method (2011)) – Indirect Calorimetry determined energy expenditure kcal.kg.hr^-1^.  Participants wore the activity monitor and the Cosmed for 15 minutes of seated rest, 8 minutes of walking and another 8 minutes of seated rest (Structured). | Mean_BIAS_=-0.14; 95% PI: -2.4 – 2.2 kcal.kg.hr^-1^.  Mean_BIAS_=0.16; 95% PI: -1.5 – 1.8 kcal.kg.hr^-1^. | Van Remoortel (2012) |
| Leenders et al. (2003)^[375]^ | ActiGraph CSA | 23.7 ± 3.9 yrs. | 28 | M = 11  F = 17 | CSA determined energy expenditure (from regression using counts.min^-1^) – Indirect Calorimeter determined energy expenditure.  CSA (ankle) determined energy expenditure (from regression using counts.min^-1^) – Indirect Calorimeter determined energy expenditure.  CSA (wrist) determined energy expenditure (from regression using counts.min^-1^) – Indirect Calorimeter determined energy expenditure.  Participants completed 5 different locomotor speeds on a treadmill while wearing 4 devices and an indirect calorimeter (Structured) | R^2^=0.74; RMSE = 0.009  R^2^=0.27; RMSE = 0.015  R^2^=0.28; RMSE = 0.015 | Loprinzi (2012) |
| Leenders et al. (2003)^[375]^ | Tritrac R3D | 23.7 ± 3.9 yrs. | 28 | M = 11  F = 17 | Tritrac determined energy expenditure (from regression using counts.min^-1^) – Indirect Calorimeter determined energy expenditure.  Tritrac determined energy expenditure (from regression using Vector Magnitude) – Indirect Calorimeter determined energy expenditure.  Participants completed 5 different locomotor speeds on a treadmill while wearing 4 devices and an indirect calorimeter (Structured) | R^2^=0.81; RMSE = 0.008  R^2^=0.85; SEE = 0.50 kcal.min^-1^; RMSE = 0.007 | Loprinzi (2012) |
| Leenders et al. (2003)^[375]^ | Mini-Logger | 23.7 ± 3.9 yrs. | 28 | M = 11  F = 17 | Mini-Logger determined energy expenditure (from regression using counts.min^-1^) – Indirect Calorimeter determined energy expenditure.  Participants completed 5 different locomotor speeds on a treadmill while wearing 4 devices and an indirect calorimeter (Structured) | R^2^=0.69; RMSE = 0.010 | Loprinzi (2012) |
| Levine et al. (2001)^[376]^ | Tracmor | 34 ± 5 yrs. | 11 | M = 5  F = 6 | Combined Inclinometer (worn on the back) and Tracmor determined NEAT (kcal) – Whole room calorimeter determined NEAT (kcal).  250 minutes in WRC wearing devices completing various spontaneous activities (structured). | ICC = 86%; p<0.05  (14% between method disagreements). | Chen (2005) |
| Levine et al. (2009)^[377]^ | Physical activity sensing earpiece | 27 ± 9 yrs. | 18 | M=9  F=9 | PASE determined energy expenditure (kcal.kg.hr^-1^) – Indirect Calorimetry determined energy expenditure (kcal.kg.hr^-1^).  Participants wore PASE and indirect calorimeter while completing sitting, standing and locomotor activities (Structured). | %_ERROR_=0.22 ± 4.8% | Van Remoortel (2012) |
| Lyden et al. (2011)^[378]^ | ActiGraph | 38.3 ± 12.4 yrs. | 274 | M= 135  F = 139 | ActiGraph determined energy expenditure (METs Freedson equation) – Indirect Calorimetry determined energy expenditure (METs).  ActiGraph determined energy expenditure (METs Swartz equation) – Indirect Calorimetry determined energy expenditure (METs).  ActiGraph determined energy expenditure (METs Crouter equation) – Indirect Calorimetry determined energy expenditure (METs).  ActiGraph determined energy expenditure (kcal Freedson equation) – Indirect Calorimetry determined energy expenditure (kcal).  Participants completed a range of treadmill based activities and self-paced activities of daily living while wearing devices and an indirect calorimeter (Structured). | Bias = -1.4; 95%CI = -1.4 - -1.3; RMSE = 2.3; p<0.05  Bias = -0.6; 95%CI = -0.6 - -0.5; RMSE = 2.0; p<0.05  Bias = -0.6; 95%CI = -0.7 - -0.6; RMSE = 2.0; p<0.05  Bias = -1.1; 95%CI = -1.2 - -1.0; RMSE = 2.9; p<0.05 | Pedisic (2014) |
| Lyden et al. (2011)^[378]^ | Actical | 38.3 ± 12.4 yrs. | 274 | M= 135  F = 139 | Actical determined energy expenditure (METs Klippel and Heil 1R equation) – Indirect Calorimetry determined energy expenditure (METs).  Actical determined energy expenditure (METs Klippel and Heil 1R equation) – Indirect Calorimetry determined energy expenditure (METs).  Actical determined energy expenditure (METs Crouter AC equation) – Indirect Calorimetry determined energy expenditure (METs).  Actical determined energy expenditure (kcal Heil 1R equation) – Indirect Calorimetry determined energy expenditure (kcal).  Actical determined energy expenditure (kcal Heil 2R equation) – Indirect Calorimetry determined energy expenditure (kcal).  Participants completed a range of treadmill based activities and self-paced activities of daily living while wearing devices and an indirect calorimeter (Structured). | Bias = -0.8; 95%CI = -0.9 - -0.7; RMSE = 2.2; p<0.05  Bias = -0.8; 95%CI = -0.8 - -0.7; RMSE = 2.1; p<0.05  Bias = -0.1; 95%CI = -0.2 - -0.0; RMSE = 2.3  Bias = -1.3; 95%CI = -1.4 - -1.2; RMSE = 2.9; p<0.05  Bias = -1.3; 95%CI = -1.4 - -1.2; RMSE = 2.8; p<0.05 | Pedisic (2014) |
| Lyden et al. (2011)^[378]^ | RT3 accelerometer | 38.3 ± 12.4 yrs. | 274 | M= 135  F = 139 | RT3 determined Gross energy expenditure (kcals) – Indirect Calorimetry determined energy expenditure (kcals).  RT3 determined activity energy expenditure (kcals) – Indirect Calorimetry determined energy expenditure (kcals).  Participants completed a range of treadmill based activities and self-paced activities of daily living while wearing devices and an indirect calorimeter (Structured). | Bias = -0.5; 95%CI = -0.6 - -0.3; RMSE = 2.9; p<0.05  Bias = -0.5; 95%CI = -0.6 - -0.4; RMSE = 2.9; p<0.05 | Pedisic (2014) |
| Maddison et al. (2009)^[304]^ | RT3 accelerometer | 39 ± 10 yrs. | 36 | M = 16  F = 20 | RT3 determined total activity counts – Doubly labelled water determined total energy expenditure.  14 days of RT3 data compared with 15 days of DLW measurement (Unstructured). | r=0.32; p<0.05 | Plasqui (2013) |
| Maliszewski et al. (1991)^[379]^ | Caltrac | 20±1.99 yrs. | 10 | M = 10 | Caltrac determined kcal –Indirect Calorimetry kcal/min  Treadmill walking at 3 different speeds (structured) | r=0.90; p<0.05 | Ainslie (2003) |
| Matthews et al (2000)^[380]^ | Actillume Monitor | 25-59 yrs. | 19 | M = 7  F = 12 | Actillume determined METs (based on regression analysis using counts/min) – Indirect Calorimeter determined energy expenditure (METs)  4 non walking and 3 treadmill walking trials (Structured) | R^2^=0.79; RMSE = 0.58 | Freedson (2000) |
| Matthews et al. (2013)^[150]^ | activPAL accelerometer | 12-17 yrs. | 91 | M = 43  F = 48 | activPAL determined active time (hrsd^-1^) - ActiGraph determined active time (hrsd^-1^).  Participants completed 3 PARQ over a 7 day period compared to7 days of ActiGraph measured free-living physical activity (Unstructured). | Mean_DIFF_= 0.04 - 1.93; CV_DIFF_% = 29%-32%; r_s_=0.08 - -0.47^*^. (*=p<0.05). | Pedisic (2014) |
| MacFarlane et al (2006)^[145]^ | Tritrac | 15-55 yrs. | 49 | M = 30  F = 19 | Tritrac determined activity intensity (Nichols cut points) -  HR determined activity intensity (Howley cut points)  Free-living physical activity behaviours over 7 days using each measure | r_s Light_=0.10  r_s Moderate_=0.14  r_s Vigorous_=0.36; p<0.05 | Andrew (2010) |
| MacFarlane et al (2006)^[145]^ | Tritrac | 15-55 yrs. | 49 | M = 30  F = 19 | Tritrac determined activity intensity (Nichols cut points) – MTI determined activity intensity (Freedson cut points)  Free-living physical activity behaviours over 7 days using each measure | r_s Light_=0.59; p<0.001  r_s Moderate_= 0.77 p<0.001  r_s Vigorous_=0.53; p<0.001 | Andrew (2010) |
| MacFarlane et al (2006)^[145]^ | ActiGraph (MTI) | 15-55 yrs. | 49 | M = 30  F = 19 | MTI determined activity intensity (Freedson cut points)-  HR determined activity intensity (Howley cut points)  Free-living physical activity behaviours over 7 days using each measure | r_s Light_=0.14  r_s Moderate_= 0.24; p<0.05  r_s Vigorous_=0.63; p<0.01 | Andrew (2010) |
| MacFarlane et al (2006)^[145]^ | ActiGraph (MTI) | 15-55 yrs. | 49 | M = 30  F = 19 | MTI determined activity intensity (Freedson cut points) - Tritrac determined activity intensity (Nichols cut points)  Free-living physical activity behaviours over 7 days using each measure | r_s Light_=0.59; p<0.001  r_s Moderate_= 0.77 p<0.001  r_s Vigorous_=0.53; p<0.001 | Andrew (2010) |
| Melanson et al. (1995)^[381]^ | Caltrac | 21.0±1.1 yrs | 28 | M = 15  F = 13 | Caltrac activity counts (stepwise multiple regression) – Indirect Calorimetry (kcal.min^-1^)  Slow walking, brisk walking and jogging on a treadmill (structured). | r=0.82; p<0.01 | Bassett (2000)  Berlin (2006) |
| Melanson et al. (1995)^[381]^ | Caltrac | 21.0±1.1 yrs | 28 | M = 15  F = 13 | Caltrac determined energy expenditure – Indirect Calorimetry (kcal.min^-1^)  Slow walking, brisk walking and jogging on a treadmill (structured). | r=0.86; p<0.01  Mean_DIFF_=0.21 kcal.min^-1^ | Bassett (2000)  Berlin (2006) |
| Melanson et al. (1995)^[381]^ | ActiGraph (CSA) | 21.0±1.1 yrs | 28 | M = 15  F = 13 | CSA activity counts (ankle) (stepwise multiple regression) – Indirect Calorimetry (kcal.min^-1^)  Slow walking, brisk walking and jogging on a treadmill (structured). | r=0.66; p<0.01 | Bassett (2000)  Berlin (2006) |
| Melanson et al. (1995)^[381]^ | ActiGraph (CSA) | 21.0±1.1 yrs | 28 | M = 15  F = 13 | CSA activity counts (hip) (stepwise multiple regression) – Indirect Calorimetry (kcal.min^-1^)  Slow walking, brisk walking and jogging on a treadmill (structured). | r=0.80; p<0.01 | Bassett (2000)  Berlin (2006) |
| Melanson et al. (1995)^[381]^ | ActiGraph (CSA) | 21.0±1.1 yrs | 28 | M = 15  F = 13 | CSA activity counts (wrist) (stepwise multiple regression) – Indirect Calorimetry (kcal.min^-1^)  Slow walking, brisk walking and jogging on a treadmill (structured). | r=0.81; p<0.01 | Bassett (2000)  Berlin (2006) |
| Midorikawa et al. (2007)^[382]^ | AC-301 Triaxial accelerometer | 33±15 yrs. | 21 | M = 8  F =13 | AC-301 determined energy expenditure (predicted from accelerometer count regression equations) – Indirect Calorimetry determined physical activity Ratio  Structured activities including walking on treadmill, sitting, standing and some activities to replicate household tasks | R^2^=0.45-0.72 (across the range of activities). | Westerterp (2009) |
| Miller et al. (2010)^[383]^ | ActiGraph 7164 | 20-69 yrs. | 90 | M = 34  F = 56 | ActiGraph determined energy expenditure (Miller Prediction Equations)– Indirect Calorimetry determined METs  ActiGraph determined energy expenditure (Brage Prediction Equations) – Indirect Calorimetry determined METs  ActiGraph determined energy expenditure (Freedson Prediction Equations) – Indirect Calorimetry determined METs  ActiGraph determined energy expenditure (Yngve Prediction Equations) – Indirect Calorimetry determined METs  Treadmill walking at 2 speeds and running (structured). | R^2^=0.62-0.89 (SEE=0.92-1.05)  R^2^=0.89 (SEE = 0.91)  R^2^=0.82 (SEE = 1.12)  R^2^=0.85; (SEE = 1.14) | Gorman (2014) |
| Nichols et al. (1999)^[384]^ | Tritrac | 23.4 ± 2.9 yrs. | 60 | M = 30  F = 30 | Tritrac determined vector magnitude and kcal/kg/min – Indirect Calorimeter determined energy expenditure (kcal/kg/min).  Walking and jogging at 3 speeds for 5 minutes on a treadmill (structured). | Mean _DIFF_=  -0.01-0.03 kcal/kg/min. | Ainslie (2003) |
| Ohkawara et al. (2011)^[385]^ | LIS3LV02DQ Triaxial accelerometer | 42.4 ± 13.5 yrs. | 66 | M=41  F=25 | LIS determined locomotor activity (regression equation converting counts to MET values) – Indirect Calorimeter determined filtered acceleration  LIS determined household activity (regression equation converting counts to MET values) – Indirect Calorimeter determined filtered acceleration  LIS determined household and locomotor activity (regression equation converting counts to MET values) – Indirect Calorimeter determined filtered acceleration  Absolute and Percentage difference available for 5 models compared to indirect Calorimetry across 11 activities. All activities conducted in structured laboratory setting (Structured). | r=0.961; p<0.001  r=0.907; p<0.001  r=0.930; p<0.001  Bland-Altman | Van Remoortel (2012) |
| Pambianco et al. (1990)^[386]^ | Caltrac | 20-35 yrs. (overweight) | 20 | M = 10  F = 10 | Caltrac determined kcal – Indirect Calorimetry determined kcal.  Treadmill walking for 15 minutes at 3 different speeds (structured). | r=0.68-0.79 | Ainslie (2003) |
| Patrick Johansson et al. (2006)^[370]^ | Combined accelerometer and Heart Rate monitor | M: 32.0 ± 12.8 yrs.  F: 26.6 ± 5.3 yrs.  M: 28-63 yrs.  F: 28-30 yrs. | 27  8 | M = 14  F = 13  M=6  F=2 | Accelerometer regression equation determined total energy expenditure (MJ.d^-1^) – Doubly Labelled Water determined total energy expenditure (MJ.d^-1^).  Accelerometer regression equation determined physical activity energy expenditure (kJ.kg.d^-1^) – Doubly Labelled Water determined total energy expenditure (kJ.kg.d^-1^).  Participants wore the HRM and the ActiGraph MTI during model development with indirect Calorimetry while completing walking and running activities on the treadmill (Structured), while 8 participants wore HRM and ACC during free-living activities for 14 days with DLW measurement (Unstructured). | RMSE=2.99; Sig Diff from 0.  RMSE=34.1; Sig Diff from 0. | Van Remoortel (2012) |
| Patterson et al. (1993)^[387]^ | ActiGraph CSA | 22-38 yrs. | 15 | M = 9  F = 6 | ActiGraph determined counts.min^-1^ – Indirect Calorimetry determined energy expenditure (ml.kg^-1^.min^-1^)  ActiGraph determined counts.min^-1^ – Heart rate monitor determined beats per minute  Participants wore all devices while completing a range of activities in a laboratory (Structured). | r=0.73; p< 0.0001  r=0.71; p< 0.0001 | Prince (2008) |
| Paul et al. (2007)^[388]^ | Actical Mini-Mitter | 30-60 yrs. | 56 | M = 28  F = 28 | Actical determined counts.d^-1^ – ActiGraph determined counts.d^-1^.  Actical determined counts.d^-1^ (Log transformed) – ActiGraph determined counts.d^-1^ (Log transformed).  Participants wore both thee ActiGraph and the Actical for 13 days during free-living activities (Unstructured). | r=0.90; p< 0.001 CV%= 15.5%  r=0.90; p< 0.001 CV%= 3.1% | Pedisic (2014) |
| Pober et al. (2006)^[389]^ | ActiGraph (MTI) | 24.8±4.2 yrs. | 6 | M = 4  F = 2 | ActiGraph determined classification (Hidden Markov Method and Quadratic Discriminant Analysis) – Compendium of physical activity determined energy expenditure  7 minutes of structured activities, classification algorithms 1) Quadratic discriminant analysis, 2) Hidden Markov model and 3) Traditional Approach (Freedson cutpoints). | Accuracy QDA = 53.6-100%  Accuracy HMM = 62.5-97.3% | Bonomi (2012) |
| Pomeroy et al. (2011)^[390]^ | ActiGraph MTI | 20-34 yrs. | 50 | M = 25  F = 25 | MTI determined steps – Accusplit determined steps.  MTI determined steps – Dynastream determined steps.  Participants wore the devices while walking on a 540m laboratory walk test (Structured). | Absolute_DIFF_ Men = 6.0; IQR = 2.0-17.8  Signed_DIFF_ Men = -2.0; IQR = -10.5 – 1.1  Absolute_DIFF_ Women = 10.4; IQR = 7.1 – 16.1  Signed_DIFF_ Women = -7.7; IQR = -16.6 – -1.2  Absolute_DIFF_ Men = 4.9; IQR = 3.0 -9.0  Signed_DIFF_ Men = -0.0; IQR = -4.9 – 4.7  Absolute_DIFF_ Women = 6.2; IQR = 3.3 – 11.1  Signed_DIFF_ Women = 4.2; IQR = -2.1 – 8.7 | Plasqui (2013) |
| Pomeroy et al. (2011)^[390]^ | ActiGraph MTI | 20-34 yrs. | 50 | M = 25  F = 25 | MTI determined steps – Accusplit determined steps.  MTI determined steps – Dynastream determined steps.  Participants wore the devices during free-living settings for a 7 day period (Unstructured). | Absolute_DIFF_ Men = 20.2; IQR = 7.6-32.1  Signed_DIFF_ Men = -11.4; IQR = -30.4 – 2.2  Absolute_DIFF_ Women = 15.2; IQR = 8.6 – 26.3  Signed_DIFF_ Women = 7.9; IQR = -9.1 – 15.7  Absolute_DIFF_ Men = 17.4; IQR = 8.8 – 30.6  Signed_DIFF_ Men = -16.4; IQR = -22.9 – -7.6  Absolute_DIFF_ Women = 18.0; IQR = 13.9 – 28.0  Signed_DIFF_ Women = -18.7; IQR = -29.9 – -13.5 | Plasqui (2013) |
| Pomeroy et al. (2011)^[390]^ | Dynastream AMP | 20-34 yrs. | 50 | M = 25  F = 25 | Dynastream determined steps – MTI determined steps.  Dynastream determined steps – Accusplit determined steps.  Participants wore the devices while walking on a 540m laboratory walk test (Structured). | Absolute_DIFF_ Men = 4.9; IQR = 3.0 -9.0  Signed_DIFF_ Men = -0.0; IQR = -4.9 – 4.7  Absolute_DIFF_ Women = 6.2; IQR = 3.3 – 11.1  Signed_DIFF_ Women = 4.2; IQR = -2.1 – 8.7  Absolute_DIFF_ Men = 4.4; IQR = 1.6 -11.4  Signed_DIFF_ Men = -1.8; IQR = -6.4 – 1.3  Absolute_DIFF_ Women = 3.7; IQR = 0.8 – 9.5  Signed_DIFF_ Women = -0.6; IQR = -8.4 – 0.9 | Plasqui (2013) |
| Pomeroy et al. (2011)^[390]^ | Dynastream AMP | 20-34 yrs. | 50 | M = 25  F = 25 | Dynastream determined steps – MTI determined steps.  Dynastream determined steps – Accusplit determined steps.  Participants wore the devices during free-living settings for a 7 day period (Unstructured). | Absolute_DIFF_ Men = 17.4; IQR = 8.8 – 30.6  Signed_DIFF_ Men = -16.4; IQR = -22.9 – -7.6  Absolute_DIFF_ Women = 18.0; IQR = 13.9 – 28.0  Signed_DIFF_ Women = -18.7; IQR = -29.9 – -13.5  Absolute_DIFF_ Men = 26.6; IQR = 12.0 -41.5  Signed_DIFF_ Men = -26.6; IQR = -41.5 – 12.0  Absolute_DIFF_ Women = 22.5; IQR = 11.3 – 28.5  Signed_DIFF_ Women = -22.0; IQR = -28.5 – -9.6 | Plasqui (2013) |
| Ried-Larsen et al. (2012)^[391]^ | ActiGraph 7164 | 37.8±8 yrs. | 20 | NA | ActiGraph 7164 determined counts/min – Actigraph GT3X (low frequency extension) determined counts/min.  ActiGraph 7164 determined minutes LIPA – Actigraph GT3X (low frequency extension) determined minutes LIPA.  ActiGraph 7164 determined minutes MVPA – Actigraph GT3X (low frequency extension) determined minutes MVPA.  ActiGraph 7164 determined counts/min – Actigraph GT3X (normal frequency) determined counts/min.  ActiGraph 7164 determined minutes LIPA – Actigraph GT3X (low frequency extension) determined minutes LIPA.  ActiGraph 7164 determined minutes MVPA – Actigraph GT3X (low frequency extension) determined minutes MVPA.  24 hours of monitoring of free-living activity behaviours using 3 devices (unstructured). | Mean_DIFF_= 18 (95% CI: 7-28)  Mean_DIFF_= 23.3 (95% CI: 14.8-31.8)  Mean_DIFF_= 1.4 (95% CI: -1.2-4.0)  Mean_DIFF_= 3 (95% CI: -10-6)  Mean_DIFF_= 2.2 (95% CI: -10.9-6.6)  Mean_DIFF_= -1.7 (95% CI: -4.0-0.7) | Gorman (2014) |
| Rothney et al. (2007)^[392]^ | ActiGraph | 38.6±13.1 yrs. | 102 | M = 46  F = 56 | ActiGraph determined energy expenditure (Freedson equation and work energy theorem (ActiGraph software) – Whole room Calorimetry determined energy expenditure.  24 hours of monitoring in a whole room calorimeter (structured). | R^2^=0.79; Perc_DIFF_=17.3% | Bonomi (2012) |
| Rothney et al. (2007)^[392]^ | IDEEA | 38.6±13.1 yrs. | 102 | M = 46  F = 56 | IDEEA determined energy expenditure – Whole room Calorimetry determined energy expenditure.  24 hours of monitoring in a whole room calorimeter (structured). | R^2^=0.83; Perc_DIFF_=10.2% | Bonomi (2012) |
| Rothney et al. (2007)^[392]^ | IDEEA | 38.6±13.1 yrs. | 102 | M = 46  F = 56 | IDEEA determined raw signal (analysed using artificial neural networks) – Whole room Calorimetry determined energy expenditure.  24 hours of monitoring in a whole room calorimeter (structured). | R^2^=0.85; Perc_DIFF_=4.47% | Bonomi (2012) |
| Rothney et al. (2008)^[393]^ | Actical | 39.3 ± 12.9 yrs. | 85 | M = 37  F = 48 | Actical determined energy expenditure (Heil equation (single)) – Indirect Calorimeter determined energy expenditure.  Actical determined energy expenditure (Heil equation (double)) – Indirect Calorimeter determined energy expenditure.  Participants wore activity monitors while in a whole room calorimeter for a 24 hour period (Structured). | p<0.001  p<0.001 | Murphy (2009) |
| Rothney et al. (2008)^[393]^ | ActiGraph | 39.3 ± 12.9 yrs. | 85 | M = 37  F = 48 | ActiGraph determined energy expenditure (Freedson equation) – Indirect Calorimeter determined energy expenditure.  ActiGraph determined energy expenditure (Hendelman equation) – Indirect Calorimeter determined energy expenditure.  ActiGraph determined energy expenditure (Yngve equation) – Indirect Calorimeter determined energy expenditure.  Participants wore activity monitors while in a whole room calorimeter for a 24 hour period (Structured). | p<0.001  p<0.001  NS | Murphy (2009) |
| Rothney et al. (2008)^[393]^ | RT3 Triaxial accelerometer | 39.3 ± 12.9 yrs. | 85 | M = 37  F = 48 | RT3 determined energy expenditure (StayHealthy equation) – Indirect Calorimeter determined energy expenditure.  RT3 determined energy expenditure (Chen and Sun equation) – Indirect Calorimeter determined energy expenditure.  RT3 determined energy expenditure (Hendelman equation) – Indirect Calorimeter determined energy expenditure.  Participants wore activity monitors while in a whole room calorimeter for a 24 hour period (Structured). | p<0.001  NS  NS | Murphy (2009) |
| Rowlands et al. (2004)^[394]^ | RT3 | 20.7±1.4 yrs. | 15 | M = 15 | RT3 counts – Indirect Calorimetry (SVO2)  Walking and Running on a treadmill and some free-living physical activities (structured). | r_p_=0.89; p≤0.01  (free play)  r_p_=0.79; p≤0.01 (Treadmill) | De Vries (2006) |
| Sherman et al. (1998)^[395]^ | Tritrac R3D | NA | 16 | NA | Energy expenditure determined from Tritrac – Energy expenditure determined from indirect Calorimetry.  Treadmill ambulation at 3 different intensities (structured). | r=.96 | Ainslie (2003) |
| Slootmaker et al. (2009)^[396]^ | Physical Activity Monitor (PAM) | 21-54 yrs. | 32 | M = 14  F = 18 | PAM determined Score – ActiGraph (MTI) determined accelerometer counts.  PAM determined Score – Indirect Calorimetry determined ml/Kg/min.  PAM regression equation (determined from Score) – Indirect Calorimetry determined ml/Kg/min.  Treadmill walking and Stair Walking (structured) | R^2^_Treadmill_=0.95; p<0.01  R^2^_Stair_=0.65; p<0.01  R^2^_Treadmill_=0.93; p<0.01  R^2^_Stair_=0.74; p<0.01  Underestimated EE by 30% - 64% | Chinapaw (2010) |
| Slootmaker et al. (2009)^[396]^ | ActiGraph (MTI | 21-54 yrs. | 32 | M = 14  F = 18 | ActiGraph (MTI) determined accelerometer counts – Indirect Calorimetry determined ml/Kg/min.  ActiGraph regression equation (Freedson) – Indirect Calorimetry determined ml/Kg/min.  ActiGraph regression equation (Hendelman) – Indirect Calorimetry determined ml/Kg/min.  ActiGraph regression equation (Crouter) – Indirect Calorimetry determined ml/Kg/min.  Treadmill walking and Stair Walking (structured) | R^2^_Treadmill_=0.82; p<0.01  R^2^_Stair_=0.64; p<0.01  Underestimated EE by 25% - >50%  Underestimated EE by 23% - >50%  Underestimated EE by 3% - 45% | Chinapaw (2010) |
| Spierer et al. (2011)^[397]^ | ActiHeart | 26.4 ± 7.3 yrs. | 27 | M=16  F=11 | ActiHeart determined energy expenditure (acc counts only) (kJ.kg.min^-1^) – Indirect Calorimetry determined energy expenditure (kJ.kg.min^-1^).  ActiHeart determined energy expenditure (HR only) (kJ.kg.min^-1^) - Indirect Calorimetry determined energy expenditure (kJ.kg.min^-1^).  ActiHeart determined energy expenditure (HR and ACC) (kJ.kg.min^-1^) - Indirect Calorimetry determined energy expenditure (kJ.kg.min^-1^).  Participants completed a range of physical activities while wearing the activity monitors and the indirect calorimeter (Structured). | Mean_DIFF_=0.1196; RMSE = 0.1958  Mean_DIFF_=0.0487; RMSE = 0.1415  Mean_DIFF_=0.0699; RMSE = 0.1318 | Van Remoortel (2012) |
| Spierer et al. (2011)^[397]^ | Actical | 26.4 ± 7.3 yrs. | 27 | M=16  F=11 | Actical determined energy expenditure (acc counts only) (kJ.kg.min^-1^) - Indirect Calorimetry determined energy expenditure (kJ.kg.min^-1^).  Participants completed a range of physical activities while wearing the activity monitors and the indirect calorimeter (Structured). | Mean_DIFF_=0.0772; RMSE = 0.1124 | Van Remoortel (2012) |
| Staudenmayer et al. (2009)^[315]^ | ActiGraph 7164 | 35.0 (21-69) yrs. | 48 | M = 24  F = 24 | ActiGraph determined METs (using Artificial Neural Networks) – Indirect Calorimetry determined METs.  ActiGraph and Cosmed worn during the completion of activity routines in the laboratory (Structured). | RMSE = 1.22 (95% CI = 1.14 – 1.30) | Liu (2012) |
| Stone et al. (2007)^[398]^ | Actical | 17.6 ± 8 yrs. | 86 | M = NA  F = NA | Actical determined energy expenditure (kcal.min^-1^) – Indirect Calorimetry determined energy expenditure (kcal.min^-1^).  Actical determined energy expenditure (kcal.min^-1^) using research development regression equation – Indirect Calorimetry determined energy expenditure (kcal.min^-1^).  Participant wore device and had indirect Calorimetry measured while completing 3 treadmill walking/running speeds (Structured) | R^2^=0.88-94;  R^2^=0.90-95; %increase in accuracy=0-2% | Van Remoortel (2012) |
| Stone et al. (2007)^[398]^ | ActiGraph | 17.6 ± 8 yrs. | 86 | M = NA  F = NA | ActiGraph determined energy expenditure (kcal.min^-1^) – Indirect Calorimetry determined energy expenditure (kcal.min^-1^).  AG determined energy expenditure (kcal.min^-1^) using research development regression equation – Indirect Calorimetry determined energy expenditure (kcal.min^-1^).  Participant wore device and had indirect Calorimetry measured while completing 3 treadmill walking/running speeds (Structured) | R^2^=0.67-87;  R^2^=0.87-92; %increase in accuracy=5-20% | Van Remoortel (2012) |
| Stone et al. (2007)^[398]^ | RT3 accelerometer | 17.6 ± 8 yrs. | 86 | M = NA  F = NA | RT3 determined energy expenditure (kcal.min^-1^) – Indirect Calorimetry determined energy expenditure (kcal.min^-1^).  RT3 determined energy expenditure (kcal.min^-1^) using research development regression equation – Indirect Calorimetry determined energy expenditure (kcal.min^-1^).  Participant wore device and had indirect Calorimetry measured while completing 3 treadmill walking/running speeds (Structured) | R^2^=0.83 - 92;  R^2^=0.88-93; %increase in accuracy=1-5% | Van Remoortel (2012) |
| Strath et al. (2001)^[399]^ | ActiGraph CSA | M 33.1± 12.2 yrs.  F31.9± 13.1 yrs. | 30 | M = 16  F = 14 | CSA determined METs (Freedson equation) – Indirect Calorimetry determined METs.  CSA determined METs (Freedson equation) – Yamax Pedometer determined METs  CSA determined METs (Freedson equation) – Heart Rate determined METs  CSA determined METs (Freedson equation) – Combined CSA and heart rate monitoring determined METs  Continuous monitoring using HR, motion sensing, pedometers and indirect Calorimetry during laboratory based conditions (structured). | R^2^=0.536; p<0.01  R^2^=0.669; p<0.01  R^2^=0.349; p<0.01  R^2^=0.536; p<0.01 | Butte (2012) |
| Strath et al. (2003)^[194]^ | ActiGraph (MTI) | 20-31 yrs. | 10 | M = 4  F = 6 | Freedson determined METs – Indirect Calorimetry determined METs  Hendelman (1) determined METs – Indirect Calorimetry determined METs  Hendelman (2) determined METs – Indirect Calorimetry determined METs  Swartz determined METs – Indirect Calorimetry determined METs  Nichols determined METs – Indirect Calorimetry determined METs  5-6 hours of free-living physical activities (unstructured). | R^2^=0.08-0.45  R^2^=0.29-0.38  R^2^=0.05-0.46  R^2^=0.20-0.55  R^2^=0.03-0.48 | Cheung (2011) |
| Swan et al. (1997)^[400]^ | Caltrac | 22.6±5 yrs. | 31 | F = 31 | Caltrac determined Energy expenditure – Indirect Calorimetry (VO2)  Treadmill based walking, running and stepping (structured) | r=0.79-0.92 | Ainslie (2003) |
| Swartz et al. (2000)^[401]^ | ActiGraph (CSA) | 19-74 yrs. | 70 | M = 31  F = 39 | CSA determined METs (regression equations based on activity counts (hip)) – Indirect Calorimetry (METs)  Wide range of free living physical activities (structured) | r=0.56; p<0.001; R^2^=0.32; SEE=1.16 | Bassett (2000) Berlin (2006) |
| Swartz et al. (2000)^[401]^ | ActiGraph (CSA) | 19-74 yrs. | 70 | M = 31  F = 39 | CSA activity METs (regression equations based on activity counts (wrist)) – Indirect Calorimetry (METs)  Wide range of free living physical activities (structured) | r=0.18; p<0.01; R^2^=0.03; SEE=1.38 | Bassett (2000) Berlin (2006) |
| Swartz et al. (2000)^[401]^ | ActiGraph (CSA) | 19-74 yrs. | 70 | M = 31  F = 39 | CSA activity METs (regression equations based on activity counts (wrist and hip)) – Indirect Calorimetry (METs)  Wide range of free living physical activities (structured) | r=0.59; p<0.001; R^2^=0.34; SEE=1.14 | Bassett (2000) Berlin (2006) |
| Terrier et al. (2001)^[402]^ | Triaxial accelerometer (lower back) | NA | 12 | NA | Triaxial accelerometer determined mean of the integral of the vector magnitude – Indirect Calorimetry determined energy expenditure (VO_2_) (Separate analysis for each incline).  Triaxial accelerometer determined mean of the integral of the vector magnitude – Indirect Calorimetry determined energy expenditure (VO_2_) (Analysis of all data together).  Participants worn an accelerometer at the lower back and had indirect Calorimetry measured while walking on a treadmill at 3 different speeds at 7 different inclines (Structured). | r=0.87; p<0.001;  Not significantly correlated.  Device unable to differentiate between level and uphill/downhill walking. | Plasqui (2007) |
| Theou et al. (2012)^[173]^ | ActiTrainer | 63-90 yrs. | 50 | F = 50 | ActiTrainer determined time engaged in non-sedentary activities - HRM determined time engaged in non-sedentary activities.  ActiTrainer determined time engaged in non-sedentary activities - MLTPAQ total time spent across all activities.  10 hours of free-living activity behaviours (ActiTrainer and HRM) and 14 days of self-reported PA (MLTPAQ). | r_p_=0.26  r_p_=0.53; p<0.01 | Gorman (2014) |
| Thompson et al. (2006)^[403]^ | ActiHeart | 25 ± 5 yrs. | 20 | M = 10  F = 10 | HR+M determined energy expenditure (kJ.min^-1^) – Indirect Calorimetry determined energy expenditure (kJ.min^-1^).  Participants wore the ActiHeart and indirect calorimeter during 6 activities in the laboratory, with data extrapolated for an average 16 hour day (Structured). | Mean_DIFF_= -521 kJ; % _DIFF_=-6% | Warren (2010) |
| Van Hees et al. (2009)^[363]^ | DynaPort MiniMod | 22 ± 2 yrs. | 15 | F = 15 | Dynaport determined energy expenditure (based on developed models) – Whole room Calorimetry energy expenditure (J/min/kg).  Model developed prior to WRC (Structured). | R^2^=0.81  Within subjects  R^2^=0.70  Between subjects | Butte (2012) |
| Wanner et al. (2013)^[404]^ | ActiGraph GT3X+ | 40-80 yrs. | 65 | M = 32  F = 33 | ActiGraph GT3X+ (Normal Filtering) determined – minutes in LIPA ActiGraph GT3X+ (Low Frequency Filtering) determined minutes in LIPA.  ActiGraph GT3X+ (Normal Filtering) determined minutes in MVPA - ActiGraph GT3X+ (Low Frequency Filtering) determined minutes in MVPA.  ActiGraph GT3X+ (Normal Filtering) determined steps - ActiGraph GT3X+ (Low Frequency Filtering) determined steps.  ActiGraph GT3X+ (Normal Filtering) determined mean counts/min - ActiGraph GT3X+ (Low Frequency Filtering) determined mean counts/min.  8 days of free living activity monitoring wearing two devices with different filtering settings (unstructured). | r_s_=0.98; Mean_DIFF_=-13.4 ± 7.0 %  r_s_=0.98;  Mean_DIFF_=-21.3 ± 67.7 %  r_s_=0.93;  Mean_DIFF_=-108.4 ± 43.2 %  r_s_=0.99;  Mean_DIFF_=-13.7 ± 7.3 % | Gorman (2014) |
| Washburn et al (1989)^[261]^ | LSI Activity Monitor | 46.1±11.4 yrs. | 35 | M = 35 | LSI activity counts –Caltrac activity counts  Worn for a 3 day period examining free-living physical activity behaviours. | r=0.58 | Ainslie (2003) |
| Welk et al. (2000)^[406]^ | ActiGraph (CSA) | Mean=29yrs | 52 | M = 21  F = 31 | CSA determined METs (based on CSA count Freedson regression equation in lab experiment) – Indirect Calorimetry regression equation (VO2 (ml/kg/min^-1^)  CSA determined METs (based on CSA count Freedson regression equation in field experiment) – Indirect Calorimetry regression equation (VO2 (ml/kg/min^-1^)  6 activities designed to simulate lifestyle activitie, including 3 treadmill based walking speeds (structured) | r_llab_=0.85; R^2^=0.64; SEE=2.00  r_lfield_=0.48; R^2^=0.65; SEE=2.05 | Bassett (2000)  Berlin (2006) |
| Welk et al. (2000)^[406]^ | Tritrac | Mean=29yrs | 52 | M = 21  F = 31 | Tritrac determined METs (based on Vmag count Nichols equation in lab experiment) – Indirect Calorimetry (VO2 (ml/kg/min^-1^)  Tritrac determined METs (based on Vmag count Nichols equation in field experiment) – Indirect Calorimetry (VO2 (ml/kg/min^-1^)  6 activities designed to simulate lifestyle activitie, including 3 treadmill based walking speeds (structured) | r_llab_=0.93; R^2^=0.82; SEE=1.41  r_lfield_=0.59; R^2^=0.90; SEE=1.09 | Bassett (2000)  Berlin (2006) |
| Welk et al. (2000)^[406]^ | Biotrainer | Mean=29yrs | 52 | M = 21  F = 31 | Biotrainer determined METs (based on manufacturer equation in lab experiment) – Indirect Calorimetry (VO2 (ml/kg/min^-1^)  Biotrainer determined METs (based on manufacturer equation in field experiment) – Indirect Calorimetry (VO2 (ml/kg/min^-1^)  6 activities designed to simulate lifestyle activitie, including 3 treadmill based walking speeds (structured) | r_llab_=0.88; R^2^=0.54; SEE=2.21  r_lfield_=0.59; R^2^=0.50; SEE=2.41 | Bassett (2000)  Berlin (2006) |
| Welk et al. (2000)^[406]^ | ActiGraph (CSA) | Mean=29yrs | 52 | M = 21  F = 31 | CSA determined counts – Tritrac determined Vmag  6 activities designed to simulate lifestyle activities, including 3 treadmill based walking speeds (structured) | r_llab_=0.85  r_lfield_=0.61 | Bassett (2000)  Berlin (2006) |
| Welk et al. (2000)^[406]^ | ActiGraph (CSA) | Mean=29yrs | 52 | M = 21  F = 31 | CSA determined counts – Biotrainer determined kcals  6 activities designed to simulate lifestyle activities, including 3 treadmill based walking speeds (structured) | r_llab_=0.87  r_lfield_=0.68 | Bassett (2000)  Berlin (2006) |
| Welk et al. (2000)^[406]^ | Tritrac | Mean=29yrs | 52 | M = 21  F = 31 | Tritrac determined Vmag - CSA determined counts  6 activities designed to simulate lifestyle activities, including 3 treadmill based walking speeds (structured) | r_llab_=0.85  r_lfield_=0.61 | Bassett (2000)  Berlin (2006) |
| Welk et al. (2000)^[406]^ | Tritrac | Mean=29yrs | 52 | M = 21  F = 31 | Tritrac determined VM – Biotrainer determined kcals  6 activities designed to simulate lifestyle activities, including 3 treadmill based walking speeds (structured) | r_llab_=0.87  r_lfield_=0.80 | Bassett (2000)  Berlin (2006) |
| Welk et al. (2000)^[406]^ | Biotrainer | Mean=29yrs | 52 | M = 21  F = 31 | Biotrainer determined kcals – CSA determined counts  6 activities designed to simulate lifestyle activities, including 3 treadmill based walking speeds (structured) | r_llab_=0.88  r_lfield_=0.59 | Bassett (2000)  Berlin (2006) |
| Welk et al. (2000)^[406]^ | Biotrainer | Mean=29yrs | 52 | M = 21  F = 31 | Biotrainer determined kcals – Tritrac determined VM  6 activities designed to simulate lifestyle activities, including 3 treadmill based walking speeds (structured) | r_llab_=0.87  r_lfield_=0.80 | Bassett (2000)  Berlin (2006) |
| Welk et al. (2003)^[405]^ | Actitrac | 32.7 ± 10.3 yrs. | 181 | M = 97  F = 84 | Actitrac (Left hip) determined counts – Indirect Calorimetry determined METs (Development group)  Actitrac (Left hip) determined counts – Indirect Calorimetry determined VO2 ml.kg^-1^.min^-1^ (Development group)  Actitrac (Left hip) determined counts – Indirect Calorimetry determined kcal.min^-1^ (Development group)  Actitrac (Right hip) determined counts – Indirect Calorimetry determined METs (Development group)  Actitrac (Right hip) determined counts – Indirect Calorimetry determined VO2 ml.kg^-1^.min^-1^ (Development group)  Actitrac (Right hip) determined counts – Indirect Calorimetry determined kcal.min^-1^ (Development group)  Regression analysis to predict kcal.min^-1^ using Actitrac determined counts – Indirect Calorimetry in Validation group.  Actitrac determined counts – Indirect Calorimetry determined VO2 ml.kg^-1^.min^-1^ (Cross-validation group)  Participants walked/jogged for three 6 minute time intervals on a treadmill while wearing devices (Structured) | r=0.91  r=0.91  r=0.82  r=0.90  r=0.90  r=0.81  R^2^=0.91; SEE=1.24  r=0.94; Mean_DIFF_=NS | Murphy (2009) |
| Welk et al. (2003)^[405]^ | Biotrainer | 32.7 ± 10.3 yrs. | 181 | M = 97  F = 84 | Biotrainer (Left hip) determined counts – Indirect Calorimetry determined METs (Development group)  Biotrainer (Left hip) determined counts – Indirect Calorimetry determined VO2 ml.kg^-1^.min^-1^ (Development group)  Biotrainer (Left hip) determined counts – Indirect Calorimetry determined kcal.min^-1^ (Development group)  Biotrainer (Right hip) determined counts – Indirect Calorimetry determined METs (Development group)  Biotrainer (Right hip) determined counts – Indirect Calorimetry determined VO2 ml.kg^-1^.min^-1^ (Development group)  Biotrainer (Right hip) determined counts – Indirect Calorimetry determined kcal.min^-1^ (Development group)  Regression analysis to predict kcal.min^-1^ using Biotrainer determined counts – Indirect Calorimetry in Validation group.  Actitrac determined counts – Indirect Calorimetry determined VO2 ml.kg^-1^.min^-1^ (Cross-validation group)  Participants walked/jogged for three 6 minute time intervals on a treadmill while wearing devices (Structured) | r=0.88  r=0.88  r=0.78  r=0.85  r=0.85  r=0.74  R^2^=0.88; SEE=1.47  r=0.94; Mean_DIFF_=NS | Murphy (2009) |
| Welk et al. (2007)^[407]^ | ActiGraph (MTI) | 24.9±6.1 yrs. | 30 | M = 13  F = 17 | ActiGraph (MTI) determined energy expenditure METs (Freedson equation) – IDEEA determined energy expenditure METs  ActiGraph (MTI) determined energy expenditure METs (Work energy theorem) – IDEEA determined energy expenditure METs  ActiGraph (MTI) determined energy expenditure METs (Combined Work energy theorem and Freedson equation) – IDEEA determined energy expenditure METs  ActiGraph (MTI) determined energy expenditure METs (Hendelman Lifestyle equation) – IDEEA determined energy expenditure METs  ActiGraph (MTI) determined energy expenditure METs (Hendelman walking equation) – IDEEA determined energy expenditure METs  ActiGraph (MTI) determined energy expenditure METs (Swartz lifestyle equation) – IDEEA determined energy expenditure METs  1 days free-living physical activity behaviours measured using IDEEA and Actigraph | Mean_DIFF_=-0.38 METs  r=0.62-0.84  Mean_DIFF_=-1.10 – 0.46 METs; p<0.05;  r=0.71-0.85  Mean_DIFF_= 1.1 METs; p<0.05  r=0.63-0.85  Mean_DIFF_=0.10 METs  r=0.70-0.88  Mean_DIFF_=0.93 METs; p<0.05  r=0.67-0.87 | Corder (2007) |
| Welk et al. (2007)^[407]^ | Sensewear Pro Armband | 24.9±6.1 yrs. | 30 | M = 13  F = 17 | SWA determined energy expenditure METs (Original Software) – IDEEA determined energy expenditure METs  SWA determined energy expenditure METs (New Algorithms) – IDEEA determined energy expenditure METs  1 days free-living physical activity behaviours measured using IDEEA and Actigraph | Mean_DIFF_= 0.12 METs  r=0.42-0.62  Mean_DIFF_= 0.01 METs;  r=0.51-0.94 | Corder (2007) |
| Yngve et al. (2003)^[408]^ | ActiGraph MTI | M = 23.7 ± 2.6 yrs.  F = 23.1± 2.6 yrs. | 28 | M = 14  F = 14 | ActiGraph determined METs (based on regression equation from counts collected on the back) – Indirect Calorimetry determined energy expenditure (METs).  ActiGraph determined counts (based on regression equation from counts collected on the Hip) – Indirect Calorimetry determined energy expenditure (METs).  Walking at a normal pace, fast pave and jogging on a treadmill. | R^2^=0.86; SEE = 1.09  R^2^=0.85; SEE = 1.14 | Ellery (2014) |
| Yngve et al. (2003)^[408]^ | ActiGraph MTI | M = 23.7 ± 2.6 yrs.  F = 23.1± 2.6 yrs. | 28 | M = 14  F = 14 | ActiGraph determined counts (based on regression equation from counts collected on the back) – Indirect Calorimetry determined energy expenditure (METs).  ActiGraph determined counts (based on regression equation from counts collected on the Hip) – Indirect Calorimetry determined energy expenditure (METs).  Walking at a normal pace, fast pave and jogging on a track. | R^2^=0.89; SEE = 0.96  R^2^=0.86; SEE = 1.10 | Ellery (2014) |

AG = ActiGraph; 24PARQ = 24 Hour Physical Activity Recall Questionnaire; ANN = Artificial Neural Networks; HMM = Hidden Markov Models; QDA = Quadratic Discriminant Analysis; CSA = Computer Science Applications accelerometer; SPA = Sensewear Pro Armband; NL = New Lifestyles; HRM = Heart Rate Monitor; PARQ = Physical Activity Recall Questionnaire; MLTPAS = Minnesota Leisure Time Physical Activity Survey; M= Male; F = Female; METs = Metabolic Equivalents; EE = Energy Expenditure; AEE = Activity Energy Expenditure; TEE = Total Energy Expenditure; PAEE = Physical Activity Energy Expenditure; LIPA = Light Intensity Physical Activity; MPA = Moderate Intensity Physical Activity; MVPA = Moderate to Vigorous Intensity Physical Activity; VM = Vector Magnitude; PAL = Physical Activity Level; r = Correlation Coefficient; rp = Pearson’s Correlation; rs = Pearson’s Correlation; NS = Non-significant; MeanDIFF = Mean Difference; CI = Confidence Intervals; SEE = Standard Error of the Estimate; LoA = Limits of Agreement; ICC = Intraclass Correlation Coefficient; IQR = Interquartile Range.

**Supplementary Table 8:** Inter-instrument reliability of accelerometer/activity monitor determined physical activity/energy expenditure.

| **Author** | **Measure** | **Age Range** | **Sample Size** | **Sex** | **Details** | **Results** | **Primary Source** |
| --- | --- | --- | --- | --- | --- | --- | --- |
| Brage et al. (2003)^[336]^ | ActiGraph (CSA) | 22.7-30.0 yrs. | 12 | M = 12 | ActiGraph worn on left and right hip.  3 walking/running trials on treadmill, with a further over ground walking/running trial (Structured). | ICC=0.91 | Chen (2005) |
| Brage et al. (2006)^[409]^ | ActiHeart | 20-39 yrs. | 24 | M = 12  F = 12 | ActiHeart worn at 3^rd^ intercostal space (Upper) and just below the apex of the sternum (Lower) (Physical activity intensity determined by both devices) | Treadmill Test: Lower device PAI 8.6% (p<0.05) higher than upper device.  NS differences between two measures for EE at all speeds | Bonomi (2012) |
| Copeland and Esliger (2009)^[343]^ | ActiGraph 7164 | 64-77 yrs. | 38 | M = 18  F = 20 | Two devices worn side by side over the right hip | ICC=0.956; p<0.001 | Gorman (2014) |
| Horner et al. (2011)^[410]^ | 3DNX accelerometer | 22 ± 3 yrs. | 11 | M = 5  F = 6 | Participants completed two treadmill tests (3 walking, 3 jogging and 4 running speeds while wearing two devices side by side at the lower back. | ICC=0.90 – 1.00  CV% = 7.7% - 16.2% | Pedisic (2014) |
| Jakicic et al. (1999)^[369]^ | Tritrac R3D | 21.5±3.4 yrs. | 20 | M = 6  F = 14 | Tritrac worn on left hip versus right hip 5 different exercises for 10 minutes in duration (Structured). | r=0.56-0.96 | Ainslie (2003) |
| Kumahara et al. (2004)^[411]^ | Lifecorder | 37.8±12.4 yrs. | 88 | M = 33  F = 55 | Lifecorder devices placed on both the waist and the wrist  24 hour stay in a whole room calorimeter (Structured). | r=0.798; p<0.001 | Corder (2007 |
| Levine et al. (2008)^[300]^ | Physical Activity Monitor (PAMS) | NA | NA | NA | Relationship between 2 paired accelerometers positioned at the lower back | ICC=0.99 | Bonomi (2012) |
| McClain et al. (2007)^[412]^ | ActiGraph | 30.1±3.8 yrs. | 10 | M = 4  F = 6 | ActiGraph device worn on left and right hip for 24 hours of free-living physical activity  Activity Counts  Steps  Activity intensity classification (Freedson Thresholds) | ICC=0.97; APE=8.6%  ICC=0.99; APE=2.4%  ICC=0.98-0.99  APE=1.2-18.8% | Cheung (2011) |
| Meijer et al. (1991)^[413]^ | Tracmor | 22±1 yrs. | 4 | M = 2  F = 2 | Devices placed side by side on the same belt.  Walking on a treadmill at 3 speeds for 5 minutes in duration. | Mean _DIFF_=30% | Ainslie (2003) |
| Nichols et al. (1999)^[384]^ | Tritrac | 23.4 ± 2.9 yrs. | 60 | M = 30  F = 30 | Tritrac device worn on both the left and right hip.  Walking and jogging at 3 speeds for 5 minutes on a treadmill. | r=0.73-0.87 | Ainslie (2003) |
| Nichols et al. (2000)^[414]^ | ActiGraph CSA | M = 23.0 ± 2.9 yrs.  F = 22.9 ± 2.9 yrs. | 20 | M = 10  F = 10 | CSA worn on left hip versus right hip during 4 different walking/running speeds (structured)  Walking/Running at 3.2 km.hr^-1^.  Walking/Running at 6.4 km.hr^-1^.  Walking/Running at 6.4 km.hr^-1^ (5% gradient).  Walking/Running at 9.7 km.hr^-1^. | r=0.55  r=0.89  r=0.91  r=0.73 | Loprinzi (2012) |
| Pambianco et al. (1990)^[386]^ | Caltrac | 20-35 yrs. (overweight) | 20 | M = 10  F = 10 | Correlation between left and right hip.  Treadmill walking for 15 minutes at 3 different speeds (Structured). | r=0.94 | Ainslie (2003) |
| Powell et al. (2004)^[415]^ | RT3 accelerometer | 24 yrs. | 1 | F = 1 | Intermonitor CV during locomotion  Intermonitor CV during sit-stand transitions  4 different RT3 device worn at the same location by one individual completing 8 structured activities in 2 different trials separated by 2 days (Structured). | CV<6%  CV=8-25% | Rowlands (2007) |
| Reneman and Helmus (2010)^[416]^ | RT3 accelerometer | 36.1 ±9.4 yrs. | 6 | M = 3  F = 3 | 6 accelerometers worn simultaneously (3 on the left hip and 3 on the right hip) while walking at specific speeds on a treadmill. | r_p_=0.72-0.95  ICC=0.46-0.89 | Pedisic (2014) |
| Ryan et al. (2006)^[314]^ | activPAL | 34.5 ± 6.9 yrs. | 20 | M = 8  F = 12 | Reliability of activPAL for step number at the various walking speeds (4 devices worn on the upper and lower thigh of both left and right leg).  Treadmill Speed 0.9 m.s^-1^  Treadmill Speed 1.12 m.s^-1^  Treadmill Speed 1.33 m.s^-1^  Treadmill Speed 1.56 m.s^-1^  Treadmill Speed 1.78 m.s^-1^  Outdoor Speed Slow  Outdoor Speed Normal  Outdoor Speed Fast | ICC=0.99  ICC=1.00  ICC=0.99  ICC=1.00  ICC=0.99  ICC=0.99  ICC=0.99  ICC=0.99 | Tudor-Locke (2012) |
| Vanhelst et al. (2012)^[417]^ | ActiGraph | M: 26.3 ± 3.6 yrs.  F = 29.4 ± 3.8 yrs. | 15 | M = 8  F = 7 | 8 ActiGraph accelerometers worn on the back during free-living conditions for one day. | CV = 3% - 10.5% | Pedisic (2014) |
| Vanhelst et al. (2012)^[417]^ | RT3 accelerometer | M: 26.3 ± 3.6 yrs.  F = 29.4 ± 3.8 yrs. | 15 | M = 8  F = 7 | 5 RT3 accelerometers worn on the back during free-living conditions for one day. | CV = 12.6% - 35.5% | Pedisic (2014) |
| Welk et al. (2003)^[405]^ | Actitrac | 32.7 ± 10.3 yrs. | 181 | M = 97  F = 84 | Correlation between left and right hip.  3 mph  4 mph  5 mph  Participants walked/jogged for three 6 minute time intervals (Structured). | r=0.97  ICC = 0.80; 95%CI =0.67-0.87; Alpha = 0.84  ICC = 0.87; 95%CI =0.69-0.93; Alpha = 0.90  ICC = 0.40; 95%CI = -0.32-0.73; Alpha = 0.65 | Murphy (2009) |
| Welk et al. (2003)^[405]^ | Biotrainer | 32.7 ± 10.3 yrs. | 181 | M = 97  F = 84 | Correlation between left and right hip.  3 mph  4 mph  5 mph  Participants walked/jogged for three 6 minute time intervals (Structured). | r=0.93  ICC = 0.72; 95%CI =0.46-0.83; Alpha = 0.76  ICC = 0.61; 95%CI =0.23-0.79; Alpha = 0.71  ICC = 0.60; 95%CI = 0.30-0.75; Alpha = 0.65 | Murphy (2009) |
| Yngve et al. (2003)^[408]^ | ActiGraph MTI | M = 23.7 ± 2.6 yrs.  F = 23.1± 2.6 yrs. | 28 | M = 14  F = 14 | ActiGraph devices placed on the hip and lower back (during treadmill setting).  ActiGraph devices placed on the hip and lower back (during over ground setting). | r=0.97; p<0.001  r=0.96; p<0.001 | Ellery (2014) |

CSA = Computer Science Applications accelerometer; EE = Energy Expenditure; PAI = Physical Activity Index; ICC = Intraclass Correlation Coefficient; NS = Non-significant; r = Correlation Coefficient; rp = Pearson’s Correlation; APE = Average Percentage Error; CV = Coefficient of Variation; CI = Confidence Intervals.

**Supplementary Table 9.** Test-retest reliability of accelerometer/activity monitor determined physical activity/energy expenditure.

| **Author** | **Measure** | **Age Range** | **Sample Size** | **Sex** | **Details** | **Results** | **Primary Source** |
| --- | --- | --- | --- | --- | --- | --- | --- |
| Brandes et al. (2004)^[418]^ | Dynaport | 34 ±10 yrs. | 9 | M=7  F=2 | Dynaport determined activity behaviours measured at baseline and 21 days after initial administration. | r_s_=0.16-0.36; NS | Van Remoortel (2012) |
| Conger et al. (2005)^[281]^ | Fitsense FS1 (Speedometer) | 27.2±5.7 yrs. | 24 | M = 15  F = 9 | Walking 1600 metres on a track twice (Structured) | ICC_Walk_=0.84  ICC_RUN_=0.87 | Chen (2005) |
| Dahlgren et al. (2010)^[419]^ | activPAL | 19-28 yrs. | 24 |  | Self-selected walking (Steps)  Treadmill walking (3.2 km/h) (Steps)  Treadmill walking (4.5 km/h) (Steps)  Treadmill walking (4.5 km/h with incline) (Steps)  Treadmill jogging (8.0 km/h) (Steps)  Stair walking at self-selected pace (Steps)  Participants performed 9 activities wearing the activPAL and repeated them 1 week after initial assessment (Laboratory-based). | ICC=0.69 (95% CI =0.35-0.87; r_s_=0.68; SEM = 16 steps  ICC=0.88 (95% CI =0.70-0.95; r_s_=0.80; SEM = 6 steps  ICC=0.94 (95% CI =0.84-0.98; r_s_=0.89; SEM = 4 steps  ICC=0.95 (95% CI =0.87-0.98; r_s_=0.90; SEM = 4 steps  ICC=0.81 (95% CI =0.57-0.93; r_s_=0.80; SEM = 22 steps  ICC=0.70 (95% CI =0.36-0.88; r_s_=0.70; SEM = 17 steps | Lowe (2014) |
| Fruin et al. (2004)^[357]^ | Sensewear Pro Armband | 19-22 yrs. | 13 | M = 13 | SWA rest measured at 2 time points (period between tests not reported) (Laboratory based). | r=0.93; p<0.01 | Corder (2008) |
| Gao et al. (2008)^[420]^ | ActiWatch | 79.8 ±11.2 yrs. | 12 | NA | 3 days of monitoring repeated after 1 week (same days monitored). | ICC=0.978 (95% CI =0.925 – 0.994). | Ellery (2014) |
| Harris et al. (2009)^[421]^ | ActiGraph GT1M | ≥65 yrs. | 20 | NA | ActiGraph GT1M determined mean daily activity count.  ActiGraph GT1M determined mean daily step count.  ActiGraph GT1M worn at baseline and 6-8 weeks later. | r_p_=0.87; p<0.001  r_p_=0.78; p<0.001 |  |
| Hart et al. (2011)^[422]^ | ActiGraph 7164 | 69.3 ± 7.4 yrs. | 52 | M = 13  F = 39 | AG worn for 21 consecutive days during free-living physical activity behaviours (Unstructured).  Total Physical Activity | ICC_3d_=0.80; ICC_4d_=0.85; ICC_6d_=0.90;  ICC_13d_=0.95; | Pedisic (2014) |
| Horner et al. (2011)^[410]^ | 3DNX accelerometer | 22 ± 3 yrs. | 11 | M = 5  F = 6 | Participants completed two treadmill tests (3 walking, 3 jogging and 4 running speeds) completed no more than 5 days apart. | Mean Ratio Bias = -0.7%; 95% LoA = ±12.4%; NS. | Pedisic (2014) |
| Jacobs et al. (1993)^[227]^ | Caltrac | 20-59 yrs. | 78 | M = 28  F = 50 | Average of energy expenditure measured by the Caltrac in 14 2 day sessions measured over a full year.  Different month to month reliability examined. | r=0.69-0.84 | Bassett (2000) |
| Kwon et al. (2010)^[296]^ | IDEEA | 42.5 ± 13.0 yrs. | 12 | F=12 | Participants repeated postures and gait patterns during the same testing session.  Gait Counts  Posture | ICC=0.972-0.992  %Accuracy=0.98 | Van Remoortel (2012) |
| Levin et al (1999)^[233]^ | Caltrac | 37.4 ± 9.7 yrs. | 77 | M = 28  F = 49 | Caltrac worn for 48 hours prior to 14 clinic visits 26 days apart (MET minutes of activity per day). | ICC = 0.42 | LaMonte (2001) |
| Matthews et al. (2002)^[423]^ | ActiGraph CSA | M = 45.8 ± 16.2 yrs.  F = 44.8 ± 16.2 yrs. | 92 | M = 42  F = 50 | AG worn for 21 consecutive days during free-living physical activity behaviours (Unstructured).  Time spent in MVPA and Activity Counts | ICC_3d-4d_=0.80  ICC_7d_=0.90 | Pedisic (2014) |
| Meijer et al. (1991)^[413]^ | Tracmor | 22±1 yrs. | 4 | M = 2  F = 2 | Test retest reliability of 12 devices measured at baseline and after 1 week.  Walking on a treadmill at 3 speeds for 5 minutes in duration. | r=0.98 | Ainslie (2003) |
| Meriwether et al. (2006)^[152]^ | ActiGraph MTI | 18-64 yrs. | 68 | M = 10  F = 58 | 7 day free living activity recorded and repeated 7 days after initial administration. | r_s_=0.527; p<0.01 | Helmorhorst (2012) |
| Montoye et al. (1983)^[424]^ | Uniaxial Accelerometer | 20-60 yrs. | 4 | NA | Subjects completed 14 activities at baseline and after a 2 week period. | r=0.94; p<0.05; SEE = 179 units. | Mathie (2004) |
| Montoye et al. (1983)^[424]^ | LSI activity monitor (wrist) | 20-60 yrs. | 4 | NA | Subjects completed 14 activities at baseline and after a 2 week period. | r=0.74; SEE = 7.1 counts. | Mathie (2004) |
| Montoye et al. (1983)^[424]^ | LSI activity monitor (waist) | 20-60 yrs. | 4 | NA | Subjects completed 14 activities at baseline and after a 2 week period. | r=0.63; p<0.05; SEE = 15.5 counts. | Mathie (2004) |
| Nang et al. (2011)^[154]^ | Actical | NA | 43 | NA | Actical determined Vigorous Physical Activity (free living measurement for 5 consecutive days) examined a mean of 6 months apart.  Actical determined Moderate Physical Activity (free living measurement for 5 consecutive days) examined a mean of 6 months apart. | r_s_=0.68; p<0.001  r_s_=0.52; p<0.001 | Helmorhorst (2012) |
| Nichols et al. (1992)^[425]^ | Caltrac activity Monitor | 26.1 ± 1.1 yrs. | 7 | NA | Test-retest reliability of Caltrac examined within two weeks of initial assessment (Laboratory based). | r=0.95 | Strath (2012) |
| Nichols et al. (1992)^[425]^ | Caltrac activity Monitor | 64.8 ± 1.0 yrs. | 7 | NA | Test-retest reliability of Caltrac examined within two weeks of initial assessment (Laboratory based). | r=0.98 | Strath (2012) |
| Nichols et al. (1999)^[384]^ | Tritrac | 23.4 ± 2.9 yrs. | 20 | M = 10  F = 10 | Walking and jogging at 3 speeds for 5 minutes on a treadmill. Test protocol repeated after 2 weeks. | r=0.87-0.92 | Ainslie (2003) |
| Powell et al. (2004)^[415]^ | RT3 accelerometer | 24 yrs. | 1 | F = 1 | RT3 worn at baseline and 2 days after initial administration.  Vector Magnitude at rest  Vector Magnitude sit-stand  Vector Magnitude at 4km.h^-1^  Vector Magnitude at 6km.h^-1^  Vector Magnitude at 8km.h^-1^  Vector Magnitude at 10km.h^-1^ | No sig differences  No sig differences  Sig differences for all remaining activities between trial 1 and trial 2. | Rowlands (2007) |
| Rowe et al. (2007)^[426]^ | ActiGraph 7164 | 74.0 ±9.5 yrs. | 91 | M = 29  F = 60 | 7 day free living activity recorded and ICCs identified for each activity across number of days.  ActiGraph Steps  ActiGraph Counts (MVPA intermittent bouts)  ActiGraph Counts (MVPA 10 minutes continuous bouts) | ICC_2d_= 0.87; ICC_7d_= 0.93  ICC_2d_= 0.90; ICC_7d_= 0.93  ICC_2d_= 0.58 - 82; ICC_7d_= 0.86 - 0.91 | Gorman (2014) |
| Rowlands et al. (2007)^[313]^ | Actigraph | 23.1 ± 3.4 yrs. | 10 | M = 10 | Actigraph steps examined when walking and jogging at 11 speeds on a treadmill. Speeds ranged from 4-26 kmph. Duration between tests not provided. | ICC=0.84 – 0.98 | Corder (2007) |
| Rowlands et al. (2007)^[313]^ | ActPed | 23.1 ± 3.4 yrs. | 10 | M = 10 | ActPed steps examined when walking and jogging at 11 speeds on a treadmill. Speeds ranged from 4-26 kmph. Duration between tests not provided. | ICC=0.40 – 0.94 | Corder (2007) |
| Rowlands et al. (2007)^[313]^ | RT3 | 23.1 ± 3.4 yrs. | 10 | M = 10 | RT3 vector magnitude examined when walking and jogging at 11 speeds on a treadmill. Speeds ranged from 4-26 kmph. Duration between tests not provided. | ICC=0.51 – 0.94 | Corder (2007) |
| Sirard et al. (2011)^[427]^ | ActiGraph 7164 | 45.5 ± 12.61 yrs. | 158 | M = 56  F = 101 | Accelerometers worn for 7 days at baseline and 1-4 weeks after initial assessment. | ICC=0.77-0.90 | Pedisic (2014) |
| Sugimoto et al (1997)^[428]^ | ActiGraph Mini Motionlogger | 40.8 ± 13.3 yrs. | 4 | NA | Test-retest reliability of 4 tasks (trunk rotation exercise, body bending exercise in sitting position, walking at 3 km/h and walking at 5 km/h) completed twice within a laboratory setting. | ICC=0.91. | Prince (2008) |
| Washburn et al (1989)^[261]^ | LSI Activity Monitor | 46.1±11.4 yrs. | 35 | M = 35 | LSI activity counts examined physical activity behaviours for 3 days (2 week days, 1 weekend day) at baseline and after 3 months | r=0.58 | Ainslie (2003) |
| Washburn et al (1989)^[261]^ | Caltrac | 46.1±11.4 yrs. | 35 | M = 35 | Caltrac activity counts examined physical activity behaviours for 3 days (2 week days, 1 weekend day) at baseline and after 3 months | r=0.57 | Ainslie (2003) |
| Welk et al. (2000)^[406]^ | ActiGraph (CSA) | Mean=29yrs | 52 | M = 21  F = 31 | Average accelerometer counts assessed in 2 identical treadmill based activities completed | r=0.85 | Bassett (2000)  Berlin (2006) |
| Welk et al. (2000)^[406]^ | Tritrac | Mean=29yrs | 52 | M = 21  F = 31 | Average accelerometer counts assessed in 2 identical treadmill based activities completed | r=0.96 | Bassett (2000)  Berlin (2006) |
| Welk et al. (2000)^[406]^ | Biotrainer | Mean=29yrs | 52 | M = 21  F = 31 | Average accelerometer counts assessed in 2 identical treadmill based activities completed | r=0.89 | Bassett (2000)  Berlin (2006) |
| Welk et al. (2004)^[429]^ | ActiGraph CSA/MTI |  | 32 | M = 7  F = 25 | Participants completed 3 treadmill walking trials while wearing each device. | ICC = 0.80; G coefficient = 0.64; SEM = 348 | McClain (2009) |
| Welk et al. (2004)^[429]^ | BioTrainer |  | 34 | M = 11  F = 23 | Participants completed 3 treadmill walking trials while wearing each device. | ICC = 0.68; G coefficient = 0.557; SEM = 0.664 | McClain (2009) |
| Welk et al. (2004)^[429]^ | Actical |  | 38 | M = 12  F = 26 | Participants completed 3 treadmill walking trials while wearing each device. | ICC = 0.62; G coefficient = 0.432; SEM = 557 | McClain (2009) |
| Welk et al. (2004)^[429]^ | Tritrac R3D |  | 33 | M = 11  F = 22 | Participants completed 3 treadmill walking trials while wearing each device. | ICC = 0.73; G coefficient = 0.573; SEM = 184 | McClain (2009) |

AG = ActiGraph; METs = Metabolic Equivalents; MVPA = Moderate to Vigorous Intensity Physical Activity; r = Correlation Coefficient; rp = Pearson’s Correlation; rs = Pearson’s Correlation; ICC = Intraclass Correlation Coefficient; SEM = Standard Error of the Mean; CI = Confidence Intervals; NS = Non-significant; LoA = Limits of Agreement.

**Supplementary Table 10.** Sensitivity to change over time of accelerometer devices.

| Gao et al. (2008) ^[420]^ | ActiWatch | 79.8 ±11.2 yrs. | 12 | NA | 3 days of monitoring repeated after 1 week (same days monitored). | ICC=0.978 (95% CI =0.925 – 0.994). | Ellery (2014) |
| --- | --- | --- | --- | --- | --- | --- | --- |

ICC = Intraclass Correlation Coefficient; CI = Confidence Intervals.

**Supplementary Table 11.** Details of studies that examined the Criterion Validity of Pedometers.

| **Author** | **Measure** | **Age Range** | **Sample Size** | **Sex** | **Reference** | **Results** | **Primary Source** |
| --- | --- | --- | --- | --- | --- | --- | --- |
| Abel et al. (2008) ^[270]^ | Kenz Lifecorder EX | 29.4 ± 7.1 yrs. | 20 | M = 10  F = 10 | Kenz Lifecorder determined steps – Direct Observation determined steps (@ 54 m.min^-1^).  Kenz Lifecorder determined steps – Direct Observation determined steps (@ 80 m.min^-1^).  Kenz Lifecorder determined steps – Direct Observation determined steps (@ 107 m.min^-1^).  Kenz Lifecorder determined steps – Direct Observation determined steps (@ 134 m.min^-1^).  Kenz Lifecorder determined steps – Direct Observation determined steps (@ 161 m.min^-1^).  Kenz Lifecorder determined steps – Direct Observation determined steps (@ 188 m.min^-1^).  Participants completed 6 treadmill trials at 54, 80, 107, 134, 161 and 188 m.min^-1^ while wearing the devices (Structured). | Underestimated; p<0.01; r=-0.38  NS diff; r=-0.60; p<0.05  Underestimated; p<0.01; r=-0.66; p<0.05  Underestimated; p<0.001; r=-0.62; p<0.05  Underestimated; p<0.01; r=-0.50; p<0.05  Underestimated; p<0.01; r=-0.51; p<0.05 | Shephard (2012) |
| Ayabe et al. (2010) ^[431]^ | Kenz Lifecorder | 23 ± 2 yrs. | 7 | M = 7 | Kenz-Lifecorder determined steps – Direct Observation (during activities less than 3 METs)  Steps determined at different paces while treadmill walking at 3 different speeds. | %Error = -35 ± 18% | Kowalski (2012) |
| Ayabe et al. (2010) ^[431]^ | Digiwalker | 23 ± 2 yrs. | 7 | M = 7 | Digiwalker determined steps – Direct Observation (during activities less than 3 METs)    Steps determined at different paces while treadmill walking at 3 different speeds. | %Error = -56 ± 20% | Kowalski (2012) |
| Bassett et al. (1996) ^[450]^ | Pacer | 18-65 yrs. | 10 | M= 5  F = 5 | Pacer determined steps – Direct Observation determined steps.  Pacer worn while steps counted using hand tally counter while walking for 3.02 miles (Structured). | Mean%_Diff_= -12.5%  Difference calculated from article information. | Bassett (2000) |
| Bassett et al. (1996) ^[450]^ | Eddie Bauer | 18-65 yrs. | 10 | M= 5  F = 5 | Eddie Bauer determined steps – Direct Observation determined steps.  Eddie Bauer worn while steps counted using hand tally counter while walking for 3.02 miles (Structured). | Mean%Diff= -8.4%  Difference calculated from article information. | Bassett (2000) |
| Bassett et al. (1996) ^[450]^ | Digiwalker DW-500 | 18-65 yrs. | 10 | M= 5  F = 5 | Digiwalker determined steps – Direct Observation determined steps.  Digiwalker worn while steps counted using hand tally counter while walking for 3.02 miles (Structured). | Mean%Diff= -0.64%  Difference calculated from article information. | Bassett (2000) |
| Bergman et al. (2008) ^[432]^ | StepWatch 3 Step Activity Monitor | 65-91 yrs. | 21 | M = 5  F = 16 | StepWatch 3 determined steps – Direct Observation determined steps.  Walking trial of 161 metres while wearing the SW3 (structured). | Error Score=-11.33 (2.56); 95%Prediction Interval = -18.01 - -4.65  R^2^=0.99; p<0.001  Bland Altman | Kowlaski (2012) |
| Bergman et al. (2008) ^[432]^ | Digiwalker SW-200 | 65-91 yrs. | 21 | M = 5  F = 16 | Digiwalker determined steps – Direct Observation determined steps.  Walking trial of 161 metres while wearing the SW3 (structured). | Error Score = 208.43 (50.07); 95%Prediction Interval = 77.62 – 339.24;  R^2^=-0.08; p>0.05  Bland Altman | Kowlaski (2012) |
| Clemes et al. (2010) ^[433]^ | Silva model 56012 | 19.2±2.7 yrs. | 68 | NA | Silva determined steps (worn on both the left and right hip) – Direct observation  Walking on a treadmill at 5 speeds (structured) | Absolute percentage error = 6.7% - 49.6% | Bonomi (2012) |
| Clemes et al. (2010) ^[433]^ | New Lifestyles NL-1000 | 19.2±2.7 yrs. | 68 | NA | New Lifestyles determined steps – Direct observation  Walking on a treadmill at 5 speeds (structured) | Absolute percentage error = 0.5% - 12.9% | Bonomi (2012) |
| Connolly et al. (2011) ^[282]^ | Digiwalker SW200 | 30.6 ± 5.6 yrs. | 30 | F=30 | Digiwalker determined steps – Direct Observation determined steps.  Participants walked on a treadmill for 8-13 minutes wearing all devices and steps were recorded using direct observation (Structured). | %Accuracy=78.6 ± 29.6%  Bland Altman | Van Remoortel (2012) |
| Connolly et al. (2011) ^[282]^ | New Lifestyles NL 2000 | 30.6 ± 5.6 yrs. | 30 | F=30 | New Lifestyles determined steps – Direct Observation determined steps.  Participants walked on a treadmill for 8-13 minutes wearing all devices and steps were recorded using direct observation (Structured). | %Accuracy=103.3 ± 11.9%  Bland Altman | Van Remoortel (2012) |
| Connolly et al. (2011) ^[282]^ | Omron HJ-720ITC | 30.6 ± 5.6 yrs. | 30 | F=30 | Omron determined steps – Direct Observation determined steps.  Participants walked on a treadmill for 8-13 minutes wearing all devices and steps were recorded using direct observation (Structured). | %Accuracy=97.7 ± 7.4%  Bland Altman | Van Remoortel (2012) |
| Crouter et al. (2003) ^[448]^ | Kenz Lifecorder | 33 ± 12 yrs.. | 10 | M = 5  F = 5 | Kenz Lifecorder determined steps – Direct observation determined steps.  Participants walked on the treadmill at 54, 67, 80, 94 and 107 m.min^-1^ for 5 minutes while wearing devices and being observed (Structured). | Overestimation @ 107 m.min^-1^; p<0.05. | McClain (2009) |
| Crouter et al. (2003) ^[448]^ | New Lifestyles NL-2000 | 33 ± 12 yrs. | 10 | M = 5  F = 5 | New Lifestyles determined steps – Direct observation determined steps.  Participants walked on the treadmill at 54, 67, 80, 94 and 107 m.min^-1^ for 5 minutes while wearing devices and being observed (Structured). | Overestimation @ 80, 94 and 107 m.min^-1^; p<0.05. | McClain (2009) |
| Crouter et al. (2003) ^[448]^ | Digiwalker | 33 ± 12 yrs. | 10 | M = 5  F = 5 | Digiwalker determined steps – Direct observation determined steps.  Participants walked on the treadmill at 54, 67, 80, 94 and 107 m.min^-1^ for 5 minutes while wearing devices and being observed (Structured). | No significant differences between actual and measured steps. | McClain (2009) |
| Crouter et al. (2003) ^[448]^ | Sportline 330 | 33 ± 12 yrs. | 10 | M = 5  F = 5 | Sportline determined steps – Direct observation determined steps.  Participants walked on the treadmill at 54, 67, 80, 94 and 107 m.min^-1^ for 5 minutes while wearing devices and being observed (Structured). | Underestimation @ 54 m.min^-1^; p<0.05 | McClain (2009) |
| Crouter et al. (2003) ^[448]^ | Omron HJ-105 | 33 ± 12 yrs. | 10 | M = 5  F = 5 | Omron -105 determined steps – Direct observation determined steps.  Participants walked on the treadmill at 54, 67, 80, 94 and 107 m.min^-1^ for 5 minutes while wearing devices and being observed (Structured). | Overestimation @ 107 m.min^-1^; p<0.05. | McClain (2009) |
| Crouter et al. (2003) ^[448]^ | Freestyle Pacer Pro | 33 ± 12 yrs. | 10 | M = 5  F = 5 | Freestyle Pacer Pro determined steps – Direct observation determined steps.  Participants walked on the treadmill at 54, 67, 80, 94 and 107 m.min^-1^ for 5 minutes while wearing devices and being observed (Structured). | Overestimation @ 54 m.min^-1^; p<0.05. | McClain (2009) |
| Crouter et al. (2003) ^[448]^ | Oregon Scientific PE316CA | 33 ± 12 yrs. | 10 | M = 5  F = 5 | Oregon Scientific determined steps – Direct observation determined steps.  Participants walked on the treadmill at 54, 67, 80, 94 and 107 m.min^-1^ for 5 minutes while wearing devices and being observed (Structured). | Overestimation @ all speeds; p<0.05. | McClain (2009) |
| Crouter et al. (2003) ^[448]^ | Sportline 345 | 33 ± 12 yrs. | 10 | M = 5  F = 5 | Sportline determined steps – Direct observation determined steps.  Participants walked on the treadmill at 54, 67, 80, 94 and 107 m.min^-1^ for 5 minutes while wearing devices and being observed (Structured). | Underestimation @ 54 and 67 m.min^-1^; p<0.05. | McClain (2009) |
| Crouter et al. (2003) ^[448]^ | Walk4Life LS 2525 | 33 ± 12 yrs. | 10 | M = 5  F = 5 | Walk4Life determined steps – Direct observation determined steps.  Participants walked on the treadmill at 54, 67, 80, 94 and 107 m.min^-1^ for 5 minutes while wearing devices and being observed (Structured). | Overestimation @ 94 and 107 m.min^-1^; p<0.05. | McClain (2009) |
| Crouter et al. (2003) ^[448]^ | Yamax Skeletone EM 180 | 33 ± 12 yrs. | 10 | M = 5  F = 5 | Skeletone determined steps – Direct observation determined steps.  Participants walked on the treadmill at 54, 67, 80, 94 and 107 m.min^-1^ for 5 minutes while wearing devices and being observed (Structured). | Underestimation @ 54 and 67 m.min^-1^.  Overestimation @ 107 m.min^-1^; p<0.05. | McClain (2009) |
| Crouter et al. (2005) ^[434]^ | Digiwalker SW 200 | 40±13 yrs. | 40 | M = 20  F = 20 | Digiwalker determined steps (in obese individuals) – Direct observation  Walking and running on a treadmill for 3 minutes at 5 different velocities. | Significant underestimation (value NR).  Bland Altman | Bonomi (2012) |
| Crouter et al. (2005) ^[434]^ | New Lifestyles NL-2000 | 40±13 yrs. | 40 | M = 20  F = 20 | New Lifestyles determined steps (in obese individuals) – Direct observation  Walking and running on a treadmill for 3 minutes at 5 different velocities (structured). | Significant underestimation  MeanDIFF=3-7%.  Bland Altman | Bonomi (2012) |
| Cyarto et al (2004) ^[435]^ | Digiwalker DW-200 | 79.4 ± 8.2 yrs. | 26 | M = 5  F = 21 | Digiwalker determined steps – Direct observation determined steps.  Walking trial of 13 metre track at 3 different speeds in nursing home individuals (structured). | %Error Slow = -73.9 ± 34.8  %Error Moderate = -55.1 ± 37.8  %Error Fast = -46.3 ± 38.1 | Kowlaski (2012) |
| Cyarto et al (2004) ^[435]^ | Digiwalker DW-200 | 70.6 ± 5.5 yrs. | 28 | M = 5  F = 23 | Digiwalker determined steps – Direct observation determined steps.  Walking trial of 13 metre track at 3 different speeds in community dwelling individuals (structured). | %Error Slow = -24.7 ± 36.1  %Error Moderate = -13.3 ± 23.6  %Error Fast = -7.1 ± 26.2 | Kowlaski (2012) |
| Foster et al. (2005) ^[436]^ | Accusplit | 21-51 yrs | 20 | M = 10  F = 10 | Accusplit determined step counts – Direct observation  Standing Stationary and walking on a treadmill at 1, 2 and 3 mph for 15 minutes (structured). | Accuracy = 26 (SEM 2.8%) | Corder (2007) |
| Foster et al. (2005) ^[436]^ | Omron HF-100 | 21-51 yrs | 20 | M = 10  F = 10 | Omron determined step counts – Direct observation  Standing Stationary and walking on a treadmill at 1, 2 and 3 mph for 15 minutes (structured). | Accuracy = 61 (SEM 3.3%) | Corder (2007) |
| Foster et al. (2005) ^[436]^ | StepWatch | 21-51 yrs | 20 | M = 10  F = 10 | StepWatch determined step counts – Direct observation  Standing Stationary and walking on a treadmill at 1, 2 and 3 mph for 15 minutes (structured). | Accuracy = 99.7 (SEM 0.67%) | Corder (2007) |
| Grant et al. (2008) ^[288]^ | Digiwalker SW-200 | 65-87 yrs. | 21 | M = 10  F = 11 | Digiwalker determined step count – Direct Observation determined step count  Walking on a treadmill at 5 different speeds and over ground walking on an outdoor track (Structured). | Mean_DIFF_=184.3 (LoA = -139.6 – 508.2).  Absolute % Error <2%  Bland Altman | Kowlaski (2012) |
| Grant et al. (2008) ^[288]^ | New-Lifestyles NL-2000 | 65-87 yrs. | 21 | M = 10  F = 11 | New-Lifestyles NL-2000determined step count – Direct Observation determined step count  Walking on a treadmill at 5 different speeds and over ground walking on an outdoor track (Structured). | Mean_DIFF_=85.4 (LoA = -84.8 – 255.6).  Absolute % Error <2%  Bland Altman | Kowlaski (2012) |
| Hasson et al. (2009) ^[437]^ | Digiwalker | 29 ±11 yrs. | 92 | M = 44  F = 48 | Digiwalker determined steps (hip) – Direct Observation determined steps (BMI<30).  Speed =1.12 m.s^-1^  Speed =1.34 m.s^-1^  Speed =1.56 m.s^-1^  Yamax determined steps (hip) – Direct Observation determined steps (BMI>30).  Speed =1.12 m.s^-1^  Speed =1.34 m.s^-1^  Speed =1.56 m.s^-1^ | %Bias=-2.0 ± 9.4  %Bias=0.0 ± 6.6  %Bias=0.9 ± 3.1  %Bias=-3.0 ± 5.0  %Bias=-0.3 ± 5.3  %Bias=-1.0 ± 7.2 | Tudor-Locke (2012) |
| Hasson et al. (2009) ^[437]^ | Omron HJ-112 | 29 ±11 yrs. | 92 | M = 44  F = 48 | Omron determined steps (hip) – Direct Observation determined steps (BMI<30).  Speed =1.12 m.s-1  Speed =1.34 m.s-1  Speed =1.56 m.s-1  OM determined steps (hip) – Direct Observation determined steps (BMI>30).  Speed =1.12 m.s-1  Speed =1.34 m.s-1  Speed =1.56 m.s-1 | %Bias=0.5 ± 2.7  %Bias=0.2 ± 2.0  %Bias=0.2 ± 2.0  %Bias=0.1 ± 3.8  %Bias=0.2 ± 3.5  %Bias=0.0 ± 5.0 | Tudor-Locke (2012) |
| Hendelman et al. (2000) ^[364]^ | Digiwalker | 30-50 yrs. | 25 | M = 10  F = 15 | Digiwalker determined step counts – Direct Observation  Over ground self-paced walking (5minutes in duration), played 2 holes of golf and performed household tasks (structured). | r=0.84 | Westerterp (2009) |
| Holbrook et al. (2009) ^[446]^ | Omron HJ-151 | M: 26 ± 6.5 yrs.  F: 22.1 ± 2.1 yrs. | 47 | M = 24  F = 23 | Omron HJ151 determined steps – Direct Observation determined steps (results presented for APE across all placements and all speeds).  Omron HJ151 worn at mid back, right and left hip. Participants walked at 3 prescribed speeds and 1 self-paced speed over a 100 m walking track (Structured). | Actual%_ERROR_= 1.4% ± 1.7% | Tudor-Locke (2012) |
| Holbrook et al. (2009) ^[446]^ | Omron HJ-720ITC | M: 26 ± 6.5 yrs.  F: 22.1 ± 2.1 yrs. | 47 | M = 24  F = 23 | Omron HJ-720ITC determined steps – Direct Observation determined steps (results presented for APE across all placements and all speeds).  Omron HJ151 worn at mid back, right and left hip. Participants walked at 3 prescribed speeds and 1 self-paced speed over a 100 m walking track (Structured). | Actual%_ERROR_= 2.3% ± 2.8% | Tudor-Locke (2012) |
| Hoyt et al. (2004) ^[453]^ | Foot-ground contact pedometer | 27 ± 4 yrs. | 8 | M = 8 | Pedometer determined energy expenditure (MJ.d-1) – Doubly labelled water determined energy expenditure (MJ.d-1).  Participants wore the pedometer for the 2 days during DLW measurement period (Unstructured). | Mean_DIFF_=0.02 ± 1.83 MJ/d.  Ab_DIFF_=1.55 ± 0.77  Mean_BIAS_= 0.02 MJ; Mean_ERROR_=1.83 MJ.  Bland Altman | Plasqui (2007) |
| Karabulut et al. (2005) ^[438]^ | StepWatch 3 (ankle) | 28 ± 3.7 yrs. | 20 | M = 10  F = 10 | Stepwatch 3 determined steps at 27 m.min^-1^ – Direct observation determined steps.  Stepwatch 3 determined steps at 40 m.min^-1^ – Direct observation determined steps.  Stepwatch 3 determined steps at 54 m.min^-1^ – Direct observation determined steps.  Stepwatch 3 determined steps at 67 m.min^-1^ – Direct observation determined steps.  Stepwatch 3 determined steps at 80 m.min^-1^ – Direct observation determined steps.  Stepwatch 3 determined steps at 107 m.min^-1^ – Direct observation determined steps.  Participants walked on a treadmill wearing devices while being observed (Structured). | p<0.05  p<0.05  NS  NS  NS  NS  Bland Altman | Murphy (2009) |
| Karabulut et al. (2005) ^[438]^ | Activity Monitor Pod 331 (ankle) | 28 ± 3.7 yrs. | 20 | M = 10  F = 10 | AMP 331 determined steps at 27 m.min^-1^ – Direct observation determined steps.  AMP 331 determined steps at 40 m.min^-1^ – Direct observation determined steps.  AMP 331 determined steps at 54 m.min^-1^ – Direct observation determined steps.  AMP 331 determined steps at 67 m.min^-1^ – Direct observation determined steps.  AMP 331 determined steps at 80 m.min^-1^ – Direct observation determined steps.  AMP 331 determined steps at 107 m.min^-1^ – Direct observation determined steps.  Participants walked on a treadmill wearing devices while being observed (Structured). | p<0.05  NS  NS  NS  NS  NS  Bland Altman | Murphy (2009) |
| Karabulut et al. (2005) ^[438]^ | New Lifestyles NL-2000 (wrist) | 28 ± 3.7 yrs. | 20 | M = 10  F = 10 | New Lifestyles -2000 determined steps at 27 m.min^-1^ – Direct observation determined steps.  New Lifestyles -2000 determined steps at 40 m.min^-1^ – Direct observation determined steps.  New Lifestyles -2000 determined steps at 54 m.min^-1^ – Direct observation determined steps.  New Lifestyles -2000 determined steps at 67 m.min^-1^ – Direct observation determined steps.  New Lifestyles -2000 determined steps at 80 m.min^-1^ – Direct observation determined steps.  New Lifestyles -2000 determined steps at 107 m.min^-1^ – Direct observation determined steps.  Participants walked on a treadmill wearing devices while being observed (Structured). | p<0.05  p<0.05  NS  NS  NS  NS  Bland Altman | Murphy (2009) |
| Karabulut et al. (2005) ^[438]^ | Digiwalker SW-701 (wrist) | 28 ± 3.7 yrs. | 20 | M = 10  F = 10 | Digiwalker determined steps at 27 m.min^-1^ – Direct observation determined steps.  Digiwalker determined steps at 40 m.min^-1^ – Direct observation determined steps.  Digiwalker determined steps at 54 m.min^-1^ – Direct observation determined steps.  Digiwalker determined steps at 67 m.min^-1^ – Direct observation determined steps.  Digiwalker determined steps at 80 m.min^-1^ – Direct observation determined steps.  Digiwalker determined steps at 107 m.min^-1^ – Direct observation determined steps.  Participants walked on a treadmill wearing devices while being observed (Structured). | NS  NS  p<0.05  p<0.05  p<0.05  p<0.05  Bland Altman | Murphy (2009) |
| Leenders et al. (2001) ^[92]^ | Digiwalker | 25.8 ± 1.6 | 13 | F = 13 | Digiwalker determined steps/d PAEE - Doubly labelled water PAEE.  7 days of activity behaviours measurement compared with 7 days doubly labelled water. | r=0.42; Mean_DIFF_ =59% | Ainslie (2003) |
| Le Masurier et al. (2004) ^[297]^ | Digiwalker | 20-55 yrs. | 12 | M = 6  F = 6 | Digiwalker steps – Direct Observation  Treadmill walking at 5 different speeds (structured). | Diff_Lab_>0.05 | Berlin (2006) |
| Le Masurier et al. (2004) ^[297]^ | Omron HJ-105 | 20-55 yrs. | 12 | M = 6  F = 6 | Omron steps – Direct Observation  Treadmill walking at 5 different speeds (structured). | Diff_Lab_>0.05 | Berlin (2006) |
| Le Masurier et al. (2004) ^[297]^ | SportLine 330 | 20-55 yrs. | 12 | M = 6  F = 6 | SportLine steps – Direct Observation  Treadmill walking at 5 different speeds (structured). | Diff_Lab_<0.05 (all speeds) | Berlin (2006) |
| Le Masurier et al. (2004) ^[297]^ | Digiwalker | 20-55 yrs. | 20 | M = 13  F = 7 | Digiwalker steps – Direct Observation  Treadmill walking at 5 different speeds (structured). | Diff_Lab_<0.05 (at 54m/min only)  Diff_Lab_>0.05 all remaining activities | Berlin (2006) |
| Maddocks et al. (2010) ^[305]^ | Digiwalker | 28 ± 8 yrs. | 40 | M = 15  F = 25 | Digiwalker determined steps – Direct Observation determined steps.  Treadmill Speed 0.6 m.s^-1^  Treadmill Speed 0.8 m.s^-1^  Treadmill Speed 1.0 m.s^-1^  Treadmill Speed 1.2 m.s^-1^  Treadmill Speed 1.4 m.s^-1^  Motor vehicle transport  Participants walked on a treadmill at 5 different speeds and on a 500m outdoor course (at 3 different speeds) while wearing the devices and being video observed (Structured). | Mean%_Error_=40.4; 95% CI = 23.9-60  Mean%_Error_=16.4; 95% CI = 9.0-23.9  Mean%_Error_=5.8; 95% CI = 3.3-8.3  Mean%_Error_=6.4; 95% CI = 2.8-10.4  Mean%_Error_=3.7; 95% CI = 0.3-7.8  Mean_Error_=25 steps | Tudor-Locke (2012) |
| Marsh et al. (2007) ^[306]^ | Accusplit Eagle 120 | 70-85 yrs. | 29 | M = 9  F = 20 | Accusplit Eagle -120 determined step count – Direct Observation determined step count.  Assessment completed during 131 metres of walking around an indoor track. | %Error = 10.3 ± 25.4  r=0.508; p<0.005  Bland Altman | Kowalski (2012) |
| Marsh et al. (2007) ^[306]^ | New Lifestyles NL-2000 | 70-85 yrs. | 29 | M = 9  F = 20 | New Lifestyles -2000 determined step count – Direct Observation determined step count.  Assessment completed during 131 metres of walking around an indoor track. | %Error = 1.7 ± 2.5  r=0.980; p<0.001  Bland Altman | Kowalski (2012) |
| McClain et al. (2010) ^[451]^ | New Lifestyles NL-1000 | 27.3 ± 7.1 yrs. | 26 | M = 9  F = 17 | New Lifestyles determined steps – Direct Observation determined steps  Devices worn during 10 treadmill based walking and running speeds and for the remainder of the day (Structured + Unstructured). | Mean%_DIFF_=-7.7%  Difference calculated from article information. | Tudor-Locke (2012) |
| McClain et al. (2010) ^[451]^ | Walk4Life Pro | 27.3 ± 7.1 yrs. | 26 | M = 9  F = 17 | Walk4Life determined steps – Direct Observation determined steps  Devices worn during 10 treadmill based walking and running speeds and for the remainder of the day (Structured + Unstructured). | Mean%_DIFF_= -15.3%  Difference calculated from article information. | Tudor-Locke (2012) |
| McClain et al. (2010) ^[451]^ | Omron HJ-151 | 27.3 ± 7.1 yrs. | 26 | M = 9  F = 17 | Omron determined steps – Direct Observation determined steps  Devices worn during 10 treadmill based walking and running speeds and for the remainder of the day (Structured + Unstructured). | Mean%_DIFF_= 0.2%  Difference calculated from article information. | Tudor-Locke (2012) |
| McClain et al. (2010) ^[451]^ | Digiwalker | 27.3 ± 7.1 yrs. | 26 | M = 9  F = 17 | Digiwalker determined steps – Direct Observation determined steps  Devices worn during 10 treadmill based walking and running speeds and for the remainder of the day (Structured + Unstructured). | Mean%_DIFF_=-14.8%  Difference calculated from article information.  Bland Altman | Tudor-Locke (2012) |
| Melanson et al. (2004) ^[439]^ | Digiwalker SW 200 | 19-85 yrs. | 259 | M = 108  F = 151 | Digiwalker determined steps– Direct observation  Walking on a treadmill at 2 self-selected speeds (structured). | Accuracy = 61.1%-105.5% | Bonomi (2012) |
| Park et al. (2011) ^[440]^ | Kenz Lifecorder | 23-41 yrs. | 18 | M = 9  F = 9 | Kenz Lifecorder determined steps – Direct Observation determined steps.  Participants wore the devices and an indirect calorimeter while walking at 3 speeds on a treadmill (Structured). | Significant Underestimation of Steps counts; | Shephard (2012) |
| Pomeroy et al. (2011) ^[390]^ | Accuspilt AX120 | 20-34 yrs. | 50 | M = 25  F = 25 | Accusplit determined steps – Direct Observation determined steps.  Participants wore the devices while walking on a 540m laboratory walk test (Structured). | AbsoluteDIFF Men = 3.6; IQR = 1.3 -11.7  SignedDIFF Men = 0.1; IQR = -5.6 – 1.6  AbsoluteDIFF Women = 4.4; IQR = 0.5 – 10.8  SignedDIFF Women = -1.6; IQR = -8.8 – 0.1 | Plasqui (2013) |
| Resnick et al. (2001) ^[449]^ | Step Activity Monitor | 86 ± 6.1 yrs. | 30 | M = 8  F = 22 | Step Activity Monitor determined steps – Direct observation determined steps.  Step Activity Monitor and direct observation conducted indoors over a track of 0.4 miles in length (structured). | %Accuracy = 96%  Mean_%Error_ = 4.0 ± 3.1 | Kowalski (2012) |
| Rowlands et al. (2007) ^[313]^ | Digiwalker | 23.1 ± 3.4 yrs. | 10 | M = 10 | Digiwalker determined steps – Direct Observation  Walking and jogging at 11 speeds on a treadmill. Speeds ranged from 4-26 kmph (structured). | Sig Diff; p<0.05  r= 0.97; p<0.01 | Corder (2007) |
| Schneider et al. (2003) ^[445]^ | Digiwalker | M = 34.7 ± 12.6 yrs.  F = 43.1 ± 19.9 yrs. | 20 | M = 10  F = 10 | Digiwalker determined steps – Direct Observation.  Walking over a 400 metre track (structured). | Accuracy= ±3% (95% of the time)  Bland Altman | Butte (2012) |
| Schneider et al. (2003) ^[445]]^ | Kenz Lifecorder | M = 34.7 ± 12.6 yrs.  F = 43.1 ± 19.9 yrs. | 20 | M = 10  F = 10 | Kenz Lifecorder determined steps – Direct Observation.  Walking over a 400 metre track (structured). | Accuracy= ±3% (95% of the time)  Bland Altman | Butte (2012) |
| Schneider et al. (2003) ^[445]^ | New Lifestyles NL-2000 | M = 34.7 ± 12.6 yrs.  F = 43.1 ± 19.9 yrs. | 20 | M = 10  F = 10 | New Lifestyles NL determined steps – Direct Observation.  Walking over a 400 metre track (structured). | Accuracy= ±3% (95% of the time)  Bland Altman | Butte (2012) |
| Schneider et al. (2003) ^[445]^ | Sportline 330 | M = 34.7 ± 12.6 yrs.  F = 43.1 ± 19.9 yrs. | 20 | M = 10  F = 10 | Sportline 330 determined steps – Direct Observation.  Walking over a 400 metre track (structured). | Accuracy= ±37% (95% of the time)  Bland Altman | Butte (2012) |
| Schneider et al. (2003) ^[445]^ | Omron HJ-105 | M = 34.7 ± 12.6 yrs.  F = 43.1 ± 19.9 yrs. | 20 | M = 10  F = 10 | Omron HJ-105 determined steps – Direct Observation.  Walking over a 400 metre track (structured). | Accuracy= ±37% (95% of the time)  Bland Altman | Butte (2012) |
| Schneider et al. (2003) ^[445]^ | Freestyle Pacer Pro | M = 34.7 ± 12.6 yrs.  F = 43.1 ± 19.9 yrs. | 20 | M = 10  F = 10 | Freestyle Pacer Pro determined steps – Direct Observation.  Walking over a 400 metre track (structured). | Accuracy= ±20% (95% of the time)  Bland Altman | Butte (2012) |
| Schneider et al. (2003) ^[445]^ | Oregon Scientific PE316CA | M = 34.7 ± 12.6 yrs.  F = 43.1 ± 19.9 yrs. | 20 | M = 10  F = 10 | Oregon Scientific determined steps – Direct Observation.  Walking over a 400 metre track (structured). | Accuracy= ±20% (95% of the time)  Bland Altman | Butte (2012) |
| Schneider et al. (2003) ^[445]^ | Sportline 345 | M = 34.7 ± 12.6 yrs.  F = 43.1 ± 19.9 yrs. | 20 | M = 10  F = 10 | Sportline 345 determined steps – Direct Observation.  Walking over a 400 metre track (structured). | Accuracy= ±20% (95% of the time)  Bland Altman | Butte (2012) |
| Schneider et al. (2003) ^[445]^ | Walk4Life LS 2525 | M = 34.7 ± 12.6 yrs.  F = 43.1 ± 19.9 yrs. | 20 | M = 10  F = 10 | Walk4Life determined steps – Direct Observation.  Walking over a 400 metre track (structured). | Accuracy= ±20% (95% of the time)  Bland Altman | Butte (2012) |
| Schneider et al. (2003) ^[445]^ | Yamax Skeletone EM 180 | M = 34.7 ± 12.6 yrs.  F = 43.1 ± 19.9 yrs. | 20 | M = 10  F = 10 | Yamax Skeletone determined steps – Direct Observation.  Walking over a 400 metre track (structured). | Accuracy= ±20% (95% of the time)  Bland Altman | Butte (2012) |
| Shephard et al. (1999) ^[441]^ | Stepwatch | 15-68 yrs. | 29 | M = 8  F = 21 | Stepwatch (Step activity monitor) steps – Direct Observation (steps  Over ground walking at difference speeds, ascending and descending steps (structured) | Mean absolute error (obese) = 0.5%  Mean absolute error (nonobese) = 0.6% | Berlin (2006) |
| Shephard et al. (1999) ^[441]^ | Sportline | 15-68 yrs. | 29 | M = 8  F = 21 | Sportline Pedometer determined steps– Direct Observation (steps  Over ground walking at difference speeds, ascending and descending steps (structured) | Mean absolute error (obese) = 6.1%  Mean absolute error (nonobese) = 1.6% | Berlin (2006) |
| Storti et al. (2008) ^[317]^ | Digiwalker | 79.2 ±6.0 yrs. | 34 | M = 10  F = 24 | Digiwalker determined steps – Direct Observation determined steps.  Participants wore the devices while completing a 100 step test (Structured). | Mean Absolute Error = 16.9 ± 21.6  %Error = 13% | Yang (2010) |
| Storti et al. (2008) ^[317]^ | StepWatch activity monitor | 79.2 ±6.0 yrs. | 34 | M = 10  F = 24 | StepWatch activity monitor determined steps – Direct Observation determined steps.  Participants wore the devices while completing a 100 step test (Structured). | Mean Absolute Error = 5.7 ± 5.0  %Error = 6.9% | Yang (2010) |
| Swartz et al. (2003) ^[442]^ | Digiwalker | NA | 66 | M = 35  F = 31 | Digiwalker determined steps (worn on front of thigh @ 54 m.min^-1^) – Direct observation  Digiwalker determined steps (worn on front of thigh @ 54 m.min^-1^) – Direct observation  Digiwalker determined steps (worn on front of thigh @ 54 m.min^-1^) – Direct observation  Digiwalker determined steps (worn on front of thigh @ 67 m.min^-1^) – Direct observation  Digiwalker determined steps (worn on front of thigh @ 67 m.min^-1^) – Direct observation  Digiwalker determined steps (worn on front of thigh @ 67 m.min^-1^) – Direct observation  Walking and running on a treadmill for 3 minutes at 5 different velocities. | Mean_DIFF_= -20%  Mean_DIFF_= -33%  Mean_DIFF_= -26%  Mean_DIFF_= -7%  Mean_DIFF_= -13%  Mean_DIFF_= -11% | Berlin (2006) |
| Tharion et al. (2004) ^[452]^ | Foot-ground contact pedometer | 23.0 ± 3.9 yrs. | 17 | M = 7  F = 10 | Pedometer determined energy expenditure (MJ.d-1) – Doubly labelled water determined energy expenditure (MJ.d-1).  Participants wore the pedometer for all 8 days during DLW measurement period (Unstructured). | Mean_DIFF_=0.81 MJ/d; Mean_BIAS_= -0.39 MJ; Mean_ERROR_=1.71 MJ.  Bland Altman | Plasqui (2007) |
| Tudor-Locke et al. (2006) ^[443]^ | Kelloggs Step Counter | 25-40 yrs. | 9 | F = 9 | Kelloggs Step Counter determined steps – Direct Observation  20 Step Test (structured)  Treadmill Walking and motor vehicle transport (structured) | Mean absolute percentage error = 24.2±33.9%  53% showed unacceptable accuracy | Corder (2007) |
| Tudor-Locke et al. (2006) ^[443]^ | Digiwalker | 25-40 yrs. | 9 | F = 9 | Digiwalker determined steps – Direct Observation  20 Step Test (structured)  Treadmill Walking and motor vehicle transport (structured) | Mean absolute percentage error = 3.9±6.6%  100% accuracy | Corder (2007) |
| Welk et al. (2000) ^[444]^ | Digiwalker | 29±8 yrs. | 31 | M = 17  F = 14 | Digiwalker determined steps – Direct Observation  Steps determined at different paces over a 37.4 m track. | Accurate within 3-5% | Dishman (2000) |

SW3 = Step Watch 3; NR = Not Reported; NS = Non-significant; r = Correlation Coefficient; Mean%Diff = Mean Percentage Difference; SEM = Standard Error of the Mean; LoA = Limits of Agreement; M= Male; F = Female; METs = Metabolic Equivalents; BMI = Body Mass Index; PAEE = Physical Activity Energy Expenditure.

**Supplementary Table 12.** Details of studies examining the concurrent validity of pedometers.

| **Author** | **Measure** | **Age Range** | **Sample Size** | **Sex** | **Reference** | **Results** | **Primary Source** |
| --- | --- | --- | --- | --- | --- | --- | --- |
| Abel et al. (2008) ^[270]^ | Kenz Lifecorder EX | 29.4 ± 7.1 yrs. | 20 | M = 10  F = 10 | KL determined activity energy expenditure – Indirect Calorimetry determined activity energy expenditure (@ 54 m.min^-1^).  KL determined activity energy expenditure – Indirect Calorimetry determined activity energy expenditure (@ 80 m.min^-1^).  KL determined activity energy expenditure – Indirect Calorimetry determined activity energy expenditure (@ 107 m.min^-1^).  KL determined activity energy expenditure – Indirect Calorimetry determined activity energy expenditure (@ 134 m.min^-1^).  KL determined activity energy expenditure – Indirect Calorimetry determined activity energy expenditure (@ 161 m.min^-1^).  KL determined activity energy expenditure – Indirect Calorimetry determined activity energy expenditure (@ 188 m.min^-1^).  KL determined total energy expenditure – Indirect Calorimetry determined activity energy expenditure (@ 54 m.min^-1^).  KL determined total energy expenditure – Indirect Calorimetry determined activity energy expenditure (@ 80 m.min^-1^).  KL determined total energy expenditure – Indirect Calorimetry determined activity energy expenditure (@ 107 m.min^-1^).  KL determined total energy expenditure – Indirect Calorimetry determined activity energy expenditure (@ 134 m.min^-1^).  KL determined total energy expenditure – Indirect Calorimetry determined energy expenditure (@ 161 m.min^-1^).  KL determined total energy expenditure – Indirect Calorimetry determined energy expenditure (@ 188 m.min^-1^).  Participants completed 6 treadmill trials at 54, 80, 107, 134, 161 and 188 m.min^-1^ while wearing the devices (Structured). | NS diff; r=0.74; p<0.05  NS diff; r=0.81; p<0.05  NS diff; r=0.74; p<0.05  NS diff; r=0.54; p<0.05  Underestimated; p<0.001; r=0.97; p<0.05  Underestimated; p<0.001; r=0.96; p<0.05  Overestimated; p<0.001; r=0.82; p<0.05  Overestimated; p<0.001; r=0.85; p<0.05  Overestimated; p<0.001; r=0.83; p<0.05  NS diff; r=0.61; p<0.05  Underestimated; p<0.001; r=0.97; p<0.05  Underestimated; p<0.001; r=0.96; p<0.05 | Shephard (2012) |
| Ayabe et al. (2010) ^[431]^ | Kenz Lifecorder | 69 ± 4 yrs. | 28 | M = 13  F =15 | Kenz Lifecorder determined steps/d –Digiwalker determined steps/d (Inactive Older Adults).  Kenz Lifecorder determined steps/d – Digiwalker determined steps/d (Active Older Adults).  7 days of consecutive wear of both devices concurrently over the left hip (Unstructured). | LoA = -164 – 3289 steps  LoA = -46 – 3484 steps  r=0.96; p<0.01  Bland Altman | Kowalski (2012) |
| Barreira et al. (2013) ^[454]^ | Digiwalker SW-200 | 52.6 ± 8.4 yrs. | 23 | M = 5  F = 18 | Digiwalker determined steps.d^-1^ – ActiGraph determined steps.d^-1^.  Participants wore both the ActiGraph and the Digiwalker pedometer on 7 consecutive days during free-living activities (Unstructured). | r=0.87; Mean %_DIFF_=-3.1 ± 30.7%; Absolute %_DIFF_=-23.9 ± 19.4%  Bland Altman | Pedisic (2014) |
| Bassett et al. (2000) ^[193]^ | Yamax Digi-Walker | 19-74 yrs. | 81 | M =38  F = 43 | Digiwalker determined energy expenditure – Indirect Calorimetry determined energy expenditure  Completion of selected free living tasks (Structured) | Mean_DIFF_=1.12; 95% CI = 0.96 – 1.28; p<0.001; r_p_=0.49; p<0.01  Bland Altman | Bassett et al. (2000) |
| Bassett et al. (2000) ^[193]^ | Kenz Lifecorder | 19-74 yrs. | 81 | M =38  F = 43 | Kenz Lifecorder determined energy expenditure – Indirect Calorimetry determined energy expenditure  Completion of selected free living tasks (Structured) | Mean_DIFF_=0.96; 95% CI = 0.82– 1.10; p<0.001; r_p_=0.49; p<0.01  Bland Altman | Bassett et al. (2000) |
| Bassett et al. (2000) ^[193]^ | Digiwalker | 19-74 yrs. | 81 | M =38  F = 43 | Digiwalker determined energy expenditure – ActiGraph (manufacturer equation) determined energy expenditure  Completion of selected free living tasks (Structured) | r_p_=0.80; p<0.01 | Bassett et al. (2000) |
| Bassett et al. (2000) ^[193]^ | Digiwalker | 19-74 yrs. | 81 | M =38  F = 43 | Digiwalker determined energy expenditure – ActiGraph (Freedson equation) determined energy expenditure  Completion of selected free living tasks (Structured) | r_p_=0.48; p<0.01 | Bassett et al. (2000) |
| Bassett et al. (2000) ^[193]^ | Digiwalker | 19-74 yrs. | 81 | M =38  F = 43 | Digiwalker determined energy expenditure – ActiGraph (Hendelman equation) determined energy expenditure  Completion of selected free living tasks (Structured) | r_p_=0.81; p<0.01 | Bassett et al. (2000) |
| Bassett et al. (2000) ^[193]^ | Digiwalker | 19-74 yrs. | 81 | M =38  F = 43 | Digiwalker determined energy expenditure – Caltrac determined energy expenditure  Completion of selected free living tasks (Structured) | r_p_=0.86; p<0.01 | Bassett et al. (2000) |
| Bassett et al. (2000) ^[193]^ | Digiwalker | 19-74 yrs. | 81 | M =38  F = 43 | Digiwalker determined energy expenditure – Kenz Lifecorder determined energy expenditure  Completion of selected free living tasks (Structured) | r_p_=0.93; p<0.01 | Bassett et al. (2000) |
| Bassett et al (1996) ^[450]^ | Digiwalker DW-500 | 18-65 yrs. | 10 | M= 5  F = 5 | Digiwalker determined steps – Eddie bauer determined steps  Significant differences at slower treadmill walking speeds, non-significant at higher walking speeds. | p<0.05 (slow treadmill)  p>0.05 (fast treadmill) | Bassett (2000) |
| Bassett et al (1996) ^[450]^ | Digiwalker DW-500 | 18-65 yrs. | 10 | M= 5  F = 5 | Digiwalker determined steps – Pacer determined steps  Significant differences at moderate treadmill walking speeds, non-significant at slowest and fastest walking speeds. | p<0.05 (moderate treadmill)  p>0.05 (slowest & fastest treadmill) | Bassett (2000) |
| Bassett et al (1996) ^[450]^ | Eddie Bauer Compustep II | 18-65 yrs. | 10 | M= 5  F = 5 | Eddie Bauer determined steps - Digiwalker determined steps  Significant differences at slower treadmill walking speeds, non-significant at higher walking speeds. | p<0.05 (slow treadmill)  p>0.05 (fast treadmill) | Bassett (2000) |
| Bassett et al (1996) ^[450]^ | Eddie Bauer Compustep II | 18-65 yrs. | 10 | M= 5  F = 5 | Eddie Bauer determined steps - Pacer determined steps  Significant differences at slower treadmill walking speeds, non-significant at higher walking speeds. | p<0.05 (slow treadmill)  p>0.05 (fast treadmill) | Bassett (2000) |
| Bassett et al (1996) ^[450]^ | Freestyle Pacer 798 | 18-65 yrs. | 10 | M= 5  F = 5 | Pacer determined steps - Eddie Bauer determined steps  Significant differences at slower treadmill walking speeds, non-significant at higher walking speeds. | p<0.05 (slow treadmill)  p>0.05 (fast treadmill) | Bassett (2000) |
| Bassett et al (1996) ^[450]^ | Freestyle Pacer 798 | 18-65 yrs. | 10 | M= 5  F = 5 | Pacer determined steps – Digiwalker determined steps  Significant differences at moderate treadmill walking speeds, non-significant at slowest and fastest walking speeds. | p<0.05 (moderate treadmill)  p>0.05 (slowest & fastest treadmill) | Bassett (2000) |
| Behrens et al. (2011) ^[455]^ | Accusplit Eagle 120 | Sample 1: 20.9 ± 1.6 yrs.  Sample 2: 38.7 ± 10.1 yrs. | 163 | M = 55  F = 108 | Accusplit determined steps.d^-1^ – ActiGraph determined steps.d^-1^.  Participants wore both the ActiGraph and the Accusplit pedometer on 7 consecutive days during free-living activities (Unstructured). | Sample 1: r_p_=0.85-0.87; Mean_DIFF_= -2199 ±1570.2.4; p<0.01; LoA = -1157.6 – 4757.6 steps.d^-1^.  Sample 1: r_p_=0.87; Mean_DIFF_= -1643.4 ±1581.9; p<0.01; LoA = -1001.0 – 5326.6 steps.d^-1^.  Bland Altman | Pedisic (2014) |
| Bergman et al. (2008) ^[432]^ | Digiwalker SW-200 | 76.7 ± 16.0 yrs. | 13 | M = 4  F = 9 | Digiwalker determined steps – StepWatch 3 determined steps.  Both devices worn for 24 hours free living activity behaviours (unstructured). | Mean Error = 208.43; 95% PI = 77.62 – 339.24;  Statistically significant differences observed (t_12_ = 6.62, P < .001).  Bland Altman | Kowlaski (2012) |
| Clemes et al. (2010) ^[433]^ | Silva model 56012 | 36.4±18.1 yrs. | 134 | NA | Silva determined steps – ActiGraph GT1M steps  Free-living physical activity behaviours over a 24 hour period (unstructured) | Absolute percentage error = 36.3%  Bland Altman | Bonomi (2012) |
| Clemes et al. (2010) ^[433]^ | New Lifestyles NL-1000 | 36.4±18.1 yrs. | 134 | NA | New Lifestyles determined steps – ActiGraph GT1M steps  Free-living physical activity behaviours over a 24 hour period (unstructured) | Absolute percentage error = 9%  Bland Altman | Bonomi (2012) |
| Colbert et al. (2011) ^[76]^ | New-Lifestyles NL-2000 | 74.7 ± 6.5 yrs. | 56 | M = 12  F = 44 | New Lifestyles-2000 determined step count.d^-1^ – ActiGraph GT1M determined step count.d^-1^.  New Lifestyles-2000 determined step count.d^-1^ – Sensewear Pro Armband determined step count.d^-1^  Participants wore the devices during the 15 days of DLW measurement and completed the questionnaire on day 8 and day 15 of the study (Unstructured) | r=0.88; Mean_DIFF_= 1116 ± 179; p<0.001  r=0.87; Mean_DIFF_ = 12 ± 184; NS  Bland Altman | Plasqui (2013) |
| Crouter et al. (2003) ^[448]^ | Digiwalker | 33 ± 12 yrs. | 10 | M = 5  F = 5 | Digiwalker determined distance travelled – Treadmill determined distance travelled.  Digiwalker determined net kcals.min^-1^ – Indirect Calorimetry determined kcals.min^-1^.  Participants walked on the treadmill at 54, 67, 80, 94 and 107 m.min^-1^ for 5 minutes while wearing devices and being observed (Structured). | Overestimation @ 67 m.min^-1^; p<0.05. Underestimation @ 107 m.min^-1^; p<0.05.  Overestimation @ all speeds; p<0.05. | McClain (2009) |
| Crouter et al. (2003) ^[448]^ | Kenz Lifecorder | 33 ± 12 yrs.. | 10 | M = 5  F = 5 | Kenz Lifecorder determined net kcals.min^-1^ – Indirect Calorimetry determined kcals.min^-1^.  Participants walked on the treadmill at 54, 67, 80, 94 and 107 m.min^-1^ for 5 minutes while wearing devices and being observed (Structured). | Overestimation @ 94 m.min^-1^; p<0.05. | McClain (2009) |
| Crouter et al. (2003) ^[448]^ | New Lifestyles NL-2000 | 33 ± 12 yrs. | 10 | M = 5  F = 5 | New Lifestyles -2000 determined net kcals.min^-1^ – Indirect Calorimetry determined kcals.min^-1^.  Participants walked on the treadmill at 54, 67, 80, 94 and 107 m.min^-1^ for 5 minutes while wearing devices and being observed (Structured). | Overestimation @ 54, 67, 80 and 94 m.min^-1^; p<0.05. | McClain (2009) |
| Crouter et al. (2003) ^[448]^ | Omron HJ-105 | 33 ± 12 yrs. | 10 | M = 5  F = 5 | Omron -105 determined distances travelled – Treadmill determined distance travelled.  Omron -105 determined net kcals.min^-1^ – Indirect Calorimetry determined kcals.min^-1^.  Participants walked on the treadmill at 54, 67, 80, 94 and 107 m.min^-1^ for 5 minutes while wearing devices and being observed (Structured). | Overestimation @ 54, 67 and 80 m.min^-1^; p<0.05.  Overestimation @ 54, 67, 80 and 94 m.min^-1^; p<0.05. | McClain (2009) |
| Crouter et al. (2003) ^[448]^ | Freestyle Pacer Pro | 33 ± 12 yrs. | 10 | M = 5  F = 5 | Freestyle Pacer Pro determined distance travelled – Treadmill determined distance travelled.  Freestyle Pacer Pro determined net kcals.min^-1^ – Indirect Calorimetry determined kcals.min^-1^.  Participants walked on the treadmill at 54, 67, 80, 94 and 107 m.min^-1^ for 5 minutes while wearing devices and being observed (Structured). | Underestimation @ 107 m.min^-1^; p<0.05.  Overestimation @ 67, 80, 94 and 107 m.min^-1^; p<0.05. | McClain (2009) |
| Crouter et al. (2003) ^[448]^ | Oregon Scientific PE316CA | 33 ± 12 yrs. | 10 | M = 5  F = 5 | Oregon Scientific determined distance travelled – Treadmill determined distance travelled.  Oregon Scientific determined net kcals.min^-1^ – Indirect Calorimetry determined kcals.min^-1^.  Participants walked on the treadmill at 54, 67, 80, 94 and 107 m.min^-1^ for 5 minutes while wearing devices and being observed (Structured). | Overestimation @ 54 and 67 m.min^-1^; Underestimation @ 94 and 107 m.min^-1^; p<0.05.  Overestimation @ all speeds; p<0.05. | McClain (2009) |
| Crouter et al. (2003) ^[448]^ | Sportline 345 | 33 ± 12 yrs. | 10 | M = 5  F = 5 | Sportline 345 determined distance travelled – Treadmill determined distance travelled.  Sportline 345 determined net kcals.min^-1^ – Indirect Calorimetry determined kcals.min^-1^.  Participants walked on the treadmill at 54, 67, 80, 94 and 107 m.min^-1^ for 5 minutes while wearing devices and being observed (Structured). | Underestimation @ 94 and 107 m.min^-1^; p<0.05.  Overestimation @ 67, 80, 94 and 107 m.min^-1^; p<0.05. | McClain (2009) |
| Crouter et al. (2003) ^[448]^ | Walk4Life LS 2525 | 33 ± 12 yrs. | 10 | M = 5  F = 5 | Walk4Life determined distance travelled – Treadmill determined distance travelled.  Walk4Life determined net kcals.min^-1^ – Indirect Calorimetry determined kcals.min^-1^.  Participants walked on the treadmill at 54, 67, 80, 94 and 107 m.min^-1^ for 5 minutes while wearing devices and being observed (Structured). | Overestimation @ 54 and 67 m.min^-1^; Underestimation @ 107 m.min^-1^; p<0.05.  Overestimation @ all speeds; p<0.05. | McClain (2009) |
| Harris et al. (2009) ^[421]^ | Digiwalker SW200 | M = 47.1 ± 6.5 yrs.  F = 47.1 ± 6.5 yrs. | 121 | M = 61  F = 60 | Digiwalker determined step count – ActiGraph GT1M determined step count.  ActiGraph and Digiwalker worn for 7 consecutive days (Unstructured). | r_p_=0.86; p<0.001  Mean_DIFF_=-44; 95% CI = -372 – 284  Bland Altman |  |
| Leenders et al. (2003) ^[375]^ | Digiwalker 500 | 23.7 ± 3.9 yrs. | 28 | M = 11  F = 17 | Digiwalker determined energy expenditure (from regression using steps.min^-1^) – Indirect Calorimeter determined energy expenditure.  Digiwalker determined energy expenditure (from regression using kcal.kg^-1^.min^-1^) – Indirect Calorimeter determined energy expenditure.  Participants completed 5 different locomotor speeds on a treadmill while wearing 4 devices and an indirect calorimeter (Structured) | R^2^=0.60; RMSE = 0.011  R^2^=0.67; SEE = 0.77 kcal.min^-1^; RMSE = 0.010  Bland Altman | Loprinzi (2012) |
| Le Masurier et al. (2004) ^[297]^ | Digiwalker | 20-55 yrs. | 12 | M = 6  F = 6 | Digiwalker determined steps – ActiGraph (CSA) determined steps  Treadmill walking at 5 different speeds (structured). | Absolute Percent Error = 12.8%; Diff_Lab_>0.05 | Berlin (2006) |
| Le Masurier et al. (2004) ^[297]^ | Omron HJ-105 | 20-55 yrs. | 12 | M = 6  F = 6 | Omron determined steps – ActiGraph (CSA) determined steps  Treadmill walking at 5 different speeds (structured). | Absolute Percent Error = 25.8%; Diff_Lab_>0.05 | Berlin (2006) |
| Le Masurier et al. (2004) ^[297]^ | SportLine 330 | 20-55 yrs. | 12 | M = 6  F = 6 | SportLine determined steps – ActiGraph (CSA) determined steps  Treadmill walking at 5 different speeds (structured). | Absolute Percent Error = 37%; Diff_Lab_<0.05 (all speeds) | Berlin (2006) |
| McClain et al. (2010) ^[451]^ | New Lifestyles NL-1000 | 27.3 ± 7.1 yrs. | 26 | M = 9  F = 17 | New Lifestyles determined mins spent in MVPA – ActiGraph determined mins spent in MVPA (Freedson 3 MET equation)  New Lifestyles determined mins spent in MVPA – ActiGraph determined mins spent in MVPA (Freedson 3.5 MET equation)  New Lifestyles determined mins spent in MVPA – ActiGraph determined mins spent in MVPA (NHANES equation)  New Lifestyles determined mins spent in MVPA – ActiGraph determined mins spent in MVPA (Swartz equation)  New Lifestyles determined mins spent in MVPA – ActiGraph determined mins spent in MVPA (Matthews equation)  Devices worn during 10 treadmill based walking and running speeds and for the remainder of the day (Structured + Unstructured). | r=0.88  r=0.82  r=0.87  r=0.47  r=0.57 | Tudor-Locke (2012) |
| McClain et al. (2010) ^[451]^ | Walk4Life Pro | 27.3 ± 7.1 yrs. | 26 | M = 9  F = 17 | Walk4Life determined activity time – ActiGraph determined mins spent in MVPA (Freedson 3 MET equation)  Walk4Life determined activity time – ActiGraph determined mins spent in MVPA (Freedson 3.5 MET equation)  Walk4Life determined activity time – ActiGraph determined mins spent in MVPA (NHANES equation)  Walk4Life determined activity time – ActiGraph determined mins spent in MVPA (Swartz equation)  Walk4Life determined activity time – ActiGraph determined mins spent in MVPA (Matthews equation)  Devices worn during 10 treadmill based walking and running speeds and for the remainder of the day (Structured + Unstructured). | r=0.32  r=0.18  r=0.21  r=0.57  r=0.54 | Tudor-Locke (2012) |
| McClain et al. (2010) ^[451]^ | Omron HJ-151 | 27.3 ± 7.1 yrs. | 26 | M = 9  F = 17 | Omron determined mins spent in MVPA – ActiGraph determined mins spent in MVPA (Freedson 3 MET equation)  Omron determined mins spent in MVPA – ActiGraph determined mins spent in MVPA (Freedson 3.5 MET equation)  Omron determined mins spent in MVPA – ActiGraph determined mins spent in MVPA (NHANES equation)  Omron determined mins spent in MVPA – ActiGraph determined mins spent in MVPA (Swartz equation)  Omron determined mins spent in MVPA – ActiGraph determined mins spent in MVPA (Matthews equation)  Devices worn during 10 treadmill based walking and running speeds and for the remainder of the day (Structured + Unstructured). | r=0.88  r=0.87  r=0.88  r=0.33  r=0.43 | Tudor-Locke (2012) |
| McClain et al. (2007) ^[456]^ | Kenz Lifecorder EX | 29.0 ± 2.3 yrs. | 10 | M = 5  F = 5 | Kenz Lifecorder determined steps – ActiGraph determined steps  Participants wore 2 KL and 1 AG for a 24 hour measurement period (a day when they would run for at least 20 minutes) (Unstructured). | Mean_DIFF_=1516 steps; p<0.05  Bland Altman | Tudor-Locke (2012) |
| Park et al. (2011) ^[440]^ | Kenz Lifecorder | 23-41 yrs. | 18 | M = 9  F = 9 | Kenz Lifecorder determined energy expenditure (METs.min^-1^) – Indirect Calorimetry determined energy expenditure (METs.min^-1^) (at 55 m.min^-1^).  Kenz Lifecorder determined energy expenditure (METs.min^-1^) – Indirect Calorimetry determined energy expenditure (METs.min^-1^) (at 75 m.min^-1^).  Kenz Lifecorder determined energy expenditure (METs.min^-1^) – Indirect Calorimetry determined energy expenditure (METs.min^-1^) (at 95 m.min^-1^).  Participants wore thee devices and an indirect calorimeter while walking at 3 speeds on a treadmill (Structured). | Mean_DIFF_=-0.3 METs; p<0.01;  Mean_DIFF_=-0.5 METs; p<0.01;  Mean_DIFF_=-0.3 METs; p<0.05; | Shephard (2012) |
| Pomeroy et al. (2011) ^[390]^ | Accuspilt AX120 | 20-34 yrs. | 50 | M = 25  F = 25 | Accusplit determined steps – MTI determined steps.  Accusplit determined steps – Dynastream determined steps.  Participants wore the devices while walking on a 540m laboratory walk test (Structured). | Absolute_DIFF_ Men = 6.0; IQR = 2.0-17.8  Signed_DIFF_ Men = -2.0; IQR = -10.5 – 1.1  Absolute_DIFF_ Women = 10.4; IQR = 7.1 – 16.1  Signed_DIFF_ Women = -7.7; IQR = -16.6 – -1.2  Absolute_DIFF_ Men = 4.4; IQR = 1.6 -11.4  Signed_DIFF_ Men = -1.8; IQR = -6.4 – 1.3  Absolute_DIFF_ Women = 3.7; IQR = 0.8 – 9.5  Signed_DIFF_ Women = -0.6; IQR = -8.4 – 0.9  Bland Altman | Plasqui (2013) |
| Pomeroy et al. (2011) ^[390]^ | Accuspilt AX120 | 20-34 yrs. | 50 | M = 25  F = 25 | Accusplit determined steps – MTI determined steps.  Accusplit determined steps – Dynastream determined steps.  Participants wore the devices during free-living settings for a 7 day period (Unstructured). | Absolute_DIFF_ Men = 20.2; IQR = 7.6-32.1  Signed_DIFF_ Men = -11.4; IQR = -30.4 – 2.2  Absolute_DIFF_ Women = 15.2; IQR = 8.6 – 26.3  Signed_DIFF_ Women = 7.9; IQR = -9.1 – 15.7  Absolute_DIFF_ Men = 26.6; IQR = 12.0 -41.5  Signed_DIFF_ Men = -26.6; IQR = -41.5 – 12.0  Absolute_DIFF_ Women = 22.5; IQR = 11.3 – 28.5  Signed_DIFF_ Women = -22.0; IQR = -28.5 – -9.6  Bland Altman | Plasqui (2013) |
| Schneider et al. (2004) ^[457]^ | Accusplit Alliance 1510 | M = 39.5 ± 16.6 yrs.  F = 43.3 ± 16.6 yrs. | 20 | M = 10  F = 10 | Accusplit Alliance 1510 determined steps – Yamax Digi-Walker determined steps.  Yamax worn on left side of body and comparison worn on other side for 24 hours. | Mean_DIFF_= -2445; 95%CI = -3454 - -1436; Yamax; p<0.05  Bland Altman | Berlin (2006) |
| Schneider et al. (2004) ^[457]^ | Freestyle Pacer Pro | M = 39.5 ± 16.6 yrs.  F = 43.3 ± 16.6 yrs. | 20 | M = 10  F = 10 | Freestyle Pacer Pro determined steps – Digiwalker determined steps.  Digiwalker worn on left side of body and comparison worn on other side for 24 hours. | Mean_DIFF_= -2189; 95%CI = -3451 - -926; Yamax; p<0.05  Bland Altman plots | Berlin (2006) |
| Schneider et al. (2004) ^[457]^ | Yamax Skeletone EM180 | M = 39.5 ± 16.6 yrs.  F = 43.3 ± 16.6 yrs. | 20 | M = 10  F = 10 | Yamax Skeletone EM180determined steps – Digiwalker determined steps.  Yamax worn on left side of body and comparison worn on other side for 24 hours. | Mean_DIFF_= -1161; 95%CI = -2228 - -94; Yamax; p<0.05  Bland Altman | Berlin (2006) |
| Schneider et al. (2004) ^[457]^ | Colorado on the Move | M = 39.5 ± 16.6 yrs.  F = 43.3 ± 16.6 yrs. | 20 | M = 10  F = 10 | Colorado on the Move determined steps – Digiwalker determined steps.  Yamax worn on left side of body and comparison worn on other side for 24 hours. | Mean_DIFF_= -1042; 95%CI = -2407 - -38; Yamax; p<0.05  Bland Altman | Berlin (2006) |
| Schneider et al. (2004) ^[457]^ | Sportline 345 | M = 39.5 ± 16.6 yrs.  F = 43.3 ± 16.6 yrs. | 20 | M = 10  F = 10 | Sportline 345 determined steps – Digiwalker determined steps.  Yamax worn on left side of body and comparison worn on other side for 24 hours. | Mean_DIFF_= -997; 95%CI = -1873 - -121; Yamax; p<0.05  Bland Altman | Berlin (2006) |
| Schneider et al. (2004) ^[457]^ | Walk4Life LS 2525 | M = 39.5 ± 16.6 yrs.  F = 43.3 ± 16.6 yrs. | 20 | M = 10  F = 10 | Walk4Life LS 2525 determined steps – Digiwalker determined steps.  Yamax worn on left side of body and comparison worn on other side for 24 hours. | Mean_DIFF_= 1099; 95%CI = 241 - 1957; Yamax; p<0.05  Bland Altman | Berlin (2006) |
| Schneider et al. (2004) ^[457]^ | Omron HJ 105 | M = 39.5 ± 16.6 yrs.  F = 43.3 ± 16.6 yrs. | 20 | M = 10  F = 10 | Omron HJ 105 determined steps – Digiwalker determined steps.  Yamax worn on left side of body and comparison worn on other side for 24 hours. | Mean_DIFF_= 2266; 95%CI = 853 - 3679; Yamax; p<0.05  Bland Altman | Berlin (2006) |
| Schneider et al. (2004) ^[457]^ | Oregon Scientific PE316CA | M = 39.5 ± 16.6 yrs.  F = 43.3 ± 16.6 yrs. | 20 | M = 10  F = 10 | Oregon Scientific PE316CA determined steps – Digiwalker determined steps.  Yamax worn on left side of body and comparison worn on other side for 24 hours. | Mean_DIFF_= 3636; 95%CI = 2390 - 4882; Yamax; p<0.05  Bland Altman | Berlin (2006) |
| Schneider et al. (2004) ^[457]^ | Kenz Lifecorder | M = 39.5 ± 16.6 yrs.  F = 43.3 ± 16.6 yrs. | 20 | M = 10  F = 10 | Kenz Lifecorder determined steps – Digiwalker determined steps.  Yamax worn on left side of body and comparison worn on other side for 24 hours. | Mean_DIFF_= -703; 95%CI = -1422 - 17; Yamax; p>0.05  Bland Altman | Berlin (2006) |
| Schneider et al. (2004) ^[457]^ | Yamax Digi-Walker SW-200 | M = 39.5 ± 16.6 yrs.  F = 43.3 ± 16.6 yrs. | 20 | M = 10  F = 10 | Digiwalker SW-200 determined steps – Digiwalker determined steps.  Yamax worn on left side of body and comparison worn on other side for 24 hours. | Mean_DIFF_= -372; 95%CI = -1161 - 417; Yamax; p>0.05  Bland Altman | Berlin (2006) |
| Schneider et al. (2004) ^[457]^ | New Lifestyles NL 2000 | M = 39.5 ± 16.6 yrs.  F = 43.3 ± 16.6 yrs. | 20 | M = 10  F = 10 | New Lifestyles NL 2000 determined steps – Digiwalker determined steps.  Yamax worn on left side of body and comparison worn on other side for 24 hours. | Mean_DIFF_= 206; 95%CI = -514 - 926; Yamax; p>0.05  Bland Altman | Berlin (2006) |
| Schneider et al. (2004) ^[457]^ | Yamax Digi-Walker SW-701 | M = 39.5 ± 16.6 yrs.  F = 43.3 ± 16.6 yrs. | 20 | M = 10  F = 10 | Yamax Digi-Walker SW-200 determined steps – Digiwalker determined steps.  Yamax worn on left side of body and comparison worn on other side for 24 hours. | Mean_DIFF_= 426; 95%CI = -298 - 1149; Yamax; p>0.05  Bland Altman | Berlin (2006) |
| Schneider et al. (2004) ^[457]^ | Sportline 330 | M = 39.5 ± 16.6 yrs.  F = 43.3 ± 16.6 yrs. | 20 | M = 10  F = 10 | Sportline 330 determined steps – Digiwalker determined steps.  Yamax worn on left side of body and comparison worn on other side for 24 hours. | Mean_DIFF_= 443; 95%CI = -439 - 1325; Yamax; p>0.05  Bland Altman | Berlin (2006) |
| Stone et al. (2007) ^[398]^ | AMP 331 | 17.6 ± 8 yrs. | 86 | M = NA  F = NA | AMP determined energy expenditure (kcal.min^-1^) – Indirect Calorimetry determined energy expenditure (kcal.min^-1^).  AMP determined energy expenditure (kcal.min^-1^) using research development regression equation – Indirect Calorimetry determined energy expenditure (kcal.min^-1^).  Participant wore device and had indirect Calorimetry measured while completing 3 treadmill walking/running speeds (Structured) | R^2^=0.33-0.67;  R^2^=0.87-0.94; %increase in accuracy=20-61% | Van Remoortel (2012) |
| Stone et al. (2007) ^[398]^ | Digiwalker | 17.6 ± 8 yrs. | 86 | M = NA  F = NA | Digiwalker determined energy expenditure (kcal.min^-1^) – Indirect Calorimetry determined energy expenditure (kcal.min^-1^).  Digiwalker determined energy expenditure (kcal.min^-1^) using research development regression equation – Indirect Calorimetry determined energy expenditure (kcal.min^-1^).  Participant wore device and had indirect Calorimetry measured while completing 3 treadmill walking/running speeds (Structured) | R^2^=0.92-96;  R^2^=0.85-94; %decrease in accuracy= -2 - -7% | Van Remoortel (2012) |
| Thompson et al. (2006) ^[403]^ | Digiwalker DW 351 | 25 ± 5 yrs. | 20 | M = 10  F = 10 | Digiwalker determined energy expenditure (kJ.min^-1^determined from stride length and prediction equations) – Indirect Calorimetry determined energy expenditure (kJ.min^-1^).  Participants wore the pedometer and indirect calorimeter during 6 activities in the laboratory, with data extrapolated for an average 16 hour day (Structured). | Mean_DIFF_= -1817 kJ; % _DIFF_=-20% | Warren (2010) |
| Tudor-Locke et al. (2002) ^[458]^ | Digiwalker SW-200 | 38.2 ± 12.0 yrs. | 52 | M = 27  F = 25 | Digiwalker determined steps.d^-1^ – CSA determined total steps.d^-1^.  Both Digiwalker and CSA devices worn for 7 consecutive days (Unstructured). | r=0.86; p<0.0001; Mean_DIFF_=1845 ± 2116 steps.d^-1^. 95%LoA = -2387 – 6077  Bland Altman | Reiser & Schlenk (2009) |
| Tudor-Locke et al. (2006) ^[443]^ | Digiwalker | 25-40 yrs. | 9 | F = 9 | Digiwalker determined steps – ActiGraph CSA determined step counts  Free-living activity behaviours for 24 hours | Mean absolute percentage error = 19.5±21.2% | Corder (2007) |
| Tudor-Locke et al. (2006) ^[443]^ | Kelloggs Step Counter | 25-40 yrs. | 9 | F = 9 | Kelloggs step counter determined steps – ActiGraph CSA determined step counts  Free-living activity behaviours for 24 hours (Unstructured). | Mean absolute percentage error = 44.9±34.5% | Corder (2007) |

DLW = Doubly Labelled Water; CSA= Computer Science Applications accelerometer; AG = ActiGraph; MVPA = Moderate to Vigorous Intensity Physical Activity; METs = Metabolic Equivalents; NHANES = National Health and Nutrition Examination Survey; M= Male; F = Female; NS = Non-significant; r = Correlation Coefficient; rp = Pearson’s Correlation; rs = Pearson’s Correlation; LoA = Limits of Agreement; CI = Confidence Intervals; MeanDIFF = Mean Difference; SEE = Standard Error of the Estimate; RMSE = Root Mean Squared Error; IQR = Interquartile Range.

**Supplementary Table 13:** Details of studies examining inter-instrument reliability in pedometer devices.

| **Author** | **Measure** | **Age Range** | **Sample Size** | **Sex** | **Details** | **Results** | **Primary Source** |
| --- | --- | --- | --- | --- | --- | --- | --- |
| Bassett et al (1996) ^[450]^ | Digiwalker DW-500 | 18-65 yrs. | 20 | M= 13  F = 7 | Left and right hip  Differences in distances walked when compared over 4.88km | No-significant differences; p>0.05 | Bassett (2000) |
| Bassett et al (1996) ^[450]^ | Freestyle Pacer 798 | 18-65 yrs. | 20 | M= 13  F = 7 | Left and right hip  Differences in distances walked when compared over 4.88km | No-significant differences; p>0.05 | Bassett (2000) |
| Bassett et al (1996) ^[450]^ | Accusplit Fitness Walker | 18-65 yrs. | 20 | M= 13  F = 7 | Left and right hip  Differences in distances walked when compared over 4.88km | No-significant differences; p>0.05 | Bassett (2000) |
| Bassett et al (1996) ^[450]^ | Eddie Bauer Compustep II | 18-65 yrs. | 20 | M= 13  F = 7 | Left and right hip  Differences in distances walked when compared over 4.88km | p<0.01 | Bassett (2000) |
| Bassett et al (1996) ^[450]^ | L.L. Bean Pedometer | 18-65 yrs. | 20 | M= 13  F = 7 | Left and right hip  Differences in distances walked when compared over 4.88km | No-significant differences; p>0.05 | Bassett (2000) |
| Crouter et al. (2003) ^[448]^ | Digiwalker | 33 ± 12 yrs.. | 10 | M = 5  F = 5 | Left and right side of the body, over the middle of the thigh  Participants walked on the treadmill at 5 different speeds for 5 minutes while wearing devices and being observed (Structured). | ICC= 0.98; 95% CI = 0.94-0.98 | McClain (2009) |
| Crouter et al. (2003) ^[448]^ | Kenz Lifecorder | 33 ± 12 yrs.. | 10 | M = 5  F = 5 | Left and right side of the body, over the middle of the thigh  Participants walked on the treadmill at 5 different speeds for 5 minutes while wearing devices and being observed (Structured). | ICC= 0.94; 95% CI = 0.90-0.97 | McClain (2009) |
| Crouter et al. (2003) ^[448]^ | New Lifestyles NL-2000 | 33 ± 12 yrs.. | 10 | M = 5  F = 5 | Left and right side of the body, over the middle of the thigh  Participants walked on the treadmill at 5 different speeds for 5 minutes while wearing devices and being observed (Structured). | ICC= 0.99; 95% CI = 0.98-0.99 | McClain (2009) |
| Crouter et al. (2003) ^[448]^ | Sportline 330 | 33 ± 12 yrs.. | 10 | M = 5  F = 5 | Left and right side of the body, over the middle of the thigh  Participants walked on the treadmill at 5 different speeds for 5 minutes while wearing devices and being observed (Structured). | ICC= 0.91; 95% CI = 0.85-0.95 | McClain (2009) |
| Crouter et al. (2003) ^[448]^ | Omron HJ-105 | 33 ± 12 yrs.. | 10 | M = 5  F = 5 | Left and right side of the body, over the middle of the thigh  Participants walked on the treadmill at 5 different speeds for 5 minutes while wearing devices and being observed (Structured). | ICC= 0.83; 95% CI = 0.71-0.90 | McClain (2009) |
| Crouter et al. (2003) ^[448]^ | Freestyle Pacer Pro | 33 ± 12 yrs.. | 10 | M = 5  F = 5 | Left and right side of the body, over the middle of the thigh  Participants walked on the treadmill at 5 different speeds for 5 minutes while wearing devices and being observed (Structured). | ICC= 0.95; 95% CI = 0.92-0.97 | McClain (2009) |
| Crouter et al. (2003) ^[448]^ | Oregon Scientific PE316CA | 33 ± 12 yrs.. | 10 | M = 5  F = 5 | Left and right side of the body, over the middle of the thigh  Participants walked on the treadmill at 5 different speeds for 5 minutes while wearing devices and being observed (Structured). | ICC= 0.76; 95% CI = 0.61-0.86 | McClain (2009) |
| Crouter et al. (2003) ^[448]^ | Sportline 345 | 33 ± 12 yrs.. | 10 | M = 5  F = 5 | Left and right side of the body, over the middle of the thigh  Participants walked on the treadmill at 5 different speeds for 5 minutes while wearing devices and being observed (Structured). | ICC= 0.57; 95% CI = 0.35-0.73 | McClain (2009) |
| Crouter et al. (2003) ^[448]^ | Walk4Life LS 2525 | 33 ± 12 yrs.. | 10 | M = 5  F = 5 | Left and right side of the body, over the middle of the thigh  Participants walked on the treadmill at 5 different speeds for 5 minutes while wearing devices and being observed (Structured). | ICC= 0.81; 95% CI = 0.68-0.89 | McClain (2009) |
| Crouter et al. (2003) ^[448]^ | Skeletone EM 180 | 33 ± 12 yrs.. | 10 | M = 5  F = 5 | Left and right side of the body, over the middle of the thigh  Participants walked on the treadmill at 5 different speeds for 5 minutes while wearing devices and being observed (Structured). | ICC= 0.83; 95% CI = 0.89-0.96 | McClain (2009) |
| Holbrook et al. (2009) ^[446]^ | Omron HJ-151 | M: 26 ± 6.5 yrs.  F: 22.1 ± 2.1 yrs. | 47 | M = 24  F = 23 | OM HJ151 worn at mid back, right and left hip.  Slow walking Speed  Moderate walking speed  Fast walking speed  Self-paced walking speed | CV=1.3%  CV=1.2%  CV=1.1%  CV=1.3% | Tudor-Locke (2012) |
| Holbrook et al. (2009) ^[446]^ | Omron HJ-720ITC | M: 26 ± 6.5 yrs.  F: 22.1 ± 2.1 yrs. | 47 | M = 24  F = 23 | OM HJ151 worn at mid back, right and left hip.  Slow walking Speed  Moderate walking speed  Fast walking speed  Self-paced walking speed | CV=3.3%  CV =2.8%  CV =2.8%  CV =1.4% | Tudor-Locke (2012) |
| McClain et al. (2007) ^[456]^ | Kenz Lifecorder EX | 29.0 ± 2.3 yrs. | 10 | M = 5  F = 5 | Kenz Lifecorder EX worn on both the left and right hip.  Steps  Time spent in moderate PA  Time spent in vigorous PA  Time spent in moderate-to-vigorous PA | ICC=0.99  ICC=0.95  ICC=0.99  ICC=0.98 | Tudor-Locke (2012) |
| Ryan et al. (2006) ^[314]^ | Digiwalker SW-200 | 34.5 ± 6.9 yrs. | 20 | M = 8  F = 12 | Reliability of the Yamax for step count at the various walking speeds (devices worn on left and right hip).  Treadmill Speed 0.9 m.s^-1^  Treadmill Speed 1.12 m.s^-1^  Treadmill Speed 1.33 m.s^-1^  Treadmill Speed 1.56 m.s^-1^  Treadmill Speed 1.78 m.s^-1^  Outdoor Speed Slow  Outdoor Speed Normal  Outdoor Speed Fast | ICC=0.30  ICC=0.52  ICC=0.45  ICC=0.37  ICC=0.89  ICC=0.48  ICC=0.56  ICC=0.74 | Tudor-Locke (2012) |
| Ryan et al. (2006) ^[314]^ | Omron HJ-109-E | 34.5 ± 6.9 yrs. | 20 | M = 8  F = 12 | Reliability of the Omron for step number at the various walking speeds (2 devices worn on left and right hip).  Treadmill Speed 0.9 m.s^-1^  Treadmill Speed 1.12 m.s^-1^  Treadmill Speed 1.33 m.s^-1^  Treadmill Speed 1.56 m.s^-1^  Treadmill Speed 1.78 m.s^-1^  Outdoor Speed Slow  Outdoor Speed Normal  Outdoor Speed Fast | ICC=0.48  ICC=0.22  ICC=0.12  ICC=0.56  ICC=0.89  ICC=0.01  ICC=0.73  ICC=0.70 | Tudor-Locke (2012) |
| Schneider et al. (2003) ^[445]^ | Digiwalker | M = 34.7 ± 12.6 yrs.  F = 43.1 ± 19.9 yrs. | 20 | M = 10  F = 10 | 4 models of each device worn over 400 metre walking track.  Cronbach Alpha presented | α=0.992 | Butte (2012) |
| Schneider et al. (2003) ^[445]^ | Kenz Lifecorder | M = 34.7 ± 12.6 yrs.  F = 43.1 ± 19.9 yrs. | 20 | M = 10  F = 10 | 4 models of each device worn over 400 metre walking track.  Cronbach Alpha presented | α=0.998 | Butte (2012) |
| Schneider et al. (2003) ^[445]^ | New Lifestyles NL-2000 | M = 34.7 ± 12.6 yrs.  F = 43.1 ± 19.9 yrs. | 20 | M = 10  F = 10 | 4 models of each device worn over 400 metre walking track.  Cronbach Alpha presented | α=0.995 | Butte (2012) |
| Schneider et al. (2003) ^[445]^ | Sportline 330 | M = 34.7 ± 12.6 yrs.  F = 43.1 ± 19.9 yrs. | 20 | M = 10  F = 10 | 4 models of each device worn over 400 metre walking track.  Cronbach Alpha presented | α=0.76 | Butte (2012) |
| Schneider et al. (2003) ^[445]^ | Omron HJ-105 | M = 34.7 ± 12.6 yrs.  F = 43.1 ± 19.9 yrs. | 20 | M = 10  F = 10 | 4 models of each device worn over 400 metre walking track.  Cronbach Alpha presented | α=0.991 | Butte (2012) |
| Schneider et al. (2003) ^[445]^ | Freestyle Pacer Pro | M = 34.7 ± 12.6 yrs.  F = 43.1 ± 19.9 yrs. | 20 | M = 10  F = 10 | 4 models of each device worn over 400 metre walking track.  Cronbach Alpha presented | α=0.90 | Butte (2012) |
| Schneider et al. (2003) ^[445]^ | Oregon Scientific PE316CA | M = 34.7 ± 12.6 yrs.  F = 43.1 ± 19.9 yrs. | 20 | M = 10  F = 10 | 4 models of each device worn over 400 metre walking track.  Cronbach Alpha presented | α=0.924 | Butte (2012) |
| Schneider et al. (2003) ^[445]^ | Sportline 345 | M = 34.7 ± 12.6 yrs.  F = 43.1 ± 19.9 yrs. | 20 | M = 10  F = 10 | 4 models of each device worn over 400 metre walking track.  Cronbach Alpha presented | α=0.87 | Butte (2012) |
| Schneider et al. (2003) ^[445]^ | Walk4Life LS 2525 | M = 34.7 ± 12.6 yrs.  F = 43.1 ± 19.9 yrs. | 20 | M = 10  F = 10 | 4 models of each device worn over 400 metre walking track.  Cronbach Alpha presented | α=0.885 | Butte (2012) |
| Schneider et al. (2003) ^[445]^ | Yamax Skeletone EM 180 | M = 34.7 ± 12.6 yrs.  F = 43.1 ± 19.9 yrs. | 20 | M = 10  F = 10 | 4 models of each device worn over 400 metre walking track.  Cronbach Alpha presented | α=0.935 | Butte (2012) |

M= Male; F = Female; ICC = Intraclass Correlation Coefficient; CI = Confidence Intervals; CV = Coefficient of Variation; α = Cronbach Alpha.

**Supplementary Table 14.** Details of studies examining the test-retest reliability of pedometers.

| **Author** | **Measure** | **Age Range** | **Sample Size** | **Sex** | **Details** | **Results** | **Primary Source** |
| --- | --- | --- | --- | --- | --- | --- | --- |
| Felton et al. (2006) ^[459]^ | Digiwalker SW-200 | 18.7 ±1.2 yrs. | 69 | F=69 | Participants wore the pedometer for 7 days during 1 week, with ICC for steps.d^-1^ across days identified. | ICC_3d_ =0.94-0.97 | Tudor Locke (2009) |
| Hart et al. (2011) ^[422]^ | Digiwalker SW-200 | 69.3 ± 7.4 yrs. | 52 | M = 13  F = 39 | Pedometer worn on 21 consecutive days during free-living physical activity behaviours, with ICC for total PA across days identified. | ICC_4d_=0.80; ICC_6d_=0.85; ICC_10d_=0.90;  ICC_21d_=0.95 | Pedisic (2014) |
| Kang et al. (2009) ^[461]^ | Digiwalker SW200 | 38±9.9 yrs. | 16 | F = 16 | 365 days of free living activity recorded and ICCs identified for number of days of monitoring (steps/day). | ICC_2d_= 0.71 ICC_3d_= 0.73 ICC_4d_= 0.78  ICC_5d_= 0.83 ICC_6d_= 0.84  ICC_7d_= 0.88 ICC_14d_= 0.92  ICC_21d_= 0.95  ICC_30d_= 0.96 | Kim (2013) |
| Rowe et al. (2007) ^[426]^ | Digiwalker | 74.0 ±9.5 yrs. | 81 | NA | 7 day free living stepping recorded and identifying ICC for steps.d^-1^ across each number of days. | ICC_2d_=0.90  ICC_3d-6d_=0.92-0.94  ICC_7d_=0.96 | Gorman (2014) |
| Rowlands et al. (2007) ^[313]^ | Yamax Digiwalker | 23.1 ± 3.4 yrs. | 10 | M = 10 | Walking and jogging at 11 speeds on a treadmill. Speeds ranged from 4-26 kmph. Duration between tests was 1 month.  . | ICC=-0.19 – 0.94; 7/11 speeds ICC>0.80; p<0.05 | Corder (2007) |
| Strycker et al. (2007) ^[460]^ | Digiwalker | 40-70 yrs. | 270 | F = 270 | Free living stepping recorded, and first 2 complete days’ data examined. Additional days added until reaching 5 days’ of data. | α_2d_=0.84  α_5d_=0.87 | De Vries (2009) |
| Tudor-Locke et al. (2005) ^[462]^ | Digiwalker | M = 49.1±16.2 yrs.  F = 44.8±16.9 yrs. | 90 | M = 33  F = 57 | 7 day free living stepping recorded, and identifying ICC for each number of days. | ICC_1d_=0.72  ICC_2d_=0.71-0.84  ICC_>2d_=0.90 | Berlin (2006) |

M= Male; F = Female; ICC = Intraclass Correlation Coefficient.

**Supplementary Table 15.** Details of studies examining the sensitivity to change of pedometers.

| **Author** | **Measure** | **Age Range** | **Sample Size** | **Sex** | **Details** | **Results** | **Primary Source** |
| --- | --- | --- | --- | --- | --- | --- | --- |
| Tudor-Locke et al. (2001) ^[463]^ | Digiwalker SW-200 | 53 ± 6 yrs. | 9 | M = 3  F = 6 | Digiwalker determined total steps at pre-intervention – Digiwalker determined total steps at post-intervention.  Activity Monitor worn for 3 consecutive days both pre and post-intervention. Intervention was 4 weeks in duration. | Mean_DIFF_=3700 steps; p<0.001; Effect size = 1.68 | Shephard (2012) |

M= Male; F = Female; Mean_DIFF_ = Mean Difference.

**Supplementary Table 16.** Details of studies examining the criterion validity of heart rate monitoring devices.

| **Author** | **Measure** | **Age Range** | **Sample Size** | **Sex** | **Reference** | **Results** | **Primary Source** |
| --- | --- | --- | --- | --- | --- | --- | --- |
| Davidson et al. (1997) ^[122]^ | Polar Sports Tester 4000 | 25-54 yrs. | 9 | M = 9 | Heart rate monitoring (FLEX HR; Individual Calibration) determined energy expenditure – Doubly labelled water energy expenditure  9 days of free-living physical activity behaviours using FLEX HR calibration curves (Unstructured) | Mean_DIFF_ = +16.3%; p<0.05  Bland Altman | Ainslie (2003) |
| Fuller et al. (2008) ^[79]^ | Polar Sports Tester | 20-66 yrs. | 60 | M = 30  F = 30 | Heart rate monitor determined energy expenditure (MJ.d^-1^) (FLEX HR; Individual Calibration) – Doubly Labelled Water determined energy expenditure (MJ.d^-1^)  Heart rate monitor determined energy expenditure (PAL) (FLEX HR; Individual Calibration) – Doubly Labelled Water determined energy expenditure (PAL)  15 days of DLW measurement and 12 days of HRM, (Unstructured). | r=0.64; p<0.01  Error s.d. = 1.6 MJ (CV=14.9)  r=0.36; p<0.01  Error s.d. = 0.24 MJ (CV=14.3)  Bland Altman | Plasqui (2013) |
| Johansson et al. (2006)^[370]^ | Heart Rate monitor | M: 32.0 ± 12.8 yrs.  F: 26.6 ± 5.3 yrs.  M: 28-63 yrs.  F: 28-30 yrs. | 27  8 | M = 14  F = 13  M=6  F=2 | Flex-HR determined total energy expenditure (MJ.d^-1^) – Doubly Labelled Water determined total energy expenditure (MJ.d^-1^).  Flex-HR determined physical activity energy expenditure (kJ.kg.d^-1^) – Doubly Labelled Water determined total energy expenditure (kJ.kg.d^-1^).  Participants wore the HRM and the ActiGraph MTI during model development with indirect Calorimetry while completing walking and running activities on the treadmill (Structured), while 8 participants wore HRM and ACC during free-living activities for 14 days with DLW measurement (Unstructured). | RMSE=3.99; Sig Diff from 0.  Mean_%DIFF_=-24.6  RMSE=47.3; Sig Diff from 0.  Mean_%DIFF_=-60.8  Bland Altman | Van Remoortel (2012) |
| Kashiwazaki (1999) ^[464]^ | Memory Mac Heart Rate Monitor | 18-65 yrs. | 10 | M = 5  F = 5 | TEE determined by Heart Rate (straight linear regression; FLEX HR; Individual Calibration) – TEE determined by doubly labelled water.  TEE determined by Heart Rate (natural logarithm linear regression; FLEX HR; Individual Calibration) – TEE determined by doubly labelled water.  TEE determined by Heart Rate (two linear regression (FLEX HR; Individual Calibration)) – TEE determined by doubly labelled water.  Heart rate recorded for a 24 hour period compared to TEE recorded from DLW for 14 days (Unstructured). | r=0.527; Mean Percentage Diff =+19.7 ± 38.1%  r=0.577; p<0.05;  Mean Percentage Diff = -3.1 ± 24.8%  r=0.563; p<0.05  Mean Percentage Diff = 7.5 ± 36.7%  Bland Altman | Levine (2005) |
| Livingston et al. (1990) ^[465]^ | Heart Rate Monitoring (Sport Tester PE 3000) | M= 29.4 ± 6.4 yrs.  F= 34.2 ± 6.9 yrs. | 14 | M = 9  F = 5 | Heart Rate monitor determined total energy expenditure (FLEX HR; Individual Calibration) – Doubly labelled water determined total energy expenditure.  Heart Rate monitor recorded for a maximum of 16 hours over 2-4 days compared with 14 days of DLW measurement (Unstructured). | Percent_DIFF_= +2.0 (17.9); Non-significant; 95% CI = -5.0 – 5.19 MJ/d.  Bland Altman | LaMonte et al. (2001) |
| Lof et al. (2002) ^[95]^ | Polar Heart Rate Monitor | 30 ± 4 yrs. | 24 | F = 24 | Heart Rate monitor (FLEX HR; Individual Calibration) determined energy expenditure (kcal.d^-1^) - Doubly labelled water determined energy expenditure (kcal.d^-1^).  Heart Rate monitor device worn during the 14 days of DLW assessment (Unstructured). | Mean_DIFF_= 58 kcal; 2SDs = 677 kcal.24hrs^-1^. Systematic bias: r=0.51; p<0.05  Bland Altman | Plasqui (2007) |
| Lof et al. (2003) ^[303]^ | Polar Vantage NV | 29 ± 4 yrs. | 37 | F = 37 | Physical activity level as determined by heart rate monitor (Using MET factor; Linear relationship; Individual calibration) – Doubly labelled water determined physical activity level.  Total energy expenditure as determined by HRM (KJ.d^-1^) – Doubly labelled water determined total energy expenditure.  4-7 days of heart rate monitoring from HRM compared to 14 days of DLW measurement (Unstructured). | Mean_DIFF_=-0.22; LoA =0.50; p<0.05  Mean_DIFF_=-83; NS; LoA = 2706 KJ.d^-1^.  Bland Altman | Nielson (2009) |
| Morio et al (1999) ^[84]^ | Heart Rate Monitoring (Life Scope 6) | 70.1 ± 2.7 yrs. | 12 | M = 6  F = 6 | Heart rate monitor determined energy expenditure (Individual Calibration based on prolonged stay in calorimetry chamber) – Doubly Labelled Water determined energy expenditure.  Heart rate monitor energy expenditure for 4 days based on prolonged stay in Calorimetry chamber compared to DLW measured for 14 days (Unstructured). | Mean_DIFF_ Men = +4.5 (SD = 14.4)%; (-9.0 – 32.3%)  Mean_DIFF_ Women = +5.9 (SD = 8.8)%; (-4.5-16.2%)  Bland Altman | Kowalski (2012) |
| Racette et al. (1995) ^[98]^ | Polar Vantage XL | 21-47 yrs. (obese) | 14 | F = 14 | Heart Rate determined TDEE (Individual Calibration; linear regression lines; Factorial method) – Doubly labelled water determined TDEE.  3 days HRM compared to 14 days DLW TDEE (Unstructured). | Mean_DIFF_=+0.9%  Bland Altman | Ainslie (2003) |
| Rafamantanantsoa et al. (2002) ^[99]^ | Accurex Plus | 48 ± 10 yrs. | 24 | M = 24 | HRM determined energy expenditure (Individual Calibration; FLEX HR) – Doubly labelled water determined energy expenditure.  Heart Rate Monitor worn for 3 day period within the 14 day examination while DLW was measure over the same period (Unstructured). | r_p_=0.67; p<0.001; Mean Diff= 57 ±603kcal/d;  Bland Altman | Plasqui (2007) |
| Rotherberg et al. (1998) ^[86]^ | Heart Rate Monitor | 73 yrs. | 20 | M = 8  F = 12 | Heart rate monitor determined energy expenditure (Individual Calibration; (MJ.d^-1^) – Doubly labelled water determined energy expenditure (MJ.d^-1^)  DLW measured for 20 days compared with 4 days of heart rate monitoring (Unstructured). | Mean_DIFF_=-0.96 ± 1.14 MJ.d^-1^; p<0.05; Mean%_DIFF_ =-10% ± 12%; r_s_=0.76; p<0.005  Bland Altman | Prince (2008) |
| Schulz et al. (1989) ^[101]^ | Heart Rate Monitor | NA | 6 | M = 4  F = 2 | Heart rate monitoring (FLEX HR) determined energy expenditure – Doubly labelled water energy expenditure  1 day of free-living physical activity behaviours using FLEX HR calibration curves (Unstructured) | r=0.53-0.73; p<0.05 | Ainslie (2003) |

DLW = Doubly Labelled Water; HR = Heart Rate; HRM = Heart Rate Monitor; TEE = Total Energy Expenditure; Mean_DIFF_ = Mean Difference; SD = Standard Deviation; r = Correlation Coefficient; CV = Coefficient of Variation; RMSE = Root Mean Squared Error; CI = Confidence Intervals; LoA = Limits of Agreement; NS = Non-significant.

**Supplementary Table 17.** Details of studies examining the concurrent validity of heart rate monitoring devices.

| **Author** | **Measure** | **Age Range** | **Sample Size** | **Sex** | **Reference** | **Results** | **Primary Source** |
| --- | --- | --- | --- | --- | --- | --- | --- |
| Ceesay et al. (1989) ^[466]^ | Sports Tester PE3000 HR monitor | 17-36 yrs. | 20 | M = 11  F = 9 | Individual Heart Rate calibration curves determined total energy expenditure (FLEX HR) – Whole room calorimeter determined total energy expenditure.  Structured laboratory based activities (21.5 hours) (Structured). | r=0.94  HR Mean underestimation = 1.2 ± 6.2 %; | Bassett et al. (2000) |
| Fuller et al. (2008) ^[79]^ | Polar Sports Tester | 20-66 yrs. | 60 | M = 30  F = 30 | Heart rate monitor determined energy expenditure (MJ.d^-1^) (from calibrated FLEX HR) – Caltrac determined energy expenditure (MJ.d^-1^)  Heart rate monitor determined energy expenditure (MJ.d^-1^) (from calibrated FLEX HR) – PAD determined energy expenditure (MJ.d^-1^)  Heart rate monitor determined energy expenditure (MJ.d^-1^) (from calibrated FLEX HR) –7DPARQ determined energy expenditure (MJ.d^-1^)  15 days of DLW measurement, HRM, Physical Activity Diary, Caltrac device and 2 versions of the 7DPARQ (Day 8 and day 15 (Unstructured). | r=0.68; p<0.01  r=0.54; p<0.01  r=0.51; p<0.01 | Plasqui (2013) |
| Fuller et al. (2008) ^[79]^ | Polar Sports Tester | 20-66 yrs. | 60 | M = 30  F = 30 | Heart rate monitor determined energy expenditure (PAL) (from calibrated FLEX HR) – Caltrac determined energy expenditure (PAL)  Heart rate monitor determined energy expenditure (PAL) (from calibrated FLEX HR) – PAD determined energy expenditure (PAL)  Heart rate monitor determined energy expenditure (PAL) (from calibrated FLEX HR) –7DPAR determined energy expenditure (PAL)  15 days of DLW measurement, HRM, Physical Activity Diary, Caltrac device and 2 versions of the 7DPARQ (Day 8 and day 15 (Unstructured). | r=0.45; p<0.01  r=0.00; NS  r=0.03; NS | Plasqui (2013) |
| Leonard et al. (1995) ^[467]^ | Polar Vantage XL | M; 38±16 yrs.  F; 37±14 yrs. | 32 | M = 16  F = 16 | Heart rate monitoring (FLEX HR) determined TDEE MJ/d– Heart rate monitoring (Factorial method) determined TDEE MJ/d.  Free living activity behaviours for 16-18 hours (Unstructured). Factorial method more likely to underestimate TDEE. | r=0.759; p<0.01  Mean_DIFF_=0.95-2.86 MJ (males)  Mean_DIFF_=0.32-1.31 MJ (males)  Bland Altman | Chen (2005) |
| MacFarlane et al (2006) ^[145]^ | Team system, Polar Oy, | 15-55 yrs. | 49 | M = 30  F = 19 | Heart Rate Monitor determined activity intensity (Howley cut points) – Tritrac determined activity intensity (Nichols cut points)  Free-living physical activity behaviours over 7 days using each measure (Unstructured). | r_s Light_=0.10  r_s Moderate_=0.14  r_s Vigorous_=0.36; p<0.05  r_s_ HEPA=0.27; NS | Andrew (2010) |
| MacFarlane et al (2006) ^[145]^ | Team system, Polar Oy, | 15-55 yrs. | 49 | M = 30  F = 19 | Heart Rate Monitor determined activity intensity (Howley cut points) – MTI determined activity intensity (Freedson cut points)  Free-living physical activity behaviours over 7 days using each measure (Unstructured). | r_s Light_=0.14  r_s Moderate_= 0.24; p<0.05  r_s Vigorous_=0.63; p<0.01  r_s_ HEPA=0.46; p<0.01 | Andrew (2010) |
| Spurr et al. (1998) ^[468]^ | Heart Rate Monitor | 18-66 yrs. | 22 | M = 16  F = 6 | Heart Rate Monitor determined energy expenditure (KJ/min) (individual calibrations) – Whole room Calorimetry determined energy expenditure (KJ/min).  Participants spent 22 hrs in a whole room calorimeter while wearing the HRM. | Mean_DIFF_=0.24 MJ;  r=0.87; SEE=0.91 MJ | Ainslie (2003) |
| Strath et al. (2000) ^[469]^ | Polar Heart Rate Monitor | 19-74 yrs. | 61 | M = 31  F = 30 | Heart Rate determined energy expenditure (after adjusting for age and fitness) - Indirect Calorimetry determined energy expenditure  Participants wore HRM and had measures of indirect calorimetry obtained during lab and field based MVPA activities (Structured). | r=0.87; Mean error = 0.04 METs; 95% CI =-1.48 – 1.56  Bland Altman | Bassett et al (2000) |
| Swan et al. (1997) ^[400]^ | Heart Rate Monitor | 22.6 ± 5 yrs. | 31 | F = 31 | Heart Rate determined energy expenditure – Caltrac determined energy expenditure  Treadmill based walking, running and stepping (Structured) | r=0.58-0.78^*^; ^*^p<0.05. | Ainslie (2003) |
| Theou et al. (2012) ^[173]^ | Polar Wearlink 31 | 63-90 yrs. | 50 | F = 50 | Heart Rate Monitor determined time engaged in non-sedentary activities - ActiTrainer determined time engaged in non-sedentary activities.  Heart Rate Monitor determined time engaged in non-sedentary activities - MLTPAS total time spent across all activities.  10 hours of free-living activity behaviours (ActiTrainer and Heart Rate Monitor) and 14 days of self-reported PA (MLTPAS) (Unstructured). | r_p_=0.26; NS  r_p_=0.20; NS | Gorman (2014) |

HR = Heart Rate; HRM = Heart Rate Monitor; PAD = Physical Activity Diary; 7DPARQ = 7 Day Physical Activity Recall Questionnaire; MLTPAS = Minnesota Leisure Time Physical Activity Survey; PA = Physical Activity; PAL = Physical Activity Level; TDEE = Total Daily Energy Expenditure; HEPA = Health Enhancing Physical Activity; METs = Metabolic Equivalents; r = Correlation Coefficient; r_p_ = Pearson’s Correlation; r_s_ = Pearson’s Correlation; NS = Non-significant; SEE = Standard Error of the Estimate; Mean_DIFF_ = Mean Difference.

**Supplementary Table 18.** Details of studies examining the criterion validity of combined sensors

| **Author** | **Measure** | **Age Range** | **Sample Size** | **Sex** | **Reference** | **Results** | **Primary Source** |
| --- | --- | --- | --- | --- | --- | --- | --- |
| Foerster et al. (1999)^[471]^ | 4 IC Sensor Model 3031 | 21-34 yrs. | 24 | M = 24 | Sensors worn at the sternum, wrist, thigh and lower leg determined activity classification (specific modelling) – Direct observation.  9 activities ranging from sitting, cycling and stair ascending/descending and then validated in free-living sample (Structured). | Accuracy = 95.8% | Bonomi (2012) |
| Foerster et al. (2000)^[470]^ | Multiple IC Sensor Model 3031 | 20-32 yrs. | 31 | M = 25  F = 6 | Multisensor determined postures and movement classification – Direct Observation.  3 uniaxial sensors worn on the sternum and two worn on the front right and left thigh during 13 free-living physical activities (Structured). | %_ERROR_=3.2% | Chen (2005) |
| Johansson et al. (2006)^[370]^ | Combined accelerometer and Heart Rate monitor | M: 32.0 ± 12.8 yrs.  F: 26.6 ± 5.3 yrs.  M: 28-63 yrs.  F: 28-30 yrs. | 27  8 | M = 14  F = 13  M=6  F=2 | Combined ACC and HR regression equation determined total energy expenditure (MJ.d^-1^) – Doubly Labelled Water determined total energy expenditure (MJ.d^-1^).  Combined ACC and HR regression equation determined physical activity energy expenditure (kJ.kg.d^-1^) – Doubly Labelled Water determined total energy expenditure (kJ.kg.d^-1^).  Participants wore the HRM and the ActiGraph MTI during model development with indirect Calorimetry while completing walking and running activities on the treadmill (Structured), while 8 participants wore HRM and ACC during free-living activities for 14 days with DLW measurement (Unstructured). | RMSE=2.99; Sig Diff from 0.  RMSE=31.1; Sig Diff from 0. | Van Remoortel (2012) |
| Lau et al. (2008)^[474]^ | Combined duel axis accelerometer and gyroscope | 25-26 yrs. | 3 | M = 3 | Combined device determined activities (based on Support vector machine learning for 5 class) – Direct Observation.  Combined device determined activities (based on Support vector machine learning for 2 class) – Direct Observation.  Walking under 5 different walking conditions (treadmill upslope, treadmill downslope, over ground, stair ascending, stair descending). | Accuracy SVM 5 class = 84.71%  Accuracy SVM 2 class = 100% | Cheung (2011) |
| Ng et al. (2003)^[475]^ | Combined Accelerometry and 2 channel ECG data | NA | 10 | NA | Accelerometer and ECG determined activity classification – Direct Observation.  A range of free-living activities including sit-to-stand transitions and ambulatory activities (Structured). | Sensitivity = 95.5%-99.2%  Specificity = 99.0%-99.8% | Cheung (2011) |
| Nyan et al. (2006)^[472]^ | Multiple accelerometers worn on a garment | 20-45 yrs. | 22 | NA | Combined accelerometer determined activity classification – Direct Observation.  Over ground walking, stair ascending and descending (structured). | Sensitivity ascending stairs = 93-100%  Specificity ascending stairs = 99-100%  Sensitivity descending stairs = 37-100%  Specificity descending stairs = 98-100% | Cheung (2011) |
| Parkka et al. (2006)^[473]^ | Multiple Sensors | 25.8±4.3 yrs. | 16 | M = 13  F = 2 | Combined sensor determined activity classification (using Decision Tree Classification, Automatically Generated Decision Tree and Artificial Neural Networks)– Direct Observation.  A range of free-living activities including sitting, standing and ambulatory activities (Unstructured) | DTC accuracy = 82%  AGDTC accuracy = 86%  ANN accuracy = 82% | Cheung (2011) |
| Rumo et al. (2011)^[476]^ | Combined HR and 2 accelerometer devices (hip and dominant wrist) | 31 ± 9 yrs. | 12 | M = 6  F = 5 | HR+ACC determined activity type – Direct Observation determined activity type (Class dependent approach).  7 activities performed, and compared between indirect Calorimetry and HR+ACC during lab based conditions (from developed/trained algorithms) (Structured). | %Accuracy=63% | Liu (2012) |
| Rumo et al. (2011)^[476]^ | Combined HR and 2 accelerometer devices (hip and dominant wrist) | 31 ± 9 yrs. | 12 | M = 6  F = 5 | HR+ACC determined activity type – Direct Observation determined activity type (Class dependent approach).  2 hours of simulated free-living activities in the laboratory based on developed/trained algorithms (Structured). | %Accuracy=78% | Liu (2012) |
| Rumo et al. (2011)^[476]^ | Combined HR and 2 accelerometer devices (hip and dominant wrist) | 31 ± 9 yrs. | 12 | M = 6  F = 5 | HR+ACC determined energy expenditure (kcal.day^-1^) – Doubly Labelled Water determined energy expenditure (kcal.day^-1^).  HR+ACC determined energy expenditure (kcal.day^-1^) – Doubly Labelled Water determined energy expenditure (kcal.day^-1^) (Class dependent approach).  HR+ACC determined energy expenditure (kcal.day^-1^) – Doubly Labelled Water determined energy expenditure (kcal.day^-1^) (Class independent linear model).  HR+ACC determined energy expenditure (kcal.day^-1^) – Doubly Labelled Water determined energy expenditure (kcal.day^-1^) (Class independent nonlinear model).  1 week of energy expenditure from free-living activities recorded using DLW compared to HR+ACC energy expenditure based on developed/trained algorithms (Structured). | r=0.88  %Accuracy=87%  %Accuracy =71%  %Accuracy =59% | Liu (2012) |
| Rumo et al. (2011)^[476]^ | Combined HR and 2 accelerometer devices (hip and dominant wrist) | 31 ± 9 yrs. | 12 | M = 6  F = 5 | HR+ACC determined energy expenditure (kcal.day^-1^) – Doubly Labelled Water determined energy expenditure (kcal.day^-1^).  HR+ACC determined energy expenditure (kcal.day^-1^) – Doubly Labelled Water determined energy expenditure (kcal.day^-1^) (Class dependent approach).  HR+ACC determined energy expenditure (kcal.day^-1^) – Doubly Labelled Water determined energy expenditure (kcal.day^-1^) (Class independent linear model).  HR+ACC determined energy expenditure (kcal.day^-1^) – Doubly Labelled Water determined energy expenditure (kcal.day^-1^) (Class independent nonlinear model).  2 weeks of energy expenditure from free-living activities recorded using DLW compared to HR+ACC energy expenditure based on developed/trained algorithms (Structured). | r=0.98  %Accuracy=91%  %Accuracy =68%  %Accuracy =52% | Liu (2012) |

HR = Heart Rate; ACC = Accelerometer; HRM = Heart Rate Monitor; DLW = Doubly Labelled Water; SVM = Sum of the Vector Magnitude; DTC = Decision Tree Classification, AGDT = Automatically Generated Decision Tree; ANN = Artificial Neural Networks; RMSE = Root Mean Squared Error; r = Correlation Coefficient.

**Supplementary Table 19.** Details of studies examining the concurrent validity of combined sensors.

| **Author** | **Measure** | **Age Range** | **Sample Size** | **Sex** | **Reference** | **Results** | **Primary Source** |
| --- | --- | --- | --- | --- | --- | --- | --- |
| Brage et al. (2004)^[477]^ | ActiGraph (CSA) and Polar Electro Heart Rate Monitoring | 22.7-30 yrs. | 12 | M = 12 | Combined HR and CSA (branched prediction equation developed) – Whole room Calorimetry determined PAEE kJ/Kg.  Prediction equation developed using HRM and CSA data collected during 24 hour WRC stay (Structured). | R^2^=0.78; p<0.001  Error individual (measured – predicted) = -1.5 ± 13%  Error group (measured – predicted) = 0.1 ± 9.8% | Butte (2012) |
| Edwards et al. (2010)^[480]^ | Wireless Device with simultaneous HR and Accelerometry | 18-48 yrs. | 20 | M = 10  F = 10 | WHA determined physical activity energy expenditure (using flex heart rate prediction model) – Indirect Calorimetry determined physical activity energy expenditure.  WHA determined physical activity energy expenditure (using flex acceleration prediction model) – Indirect Calorimetry determined physical activity energy expenditure.  WHA determined physical activity energy expenditure (HR and ACC data from calibration study fit via multiple linear regression model) – Indirect Calorimetry determined physical activity energy expenditure.  WHA determined physical activity energy expenditure (Brages branched algorithm) – Indirect Calorimetry determined physical activity energy expenditure.  WHA determined physical activity energy expenditure (Brages branched algorithm using optimal parameters gaining the lowest RMS) – Indirect Calorimetry determined physical activity energy expenditure.  WHA determined physical activity energy expenditure (Brages branched algorithm unconstrained) – Indirect Calorimetry determined physical activity energy expenditure.  Data collected during treadmill calibration protocol and then applied to “free-living” protocol (Structured). | Individual = RMSE for HR were 59% of mean criterion PA;  Group = RMSE for HR were 63% of mean criterion PA; R^2^=0.60; p<0.05  Individual = RMSE for ACC were 53% of mean criterion PA;  Group = RMSE for ACC were 64% of mean criterion PA; R^2^=0.01; NS  Individual; r=0.38  Group; RMSE for HR were 26% of mean criterion PA; r=0.64  Individual = RMSE for ACC were 42% of mean criterion PA;  Group = RMSE for ACC were 45% of mean criterion PA;  Individual = RMSE for ACC were 16% of mean criterion PA;  Group = RMSE for ACC were 18% of mean criterion PA;  Individual = RMSE for ACC were 12% of mean criterion PA;  Group = RMSE for ACC were 14% of mean criterion PA; | Liu (2012) |
| Haskell et al. (1993)^[481]^ | Combined Vitalog and Heart Rate Monitoring | 22-64 yrs. | 19 | M = 19 | Combined regression equations including both VL and HRM determined ml.Kg.min – Indirect Calorimetry determined ml.Kg.min  Walking, and running at various speeds on a treadmill, Arm cranking, cycling, Air Dyne and Bench step (Structured). | R^2^_individuak_ =0.89  SEE = 2.3 ml.Kg.min; Range = 1.36-3.16.  R^2^_all_=0.73; SEE=5.2 ml.Kg.min | Dishman (2001) |
| Luke et al. (1997)^[482]^ | Combined Heart Rate (Escort 200T) and Motion (AMS-1000) | 29±5 yrs. | 10 | M = 2  F = 8 | Combined heart rate and motion sensor predicted VO_2_·ml·min^-1^ – Indirect Calorimetry determined VO_2_·ml·min^-1^.  Predicted VO_2_·ml·min^-1^ from pooled data for combined HRM and Motion counts.  Combination of activities of daily living circuit and treadmill test (Structured). | R_ADLC_ =0.86 (±0.09); R_Tread_ =0.90 (±0.05)  R^2^=0.74 (SEE = 4.16 ml·kg^-1^·min^-1^) | Haskell (2000) |
| Meijer et al. (1989)^[483]^ | Heart Rate and accelerometer | 20-40 yrs. | 16 | M = 13  F == 3 | Heart rate monitoring and accelerometry determined energy expenditure (linear regression) – Indirect Calorimetry determined energy expenditure (J/min/kg).  Structured activities including sitting, standing and treadmill based activities (Structured). | SEE = 79.1 J/min/kg. | Ainslie (2003) |
| Moon et al. (1996)^[478]^ | Mini-mitter and Heart Rate Monitoring | 19-40 yrs. | 20 | M = 10  F = 10 | Combined HR and Mini-mitter determined VO2 and VCO2 (prediction equation developed) – Whole room Calorimetry determined VO2 and VCO2.  Prediction equation developed using HRM and Mini-mitter data collected during 24 hour WRC stay (Structured). | Error (measured – predicted) = -3.4 - -7.8 | Butte (2012) |
| Rennie et al. (2000) ^[479]^ | Combined Heart Rate and Activity Monitor | 31.3 (23-54) yrs. | 8 | M = 5  F = 3 | HR+M determined energy expenditure (kJ) – Whole Room Calorimetry determined energy expenditure (kJ).  HR determined energy expenditure (kJ) – Whole Room Calorimetry determined energy expenditure (kJ).  Participants wore the combined device while spending 24 hours in a while room calorimeter (Structured). | %_DIFF_=0.00 ± 12.5 %; Mean_DIFF_ =7.78 ± 821.7 kJ.  %_DIFF_= 16.5 ± 30.2 %; Mean_DIFF_ =1060.6 ± 1945.4 kJ. | Warren (2010) |
| Rumo et al. (2011)^[476]^ | Combined HR and 2 accelerometer devices (hip and dominant wrist) | 31 ± 9 yrs. | 12 | M = 6  F = 5 | HR+ACC determined energy expenditure (kJ.min^-1^) – Indirect Calorimetry determined energy expenditure (kJ.min^-1^) (Class dependent approach).  HR+ACC determined energy expenditure (kJ.min^-1^) – Indirect Calorimetry determined energy expenditure (kJ.min^-1^) (Class independent linear model).  HR+ACC determined energy expenditure (kJ.min^-1^) – Indirect Calorimetry determined energy expenditure (kJ.min^-1^) (Class independent nonlinear model).  7 activities performed, and compared between indirect Calorimetry and HR+ACC during lab based conditions (from developed/trained algorithms) (Structured). | Mean_ERROR_=12%  Mean_ERROR_=10%  Mean_ERROR_=13% | Liu (2012) |
| Rumo et al. (2011)^[476]^ | Combined HR and 2 accelerometer devices (hip and dominant wrist) | 31 ± 9 yrs. | 12 | M = 6  F = 5 | HR+ACC determined energy expenditure (kJ.min^-1^) – Indirect Calorimetry determined energy expenditure (kJ.min^-1^) (Class dependent approach).  HR+ACC determined energy expenditure (kJ.min^-1^) – Indirect Calorimetry determined energy expenditure (kJ.min^-1^) (Class independent linear model).  HR+ACC determined energy expenditure (kJ.min^-1^) – Indirect Calorimetry determined energy expenditure (kJ.min^-1^) (Class independent nonlinear model).  2 hours of simulated free-living activities in the laboratory based on developed/trained algorithms (Structured). | %Accuracy=55%  %Accuracy =47%  %Accuracy =39% | Liu (2012) |
| Strath et al. (2001)^[399]^ | Combined heart rate (Polar) and motion sensor (ActiGraph CSA). | M 33.1± 12.2 yrs.  F31.9± 13.1 yrs. | 30 | M = 16  F = 14 | Combined CSA and heart rate monitoring determined METs – Indirect Calorimetry determined METs.  Continuous monitoring using HR, motion sensing, pedometers and indirect Calorimetry during laboratory based conditions (Structured). | R^2^=0.81; p<0.01 | Butte (2012) |
| Strath et al. (2002)^[484]^ | Combined heart rate (Polar) and motion sensor (ActiGraph CSA). | M 26.7±1.5 yrs.  F 26.5±3.3 yrs. | 10 | M = 4  F = 6 | Arm and leg worn motion sensor with heart rate monitoring determined METs – Indirect Calorimetry determined METs.  6 hrs. of continuous monitoring of HR, motion sensing and indirect Calorimetry (Unstructured). | R^2^=0.79-0.89; p<0.01 | Ainslie (2003) |
| Strath et al. (2005)^[485]^ | Polar HR monitor and ActiGraph 7164 | 25.8 ± 3.4 yrs. | 10 | M = 4  F = 6 | ActiGraph determined physical activity energy expenditure (Freedson equation) – Indirect Calorimetry determined physical activity energy expenditure.  ActiGraph and HR determined physical activity energy expenditure (Arm-Leg heart rate motion sensor technique; individual calibration) – Indirect Calorimetry determined physical activity energy expenditure.  ActiGraph and HR determined physical activity energy expenditure (Brage branched equation model; individual calibration) – Indirect Calorimetry determined physical activity energy expenditure.  ActiGraph and HR determined physical activity energy expenditure (Arm-Leg heart rate motion sensor technique; group calibration) – Indirect Calorimetry determined physical activity energy expenditure.  ActiGraph and HR determined physical activity energy expenditure (Brage branched equation model; group calibration) – Indirect Calorimetry determined physical activity energy expenditure.  Calibration from arm crank and treadmill based activities, with calibrations completed for both individuals and groups. 6hrs of free-living data available for validation (including indirect Calorimetry (Structured). | R^2^=0.41; SEE = 0.96 METs.  R^2^=0.81; SEE = 0.55 METs.  R^2^=0.75; SEE = 0.61 METs.  R^2^=0.67; SEE = 0.88 METs.  R^2^=0.75; SEE = 0.67 METs. | Liu (2012) |

HR = Heart Rate; ACC = Accelerometer; CSA= Computer Science Applications accelerometer; HRM = Heart Rate Monitor; PA = Physical Activity; PAEE = Physical Activity Energy Expenditure; METs = Metabolic Equivalents; M= Male; F = Female; RMSE = Root Mean Squared Error; NS = Non-significant; SEE = Standard Error of the Estimate; r = Correlation Coefficient; Mean_DIFF_ = Mean Difference.

**Supplementary Figure 1. PRISMA Checklist**

| **Section/topic** | **#** | **Checklist item** | **Reported on page #** |
| --- | --- | --- | --- |
| **TITLE** | | |  |
| Title | 1 | Identify the report as a systematic review, meta-analysis, or both. | 1 |
| **ABSTRACT** | | |  |
| Structured summary | 2 | Provide a structured summary including, as applicable: background; objectives; data sources; study eligibility criteria, participants, and interventions; study appraisal and synthesis methods; results; limitations; conclusions and implications of key findings; systematic review registration number. | 2 |
| **INTRODUCTION** | | |  |
| Rationale | 3 | Describe the rationale for the review in the context of what is already known. | 3 |
| Objectives | 4 | Provide an explicit statement of questions being addressed with reference to participants, interventions, comparisons, outcomes, and study design (PICOS). | 3 |
| **METHODS** | | |  |
| Protocol and registration | 5 | Indicate if a review protocol exists, if and where it can be accessed (e.g., Web address), and, if available, provide registration information including registration number. | 4 |
| Eligibility criteria | 6 | Specify study characteristics (e.g., PICOS, length of follow-up) and report characteristics (e.g., years considered, language, publication status) used as criteria for eligibility, giving rationale. | 4-5 |
| Information sources | 7 | Describe all information sources (e.g., databases with dates of coverage, contact with study authors to identify additional studies) in the search and date last searched. | 4 |
| Search | 8 | Present full electronic search strategy for at least one database, including any limits used, such that it could be repeated. | 4 |
| Study selection | 9 | State the process for selecting studies (i.e., screening, eligibility, included in systematic review, and, if applicable, included in the meta-analysis). | 4-5 |
| Data collection process | 10 | Describe method of data extraction from reports (e.g., piloted forms, independently, in duplicate) and any processes for obtaining and confirming data from investigators. | 4-5 |
| Data items | 11 | List and define all variables for which data were sought (e.g., PICOS, funding sources) and any assumptions and simplifications made. | 4-5 |
| Risk of bias in individual studies | 12 | Describe methods used for assessing risk of bias of individual studies (including specification of whether this was done at the study or outcome level), and how this information is to be used in any data synthesis. | NA |
| Summary measures | 13 | State the principal summary measures (e.g., risk ratio, difference in means). | NA |
| Synthesis of results | 14 | Describe the methods of handling data and combining results of studies, if done, including measures of consistency (e.g., I^2^) for each meta-analysis. | NA |

| **Section/topic** | **#** | **Checklist item** | **Reported on page #** |
| --- | --- | --- | --- |
| Risk of bias across studies | 15 | Specify any assessment of risk of bias that may affect the cumulative evidence (e.g., publication bias, selective reporting within studies). | NA |
| Additional analyses | 16 | Describe methods of additional analyses (e.g., sensitivity or subgroup analyses, meta-regression), if done, indicating which were pre-specified. | NA |
| **RESULTS** | | |  |
| Study selection | 17 | Give numbers of studies screened, assessed for eligibility, and included in the review, with reasons for exclusions at each stage, ideally with a flow diagram. | 6, 31 |
| Study characteristics | 18 | For each study, present characteristics for which data were extracted (e.g., study size, PICOS, follow-up period) and provide the citations. | 16-28, 37-290 |
| Risk of bias within studies | 19 | Present data on risk of bias of each study and, if available, any outcome level assessment (see item 12). | NA |
| Results of individual studies | 20 | For all outcomes considered (benefits or harms), present, for each study: (a) simple summary data for each intervention group (b) effect estimates and confidence intervals, ideally with a forest plot. | NA |
| Synthesis of results | 21 | Present results of each meta-analysis done, including confidence intervals and measures of consistency. | NA |
| Risk of bias across studies | 22 | Present results of any assessment of risk of bias across studies (see Item 15). | NA |
| Additional analysis | 23 | Give results of additional analyses, if done (e.g., sensitivity or subgroup analyses, meta-regression [see Item 16]). | NA |
| **DISCUSSION** | | |  |
| Summary of evidence | 24 | Summarize the main findings including the strength of evidence for each main outcome; consider their relevance to key groups (e.g., healthcare providers, users, and policy makers). | 12-14 |
| Limitations | 25 | Discuss limitations at study and outcome level (e.g., risk of bias), and at review-level (e.g., incomplete retrieval of identified research, reporting bias). | 12-14 |
| Conclusions | 26 | Provide a general interpretation of the results in the context of other evidence, and implications for future research. | 12-14 |
| **FUNDING** | | |  |
| Funding | 27 | Describe sources of funding for the systematic review and other support (e.g., supply of data); role of funders for the systematic review. | 15 |

*From:* Moher D, Liberati A, Tetzlaff J, Altman DG, The PRISMA Group (2009). Preferred Reporting Items for Systematic Reviews and Meta-Analyses: The PRISMA Statement. PLoS Med 6(6): e1000097. doi:10.1371/journal.pmed1000097

For more information, visit: **www.prisma-statement.org**

† denotes a mean percentage differences greater than 250%. For the purposes of forest plot presentations, all values greater than 250% were set at this value.

Activity Log/Diary time spent in Physical Activity

Activity Log/Diary determined Energy Expenditure

**Supplementary Figure 2a.** Forest plot of percentage mean difference between self-report measures of energy expenditure compared to energy expenditure from activity logs/diaries.

Self-reported Energy Expenditure

**Supplementary Figure 2b.** Forest plot of percentage mean difference between self-report measures of energy expenditure and time spent in physical activity compared to other self-report measures of energy expenditure or time spent in physical activity.

Self-reported time spent in Physical Activity

**Supplementary Figure 2c.** Forest plot of percentage mean difference between self-report measures of energy expenditure compared to energy expenditure determined from activity monitors.

Moderate-to-Vigorous Intensity Physical Activity

Light Intensity Physical Activity

Moderate Intensity Physical Activity

^† denotes a mean percentage differences greater than 250%. For forest plot presentations, all values greater than 250% were set at this value.^

Moderate-to-Vigorous Intensity Physical Activity

**Supplementary Figure 2d.** Forest plot of percentage mean difference between self-report measures of time spent in physical activity intensities (Light, Moderate and Moderate-to-Vigorous intensity physical activity) compared to time spent in physical activity intensities determined from activity monitors (TBC).

^† denotes a mean percentage differences greater than 250%. For the purposes of forest plot presentations, all values greater than 250% were set at this value.^

Vigorous Intensity Physical Activity

Additional Physical Activity Variables

**Supplementary Figure 2e.** Forest plot of percentage mean difference between self-report measures of time spent in physical activity intensities (Vigorous physical activity, Total physical activity, times active, time standing, time stepping) compared to time spent in physical activity intensities determined from activity monitors.

Pedometer determined Energy Expenditure

Heart Rate Monitor determined Energy Expenditure

Heart Rate Monitor determined time spent in Physical Activity Intensities

^† denotes a mean percentage differences greater than 250%. For the purposes of forest plot presentations, all values greater than 250% were set at this value.^

**Supplementary Figure 2f.** Forest plot of percentage mean difference between self-report measures of energy expenditure and time spent in physical activity intensities (Vigorous physical activity, Total physical activity, times active, time standing, time stepping) compared to energy expenditure time spent in physical activity intensities determined from pedometers and heart rate monitors.

Moderate Intensity Physical Activity

Moderate-to-vigorous Intensity Physical Activity

Vigorous Intensity Physical Activity

Total Activity

Stepping

Energy Expenditure

Step Counts

Light Intensity Physical Activity

Activity Counts

**Supplementary Figure 3c.** Forest plot of percentage mean difference between accelerometer/activity monitor determined variables (activity counts, time spent in light intensity physical activity, time spent in moderate intensity physical activity, time spent in moderate-to-vigorous intensity physical activity, time spent in vigorous intensity physical activity, total physical activity, stepping and energy expenditure) compared to an alternative accelerometer/activity monitor.

Total EE (WRC)

EE from Vigorous Intensity Physical Activity (IC)

EE from Total Physical Activity (IC)

EE from Moderate Intensity Physical Activity (IC)

EE from Light Intensity Physical Activity (IC)

**Supplementary Figure 3d.** Forest plot of percentage mean difference between accelerometer/activity monitor determined energy expenditure (METs) in light intensity physical activity, moderate intensity physical activity, vigorous intensity physical activity and total physical activity (METs, MJ.d, KJ.h, KJ.kg.min^-1^) compared to estimates from indirect (IC) and whole room calorimetry (WRC).

Total EE (HRM)

EE from Light Intensity Physical Activity (HRM)

EE from Moderate Intensity Physical Activity (HRM)

EE from Vigorous Intensity Physical Activity (HRM)

EE from Total Physical Activity (HRM)

EE from Total Physical Activity (SR)

**Supplementary Figure 3e.** Forest plot of percentage mean difference between accelerometer/activity monitor determined energy expenditure, energy expenditure from light intensity physical activity, moderate intensity physical activity, vigorous intensity physical activity, total physical activity compared to estimates from Heart Rate Monitoring (HRM) and Self-Report (SR) measures.

**Supplementary Figure 3f.** Forest plot of percentage mean difference between accelerometer/activity monitor determined energy expenditure (kcal.min^-1^, kcal.kg.hr^-1^) compared to indirect calorimetry determined energy expenditure (kcal.min^-1^, kcal.kg.hr^-1^).

**Supplementary Figure 3g.** Forest plot of percentage mean difference between accelerometer/activity monitor determined energy expenditure (METs.min^-1^, METs.hr^-1^) compared to indirect calorimetry determined energy expenditure (METs.min^-1^, METs.hr^-1^).

**Supplementary Figure 3h.** Forest plot of percentage mean difference between accelerometer/activity monitor determined total energy expenditure compared to indirect calorimetry determined total energy expenditure.

**Supplementary Figure 3h (Cont).** Forest plot of percentage mean difference between accelerometer/activity monitor determined energy expenditure (kcal.min^-1^, kcal.kg.hr^-1^) compared to indirect calorimetry determined energy expenditure (kcal.min^-1^, kcal.kg.hr^-1^).

* denotes multiple devices compared in the same study; † denotes multiple equations for accelerometer determined energy expenditure/MVPA compared to pedometer determined energy expenditure/MVPA.

Accelerometer (Steps)

Accelerometer Energy Expenditure

Accelerometer (MVPA)

Pedometer (Steps)

**Supplementary Figure 4a.** Forest plot of percentage mean difference between pedometer determined step count/energy expenditure/MVPA compared to concurrent measures (i.e. accelerometry, indirect calorimetry, pedometers).

^† denotes a mean percentage differences greater than 250%. For the purposes of forest plot presentations, all values greater than 250% were set at this value.^

Physical Activity Intensity

Physical Activity Level (PAL)

Energy Expenditure

**Supplementary Figure 5a.** Forest plot of percentage mean difference between heart rate monitor determined energy expenditure/physical activity level/physical activity intensity compared to concurrent measures (accelerometers, self-report, indirect calorimetry)

**Supplementary** **Figure 6.** Forest plot of percentage mean difference between energy expenditure determined by combined sensors compared to concurrent measure (indirect calorimetry).
